# Cytotoxic Peptidic Metabolites Isolated from the Soil-Derived Fungus *Trichoderma atroviride*

**DOI:** 10.3390/molecules30163422

**Published:** 2025-08-19

**Authors:** Jun Gu Kim, Jae Sang Han, Dahyeon Lee, Mi Kyeong Lee, Bang Yeon Hwang, Jin Woo Lee

**Affiliations:** 1 College of Pharmacy, Chungbuk National University, Cheongju 28160, Republic of Korea; sossgi@naver.com (J.G.K.); han00768@hanmail.net (J.S.H.); mklee@chungbuk.ac.kr (M.K.L.); 2 College of Pharmacy, Duksung Women’s University, Seoul 01369, Republic of Korea; 99dian@naver.com; 3 Department of AI Drug Discovery, Duksung Women’s University, Seoul 01369, Republic of Korea

**Keywords:** peptaibols, soil fungus, *Trichoderma atroviride*, structure elucidation, cytotoxic activity

## Abstract

Twelve undescribed peptidic compounds, bukhansantaibols A–K (**1**–**10**) and bukhansantaibals A–B (**11**–**12**), were isolated from the soil fungus *Trichoderma atroviride* through LC-MS and bioactivity-guided purification. Their structures were elucidated by the analysis of 1D and 2D NMR spectra, HRESIMS, and acid hydrolysis using modified Marfey’s method. All compounds were evaluated for their cytotoxic activity against HCT-8 (colon cancer) and SK-OV-3 (ovarian cancer) cells. Among them, compounds **1**–**5** exhibited significant inhibitory effects, with IC_50_ values ranging from 2.1 to 19.6 μM.

## 1. Introduction

Peptaibols are fungal secondary metabolites produced by non-ribosomal peptide synthetases (NRPSs) and constitute a class of linear peptides characterized by N-terminal acetylation, the incorporation of non-proteinogenic amino acids, and a C-terminal amino alcohol [1,2]. Peptaibols are mainly produced by fungi belonging to the order Hypocreales, which includes culturable genera such as *Trichoderma* and *Escovopsis* [3]. Recently, the weeding behaviour of the leaf-cutting fungus-growing ant *Trachymyrmex septentrionalis* was reported to be triggered by the detection of peptaibols [4]. The production of peptaibol compounds during mycoparasitic interactions has inspired studies on their antimicrobial properties, which have been experimentally validated in various systems [5,6]. Their proposed mechanism of action—formation of transmembrane ion channels leading to cell lysis—has also prompted investigations into their cytotoxic potential against a range of cancer cells [7,8,9].

Through inter-university collaboration research, we aimed to discover novel peptaibols with cell membrane-permeable properties from soil fungi. As a result of this effort, BHM16-2 strain from the BHM collection was identified to produce peptaibols, as confirmed by our in-house HPLC analysis and dereplication process. Large-scale cultivation of this fungus was carried out, and 12 new peptidic molecules were purified using multiple rounds of column chromatography and preparative HPLC purification. The isolated compounds were subsequently evaluated for their cytotoxic activities against HCT-8 (colon cancer) and SK-OV-3 (ovarian cancer) cell lines. Herein, we report the isolation, structure elucidation, and bioactivity of these 12 novel peptaibols.

## 2. Results and Discussion

In an effort to discover novel cytotoxic molecules, the Natural Products Drug Discovery laboratory at Duksung Women’s University has established a fungal library comprising approximately 2000 fungal strains. Crude extracts of these strains have been subjected to in-house HPLC analysis for preliminary dereplication [10,11]. Among these, the BHM collection (Bukhansan Mountain collection) comprises approximately 200 soil fungi strains isolated from 33 different locations in the Bukhansan Mountain area, located in northern Seoul, South Korea. Within this collection, the strain BHM16-2, isolated near Insubong Peak, was identified as a peptaibol-producing fungus. Given the well-known membrane-permeable properties of peptaibols and their associated antimicrobial and cytotoxic activities, we performed further dereplication using HRESIMS data, which revealed that BHM16-2 produces a new class of peptaibols. Based on this dereplication, BHM16-2 was selected for large-scale cultivation, and reproducibility of the novel peptaibol production was confirmed in the large-scale extract by the HRESIMS analysis. A total of 12 metabolites (**1**–**12**) were isolated, and their structures were elucidated. Several of these compounds featured modifications at the C-terminal amino acid residues. Notably, all isolates were determined to be new natural molecules. Herein, we report the structure elucidation of the 12 novel peptidic compounds and the evaluation of their cytotoxic activities against cancer cells.

**Figure 1:**
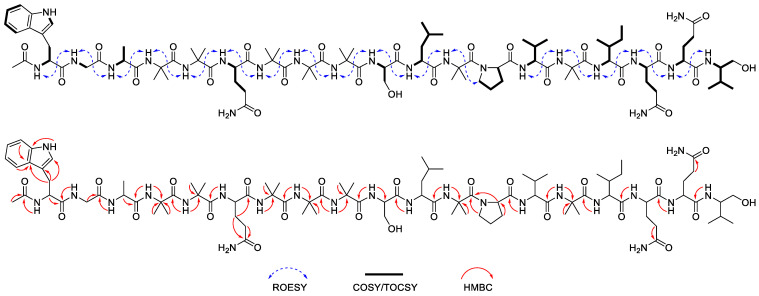
Key ROESY, COSY/TOCSY, and HMBC correlations of compound **1**.

Compound **1** (Structure shown in Table 1) was isolated as a white amorphous powder, and its molecular formula was determined as C_91_H_149_N_23_O_24_ by the HRESIMS data (*m*/*z* 975.0645 [M + 2H]^2+^; calcd 975.0646). The ^1^H and ^13^C-NMR spectrum of **1** exhibited characteristic features of a peptaibol, including 24 exchangeable amide proton signals in the range of δ_H_ 7.28–9.93, α-proton signals in the range of δ_H_ 4.06–5.02, and an acetyl CH_3_ at δ_H_ 2.07, and 23 methyl group proton signals within δ_H_ 0.85–1.83 (Table 2 and Table 3, Figures S2 and S3) [12]. The primary amino acid sequence of **1** was elucidated based on the ROESY correlations between sequential amide protons. In particular, the connectivity among Aib^12^, Pro^13^, and Val^14^ was supported by the ROESY correlations between the NH proton of Aib^12^ (δ_H_ 8.53) and the δ-protons of Pro^13^ (δ_H_ 3.99 and 4.07), as well as between a proton of Pro^13^ (δ_H_ 4.57) and the NH proton of Val^14^ (δ_H_ 8.04). The spin systems of individual amino acid residues were further confirmed by TOCSY experiments (Figure 1). The presence of the non-proteinogenic amino acids Aib and Valol was validated through analysis of COSY and HMBC spectra. The COSY correlations among geminal methylene protons (δ_H_ 4.04 and 4.08), methine protons (δ_H_ 2.38 and 4.27), and methyl protons (δ_H_ 1.19 and 1.04) confirmed the presence of a Valol residue at the C-terminal end. In addition, the HMBC correlations from the α-methyl protons of Aib to the corresponding α-carbons, along with the correlations from neighbouring NH protons to the carbonyl carbon of Aib, further substantiated the assignment and position of each Aib residue (Figure 1). The presence of an N-terminal acetyl group was established based on the HMBC correlations among the acetyl methyl proton (δ_H_ 2.07), the NH proton (δ_H_ 9.45), and the α-proton of Trp^1^ (δ_H_ 5.02) with the acetyl carbonyl carbon (δ_C_ 172.5). The absolute configurations of the amino acid residues were determined by modified Marfey’s analysis [13] which identified Trp, Ala, Gln, Ser, Leu, Pro, Val, Ile, and Valol as possessing the L-configuration (Figure S13). The negative Cotton effect around 205 nm was observed on the electronic circular dichroism (ECD) spectrum, suggesting that compound **1** adopts a right-handed helical conformation (Figure S11) [14]. Based on these results, the complete structure of compound **1** was determined to be N-acetyl-_L_-Trp^1^-Gly^2^-_L_-Ala^3^-Aib^4^-Aib^5^-_L_-Gln^6^-Aib^7^-Aib^8^-Aib^9^-_L_-Ser^10^-_L_-Leu^11^-Aib^12^-_L_-Pro^13^-_L_-Val^14^-Aib^15^-_L_-Ile^16^-_L_-Gln^17^-_L_-Gln^18^-_L_-Valol^19^, and it was designated as bukhansantaibol A (**1**).

Compound **2** was obtained as a white amorphous powder and exhibited the same exact mass as **1** (*m*/*z* 975.0643 [M + 2H]^2+^; calcd 975.0646), consistent with the molecular formula C_91_H_149_N_23_O_24_. The ^1^H and ^13^C-NMR spectra of **2** closely resembled those of **1** (Tables S1 and S2, and Figures S14 and S15). However, detailed analysis of the COSY and HMBC spectra revealed a substitution of the Ile^16^ residue in **1** with a Leu residue in **2** (Table 1). This substitution was supported by the TOCSY correlations corresponding to a leucine spin system, including the NH proton (δ_H_ 7.89), α-methine proton (δ_H_ 4.55), β-methylene protons (δ_H_ 1.91 and 2.01), γ-methine proton (δ_H_ 2.16), and two δ-methyl doublets (δ_H_ 0.96 and 0.86), indicating the presence of Leu at position 16. Furthermore, modified Marfey’s analysis yielded only the _L_-FDLA derivative of Leu, confirming the exclusive incorporation of _L_-Leu at this position (Figure S25). The ECD spectrum also indicated a right-handed helical conformation, consistent with compound **1** (Figure S23). Accordingly, the structure of compound **2** was determined to be N-acetyl-_L_-Trp^1^-Gly^2^-_L_-Ala^3^-Aib^4^-Aib^5^-_L_-Gln^6^-Aib^7^-Aib^8^-Aib^9^-_L_-Ser^10^-_L_-Leu^11^-Aib^12^-_L_-Pro^13^-_L_-Val^14^-Aib^15^-_L_-Leu^16^-_L_-Gln^17^-_L_-Gln^18^-_L_-Valol^19^, and it was designated as bukhansantaibol B (**2**).

Compound **3** was obtained as a white amorphous powder, and its molecular formula was determined to be C_89_H_148_N_22_O_24_ based on the HRESIMS data (*m*/*z* 955.5593 [M + 2H]^2+^; calcd 955.5591). The ^1^H-NMR spectrum of **3** closely resembled that of **1**, except for distinct aromatic proton signals at δ_H_ 7.34 (2H, m), 7.27 (2H, m), and 7.23 (1H, m), consistent with the presence of a phenyl moiety (Table S3 and Figure S26). The TOCSY correlations from the amide proton (δ_H_ 9.58) to the β-methylene protons (δ_H_ 3.33 and 3.43) confirmed the spin system of a phenylalanine residue. The position of this phenylalanine residue at the N-terminus was supported by HMBC correlations from the N-acetyl methyl proton (δ_H_ 2.07) and the amide proton of phenylalanine (δ_H_ 9.58) to the acetyl carbonyl carbon (δ_C_ 172.2). Furthermore, modified Marfey’s analysis verified the substitution of the N-terminal Trp with Phe and confirmed the L-configuration of all amino acid residues (Figure S37). In addition, the ECD spectrum indicated a right-handed helical conformation (Figure S35). Based on these findings, the structure of compound **3** was determined to be N-acetyl-_L_-Phe^1^-Gly^2^-_L_-Ala^3^-Aib^4^-Aib^5^-_L_-Gln^6^-Aib^7^-Aib^8^-Aib^9^-_L_-Ser^10^-_L_-Leu^11^-Aib^12^-_L_-Pro^13^-_L_-Val^14^-Aib^15^-_L_-Ile^16^-_L_-Gln^17^-_L_-Gln^18^-_L_-Valol^19^, and it was designated as bukhansantaibol C (**3**).

Compound **4** was isolated as a white amorphous powder, and the HRESIMS analysis established its molecular formula as C_91_H_149_N_23_O_23_ (*m*/*z* 967.0672 [M + 2H]^2+^; calcd 967.0671), indicating a deficiency of one oxygen atom compared to **1** (Table 1). Comparison of the 1D and 2D NMR spectra with those of **1** revealed the substitution of Ser^10^ with Ala^10^ in compound **4** (Tables S5 and S6, and Figures S38–S44). Furthermore, modified Marfey’s analysis detected only alanine, not serine, supporting the presence of two alanine residues in **4** (Figure S49). The ECD spectrum also supported a right-handed helical conformation, consistent with the structural characteristics observed in related peptaibols (Figure S47). Thus, the structure of **4** was determined as N-acetyl-_L_-Trp^1^-Gly^2^-_L_-Ala^3^-Aib^4^-Aib^5^-_L_-Gln^6^-Aib^7^-Aib^8^-Aib^9^-_L_-Ala^10^-_L_-Leu^11^-Aib^12^-_L_-Pro^13^-_L_-Val^14^-Aib^15^-_L_-Ile^16^-_L_-Gln^17^-_L_-Gln^18^-_L_-Valol^19^ and named as bukhansantaibol D (**4**).

**Figure 2:**
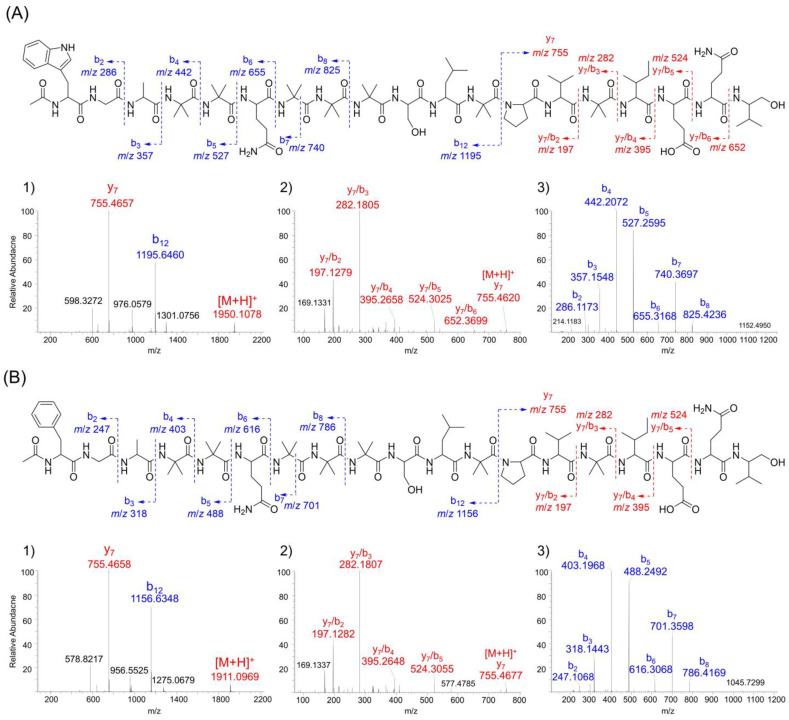
MS/MS analysis of compounds **5** (**A**) and **6** (**B**). (**1**) Full MS^1^ spectrum. (**2**) MS/MS spectrum of y_7_ ion. (**3**) MS/MS spectrum of b_12_ ion.

Compound **5** showed the molecular formula of C_91_H_148_N_22_O_25_ by the HRESIMS (*m*/*z* 975.5563 [M + 2H]^2+^; calcd 975.5566). The ^1^H and ^13^C-NMR spectrum of **5** closely resembled those of **1** (Tables S7 and S8), with a molecular weight difference of 0.984 Da. A detailed analysis of the molecular formula revealed that **5** contains one additional oxygen atom and one fewer nitrogen and hydrogen atom compared to **1**, indicating a substitution of one glutamine (Gln) residue to glutamic acid (Glu). The position of Glu substitution was determined by the MS/MS fragmentation (Figure 2). 

In the full MS^1^ spectrum of **5**, in-source fragmentation produced b_12_ (*m*/*z* 1195.6460) and y_7_ (*m*/*z* 755.4657) ion (Figure 2(A1)). Notably, the y_7_ ion of **5** was 0.984 Da higher than the corresponding y_7_ ion in **1** (*m*/*z* 754.4819), supporting the replacement of Gln with Glu. Further MS/MS analysis of the y_7_ ion revealed a Glu-containing y_7_/b_5_ ion at *m*/*z* 524.3025 (Figure 2(A2)), further validating the site of substitution. In addition, comprehensive NMR analysis, including COSY, TOCSY, and ROESY spectrum confirmed the amino acid sequence. With modified Marfey’s analysis (Figure S61) and ECD spectral analysis (Figure S59), the structure of compound **5** and was determined to be N-acetyl-_L_-Trp^1^-Gly^2^-_L_-A We appreciate your feedback. We have revised the figure labels accordingly.la^3^-Aib^4^-Aib^5^-_L_-Gln^6^-Aib^7^-Aib^8^-Aib^9^-_L_-Ser^10^-_L_-Leu^11^-Aib^12^-_L_-Pro^13^-_L_-Val^14^-Aib^15^-_L_-Ile^16^-_L_-Glu^17^-_L_-Gln^18^-_L_-Valol^19^ and named as bukhansantaibol E (**5**).

Compound **6** exhibited an *m*/*z* value of 956.0508 in the HRESIMS data, corresponding to the molecular formula of C_89_ H_147_N_21_O_25_ ([M + 2H]^2+^, calcd 956.0511). The ^1^H-NMR spectrum of **6** was similar to that of **5**, except for differences observed in the aromatic proton region. In place of the characteristic indole signals of tryptophan, aromatic signals corresponding to a phenyl group (δ_H_ 7.38, 7.28, and 7.22) were observed, suggesting substitution of tryptophan with phenylalanine. (Table S9 and Figure S62). Moreover, a mass difference of 0.984 Da between compounds **3** and **6** indicated Glu substitution, mirroring the relationship observed between compounds **1** and **5**. The MS/MS fragmentation analysis further confirmed the substitution of Glu at position 17 in **6** (Figure 2B). Comprehensive analysis of the COSY, TOCSY, and ROESY correlations established its amino acid connectivity. Furthermore, the HMBC correlations between the acetyl methyl protons (δ_H_ 2.09), the amide proton of phenylalanine (δ_H_ 9.60), and the acetyl carbonyl carbon (δ_C_ 172.3) confirm the presence of an N-terminal acetyl group attached to the phenylalanine residue. The absolute configurations of all amino acid residues, as well as the right-handed helical conformation of **6**, were determined by modified Marfey’s analysis and the ECD spectral data, respectively (Figures S71 and S73). Based on these results, the structure of **6** was elucidated as N-acetyl-_L_-Phe^1^-Gly^2^-_L_-Ala^3^-Aib^4^-Aib^5^-_L_-Gln^6^-Aib^7^-Aib^8^-Aib^9^-_L_-Ser^10^-_L_-Leu^11^-Aib^12^-_L_-Pro^13^-_L_-Val^14^-Aib^15^-_L_-Ile^16^-_L_-Glu^17^-_L_-Gln^18^-_L_-Valol^18^ and named as bukhansantaibol F (**6**).

**Figure 3:**
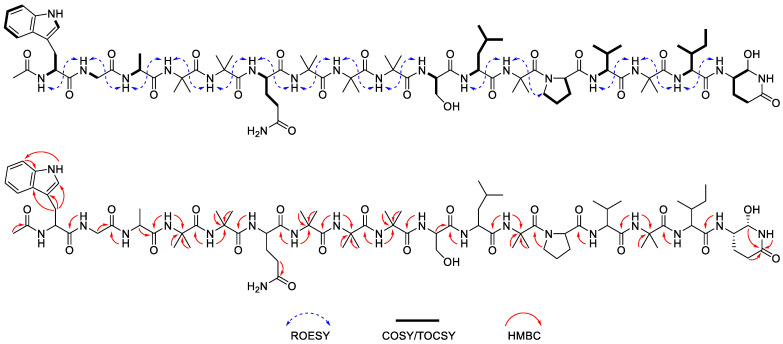
Key ROESY, COSY/TOCSY, and HMBC correlations of compound **7**.

Compound **7** (Structure shown in Table 4) was determined to have a molecular formula of C_81_H_130_N_20_O_21_ based on the HRESIMS data (*m*/*z* 851.4886 [M + 2H – H_2_O]^2+^; calcd 851.4880). Comparison of the ^1^H and ^13^C-NMR spectra of **1** and **7** revealed the absence of characteristic signals corresponding to Valol residue in **7** (Table 5 and Table 6, and Figures S74 and S75). Furthermore, COSY correlation among δ_H_ 5.67 (CH)/4.75 (CH)/2.25 and 2.52 (CH_2_)/2.52 and 3.04 (CH_2_), along with HMBC correlation from δ_H_ 5.67 to amide carbonyl carbon (δ_C_ 171.0) suggested that the C-terminal Gln residue underwent intramolecular cyclization (Figure 3 and Figures S74–S80). 

**Figure 4:**
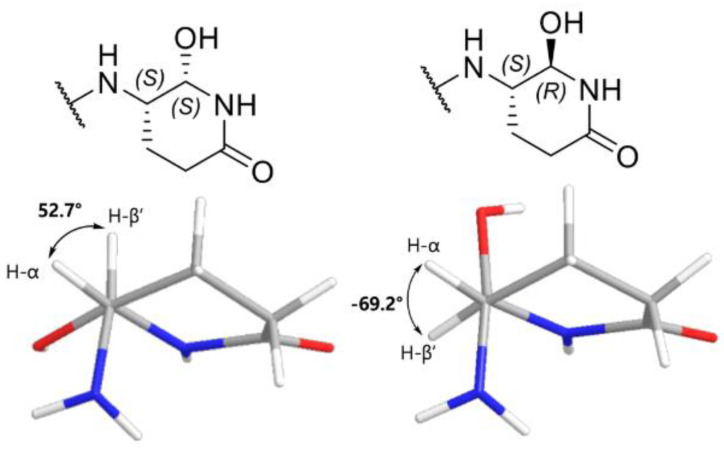
MM2-minimized model of **7** for (*S*,*R*) and (*S*,*S*) configuration.

To the best of our knowledge, this is the first report of C-terminal cyclo-Glnol moiety (2-hydroxy-3-amino-6-piperidone) among peptaibol derivatives. Similar cyclization of C-terminal Asn or Gln residues has been observed during protein splicing in *Pyrococcus abyssi*, resulting in the formation of C-terminal aminosuccinimide or aminoglutarimide, respectively [15,16]. It could be proposed that this occurs via a nucleophilic attack by the γ-amide nitrogen on the adjacent carbonyl carbon of the peptide bond. A subsequent reduction step may then lead to the formation of the unique cyclic structure observed at the C-terminus of compound **7**. The absolute configurations of the amino acid residues and the right-handed helical conformation were confirmed by modified Marfey’s analysis and ECD spectrum, respectively (Figures S83 and S85). Based on the L-configuration of Gln^17^, the configuration of hydroxy group was determined through analysis of the coupling constant. In the MM2-minimized models, the two possible configurations, (*S*,*S*) and (*S*,*R*) exhibited the dihedral angles of 52.7° and −69.2°, respectively, between the α-proton and β’-proton (Figure 4). Given that the measured *J* value of the triplet methine proton at δ_H_ 5.67 was 3.6 Hz, the smaller dihedral angle associated with (*S*,*S*) configuration is more consistent with the observed data, according to the Karplus equation [17]. Based on these results, the structure of **7** was established as N-acetyl-_L_-Trp^1^-Gly^2^-_L_-Ala^3^-Aib^4^-Aib^5^-_L_-Gln^6^-Aib^7^-Aib^8^-Aib^9^-_L_-Ser^10^-_L_-Leu^11^-Aib^12^-_L_-Pro^13^-_L_-Val^14^-Aib^15^-_L_-Ile^16^-_L_-cycGlnol^17^ and named as bukhansantaibol G (**7**).

Compound **8** showed the identical molecular formular with **7**, as determined by the HRESIMS (*m*/*z* 851.4886 [M + 2H – H_2_O]^2+^; calcd 851.4880). The ^1^H and ^13^C-NMR spectra were highly similar to those of **7**, and analysis of the 2D NMR spectra confirmed that **7** and **8** shared the same planar structure. However, subtle differences in the chemical shifts of the C-terminal cyclo-Glnol residue suggested that **8** is a diastereomer of **7**; δ_H_ 9.14 in **8** vs. 8.80 in **7**; δ_C_ 75.8/δ_H_ 5.45 in **8** vs. δ_C_ 78.9/δ_H_ 5.67 in **7**; δ_C_ 21.7/δ_H_ 1.96, 2.73 in **8** vs. δ_C_ 22.7/δ_H_ 2.25, 2.52 in **7**; δ_C_ 30.9/δ_H_ 2.53, 2.59 in **8** vs. δ_C_ 28.9/δ_H_ 2.52, 3.04 in **7**. Notably, the coupling constant 2.5 Hz between the α-proton and β’-proton in **8** was smaller than that observed in **7** (3.6 Hz). Based on the dihedral angle derived from the previously mentioned MM2-minimized model (Figure 4) and interpretation of the Karplus equation, this difference supported the assignment of an (*S*,*R*)-configuration for **8**. The absolute configurations of amino acid residues were confirmed by modified Marfey’s analysis (Figure S97). In addition, ECD spectrum revealed the right-handed helical conformation (Figure S95). Accordingly, the compound was named as bukhansantaibol H (**8**).

Compounds **9** and **10** were assigned the molecular formulas C_82_H_132_N_20_O_21_ and C_80_H_131_N_19_O_21_, respectively, based on the HRESIMS (Table 4). The ^1^H-NMR, COSY, and TOCSY spectra of **9** and **10** revealed similar cyclic-Glnol moiety at the C-terminal with **7** (Tables S13–S16). Additionally, a methoxy singlet (δ_H_ 3.29, 3H, s) was observed in **9** and **10** (Figures S89 and S100). Compound **9** exhibited indole proton signals characteristic of tryptophan (δ_H_ 11.96, 7.41, 7.83, 7.18, 7.28, 7.55; δ_C_ 124.2, 110.2, 128.1, 118.7, 119.1, 121.8, 111.9, 137.4), while compound **10** showed aromatic proton signals consistent with a phenyl ring (δ_H_ 7.30, 7.26, 7.27; δ_C_ 137.5, 129.3, 128.6, 126.9), indicating substitution of tryptophan with phenylalanine. Combined with the result of modified Marfey’s analysis (Figures S109 and S121), the structure of compounds **9** and **10** elucidated as N-acetyl-_L_-Trp^1^-Gly^2^-_L_-Ala^3^-Aib^4^-Aib^5^-_L_-Gln^6^-Aib^7^-Aib^8^-Aib^9^-_L_-Ser^10^-_L_-Leu^11^-Aib^12^-_L_-Pro^13^-_L_-Val^14^-Aib^15^-_L_-Ile^16^-_L_-cycGlnol^17^-Me (**9**) and N-acetyl-_L_-Phe^1^-Gly^2^-_L_-Ala^3^-Aib^4^-Aib^5^-_L_-Gln^6^-Aib^7^-Aib^8^-Aib^9^-_L_-Ser^10^-_L_-Leu^11^-Aib^12^-_L_-Pro^13^-_L_-Val^14^-Aib^15^-_L_-Ile^16^-_L_-cycGlnol^17^-Me (**10**). In addition, compounds **9** and **10** found to have a right-handed helical conformation based on ECD data (Figures S107 and S119). These compounds are assigned to trivial name as bukhansantaibol I (**9**) and bukhansantaibol J (**10**).

**Figure 5:**
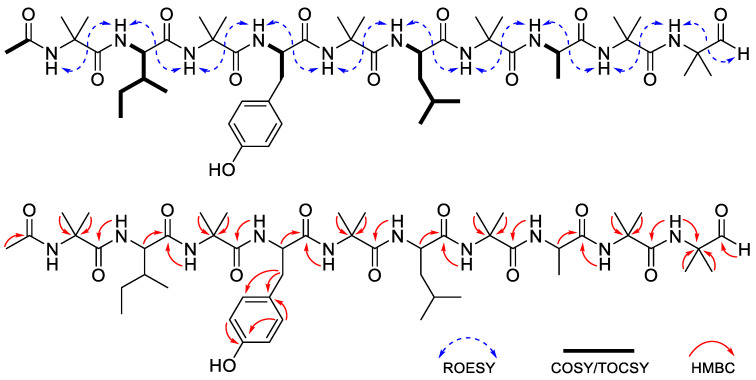
Key ROESY, COSY/TOCSY, and HMBC correlations of compound **11**.

Compounds **11** and **12** (Structure shown in Table 7) were both assigned the molecular formular of C_50_H_82_N_10_O_12_ based on HRESIMS (*m*/*z* 1015.6174 [M + H]^+^; calcd 1015.6186). The ^1^H-NMR spectrum of **11** displayed characteristic signals including one aldehyde proton (δ_H_ 9.90), ten amide protons (δ_H_ 8.04–9.56), two aromatic protons (δ_H_ 7.48 and 7.17), four α-protons (δ_H_ 4.21–4.57), and eighteen methyl protons (δ_H_ 0.78–2.06) (Table 8). The TOCSY correlation enabled the identification of spin systems from amide proton to side chain protons for most amino acid residues, except Aib. Sequential connectivity of the amino acids was further elucidated through the ROESY correlations between amide protons (Figure 5). The HMBC correlation between an amide proton (δ_H_ 9.56), two singlet methyl groups (δ_H_ 1.63, 1.73) and the corresponding α-carbon (δ_C_ 56.7), together with the correlations between the same amide proton (δ_H_ 9.56), the acetyl methyl proton (δ_H_ 2.06), and the acetyl carbonyl carbons (δ_C_ 171.7) confirmed the presence of N-terminal acetylated Aib residue. Additional Aib residues were similarly assigned by the HMBC data. Furthermore, HMBC correlations from the aldehyde proton (δ_H_ 9.90) and two singlet CH_3_ (δ_H_ 1.59 and 1.66) to α-carbon (δ_C_ 59.2) supported the presence of aldehyde moiety of C-terminal Aib residue (Aibal, 2-amino-2-methylpropanal). Based on the etymology of peptaibol, we propose the term ‘peptaibal’ as a new class name for this sequence, which terminates with an aldehyde group instead of an alcohol at the C-terminus. Compound **12** exhibited similar 1D and 2D-NMR spectra with **11**, with the primary difference being the substitution of the Ile^6^ in **11** with Leu^6^ in **12**. ECD spectral analysis revealed that both compounds have a right-handed helical conformation (Figures S131 and S143). Along with the result of modified Marfey’s analysis (Figures S133 and S145), the structures of **11** and **12** were determined as N-acetyl-Aib^1^-_L_-Ile^2^-Aib^3^-_L_-Tyr^4^-Aib^5^-_L_-Leu^6^-Aib^7^-_L_-Ala^8^-Aib^9^-Aibal^10^ and N-acetyl-Aib^1^-_L_-Ile^2^-Aib^3^-_L_-Tyr^4^-Aib^5^-_L_-Ile^6^-Aib^7^-_L_-Ala^8^-Aib^9^-Aibal^10^, respectively, and named as bukhansantaibal A (**11**) and bukhansantaibal B (**12**).

These peptaibals **11** and **12** are presumed to represent intermediates formed during the biosynthetic conversion of a carboxylic acid to an alcohol in peptaibol biosynthesis. The assembly of peptaibol is mediated by NRPS multi-modular enzyme, which iteratively condenses amino acid onto a peptide carrier protein module (PCP)-tethered amino acyl intermediate. Upon completion of chain elongation, the thioesterase domain (TE) typically catalyzes the release of the mature peptide [2]. A reductase domain (thioreductase, TR), located adjacent to the PCP or TE domains, enables the release of the peptide as either an aldehyde or an alcohol [18,19]. Alcohol formation involves a two-step reduction process catalyzed by the TR domain with NADPH as a cofactor, necessitating the reintroduction of an aldehyde intermediate for the second reduction. In certain NRPS systems, structural variations allow only the initial two-electron reduction step to occur, resulting in the predominant formation of aldehyde products [20,21]. While most peptaibol are reduced to alcohol, the isolation of peptaibols bearing C-terminal aldehydes is particularly noteworthy. Further biochemical characterization of the associated enzymatic machinery may elucidate the mechanism underlying aldehyde formation in these systems.

All compounds were evaluated for their inhibitory effects against HCT-8 (colon cancer) and SK-OV-3 (ovarian cancer) cell lines (Table 9). Among them, compounds **1**–**5** exhibited notable cytotoxicity, with IC_50_ values ranging from 2.1 to 19.6 μM. In particular, compound **1**, a 19-residue peptaibol, exhibited potent activity against both HCT-8 and SK-OV-3 cells, with IC_50_ values of 2.1 and 3.4 μM, respectively. Compound **5**, which differ from compound **1** by substitution of one Gln at position 17, showed comparable cytotoxicity, with IC_50_ values of 2.6 and 3.5 μM against HCT-8 and SK-OV-3 cells, respectively. In contrast, substitution of the C-terminal Valol with a cyclic Glnol moiety, as seen in compounds **7**–**10**, resulted in a marked loss of cytotoxicity. The importance of the C-terminal Valol group was further supported by the inactivity of compounds **11** and **12**, which bear a C-terminal aldehyde instead of amino alcohol.

In conclusion, twelve previously undescribed peptidic compounds were isolated from the culture extract of *Trichoderma atroviride*, obtained from soil samples through LC-MS and bioactivity-guided purification. Notably, compounds **7**–**10** and **11**–**12** were characterized by the presence of a unique C-terminal cyclic Glnol moiety and aldehyde group, respectively. Given their structural diversity and potent cytotoxicity against cancer cell lines, peptaibols from *Trichoderma* species, including the strain examined in this study, represent a promising source for the discovery of novel anticancer drug leads.

## 3. Materials and Methods

### 3.1. General Experimental Procedures

Optical rotation was detected using a JASCO DIP-1000 polarimeter. The optical densities (O.D) based on the UV spectra were measured using a JASCO UV-550 spectrophotometer (JASCO, Tokyo, Japan). Electronic circular dichroism (ECD) spectra were recorded on a ChirascanVX spectrometer (Applied Photophysics Ltd., Leatherhead, UK). Nuclear magnetic resonance (NMR) spectra were recorded on Bruker AVANCE 600, 800, and 900 MHz spectrometers (Bruker, Billerica, MA, USA). LC-MS/MS analysis was performed using a UHPLC-HR-Orbitrap mass spectrometer (Thermo Fisher Scientific, Waltham, MA, USA) system equipped with a diode array detector and an YMC-Triart C_18_ column (2.0 μm, 100 × 2.1 mm, i.d., flow rate 0.3 mL/min, YMC Co., Kyoto, Japan). Column chromatography was performed using silica gel (230–400 mesh, Merck, Darmstadt, Germany) and HP20ss gel (Mitsubishi Chemical, Tokyo, Japan). The preparative HPLC was performed using Waters HPLC system equipped with pumps, a 996 photodiode-array detector (Waters Corporation, Milford, MA, USA), and a Luna 5 μm C_18_ column (100 Å, 250 × 21.2 mm I.D., Phenomenex, Torrance, CA, USA) with a flow rate of 10 mL/min. The semi-preparative HPLC were conducted on the same Waters HPLC system using a Luna 5 μm C_18_ (100 Å, 250 × 10 mm I.D., Phenomenex, Torrance, CA, USA) and a Kinetex 5 μm F5 (100 Å, 250 × 10 mm I.D., Phenomenex, Torrance, CA, USA) with a flow rate of 4 mL/min. All solvents used were of ACS grade or better.

### 3.2. Fungal Material

The *Trichoderma atroviride* isolate (soil collection from the territory of Mountain Bukhansan; Deposit No.: BHM16-2) was obtained from a soil sample collected near Insubong Peak in the area of Bukhansan Mountain, South Korea (37.6597281, 126.9845630) in February 2023. The fungi were identified based on the ribosomal internal transcribed spacer (ITS) region (Macrogen, Korea). The resulting sequence data were compared to fungal sequences contained in GenBank, which revealed 100% identity matches to isolates described as *Trichoderma atroviride* (GenBank accession no. KJ786757.1). The sequence data were deposited in GenBank (*Trichoderma atroviride*: GenBank accession no. PV809826).

### 3.3. Fermentation

To carry out large-scale cultivation for the isolation and purification of the bioactive constituents, the fungi were recovered from cryogenic storage (stored in a vial at −80 °C as mycelium with 20% aqueous glycerol). After recovery on Rose Bengal medium plates (15 g of agar, 10 g of malt extract, 1 g of yeast, 0.05 g of chloramphenicol, 0.025 g Rose Bengal, 1 L of DI H_2_O), fungal mycelia were aseptically cut into small pieces (~1 cm^2^) to prepare the inoculum for large-scale fermentation. The fermentation was performed in 1 L flask containing bilayered Cheerios breakfast cereal as the cultivation medium, supplemented with a 0.3% sucrose solution and 0.005% chloramphenicol. Three mycelial fragments were aseptically inoculated onto the surface of Cheerios cereal solid medium in each of ten 1L flasks. The cultures were incubated at room temperature for 3 weeks.

### 3.4. Extraction and Isolation of Compounds ***1**–**12***

Cultured fungi were extracted with EtOAc (0.5 L × 3) for 12 h at room temperature. The extract was filtered and evaporated under reduced pressure to obtain the EtOAc soluble residue, fraction A (3 g). Fraction A was subjected to silica gel vacuum liquid column chromatography (VLC), eluted sequentially with dichloromethane (100%, fraction B), dichloromethane-MeOH (9:1, fraction C), and MeOH (100%, fraction D). Fraction C (1.5 g) was separated using HP20ss gel VLC, eluted sequentially with aqueous MeOH (30% MeOH for fraction E, 50% MeOH for fraction F, 70% MeOH for fraction G, 90% MeOH for fraction H, and 100% MeOH for fraction I), and dichloromethane (CH_2_Cl_2_)-MeOH (1:1, fraction J). Fraction D (1.0 g) was also subjected to the same procedure using HP20ss gel VLC, yielding fractions K (30% MeOH), L (50% MeOH), M (70% MeOH), N (90% MeOH), O (100% MeOH), and P (CH_2_Cl_2_/MeOH 1:1).

Fraction H (244 mg) was fractionated by semi-preparative HPLC (C_18_, isocratic 60% MeOH in H_2_O, flow rate of 4 mL/min) to obtain 13 subfractions (H-1~H-13). Compound **11** (2.3 mg, *t*_R_ = 12.4 min) was purified from subfraction H-9 (4.4 mg) by semi-preparative HPLC using C_18_ (isocratic 65% ACN in H_2_O, flow rate of 4 mL/min). Compound **12** (3.1 mg, *t*_R_ = 14.0 min) was obtained from subfraction H-13 (8.7 mg) via semi-preparative HPLC (C_18_, isocratic 60% ACN in H_2_O, flow rate of 4 mL/min).

Since fractions N (179 mg) and O (106 mg) shared most of metabolites, as observed in their HPLC chromatograms, they were combined and subsequently subjected to further separation (fraction NO). Fraction NO (285 mg) was subjected to semi-preparative HPLC (C_18_, isocratic 55% MeOH in H_2_O with 0.1% formic acid, flow rate of 4 mL/min) to obtain 13 subfractions (NO-1~NO-13). Subfraction NO-1 (10.2 mg) was further purified by semi-preparative HPLC (F5, isocratic 40% ACN in H_2_O, flow rate of 4 mL/min) to give compounds **10** (1.6 mg, *t*_R_ = 12.9 min) and **9** (2.0 mg, *t*_R_ = 14.8 min). Subfraction NO-2 (4.9 mg) was separated by semi-preparative HPLC (F5, isocratic 35% ACN in H_2_O, flow rate of 4 mL/min), yielding six subfractions (NO-2-1~NO-2-6). Compound **7** (1.2 mg, *t*_R_ = 23.2 min) was purified from subfraction NO-2-4 (1.6 mg) by semi-preparative HPLC (C_18_, isocratic 45% ACN in H_2_O, flow rate of 4 mL/min), and compound **8** (1.3 mg, *t*_R_ = 18.9 min) was purified from subfraction NO-2-5 (1.5 mg) by semi-preparative HPLC (C_18_, isocratic 45% ACN in H_2_O, flow rate of 4 mL/min). Subfraction NO-4 (27.2 mg) was subjected to semi-preparative HPLC (C_18_, isocratic 50% ACN in H_2_O, flow rate of 4 mL/min), giving compound **2** (3.4 mg, *t*_R_ = 18.2 min). Compound **1** (6.0 mg, *t*_R_ = 19.3 min) was purified from subfraction NO-5 (30.3 mg) by semi-preparative HPLC (F5, isocratic 37% ACN in H_2_O, flow rate of 4 mL/min). Subfraction NO-6 (17.4 mg) was separated using semi-preparative HPLC (F5, isocratic 35% ACN in H_2_O, flow rate of 4 mL/min) to yield compounds **3** (5.0 mg, *t*_R_ = 61.4 min) and **4** (1.4 mg, *t*_R_ = 95.2 min). Compound **5** (28.9 mg, *t*_R_ = 23.7 min) was purified from subfraction NO-7 (37.0 mg) by semi-preparative HPLC (C_18_, isocratic 52% ACN in H_2_O with 0.1% formic acid, flow rate of 4 mL/min), and compound **6** (16.0 mg, *t*_R_ = 23.1 min) was purified from subfraction NO-8 (22.1 mg) by semi-preparative HPLC (C_18_, isocratic 52% ACN in H_2_O with 0.1% formic acid, flow rate of 4 mL/min).

Bukhansantaibol A (**1**): white amorphous powder; [*α*]^25^_D_ −6.8 (*c* 0.05, MeOH); UV (*c* 0.05, MeOH) λ_max_ (log ε) 202 (4.67), 219 (4.52), 280 (3.65) nm; ECD (c 0.5, CH_3_CN) λmax (Δε) 208 (−67.9), 221 (−48.1); ^1^H-NMR (600 MHz, pyridine-*d*_5_) and ^13^C-NMR (150 MHz, pyridine-*d*_5_), see Table 2 and Table 3; HRESIMS *m*/*z* 975.0645 [M + 2H]^2+^ (calcd for C_91_H_151_N_23_O_24_, 975.0646).

Bukhansantaibol B (**2**): white amorphous powder; [*α*]^25^_D_ −6.7 (*c* 0.05, MeOH); UV (*c* 0.05, MeOH) λ_max_ (log ε) 201 (4.54), 220 (4.37), 280 (3.48) nm; ECD (c 0.3, CH_3_CN) λmax (Δε) 207 (−32.6), 221 (−22.1); ^1^H-NMR (600 MHz, pyridine-*d*_5_) and ^13^C-NMR (150 MHz, pyridine-*d*_5_), see Tables S1 and S2; HRESIMS *m*/*z* 975.0643 [M + 2H]^2+^ (calcd for C_91_H_151_N_23_O_24_, 975.0646).

Bukhansantaibol C (**3**): white amorphous powder; [*α*]^25^_D_ −7.4 (*c* 0.10, MeOH); UV (*c* 0.10, MeOH) λ_max_ (log ε) 201 (4.46) nm; ECD (c 0.5, CH_3_CN) λmax (Δε) 207 (−39.5), 223 (−27.8); ^1^H-NMR (600 MHz, pyridine-*d*_5_) and ^13^C-NMR (150 MHz, pyridine-*d*_5_), see Tables S3 and S4; HRESIMS *m*/*z* 955.5593 [M + 2H]^2+^ (calcd for C_89_H_150_N_22_O_24_, 955.5591).

Bukhansantaibol D (**4**): white amorphous powder; [*α*]^25^_D_ −7.5 (*c* 0.05, MeOH); UV (*c* 0.05, MeOH) λ_max_ (log ε) 200 (4.46), 219 (4.19), 280 (3.33) nm; ECD (c 0.5, CH_3_CN) λmax (Δε) 208 (−25.5), 224 (−16.8); ^1^H-NMR (800 MHz, pyridine-*d*_5_) and ^13^C-NMR (200 MHz, pyridine-*d*_5_), see Tables S5 and S6; HRESIMS *m*/*z* 967.0672 [M + 2H]^2+^ (calcd for C_91_H_151_N_23_O_23_, 967.0671).

Bukhansantaibol E (**5**): white amorphous powder; [*α*]^25^_D_ −7.5 (*c* 0.10, MeOH); UV (*c* 0.10, MeOH) λ_max_ (log ε) 204 (4.45), 219 (4.33), 281 (3.57) nm; ECD (c 0.6, CH_3_CN) λmax (Δε) 208 (−24.6), 220 (−17.0); ^1^H-NMR (600 MHz, pyridine-*d*_5_) and ^13^C-NMR (150 MHz, pyridine-*d*_5_), see Tables S7 and S8; HRESIMS *m*/*z* 975.5563 [M + 2H]^2+^ (calcd for C_91_H_150_N_22_O_25_, 975.5566).

Bukhansantaibol F (**6**): white amorphous powder; [*α*]^25^_D_ −10.3 (*c* 0.10, MeOH); UV (*c* 0.10, MeOH) λ_max_ (log ε) 201 (4.57), 256 (3.54) nm; ECD (c 0.6, CH_3_CN) λmax (Δε) 208 (−42.7), 222 (−30.9); ^1^H-NMR (600 MHz, pyridine-*d*_5_) and ^13^C-NMR (150 MHz, pyridine-*d*_5_), see Tables S9 and S10; HRESIMS *m*/*z* 956.0508 [M + 2H]^2+^ (calcd for C_89_H_149_N_21_O_25_, 956.0511).

Bukhansantaibol G (**7**): white amorphous powder; [*α*]^25^_D_ −6.7 (*c* 0.12, MeOH); UV (*c* 0.12, MeOH) λ_max_ (log ε) 200 (4.06), 219 (3.81), 280 (2.84) nm; ECD (c 0.3, CH_3_CN) λmax (Δε) (Δε) 207 (−28.2), 224 (−19.0); ^1^H-NMR (900 MHz, pyridine-*d*_5_) and ^13^C-NMR (225 MHz, pyridine-*d*_5_), see Table 5 and Table 6; HRESIMS *m*/*z* 851.4886 [M + 2H − H_2_O]^2+^ (calcd for C_81_H_130_N_20_O_20_, 851.4880).

Bukhansantaibol H (**8**): white amorphous powder; [*α*]^25^_D_ −3.8 (*c* 0.12, MeOH); UV (*c* 0.12, MeOH) λ_max_ (log ε) 201 (4.27), 219 (4.01), 280 (3.18) nm; ECD (c 0.6, CH_3_CN) λmax (Δε) 209 (−22.8), 223 (−17.0); ^1^H-NMR (800 MHz, pyridine-*d*_5_) and ^13^C-NMR (200 MHz, pyridine-*d*_5_), see Tables S11 and S12; HRESIMS *m*/*z* 851.4886 [M + 2H − H_2_O]^2+^ (calcd for C_81_H_130_N_20_O_20_, 851.4880).

Bukhansantaibol I (**9**): white amorphous powder; [*α*]^25^_D_ +2.0 (*c* 0.06, MeOH); UV (*c* 0.06, MeOH) λ_max_ (log ε) 200 (4.40), 219 (4.11), 280 (3.35) nm; ECD (c 0.6, CH_3_CN) λmax (Δε) 207 (−34.0), 223 (−22.1); ^1^H-NMR (800 MHz, pyridine-*d*_5_) and ^13^C-NMR (200 MHz, pyridine-*d*_5_), see Tables S13 and S14; HRESIMS *m*/*z* 1733.9958 [M + H]^+^ (calcd for C_82_H_133_N_20_O_21_, 1733.9949).

Bukhansantaibol J (**10**): white amorphous powder; [*α*]^25^_D_ +3.0 (*c* 0.06, MeOH); UV (*c* 0.10, MeOH) λ_max_ (log ε) 200 (4.48) nm; ECD (c 0.6, CH_3_CN) λmax (Δε) 207 (−22.6), 226 (−14.3); ^1^H-NMR (900 MHz, pyridine-*d*_5_) and ^13^C-NMR (225 MHz, pyridine-*d*_5_), see Tables S15 and S16; HRESIMS *m*/*z* 1694.9852 [M + H]^+^ (calcd for C_80_H_132_N_19_O_21_, 1694.9840).

Bukhansantaibal A (**11**): white amorphous powder; [*α*]^25^_D_ +4.6 (*c* 0.10, MeOH); UV (*c* 0.10, MeOH) λ_max_ (log ε) 200 (4.21), 224 (3.71), 277 (2.71) nm; ECD (c 0.5, CH_3_CN) λmax (Δε) 210 (−20.4), 221 (−18.1); ^1^H-NMR (600 MHz, pyridine-*d*_5_) and ^13^C-NMR (150 MHz, pyridine-*d*_5_), see Table 8; HRESIMS *m*/*z* 1015.6174 [M + H]^+^ (calcd for C_50_H_83_N_10_O_12_, 1015.6186).

Bukhansantaibal B (**12**): white amorphous powder; [*α*]^25^_D_ +4.4 (*c* 0.10, MeOH); UV (*c* 0.10, MeOH) λ_max_ (log ε) 203 (4.40), 224 (4.01), 278 (3.09) nm; ECD (c 1.0, CH_3_CN) λmax (Δε) 211 (−20.9), 221 (−17.7); ^1^H-NMR (600 MHz, pyridine-*d*_5_) and ^13^C-NMR (150 MHz, pyridine-*d*_5_), see Table S17; HRESIMS *m*/*z* 1015.6174 [M + H]^+^ (calcd for C_50_H_83_N_10_O_12_, 1015.6186).

### 3.5. Absolute Configuration of Amino Acids in ***1**–**12*** Using Marfey’s Method

Compounds **1**–**12** (each 0.2 mg) were hydrolyzed at 110 °C for 24 h with 400 μL of 6 N HCl containing 1% 2-mercaptoethanol (2-ME) [22]. After cooling, the hydrolysates were evaporated to dryness under reduced pressure at room temperature. The dried residues were then dissolved in H_2_O (100 μL) and 1 M NaHCO_3_ (30 μL). 100 μL of 1% solution of N-*α*-(2,4-dinitro-5-fluorophenyl)-_D/L_-leucinamide (_D/L_-FDLA, Marfey’s reagent, Sigma, St. Louis, MO, USA) in acetone was added to each vial and incubated at 45 °C for 1 h. The mixture was neutralized with 1 N HCl (30 μL) and diluted with ACN (740 μL) before filtering with a PTFE filter (0.22 μm). LC/MS analysis (YMC-Triart C_18_ column, 2.0 μm, 100 × 2.1 mm, i.d.) of the DLA derivatives was performed using a gradient system consisting of solvent A (H_2_O with 0.1% formic acid) and solvent B (ACN with 0.1% formic acid). The gradient was programmed from 90: 10 to 40: 60 (A: B) over 34 min at a flow rate of 0.3 mL/min. The injection volume was 2 μL, and the column oven temperature was maintained at 40 °C. The retention times (min) of the _L_ and _D_-DLA derivatives obtained from hydrolysis were as follows: 18.43 (_L_-Ser-_L_-DLA), 19.69 (_L_-Glu-_L_-DLA), 21.26 (_L_-Ala-_L_-DLA), 21.31 (_L_-Pro-_L_-DLA), 23.94 (_L_-Val-_L_-DLA), 24.28 (_L_-Valol-_L_-DLA), 25.83 (_L_-Ile-_L_-DLA), 26.03 (_L_-Leu-_L_-DLA), 26.05 (_L_-Trp-_L_-DLA), 26.35 (_L_-Phe-_L_-DLA), 19.02 (_L_-Ser-_D_-DLA), 20.67 (_L_-Glu-_D_-DLA), 23.18 (_L_-Pro-_D_-DLA), 23.89 (_L_-Ala-_D_-DLA), 28.26 (_L_-Val-_D_-DLA), 28.38 (_L_-Trp-_D_-DLA), 28.51 (_L_-Valol-_D_-DLA), 29.72 (_L_-Phe-_D_-DLA), 30.52 (_L_-Ile-_D_-DLA), and 30.72 (_L_-Leu-_D_-DLA). The _L_-DLA or _D_-DLA derivatives of authentic amino acid standards of _L_-Ile and _L_-*allo*-Ile were subjected to LC-MS analysis under the same condition. The retention times of the derivatives were 25.84 (_L_-Ile-_L_-DLA) and 25.71 (_L_-*allo*-Ile-_L_-DLA), respectively (Figure S146).

### 3.6. Cell Culture

HCT-8 (colorectal carcinoma), and SK-OV-3 (ovarian cancer) cell lines were obtained from the American Type Culture Collection (ATCC; Manassas, VA, USA). The cells were cultured in RPMI-1640 medium (Cytiva, Marlborough, MA, USA) supplemented with 10% fetal bovine serum (FBS), 100 U/mL penicillin, and streptomycin, and incubated in a humidified atmosphere with 5% CO_2_ at 37 °C [23].

### 3.7. Cell Viability Assay

Cell viability was determined using the 3-(4,5-dimethylthiazol-2-yl)-2,5-diphenyltetrazolium bromide (MTT) colorimetric assay (Sigma Chemical Co., St. Louis, MO). Cells were seeded in 96-well plates and incubated at 37 °C for 24 h. Subsequently, cells were treated with isolated compounds at concentrations of 2, 20, and 100 μM for an additional 24 h. After treatment, 25 μL of MTT solution (5 mg/mL; Thermo Fisher Scientific, Waltham, MA, USA) was added to each well and incubated for 3 h. The resulting formazan crystals were dissolved in 100 μL of dimethyl sulfoxide, and the absorbance was measured at 550 nm using an ELx808 microplate reader (BioTek Instruments Inc., Winooski, VT, USA) [23].

## 4. Conclusions

Twelve new compounds, bukhansantaibols A–J (**1**–**10**) and bukhansantaibals A–B (**11**–**12**), were isolated from the soil fungus *Trichoderma atroviride* collected from the area of Bukhansan Mountain. Among them, six were identified as typical peptaibols (**1**–**6**), while the remaining six were elucidated highly unusual peptaibol derivatives that have not been previously reported: four compounds (**7**–**10**) featured cyclized alcohol-containing amino acids at the C-terminal, and two metabolites (**11**–**12**) possessed an aldehyde group at the C-terminal, which is considered an intermediate in the biosynthetic pathway of peptaibols. All isolated compounds were evaluated from their cytotoxicity against two human cancer cell, HCT-8 and SK-OV-3. As a result, the typical peptaibols exhibited greater cytotoxicity compared to the new class of molecules. Notably, compounds **1**, **4**, **5** showed IC_50_ values of below 10 μM against both cell lines. These results were thought to be associated with the membrane-permeabilizing properties of peptaibols, and suggest that, in addition to their cytotoxicity against cancer cells, these compounds may serve as promising leads for the development of antimicrobial and antiprotozoal agents.

## Figures and Tables

**Table 1:** Structure of compounds **1**–**6**.

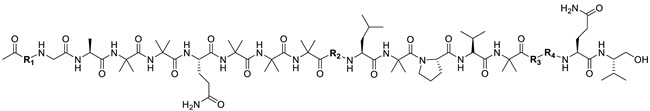					
Compound	Mass	R_1_	R_2_	R_3_	R_4_
**1**	1948.1146	_L_-Trp	_L_-Ser	_L_-Ile	_L_-Gln
**2**	1948.1146	_L_-Trp	_L_-Ser	_L_-Leu	_L_-Gln
**3**	1909.1037	_L_-Phe	_L_-Ser	_L_-Ile	_L_-Gln
**4**	1932.1197	_L_-Trp	_L_-Ala	_L_-Ile	_L_-Gln
**5**	1949.0986	_L_-Trp	_L_-Ser	_L_-Ile	_L_-Glu
**6**	1910.0877	_L_-Phe	_L_-Ser	_L_-Ile	_L_-Glu

**Table 2:** ^1^H (600 MHz) data for compound **1** in pyridine-*d*_5_ (δ in ppm, *J* in Hz).

	Position	δ_H_		Position	δ_H_
Ac	CH_3_	2.07, s	Leu^11^	N-H	8.19, m
Trp^1^	N-H	9.45, d (4.5)		α	4.93, m
	α	5.02, m		β	2.00, m; 2.23, m
	β	3.63, m; 3.70, m		γ	2.16, m
	1	11.94, d (2.4)		δ_1_	0.99, d (6.5)
	2	7.49, d (2.4)		δ_2_	0.98, d (6.5)
	4	7.84, d (8.0)	Aib^12^	N-H	8.53, s
	5	7.19, m		α-Me^1^	1.80, s
	6	7.28, m		α-Me^2^	1.74, s
	7	7.56, m	Pro^13^	α	4.57, m
Gly^2^	N-H	9.93, t (5.6)		β	1.74, m; 2.16, m
	α	4.06, m; 4.17, m		γ	1.75, m; 1.97, m
Ala^3^	N-H	8.48, d (5.4)		δ	3.99, m; 4.07, m
	α	4.49, m	Val^14^	N-H	8.04, m
	β	1.55, d (7.3)		α	4.07, m
Aib^4^	N-H	8.52, s		β	2.57, m
	α-Me^1^	1.80, s		γ_1_	1.20, d (6.5)
	α-Me^2^	1.71, s		γ_2_	1.14, d (6.5)
Aib^5^	N-H	8.27, s	Aib^15^	N-H	7.94, s
	α-Me^1^	1.72, s		α-Me^1^	1.80, s
	α-Me^2^	1.71, s		α-Me^2^	1.71, s
Gln^6^	N-H	8.31, d (4.7)	Ile^16^	N-H	7.73, d (4.9)
	α	4.36, m		α	4.28, m
	β	2.69, m; 2.79, m		β	2.23, m
	γ	2.77, m; 2.89, m		γ_1_	1.40, m
	CONH_2_	7.74, s; 8.14, s		γ_2_	1.07, d (6.7)
Aib^7^	N-H	8.43, s		δ	0.86, t (7.2)
	α-Me^1^	1.75, s	Gln^17^	N-H	8.38, d (5.5)
	α-Me^2^	1.70, s		α	4.69, m
Aib^8^	N-H	8.07, s		β	2.81, m; 2.88, m
	α-Me^1^	1.81, s		γ	2.75, m; 2.97, m
	α-Me^2^	1.74, s		CONH_2_	7.60, s; 8.02, s
Aib^9^	N-H	8.39, s	Gln^18^	N-H	8.18, m
	α-Me^1^	1.82, s		α	5.04, m
	α-Me^2^	1.72, s		β	2.73, m; 3.00, m
Ser^10^	N-H	8.46, d (4.4)		γ	2.81, m; 2.97, m
	α	4.70, m		CONH_2_	7.50, s; 8.10, s
	β	4.37, m; 4.52, m	Valol^19^	N-H	7.76, d (9.4)
				α	4.27, m
				β	2.38, m
				γ_1_	1.19, d (6.8)
				γ_2_	1.04, d (6.8)
				β’	4.04, m; 4.08, m

**Table 3:** ^13^C NMR (150 MHz) data for compound **1** in pyridine-*d*_5_.

	Position	δ_C_		Position	δ_C_		Position	δ_C_
Ac	CH_3_	22.7	Aib^7^	C=O	177.0	Val^14^	C=O	174.0
	C=O	172.5		α	56.7		α	63.4
Trp^1^	C=O	175.0		α-Me^1^	26.9		β	29.4
	α	57.1		α-Me^2^	23.7		γ_1_	19.2
	β	27.9	Aib^8^	C=O	176.4		γ_2_	19.1
	2	124.3		α	56.9	Aib^15^	C=O	177.2
	3	110.3		α-Me^1^	26.9		α	56.7
	3a	128.1		α-Me^2^	23.7		α-Me^1^	27.0
	4	118.7	Aib^9^	C=O	177.7		α-Me^2^	22.7
	5	119.1		α	56.8	Ile^16^	C=O	174.3
	6	121.8		α-Me^1^	27.1		α	61.6
	7	112.0		α-Me^2^	23.0		β	35.9
	7a	137.4	Ser^10^	C=O	171.5		γ_1_	26.5
Gly^2^	C=O	171.9		α	60.4		γ_2_	15.7
	α	44.4		β	62.0		δ	11.1
Ala^3^	C=O	175.2	Leu^11^	C=O	173.6	Gln^17^	C=O	173.6
	α	52.4		α	52.2		α	56.3
	β	16.4		β	40.2		β	27.4
Aib^4^	C=O	175.8		γ	24.7		γ	32.8
	α	56.8		δ_1_	20.8		CONH_2_	174.6
	α-Me^1^	26.4		δ_2_	23.1	Gln^18^	C=O	172.8
	α-Me^2^	22.6	Aib^12^	C=O	175.0		α	55.5
Aib^5^	C=O	177.2		α	56.9		β	28.3
	α	56.5		α-Me^1^	23.3		γ	32.9
	α-Me^1^	26.9		α-Me^2^	27.1		CONH_2_	174.6
	α-Me^2^	22.7	Pro^13^	C=O	175.0	Valol^19^	α	57.3
Gln^6^	C=O	174.8		α	64.0		β	29.2
	α	57.4		β	29.0		γ_1_	19.2
	β	27.2		γ	26.3		γ_2_	19.9
	γ	32.5		δ	49.3		β’	63.0
	CONH_2_	174.5						

**Table 4:** Structure of compounds **7**–**10**.

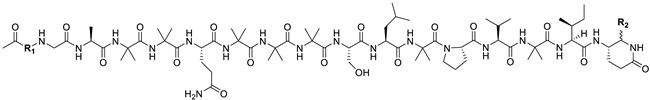			
Compound	Mass	R_1_	R_2_
**7**	1718.9719	_L_-Trp	OH (*S*)
**8**	1718.9719	_L_-Trp	OH (*R*)
**9**	1732.9876	_L_-Trp	OCH_3_ (*S*)
**10**	1693.9767	_L_-Phe	OCH_3_ (*S*)

**Table 5:** ^1^H (900 MHz) data for compound **7** in pyridine-*d*_5_ (δ in ppm, *J* in Hz).

	Position	δ_H_		Position	δ_H_
Ac	CH_3_	2.02, s	Ser^10^	N-H	8.45, m
Trp^1^	N-H	9.44, d (4.6)		α	4.71, m
	α	5.02, m		β	4.37, m; 4.53, m
	β	3.62, m; 3.68, m	Leu^11^	N-H	8.16, m
	1	11.95, d (2.2)		α	5.01, m
	2	7.41, d (2.2)		β	2.05, m; 2.23, m
	4	7.82, d (8.0)		γ	2.14, m
	5	7.18, m		δ_1_	0.99, d (6.6)
	6	7.28, m		δ_2_	0.98, d (6.6)
	7	7.54, m	Aib^12^	N-H	8.41, s
Gly^2^	N-H	9.99, m		α-Me^1^	1.80, s
	α	4.04, m; 4.14, m		α-Me^2^	1.78, s
Ala^3^	N-H	8.52, m	Pro^13^	α	4.61, m
	α	4.51, m		β	1.75, m; 2.21, m
	β	1.53, d (7.3)		γ	1.68, m; 1.91, m
Aib^4^	N-H	8.52, s		δ	3.96, m; 4.02, m
	α-Me^1^	1.69, s	Val^14^	N-H	8.28, d (6.0)
	α-Me^2^	1.72, s		α	4.22, m
Aib^5^	N-H	8.23, s		β	2.61, m
	α-Me^1^	1.69, s		γ_1_	1.29, m
	α-Me^2^	1.70, s		γ_2_	1.19, m
Gln^6^	N-H	8.31, d (4.6)	Aib^15^	N-H	7.94, s
	α	4.36, m		α-Me^1^	1.83, s
	β	2.68, m; 2.77, m		α-Me^2^	1.82, s
	γ	2.77, m; 2.87, m	Ile^16^	N-H	7.53, d (5.3)
	CONH_2_	7.74, s; 8.14, s		α	4.98, m
Aib^7^	N-H	8.35, s		β	2.57, m
	α-Me^1^	1.82, s		γ_1_	1.61, m; 1.95 m
	α-Me^2^	1.78, s		γ_2_	1.13, d (6.8)
Aib^8^	N-H	8.06, s		δ	0.95, t (7.4)
	α-Me^1^	1.80, s	cycGln^17^	N-H	8.19, d (7.5)
	α-Me^2^	1.72, s		α	4.75, m
Aib^9^	N-H	8.39, s		β	2.25, m; 2.52, m
	α-Me^1^	1.82, s		γ	2.52, m; 3.04, m
	α-Me^2^	1.83, s		β’	5.67, t (3.6)
				CONH	8.80, d (3.6)

**Table 6:** ^13^C NMR (225 MHz) data for compound **7** in pyridine-*d*_5_.

	Position	δ_C_		Position	δ_C_		Position	δ_C_
Ac	CH_3_	22.7	Gln^6^	C=O	176.2	Pro^13^	C=O	174.7
	C=O	172.4		α	57.4		α	63.9
Trp^1^	C=O	174.2		β	27.0		β	29.0
	α	57.0		γ	32.6		γ	26.3
	β	27.9		CONH_2_	174.5		δ	49.4
	2	124.0	Aib^7^	C=O	176.9	Val^14^	C=O	173.4
	3	110.3		α	56.9		α	62.7
	3a	128.1		α-Me^1^	26.9		β	29.4
	4	118.7		α-Me^2^	23.2		γ_1_	19.8
	5	119.1	Aib^8^	C=O	176.2		γ_2_	19.3
	6	121.8		α	56.7	Aib^15^	C=O	175.3
	7	111.9		α-Me^1^	26.8		α	57.2
	7a	137.4		α-Me^2^	23.3		α-Me^1^	26.9
Gly^2^	C=O	174.8	Aib^9^	C=O	177.6		α-Me^2^	23.1
	α	44.3		α	57.2	Ile^16^	C=O	172.2
Ala^3^	C=O	175.1		α-Me^1^	27.9		α	58.9
	α	52.2		α-Me^2^	23.5		β	36.7
	β	16.4	Ser^10^	C=O	171.3		γ_1_	25.3
Aib^4^	C=O	176.7		α	60.3		γ_2_	16.3
	α	56.5		β	62.0		δ	11.9
	α-Me^1^	26.8	Leu^11^	C=O	173.9	cycGln^17^	α	50.4
	α-Me^2^	23.2		α	59.0		β	22.7
Aib^5^	C=O	177.1		β	40.3		γ	28.9
	α	56.6		γ	24.7		δ	171.0
	α-Me^1^	23.2		δ_1_	20.8		β’	78.9
	α-Me^2^	26.3		δ_2_	23.3			
			Aib^12^	C=O	174.5			
				α	56.9			
				α-Me^1^	23.0			
				α-Me^2^	26.9			

**Table 7:** Structure of compounds **11**–**12**.

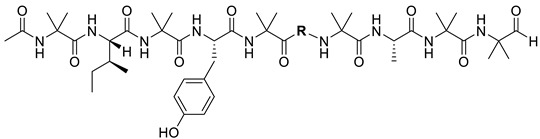					
Compound	Mass	R	Compound	Mass	R
**11**	1014.6114	_L_-Leu	**12**	1014.6114	_L_-Ile

**Table 8:** ^1^H (600 MHz) and ^13^C (150 MHz) data for compound **11** in pyridine-*d*_5_.

	Position	δ_C_	δ_H_		Position	δ_C_	δ_H_
Ac	CH_3_	23.0	2.06, s	Leu^6^	N-H	-	8.13, d (6.4)
	C=O	171.7	-		C=O	174.8	-
Aib^1^	N-H	-	9.56, s		α	55.8	4.49, m
	C=O	177.4	-		β	39.8	1.96, m; 2.11, m
	α	56.7	-		γ	24.8	2.11, m
	α-Me^1^	26.5	1.63, s		δ_1_	21.2	0.93, d (6.2)
	α-Me^2^	24.2	1.73, s		δ_2_	22.7	0.99, d (6.1)
Ile^2^	N-H	-	8.93, d (4.0)	Aib^7^	N-H	-	8.24, s
	C=O	172.7	-		C=O	176.2	-
	α	61.4	4.21, m		α	56.7	-
	β	35.4	1.98, m		α-Me^1^	26.5	1.84, s
	γ_1_	26.8	1.67, m		α-Me^2^	23.4	1.85, s
	γ_2_	15.6	1.02, d (6.8)	Ala^8^	N-H	-	8.18, m
	δ	11.1	0.78, t (7.4)		C=O	173.4	-
Aib^3^	N-H	-	8.29, s		α	52.4	4.52, m
	C=O	177.1	-		α-Me^1^	16.9	1.74, d (2.1)
	α	56.7	-	Aib^9^	N-H	-	8.12, s
	α-Me^1^	26.8	1.72, s		C=O	176.1	-
	α-Me^2^	23.0	1.75, s		α	56.7	-
Tyr^4^	N-H	-	8.04, d (6.0)		α-Me^1^	26.8	1.96, s
	C=O	173.6	-		α-Me^2^	24.4	1.97, s
	α	59.4	4.60, m	Aibal^10^	N-H	-	8.06, s
	β	36.7	3.54, m; 3.53, m		HC=O	201.8	9.90, s
	γ	127.9	-		α	59.2	
	δ	130.5	7.48, d (6.0)		α-Me^1^	21.6	1.66, s
	ε	116.1	7.17, m		α-Me^2^	21.7	1.59, s
	ζ	157.4	-				
Aib^5^	N-H		8.35, s				
	C=O	176.9	-				
	α	56.6	-				
	α-Me^1^	26.6	1.84, s				
	α-Me^2^	23.2	1.78, s				

**Table 9:** IC_50_ values (μM) of compounds **1**–**12** against HCT-8 and SK-OV-3 cancer cells *^a^*.

No.	HCT-8 (Colon Cancer)	SK-OV-3 (Ovarian Cancer)
**1**	2.1 ± 0.2	3.4 ± 0.2
**2**	11.7 ± 0.8	13.8 ± 1.0
**3**	18.5 ± 0.6	19.6 ± 0.1
**4**	7.7 ± 0.3	5.7 ± 0.04
**5**	2.6 ± 1.0	3.5 ± 0.2
**6**	>50	>50
**7**	>50	>50
**8**	>50	>50
**9**	>50	>50
**10**	>50	>50
**11**	>50	>50
**12**	>50	>50
Etoposide *^b^*	33.7 ± 1.5	37.3 ± 1.8

*^a^* Results are presented as mean ± standard deviation from triplicate experiments. *^b^* Etoposide was used as positive control.

## Data Availability

All data are included in the article or Supplementary Materials.

## Supplementary Materials

The following supporting information can be downloaded at: https://www.mdpi.com/article/10.3390/molecules30163422/s1, Figure S1. Isolation scheme for compounds **1**–**12** from BHM16-2-A. Figure S2. ^1^H NMR spectrum of compound **1** (Pyridine-*d*_5_, 600 MHz). Figure S3. ^13^C NMR spectrum of compound **1** (Pyridine-*d*_5_, 150 MHz). Figure S4. COSY spectrum of compound **1**. Figure S5. TOCSY spectrum of compound **1**. Figure S6. HSQC-DEPT spectrum of compound **1**. Figure S7. HMBC spectrum of compound **1**. Figure S8. ROESY spectrum of compound **1**. Figure S9. Key ROESY, COSY/TOCSY, and HMBC correlations of compound **1**. Figure S10. MS fragmentation analysis of compound **1**. Figure S11. Experimental ECD spectrum of compound **1**. Figure S12. HRESIMS spectrum of compound **1**. Figure S13. Advanced Marfey’s analysis of compound **1**. Figure S14. ^1^H NMR spectrum of compound **2** (Pyridine-*d*_5_, 600 MHz). Figure S15. ^13^C NMR spectrum of compound **2** (Pyridine-*d*_5_, 150 MHz). Figure S16. COSY spectrum of compound **2**. Figure S17. TOCSY spectrum of compound **2**. Figure S18. HSQC-DEPT spectrum of compound **2**. Figure S19. HMBC spectrum of compound **2**. Figure S20. ROESY spectrum of compound **2**. Figure S21. Key ROESY, COSY/TOCSY, and HMBC correlations of compound **2**. Figure S22. MS fragmentation analysis of compound **2**. Figure S23. Experimental ECD spectrum of compound **2**. Figure S24. HRESIMS spectrum of compound **2**. Figure S25. Advanced Marfey’s analysis of compound **2**. Figure S26. ^1^H NMR spectrum of compound **3** (Pyridine-*d*_5_, 600 MHz). Figure S27. ^13^C NMR spectrum of compound **3** (Pyridine-*d*_5_, 150 MHz). Figure S28. COSY spectrum of compound **3**. Figure S29. TOCSY spectrum of compound **3**. Figure S30. HSQC-DEPT spectrum of compound **3**. Figure S31. HMBC spectrum of compound **3**. Figure S32. ROESY spectrum of compound **3**. Figure S33. Key ROESY, COSY/TOCSY, and HMBC correlations of compound **3**. Figure S34. MS fragmentation analysis of compound **3**. Figure S35. Experimental ECD spectrum of compound **3**. Figure S36. HRESIMS spectrum of compound **3**. Figure S37. Advanced Marfey’s analysis of compound **3**. Figure S38. ^1^H NMR spectrum of compound **4** (Pyridine-*d*_5_, 800 MHz). Figure S39. ^13^C NMR spectrum of compound **4** (Pyridine-*d*_5_, 200 MHz). Figure S40. COSY spectrum of compound **4**. Figure S41. TOCSY spectrum of compound **4**. Figure S42. HSQC-DEPT spectrum of compound **4**. Figure S43. HMBC spectrum of compound **4**. Figure S44. ROESY spectrum of compound **4**. Figure S45. Key ROESY, COSY/TOCSY, and HMBC correlations of compound **4**. Figure S46. MS fragmentation analysis of compound **4**. Figure S47. Experimental ECD spectrum of compound **4**. Figure S48. HRESIMS spectrum of compound **4**. Figure S49. Advanced Marfey’s analysis of compound **4**. Figure S50. ^1^H NMR spectrum of compound **5** (Pyridine-*d*_5_, 600 MHz). Figure S51. ^13^C NMR spectrum of compound **5** (Pyridine-*d*_5_, 150 MHz). Figure S52. COSY spectrum of compound **5**. Figure S53. TOCSY spectrum of compound **5**. Figure S54. HSQC-DEPT spectrum of compound **5**. Figure S55. HMBC spectrum of compound **5**. Figure S56. ROESY spectrum of compound **5**. Figure S57. Key ROESY, COSY/TOCSY, and HMBC correlations of compound **5**. Figure S58. MS fragmentation analysis of compound **5**. Figure S59. Experimental ECD spectrum of compound **5**. Figure S60. HRESIMS spectrum of compound **5**. Figure S61. Advanced Marfey’s analysis of compound **5**. Figure S62. ^1^H NMR spectrum of compound **6** (Pyridine-*d*_5_, 600 MHz). Figure S63. ^13^C NMR spectrum of compound **6** (Pyridine-*d*_5_, 150 MHz). Figure S64. COSY spectrum of compound **6**. Figure S65. TOCSY spectrum of compound **6**. Figure S66. HSQC-DEPT spectrum of compound **6**. Figure S67. HMBC spectrum of compound **6**. Figure S68. ROESY spectrum of compound **6**. Figure S69. Key ROESY, COSY/TOCSY, and HMBC correlations of compound **6**. Figure S70. MS fragmentation analysis of compound **6**. Figure S71. Experimental ECD spectrum of compound **6**. Figure S72. HRESIMS spectrum of compound **6**. Figure S73. Advanced Marfey’s analysis of compound **6**. Figure S74. ^1^H NMR spectrum of compound **7** (Pyridine-*d*_5_, 900 MHz). Figure S75. ^13^C NMR spectrum of compound **7** (Pyridine-*d*_5_, 225 MHz). Figure S76. COSY spectrum of compound **7**. Figure S77. TOCSY spectrum of compound **7**. Figure S78. HSQC-DEPT spectrum of compound **7**. Figure S79. HMBC spectrum of compound **7**. Figure S80. NOESY spectrum of compound **7**. Figure S81. Key ROESY, COSY/TOCSY, and HMBC correlations of compound **7**. Figure S82. MS fragmentation analysis of compound **7**. Figure S83. Experimental ECD spectrum of compound **7**. Figure S84. HRESIMS spectrum of compound **7**. Figure S85. Advanced Marfey’s analysis of compound **7**. Figure S86. ^1^H-NMR spectrum of compound **8** (Pyridine-*d*_5_, 800 MHz). Figure S87. ^13^C-NMR spectrum of compound **8** (Pyridine-*d*_5_, 200 MHz). Figure S88. COSY spectrum of compound **8**. Figure S89. TOCSY spectrum of compound **8**. Figure S90. HSQC-DEPT spectrum of compound **8**. Figure S91. HMBC spectrum of compound **8**. Figure S92. ROESY spectrum of compound **8**. Figure S93. Key ROESY, COSY/TOCSY, and HMBC correlations of compound **8**. Figure S94. MS fragmentation analysis of compound **8**. Figure S95. Experimental ECD spectrum of compound **8**. Figure S96. HRESIMS spectrum of compound **8**. Figure S97. Advanced Marfey’s analysis of compound **8**. Figure S98. ^1^H-NMR spectrum of compound **9** (Pyridine-*d*_5_, 800 MHz). Figure S99. ^13^C-NMR spectrum of compound **9** (Pyridine-*d*_5_, 200 MHz). Figure S100. COSY spectrum of compound **9**. Figure S101. TOCSY spectrum of compound **9**. Figure S102. HSQC-DEPT spectrum of compound **9**. Figure S103. HMBC spectrum of compound **9**. Figure S104. ROESY spectrum of compound **9**. Figure S105. Key ROESY, COSY/TOCSY, and HMBC correlations of compound **9**. Figure S106. MS fragmentation analysis of compound **9**. Figure S107. Experimental ECD spectrum of compound **9**. Figure S108. HRESIMS spectrum of compound **9**. Figure S109. Advanced Marfey’s analysis of compound **9**. Figure S110. ^1^H-NMR spectrum of compound **10** (Pyridine-*d*_5_, 900 MHz). Figure S111. ^13^C-NMR spectrum of compound **10** (Pyridine-*d*_5_, 225 MHz). Figure S112. COSY spectrum of compound **10**. Figure S113. TOCSY spectrum of compound **10**. Figure S114. HSQC-DEPT spectrum of compound **10**. Figure S115. HMBC spectrum of compound **10**. Figure S116. ROESY spectrum of compound **10**. Figure S117. Key ROESY, COSY/TOCSY, and HMBC correlations of compound **10**. Figure S118. MS fragmentation analysis of compound **10**. Figure S119. Experimental ECD spectrum of compound **10**. Figure S120. HRESIMS spectrum of compound **10**. Figure S121. Advanced Marfey’s analysis of compound **10**. Figure S122. ^1^H-NMR spectrum of compound **11** (Pyridine-*d*_5_, 600 MHz). Figure S123. ^13^C-NMR spectrum of compound **11** (Pyridine-*d*_5_, 150 MHz). Figure S124. COSY spectrum of compound **11**. Figure S125. TOCSY spectrum of compound **11**. Figure S126. HSQC-DEPT spectrum of compound **11**. Figure S127. HMBC spectrum of compound **11**. Figure S128. ROESY spectrum of compound **11**. Figure S129. Key ROESY, COSY/TOCSY, and HMBC correlations of compound **11**. Figure S130. MS fragmentation analysis of compound **11**. Figure S131. Experimental ECD spectrum of compound **11**. Figure S132. HRESIMS spectrum of compound **11**. Figure S133. Advanced Marfey’s analysis of compound **11**. Figure S134. ^1^H-NMR spectrum of compound **12** (Pyridine-*d*_5_, 600 MHz). Figure S135. ^13^C-NMR spectrum of compound **12** (Pyridine-*d*_5_, 150 MHz). Figure S136. COSY spectrum of compound **12**. Figure S137. TOCSY spectrum of compound **12**. Figure S138. HSQC-DEPT spectrum of compound **12**. Figure S139. HMBC spectrum of compound **12**. Figure S140. ROESY spectrum of compound **12**. Figure S141. Key ROESY, COSY/TOCSY, and HMBC correlations of compound **12**. Figure S142. MS fragmentation analysis of compound **12**. Figure S143. Experimental ECD spectrum of compound **12**. Figure S144. HRESIMS spectrum of compound **12**. Figure S145. Advanced Marfey’s analysis of compound **12**. Figure S146. Advanced Marfey’s analysis for L-Ile identification. Table S1. ^1^H (600 MHz) data for compound **2** in pyridine-*d*_5_ (δ in ppm, *J* in Hz). Table S2. ^13^C NMR (150 MHz) data for compound **2** in pyridine-*d*_5_. Table S3. ^1^H (600 MHz) data for compound **3** in pyridine-*d*_5_ (δ in ppm, *J* in Hz). Table S4. ^13^C (150 MHz) data for compound **3** in pyridine-*d*_5_. Table S5. ^1^H (800 MHz) data for compound **4** in pyridine-*d*_5_ (δ in ppm, *J* in Hz). Table S6. ^13^C (200 MHz) data for compound **4** in pyridine-*d*_5_. Table S7. ^1^H (600 MHz) data for compound **5** in pyridine-*d*_5_ (δ in ppm, *J* in Hz). Table S8. ^13^C (150 MHz) data for compound **5** in pyridine-*d*_5_. Table S9. ^1^H (600 MHz) data for compound **6** in pyridine-*d*_5_ (δ in ppm, *J* in Hz). Table S10. ^13^C (150 MHz) data for compound **6** in pyridine-*d*_5_. Table S11. ^1^H (800 MHz) data for compound **8** in pyridine-*d*_5_ (δ in ppm, *J* in Hz). Table S12. ^13^C (200 MHz) data for compound **8** in pyridine-*d*_5_. Table S13. ^1^H (800 MHz) data for compound **9** in pyridine-*d*_5_ (δ in ppm, *J* in Hz). Table S14. ^13^C (200 MHz) data for compound **9** in pyridine-*d*_5_. Table S15. ^1^H (900 MHz) data for compound **10** in pyridine-*d*_5_ (δ in ppm, *J* in Hz). Table S16. ^13^C (225 MHz) data for compound **10** in pyridine-*d*_5_. Table S17. ^1^H (600 MHz) and ^13^C (150 MHz) data for compound **12** in pyridine-*d*_5_.

## References

[1] Neumann N.K.N., Stoppacher N., Zeilinger S., Degenkolb T., Brückner H., Schuhmacher R. (2015). The Peptaibiotics Database—A Comprehensive Online Resource. Chem. Biodivers..

[2] Pereira-Dias L., Oliveira-Pinto P.R., Fernandes J.O., Regalado L., Mendes R., Teixeira C., Mariz-Ponte N., Gomes P., Santos C. (2023). Peptaibiotics: Harnessing the Potential of Microbial Secondary Metabolites for Mitigation of Plant Pathogens. Biotechnol. Adv..

[3] Degenkolb T., Kirschbaum J., Brückner H. (2007). New Sequences, Constituents, and Producers of Peptaibiotics: An Updated Review. Chem. Biodivers..

[4] Kyle K.E., Puckett S.P., Caraballo-Rodríguez A.M., Rivera-Chávez J., Samples R.M., Earp C.E., Raja H.A., Pearce C.J., Ernst M., van der Hooft J.J.J. (2023). *Trachymyrmex septentrionalis* Ants Promote Fungus Garden Hygiene Using *Trichoderma*-derived Metabolite Cues. Proc. Natl. Acad. Sci. USA.

[5] Röhrich C.R., Iversen A., Jaklitsch W.M., Voglmayr H., Berg A., Dörfelt H., Thrane U., Vilcinskas A., Nielsen K.F., von Döhren H. (2012). Hypopulvins, Novel Peptaibiotics from the Polyporicolous Fungus *Hypocrea pulvinata* are Produced During Infection of Its Natural Hosts. Fungal Biol..

[6] Marik T., Tyagi C., Balázs D., Urbán P., Szepesi Á., Bakacsy L., Endre G., Rakk D., Szekeres A., Andersson M.A. (2019). Structural Diversity and Bioactivities of Peptaibol Compounds from the Longibrachiatum Clade of the Filamentous Fungal Genus *Trichoderma*. Front. Microbiol..

[7] Hou X., Sun R., Feng Y., Zhang R., Zhu T., Che Q., Zhang G., Li D. (2022). Peptaibols: Diversity, bioactivity, and biosynthesis. Eng. Microbiol..

[8] Han J.S., Kim E.-S., Cho Y.B., Kim S.Y., Lee M.K., Hwang B.Y., Lee J.W. (2024). Cytotoxic Peptaibols from *Trichoderma guizhouense*, a Fungus Isolated from an Urban Soil Sample. J. Nat. Prod..

[9] Senadeera S.P.D., Wang D., Kim C.-K., Smith E.A., Durrant D.E., Alexander P.A., Wendt K.L., Stephen A.G., Morrison D.K., Cichewicz R.H. (2022). Tolypocladamides A–G: Cytotoxic Peptaibols from *Tolypocladium inflatum*. J. Nat. Prod..

[10] Kim E.-S., Lee J.W. (2025). Cytotoxic Compounds Obtained through Cell Viability Screening of Fungal Extracts, Isolated from Urban Soil Samples I. Nat. Prod. Sci..

[11] Lee D., Lee J.W. (2025). Cytotoxicity against MDA-MB-231 Breast Cancer Cells of Fungal Metabolites of Trichoderma sp. Collected from Medicinal Herbal Garden. Nat. Prod. Sci..

[12] Lee J.W., Collins J.E., Wendt K.L., Chakrabarti D., Cichewicz R.H. (2021). Leveraging Peptaibol Biosynthetic Promiscuity for Next-Generation Antiplasmodial Therapeutics. J. Nat. Prod..

[13] Harada K.-I., Fujii K., Hayashi K., Suzuki M., Ikai Y., Oka H. (1996). Application of _D,L_-FDLA Derivatization to Determination of Absolute Configuration of Constituent Amino Acids in Peptide by Advanced Marfey’s Method. Tetrahedron Lett..

[14] Liu D., Lin H., Proksch P., Tang X., Shao Z., Lin W. (2015). Microbacterins A and B, New Peptaibols from the Deep Sea Actinomycete *Microbacterium sediminis* sp. nov. YLB-01(T). Org. Lett..

[15] Mills K.V., Manning J.S., Garcia A.M., Wuerdeman L.A. (2004). Protein Splicing of a *Pyrococcus abyssi* Intein with a C-terminal Glutamine. J. Biol. Chem..

[16] Amitai G., Dassa B., Pietrokovski S. (2004). Protein Splicing of Inteins with Atypical Glutamine and Aspartate C-terminal Residues. J. Biol. Chem..

[17] Haasnoot C.A.G., de Leeuw F.A.A.M., Altona C. (1980). The Relationship Between Proton-proton NMR Coupling Constants and Substituent Electronegativities-I: An Empirical Generalization of the Karplus Equation. Tetrahedron.

[18] Reimer J.M., Haque A.S., Tarry M.J., Schmeing T.M. (2018). Piecing Together Nonribosomal Peptide Synthesis. Curr. Opin. Struct. Biol..

[19] Mullowney M., McClure R.A., Robey M.T., Kelleher N.L., Thomson R.J. (2018). Natural Products from Thioester Reductase Containing Biosynthetic Pathways. Nat. Prod. Rep..

[20] Gahloth D., Dunstan M.S., Quaglia D., Klumbys E. (2017). Lockhart-Cairns, M.P.; Hill, A.M.; Derrington, S.R.; Scrutton, N.S.; Turner, N.J.; Leys, D. Structures of Carboxylic Acid Reductase Reveal Domain Dynamics Underlying Catalysis. Nat. Chem. Biol..

[21] Chen Y.Q., McClure R.A., Zheng Y.P., Thomson R.J., Kelleher N.L. (2013). Proteomics Guided Discovery of Flavopeptins: Anti-proliferative Aldehydes Synthesized by a Reductase Domain-Containing Non-ribosomal Peptide Synthetase. J. Am. Chem. Soc..

[22] Adebiyi A.P., Jin D.-H., Ogawa T., Muramoto K. (2005). Acid Hydrolysis of Protein in a Microcapillary Tube for the Recovery of Tryptophan. Biosci. Biotechnol. Biochem..

[23] Kim E.-S., Moon A. (2015). Ursolic acid inhibits the invasive phenotype of SNU-484 human gastric cancer cells. Oncol. Lett..

