# Supplementary material for: Cytotoxic Peptidic Metabolites Isolated from the Soil-Derived Fungus Trichoderma atroviride
Source: Molecules. 2025 Aug 19;30(16):3422. doi: 10.3390/molecules30163422 (PMC12388242; doi:10.3390/molecules30163422)
Supplement: Supplementary file 1 [file molecules-30-03422-s001.zip › molecules-3764107-supplementary.pdf]

Supplementary Data

Cytotoxic Peptidic Metabolites Isolated from the Soil-Derived Fungus *Trichoderma atroviride*

Jun Gu Kim [1,†], Jae Sang Han [1,†], Dahyeon Lee [2], Mi Kyeong Lee [1], Bang Yeon Hwang [1],* and Jin Woo Lee [2.3,*]

[1] College of Pharmacy, Chungbuk National University, Cheongju 28160, Republic of Korea

[2] College of Pharmacy, Duksung Women′s University, Seoul 01369, Republic of Korea

[3] Department of AI Drug Discovery, Duksung Women′s University, Seoul 01369, Republic of Korea

[†] These authors contributed equally to this work.

Figure S1. Isolation scheme for compounds **1–12** from BHM16-2-A

Figure S2. ^{1}H NMR spectrum of compound **1** (Pyridine-d_5, 600 MHz)

Figure S3. ^{13}C NMR spectrum of compound **1** (Pyridine-d_5, 150 MHz)

Figure S4. COSY spectrum of compound **1**

Figure S5. TOCSY spectrum of compound **1**

Figure S6. HSQC-DEPT spectrum of compound **1**

Figure S7. HMBC spectrum of compound **1**

Figure S8. ROESY spectrum of compound **1**

Figure S9. Key ROESY, COSY/TOCSY, and HMBC correlations of compound **1**

Figure S10. MS fragmentation analysis of compound **1**

Figure S11. Experimental ECD spectrum of compound **1**

Figure S12. HRESIMS spectrum of compound **1**

Figure S13. Advanced Marfey's analysis of compound **1**

Figure S14. ^{1}H NMR spectrum of compound **2** (Pyridine-d_5, 600 MHz)

Figure S15. ^{13}C NMR spectrum of compound **2** (Pyridine-d_5, 150 MHz)

Figure S16. COSY spectrum of compound **2**

Figure S17. TOCSY spectrum of compound **2**

Figure S18. HSQC-DEPT spectrum of compound **2**

Figure S19. HMBC spectrum of compound **2**

Figure S20. ROESY spectrum of compound **2**

Figure S21. Key ROESY, COSY/TOCSY, and HMBC correlations of compound **2**

Figure S22. MS fragmentation analysis of compound **2**

Figure S23. Experimental ECD spectrum of compound **2**

Figure S24. HRESIMS spectrum of compound **2**

Figure S25. Advanced Marfey's analysis of compound **2**

Figure S26. ^{1}H NMR spectrum of compound **3** (Pyridine-d_5, 600 MHz)

Figure S27. ^{13}C NMR spectrum of compound **3** (Pyridine-d_5, 150 MHz)

Figure S28. COSY spectrum of compound **3**

Figure S29. TOCSY spectrum of compound **3**

Figure S30. HSQC-DEPT spectrum of compound **3**

Figure S31. HMBC spectrum of compound **3**

Figure S32. ROESY spectrum of compound **3**

Figure S33. Key ROESY, COSY/TOCSY, and HMBC correlations of compound **3**

Figure S34. MS fragmentation analysis of compound **3**

Figure S35. Experimental ECD spectrum of compound **3**

Figure S36. HRESIMS spectrum of compound **3**

Figure S37. Advanced Marfey's analysis of compound **3**

Figure S38. ^{1}H NMR spectrum of compound **4** (Pyridine-d_5, 800 MHz)

Figure S39. ^{13}C NMR spectrum of compound **4** (Pyridine-d_5, 200 MHz)

Figure S40. COSY spectrum of compound **4**

Figure S41. TOCSY spectrum of compound **4**

Figure S42. HSQC-DEPT spectrum of compound **4**

Figure S43. HMBC spectrum of compound **4**

Figure S44. ROESY spectrum of compound **4**

Figure S45. Key ROESY, COSY/TOCSY, and HMBC correlations of compound **4**

Figure S46. MS fragmentation analysis of compound **4**

Figure S47. Experimental ECD spectrum of compound **4**

Figure S48. HRESIMS spectrum of compound **4**

Figure S49. Advanced Marfey's analysis of compound **4**

Figure S50. ^{1}H NMR spectrum of compound **5** (Pyridine-d_5, 600 MHz)

Figure S51. ^{13}C NMR spectrum of compound **5** (Pyridine-d_5, 150 MHz)

Figure S52. COSY spectrum of compound **5**

Figure S53. TOCSY spectrum of compound **5**

Figure S54. HSQC-DEPT spectrum of compound **5**

Figure S55. HMBC spectrum of compound **5**

Figure S56. ROESY spectrum of compound **5**

Figure S57. Key ROESY, COSY/TOCSY, and HMBC correlations of compound **5**

Figure S58. MS fragmentation analysis of compound **5**

Figure S59. Experimental ECD spectrum of compound **5**

Figure S60. HRESIMS spectrum of compound **5**

Figure S61. Advanced Marfey's analysis of compound **5**

Figure S62. ^{1}H NMR spectrum of compound **6** (Pyridine-d_5, 600 MHz)

Figure S63. ^{13}C NMR spectrum of compound **6** (Pyridine-d_5, 150 MHz)

Figure S64. COSY spectrum of compound **6**

Figure S65. TOCSY spectrum of compound **6**

Figure S66. HSQC-DEPT spectrum of compound **6**

Figure S67. HMBC spectrum of compound **6**

Figure S68. ROESY spectrum of compound **6**

Figure S69. Key ROESY, COSY/TOCSY, and HMBC correlations of compound **6**

Figure S70. MS fragmentation analysis of compound **6**

Figure S71. Experimental ECD spectrum of compound **6**

Figure S72. HRESIMS spectrum of compound **6**

Figure S73. Advanced Marfey's analysis of compound **6**

Figure S74. ^{1}H NMR spectrum of compound **7** (Pyridine-d_5, 900 MHz)

Figure S75. ^{13}C NMR spectrum of compound **7** (Pyridine-d_5, 225 MHz)

Figure S76. COSY spectrum of compound **7**

Figure S77. TOCSY spectrum of compound **7**

Figure S78. HSQC-DEPT spectrum of compound **7**

Figure S79. HMBC spectrum of compound **7**

Figure S80. NOESY spectrum of compound **7**

Figure S81. Key ROESY, COSY/TOCSY, and HMBC correlations of compound **7**

Figure S82. MS fragmentation analysis of compound **7**

Figure S83. Experimental ECD spectrum of compound **7**

Figure S84. HRESIMS spectrum of compound **7**

Figure S85. Advanced Marfey's analysis of compound **7**

Figure S86. ^{1}H-NMR spectrum of compound **8** (Pyridine-d_5, 800 MHz)

Figure S87. ^{13}C-NMR spectrum of compound **8** (Pyridine-d_5, 200 MHz)

Figure S88. COSY spectrum of compound **8**

Figure S89. TOCSY spectrum of compound **8**

Figure S90. HSQC-DEPT spectrum of compound **8**

Figure S91. HMBC spectrum of compound **8**

Figure S92. ROESY spectrum of compound **8**

Figure S93. Key ROESY, COSY/TOCSY, and HMBC correlations of compound **8**

Figure S94. MS fragmentation analysis of compound **8**

Figure S95. Experimental ECD spectrum of compound **8**

Figure S96. HRESIMS spectrum of compound **8**

Figure S97. Advanced Marfey's analysis of compound **8**

Figure S98. ^{1}H-NMR spectrum of compound **9** (Pyridine-d_5, 800 MHz)

Figure S99. ^{13}C-NMR spectrum of compound **9** (Pyridine-d_5, 200 MHz)

Figure S100. COSY spectrum of compound **9**

Figure S101. TOCSY spectrum of compound **9**

Figure S102. HSQC-DEPT spectrum of compound **9**

Figure S103. HMBC spectrum of compound **9**

Figure S104. ROESY spectrum of compound **9**

Figure S105. Key ROESY, COSY/TOCSY, and HMBC correlations of compound **9**

Figure S106. MS fragmentation analysis of compound **9**

Figure S107. Experimental ECD spectrum of compound **9**

Figure S108. HRESIMS spectrum of compound **9**

Figure S109. Advanced Marfey's analysis of compound **9**

Figure S110. ^{1}H-NMR spectrum of compound **10** (Pyridine-d_5, 900 MHz)

Figure S111. ^{13}C-NMR spectrum of compound **10** (Pyridine-d_5, 225 MHz)

Figure S112. COSY spectrum of compound **10**

Figure S113. TOCSY spectrum of compound **10**

Figure S114. HSQC-DEPT spectrum of compound **10**

Figure S115. HMBC spectrum of compound **10**

Figure S116. ROESY spectrum of compound **10**

Figure S117. Key ROESY, COSY/TOCSY, and HMBC correlations of compound **10**

Figure S118. MS fragmentation analysis of compound **10**

Figure S119. Experimental ECD spectrum of compound **10**

Figure S120. HRESIMS spectrum of compound **10**

Figure S121. Advanced Marfey's analysis of compound **10**

Figure S122. ^{1}H-NMR spectrum of compound **11** (Pyridine-d_5, 600 MHz)

Figure S123. ^{13}C-NMR spectrum of compound **11** (Pyridine-d_5, 150 MHz)

Figure S124. COSY spectrum of compound **11**

Figure S125. TOCSY spectrum of compound **11**

Figure S126. HSQC-DEPT spectrum of compound **11**

Figure S127. HMBC spectrum of compound **11**

Figure S128. ROESY spectrum of compound **11**

Figure S129. Key ROESY, COSY/TOCSY, and HMBC correlations of compound **11**

Figure S130. MS fragmentation analysis of compound **11**

Figure S131. Experimental ECD spectrum of compound **11**

Figure S132. HRESIMS spectrum of compound **11**

Figure S133. Advanced Marfey's analysis of compound **11**

Figure S134. ^{1}H-NMR spectrum of compound **12** (Pyridine-d_5, 600 MHz)

Figure S135. ^{13}C-NMR spectrum of compound **12** (Pyridine-d_5, 150 MHz)

Figure S136. COSY spectrum of compound **12**

Figure S137. TOCSY spectrum of compound **12**

Figure S138. HSQC-DEPT spectrum of compound **12**

Figure S139. HMBC spectrum of compound **12**

Figure S140. ROESY spectrum of compound **12**

Figure S141. Key ROESY, COSY/TOCSY, and HMBC correlations of compound **12**

Figure S142. MS fragmentation analysis of compound **12**

Figure S143. Experimental ECD spectrum of compound **12**

Figure S144. HRESIMS spectrum of compound **12**

Figure S145. Advanced Marfey's analysis of compound **12**

Figure S146. Advanced Marfey's analysis for L-Ile identification

Table S1. ^{1}H (600 MHz) data for compound **2** in pyridine-d_5 (δ in ppm, *J* in Hz)[a]

Table S2. ^{13}C NMR (150 MHz) data for compound **2** in pyridine-d_5

Table S3. ^{1}H (600 MHz) data for compound **3** in pyridine-d_5 (δ in ppm, *J* in Hz)[a]

Table S4. ^{13}C (150 MHz) data for compound **3** in pyridine-d_5

Table S5. ^{1}H (800 MHz) data for compound **4** in pyridine-d_5 (δ in ppm, *J* in Hz)[a]

Table S6. ^{13}C (200 MHz) data for compound **4** in pyridine-d_5.

Table S7. ^{1}H (600 MHz) data for compound **5** in pyridine-d_5 (δ in ppm, *J* in Hz)[a]

Table S8. ^{13}C (150 MHz) data for compound **5** in pyridine-d_5.

Table S9. ^{1}H (600 MHz) data for compound **6** in pyridine-d_5 (δ in ppm, *J* in Hz)[a]

Table S10. ^{13}C (150 MHz) data for compound **6** in pyridine-d_5.

Table S11. ^{1}H (800 MHz) data for compound **8** in pyridine-d_5 (δ in ppm, *J* in Hz)[a]

Table S12. ^{13}C (200 MHz) data for compound **8** in pyridine-d_5.

Table S13. ^{1}H (800 MHz) data for compound **9** in pyridine-d_5 (δ in ppm, *J* in Hz)[a]

Table S14. ^{13}C (200 MHz) data for compound **9** in pyridine-d_5.

Table S15. ^{1}H (900 MHz) data for compound **10** in pyridine-d_5 (δ in ppm, *J* in Hz)[a]

Table S16. ^{13}C (225 MHz) data for compound **10** in pyridine-d_5.

Table S17. ^{1}H (600 MHz) and ^{13}C (150 MHz) data for compound **12** in pyridine-d_5.

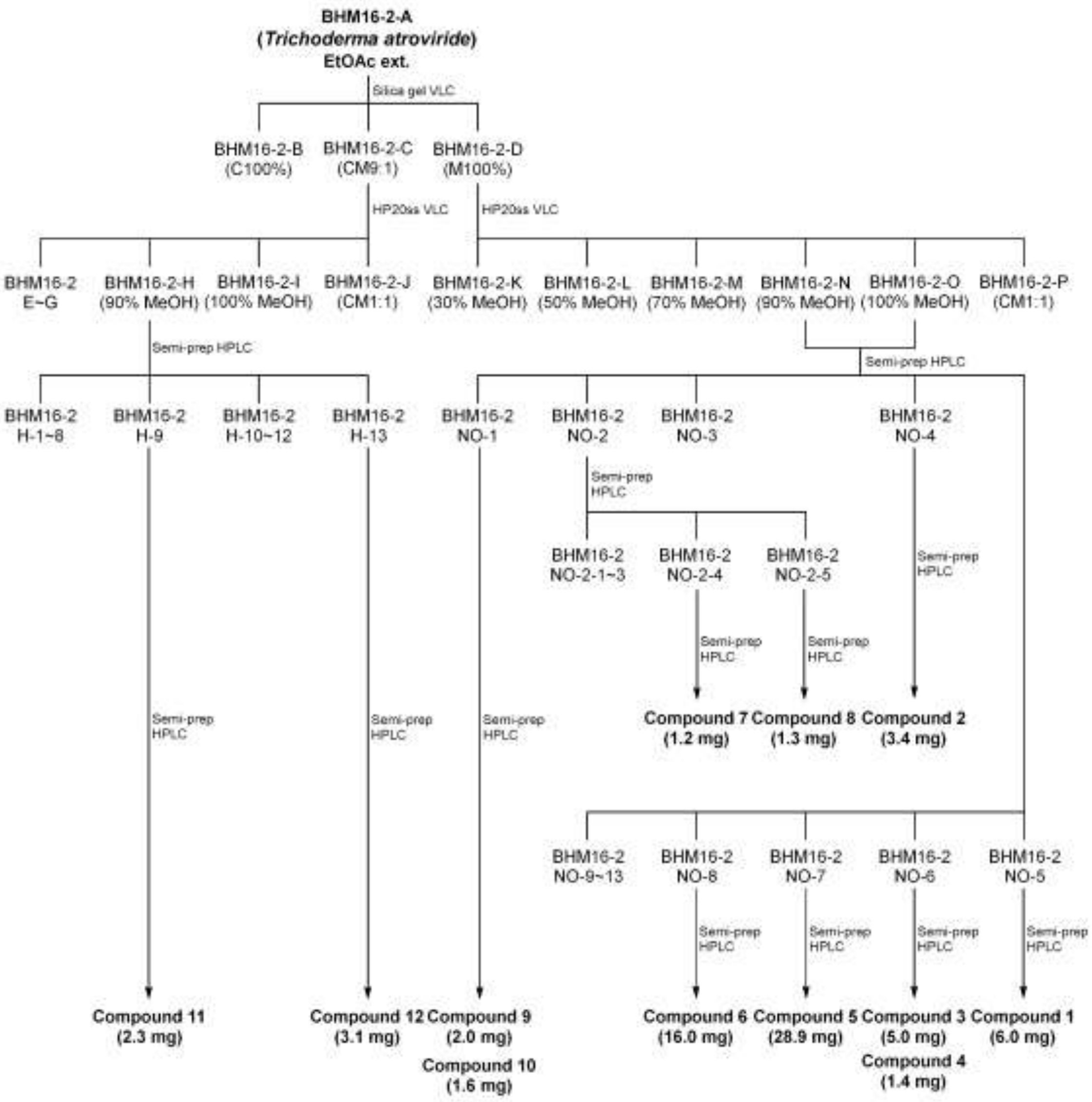

Figure S1. Isolation scheme for compounds **1**–**12** from BHM16-2-A

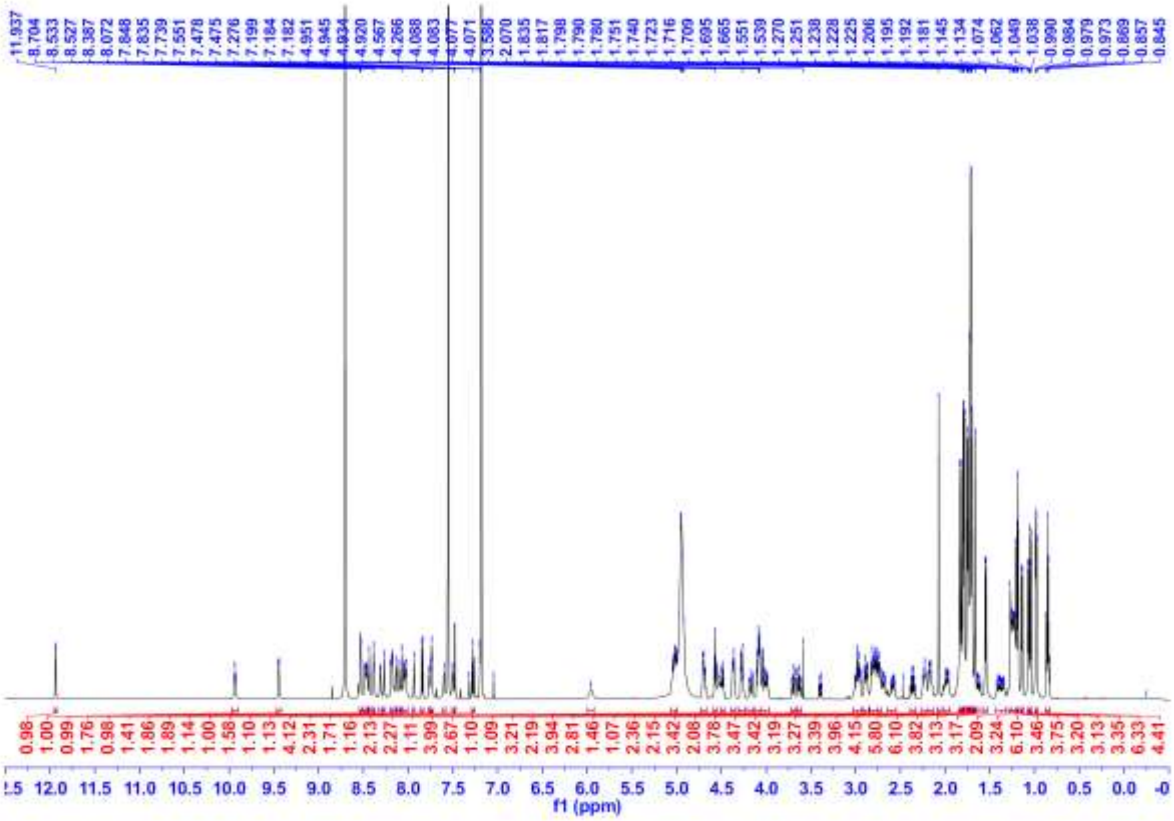

Figure S2. ^{1}H NMR spectrum of compound **1** (Pyridine-d_5, 600 MHz)

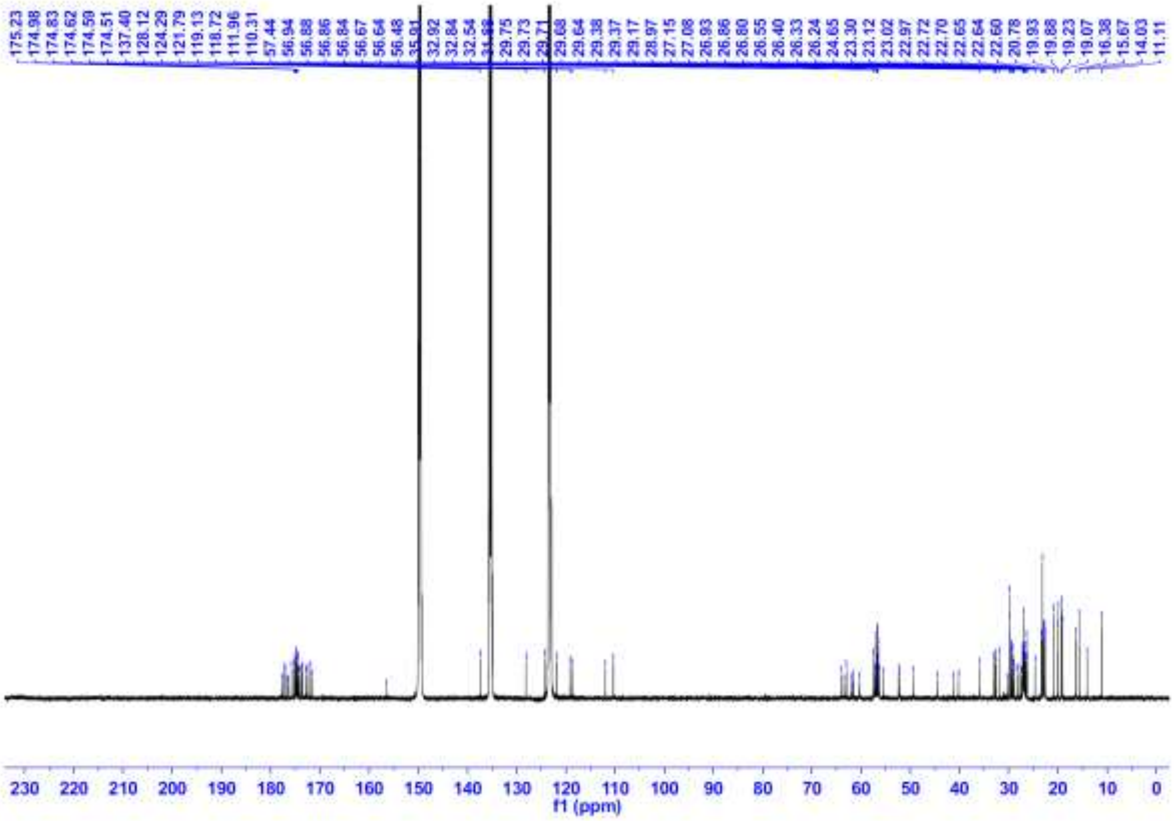

Figure S3. ^{13}C NMR spectrum of compound **1** (Pyridine-d_5, 150 MHz)

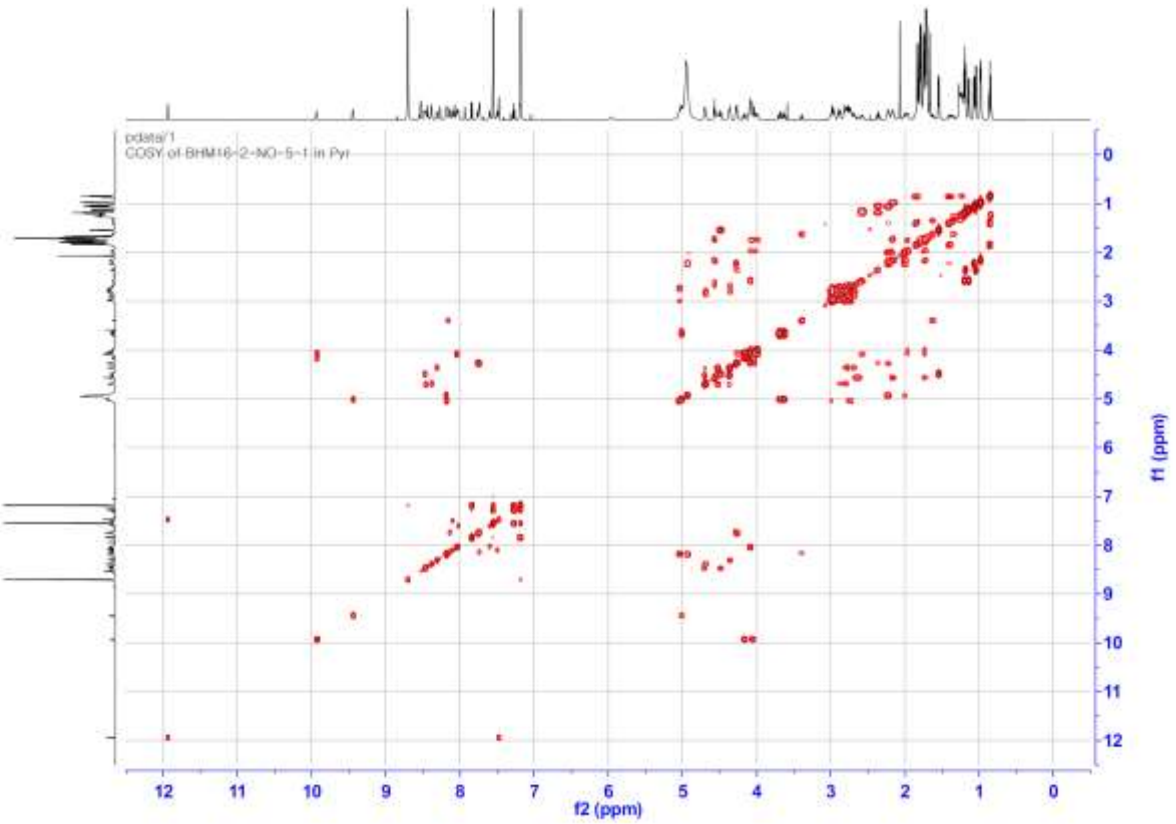

Figure S4. COSY spectrum of compound **1**

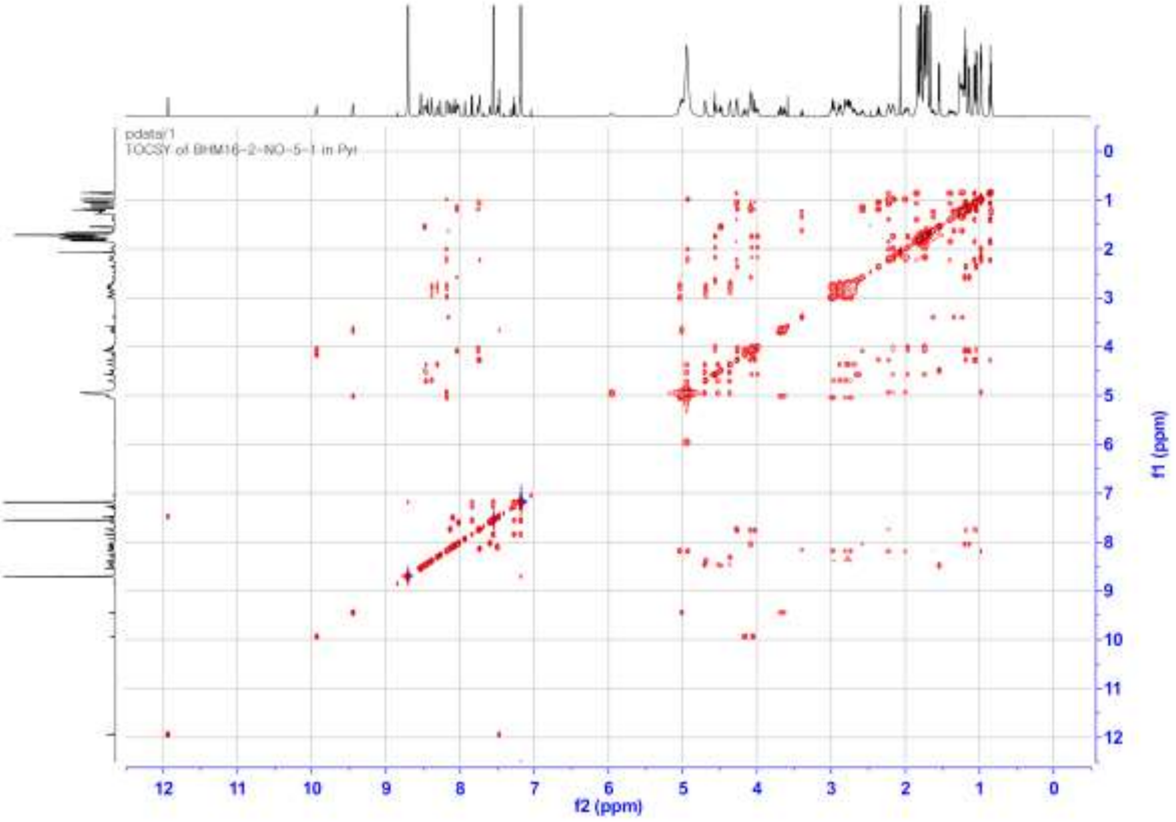

Figure S5. TOCSY spectrum of compound **1**

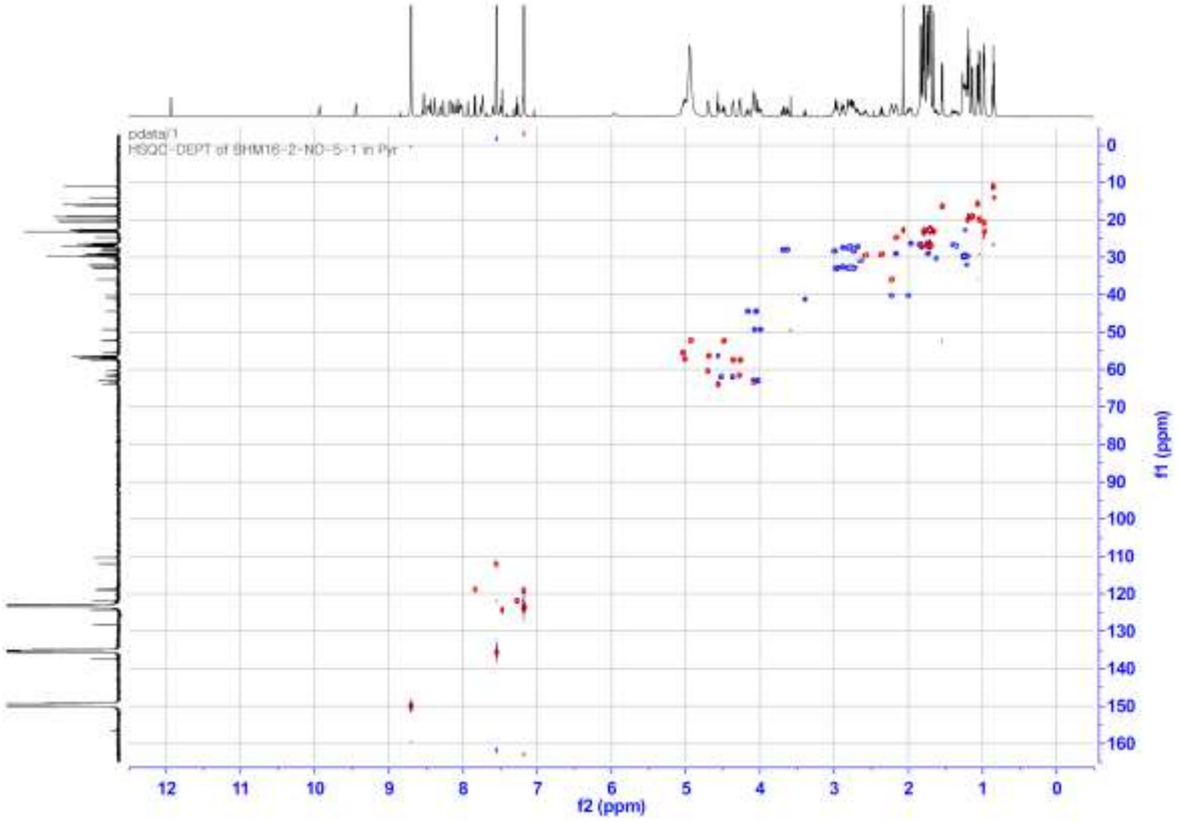

Figure S6. HSQC-DEPT spectrum of compound **1**

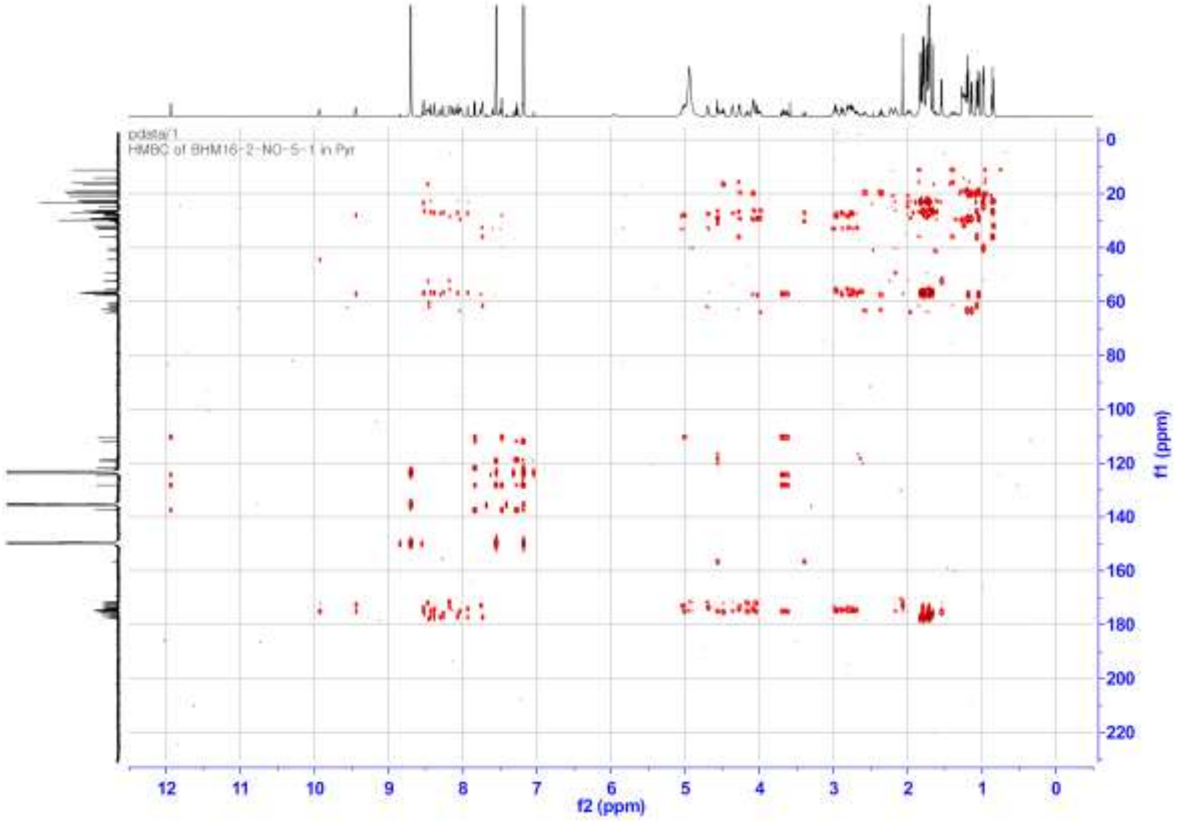

Figure S7. HMBC spectrum of compound **1**

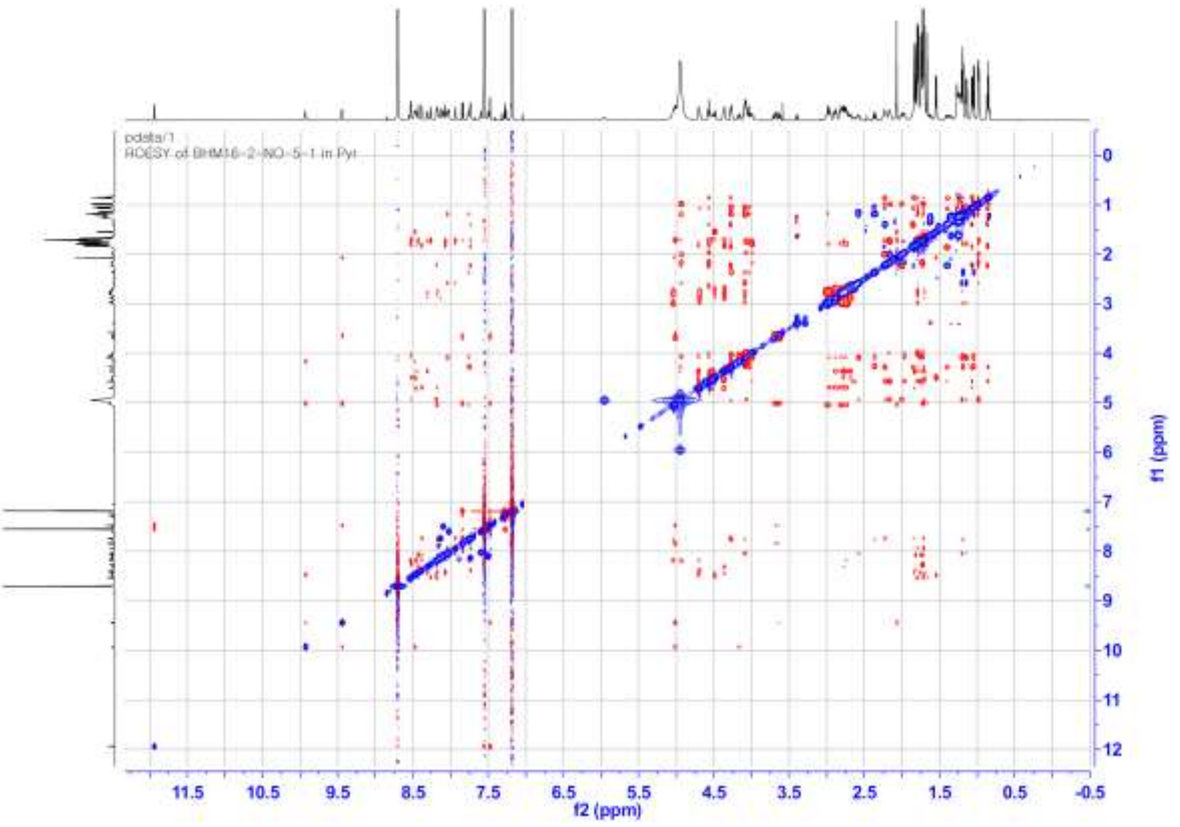

Figure S8. ROESY spectrum of compound **1**

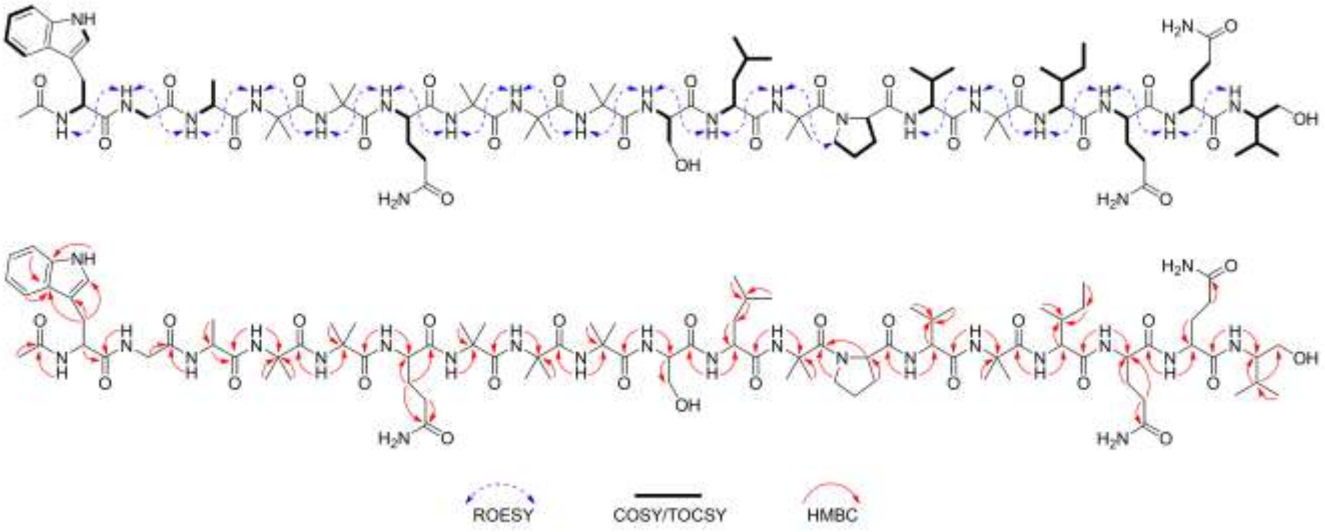

Figure S9. Key ROESY, COSY/TOCSY, and HMBC correlations of compound **1**

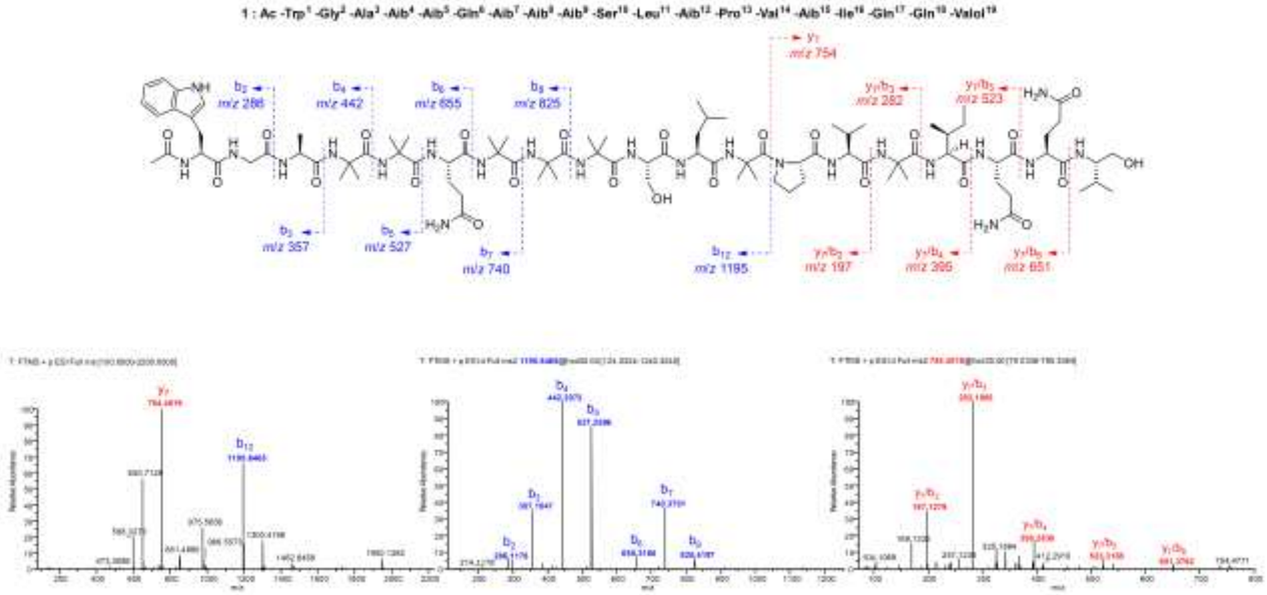

Figure S10. MS fragmentation analysis of compound **1**

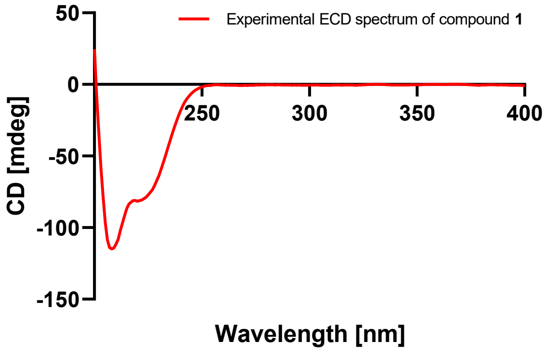

Figure S11. Experimental ECD spectrum of compound **1**

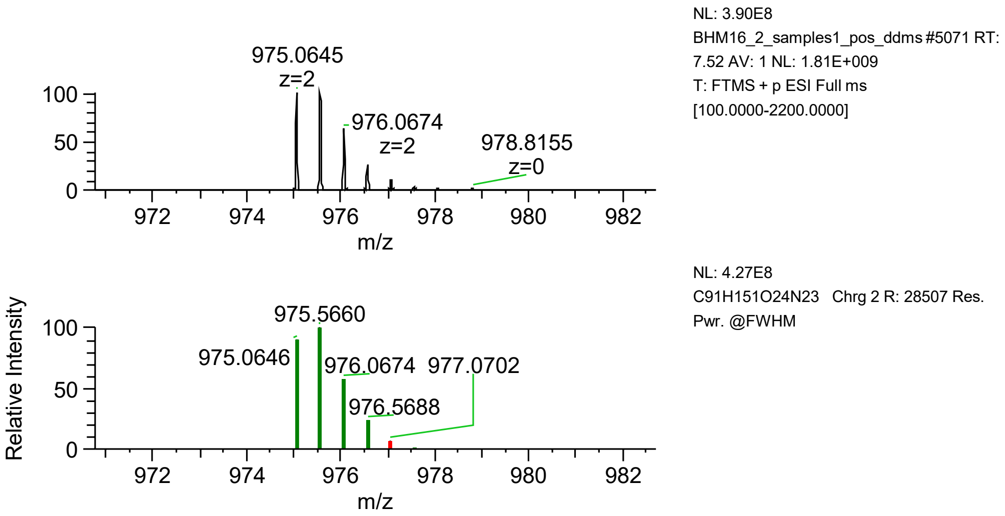

Figure S12. HRESIMS spectrum of compound **1**

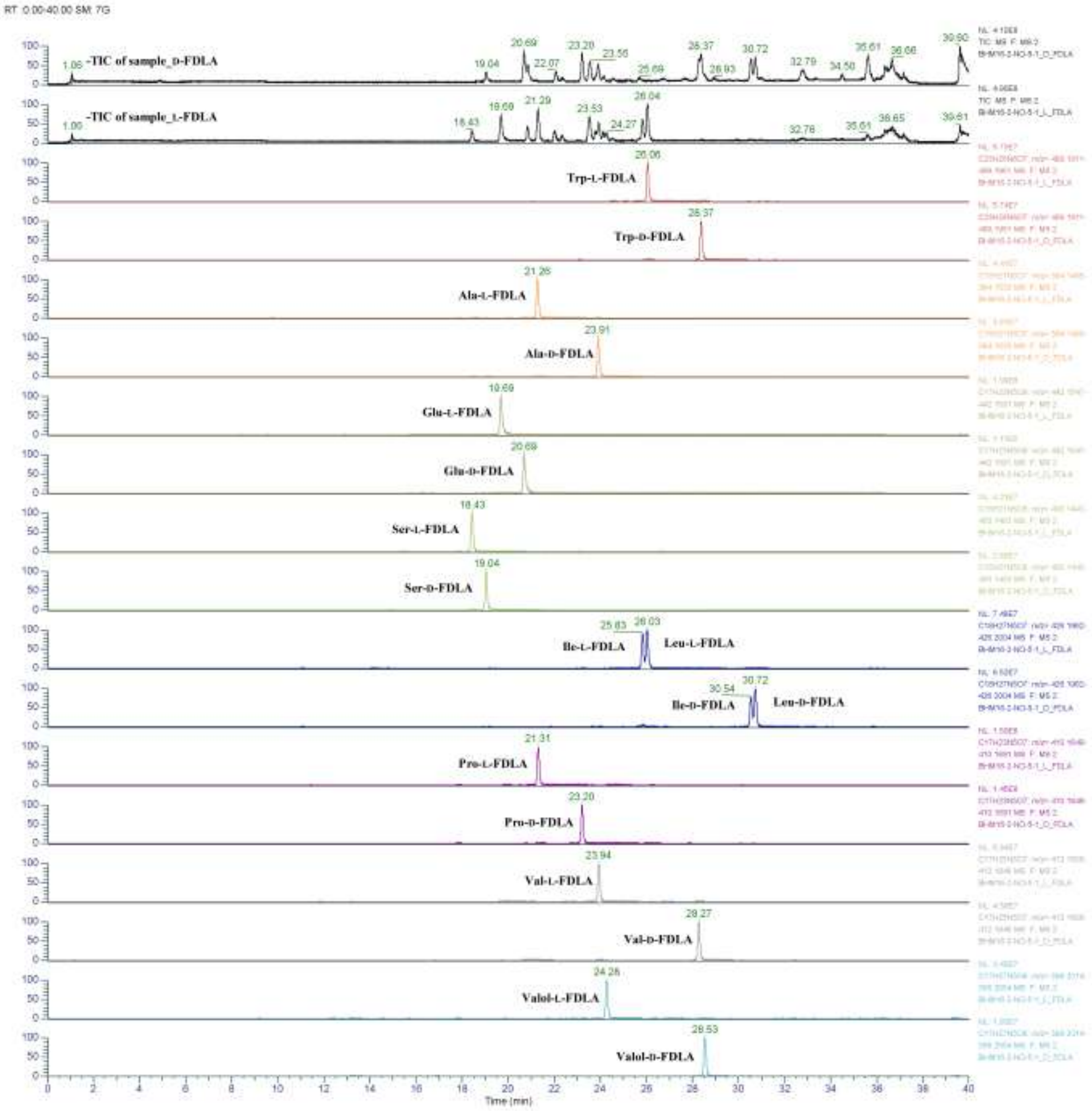

Figure S13. Advanced Marfey's analysis of compound **1**

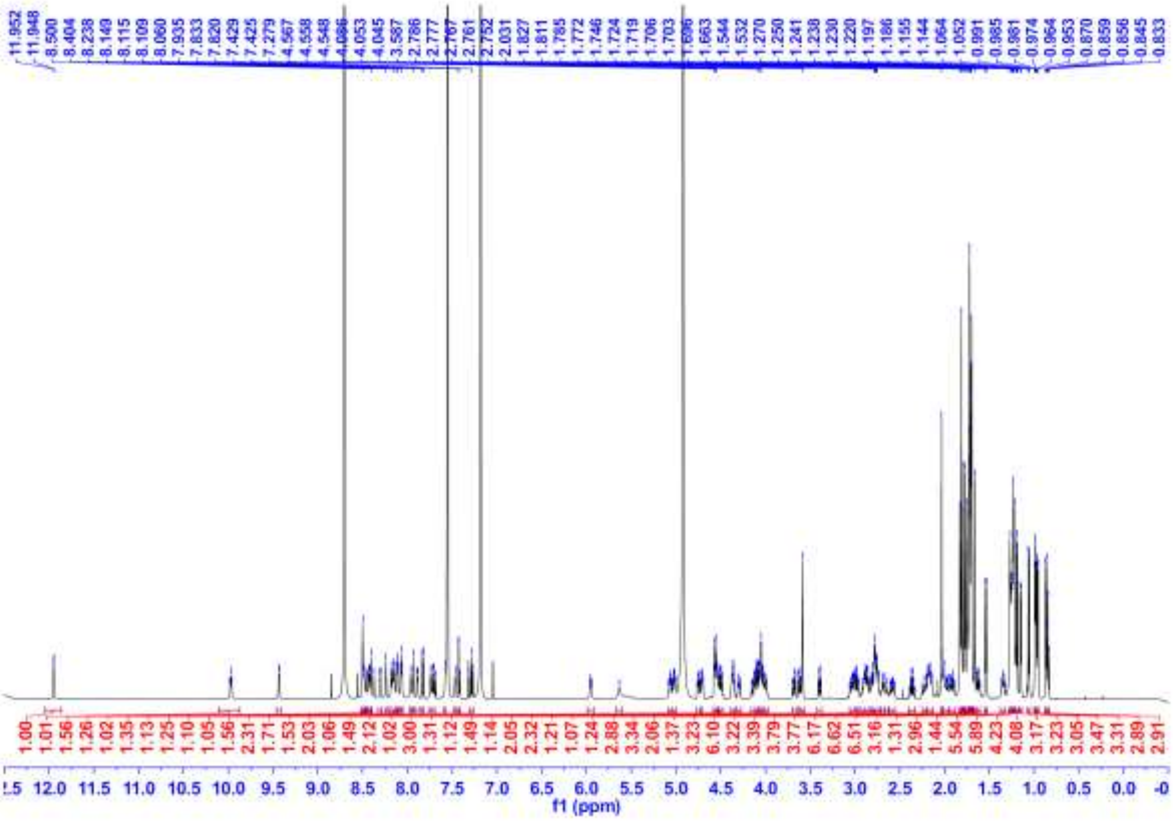

Figure S14. ^{1}H NMR spectrum of compound **2** (Pyridine-d_5, 600 MHz)

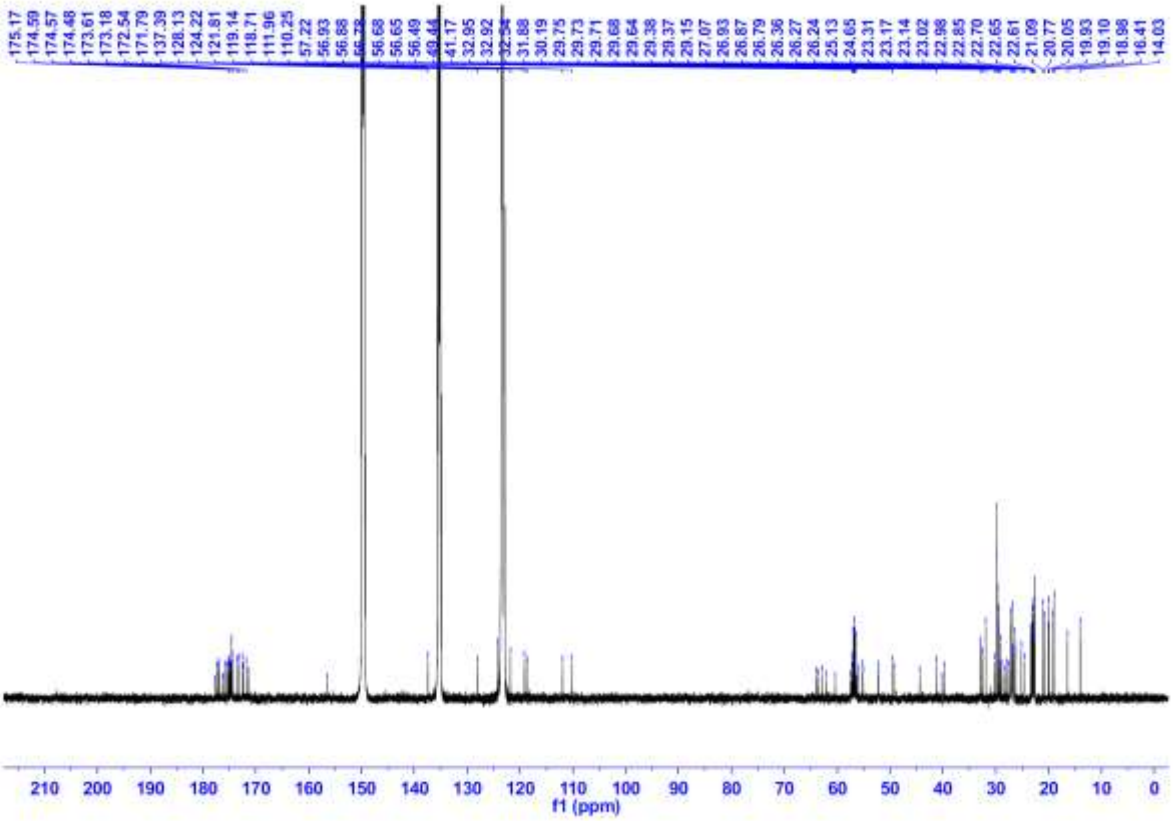

Figure S15. ^{13}C NMR spectrum of compound **2** (Pyridine-d_5, 150 MHz)

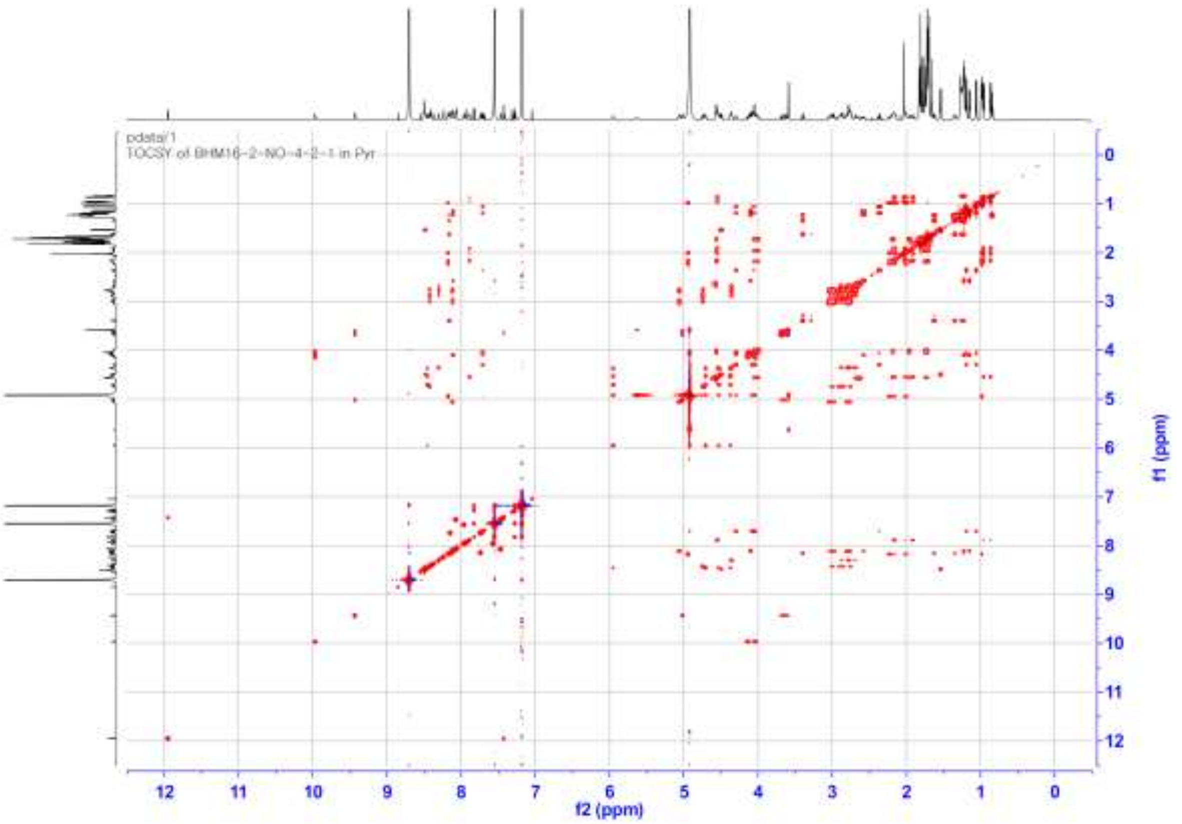

Figure S16. COSY spectrum of compound **2**

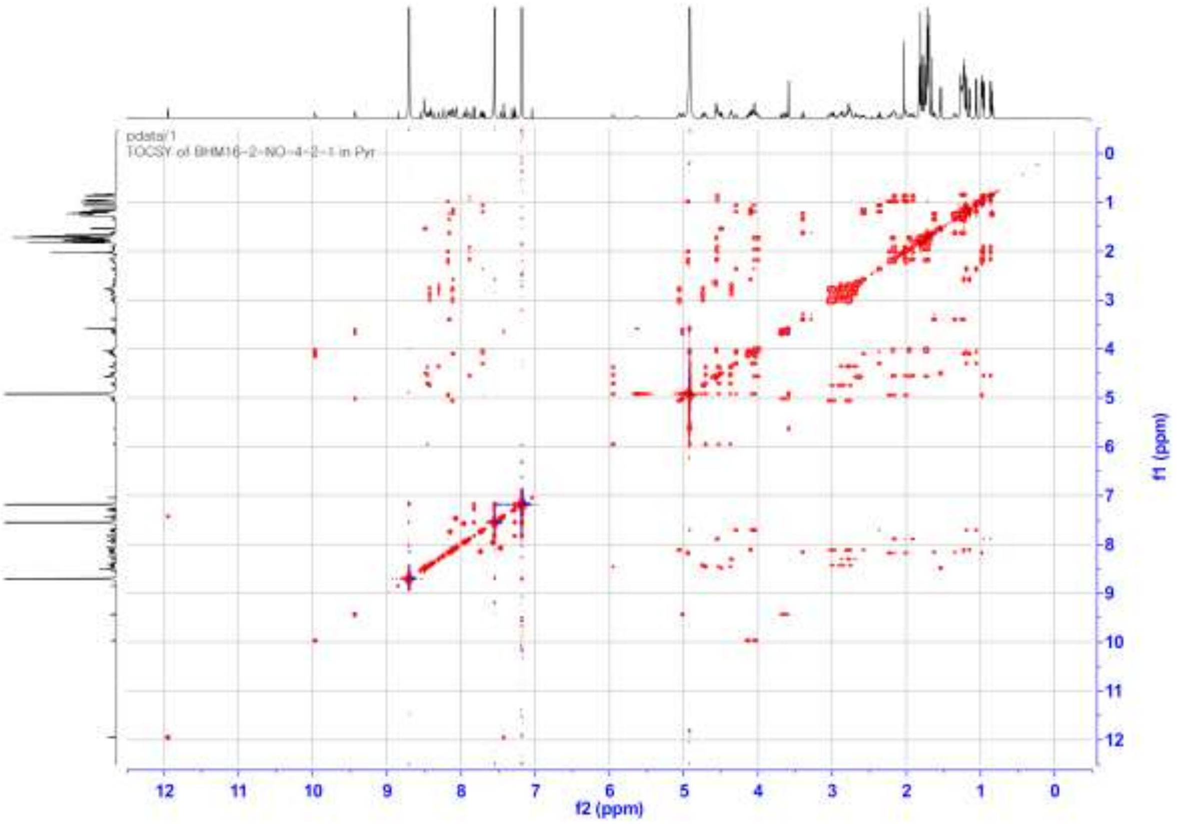

Figure S17. TOCSY spectrum of compound **2**

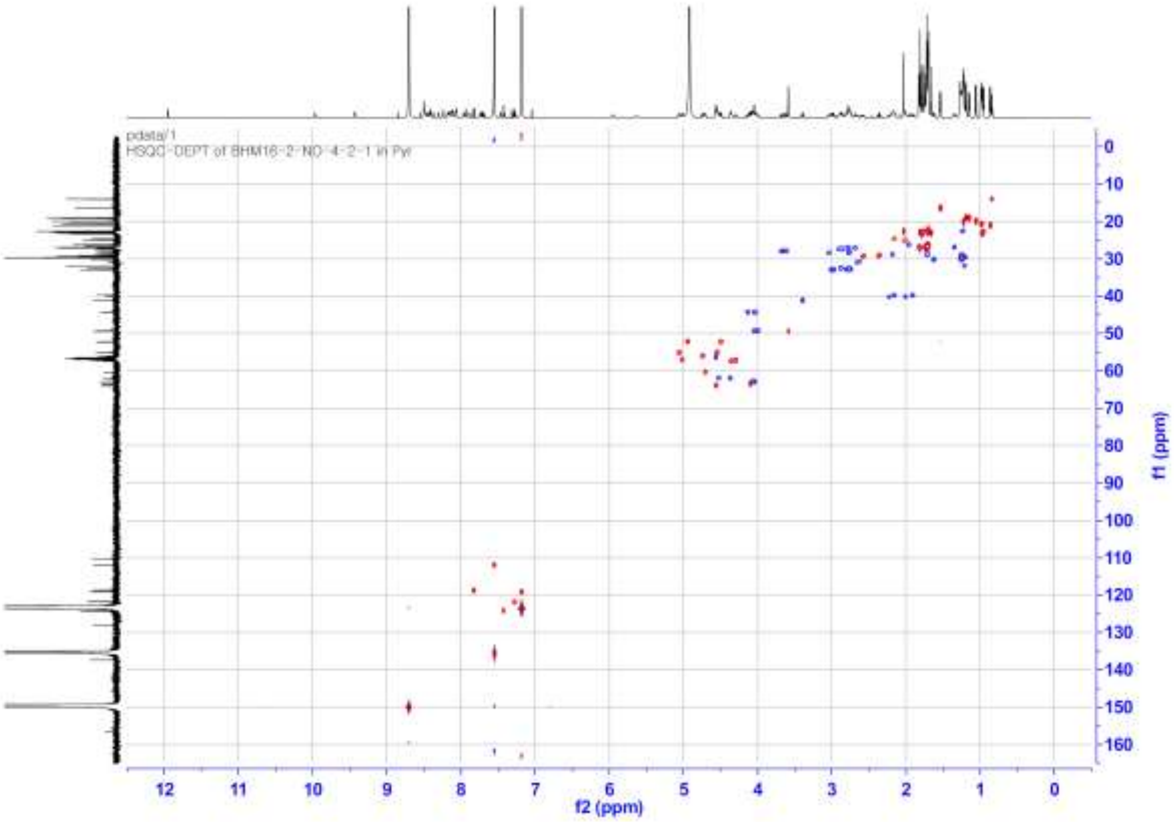

Figure S18. HSQC-DEPT spectrum of compound **2**

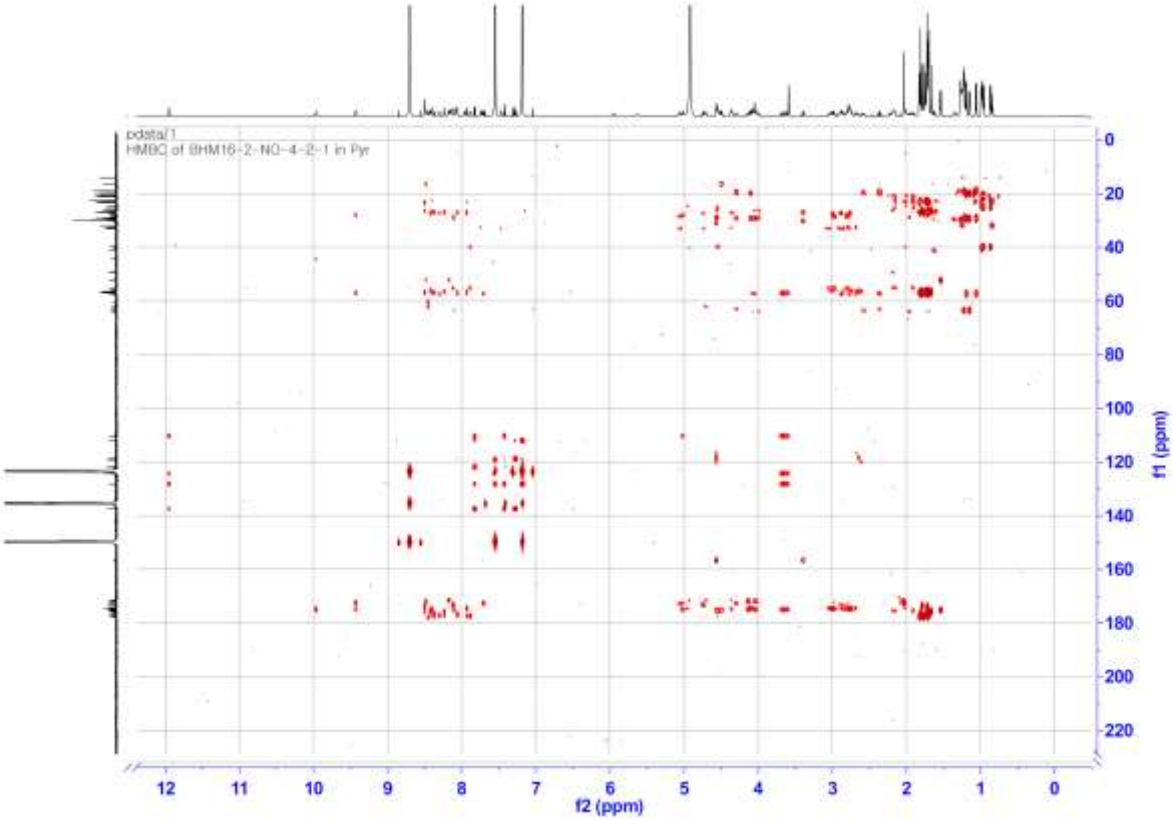

Figure S19. HMBC spectrum of compound **2**

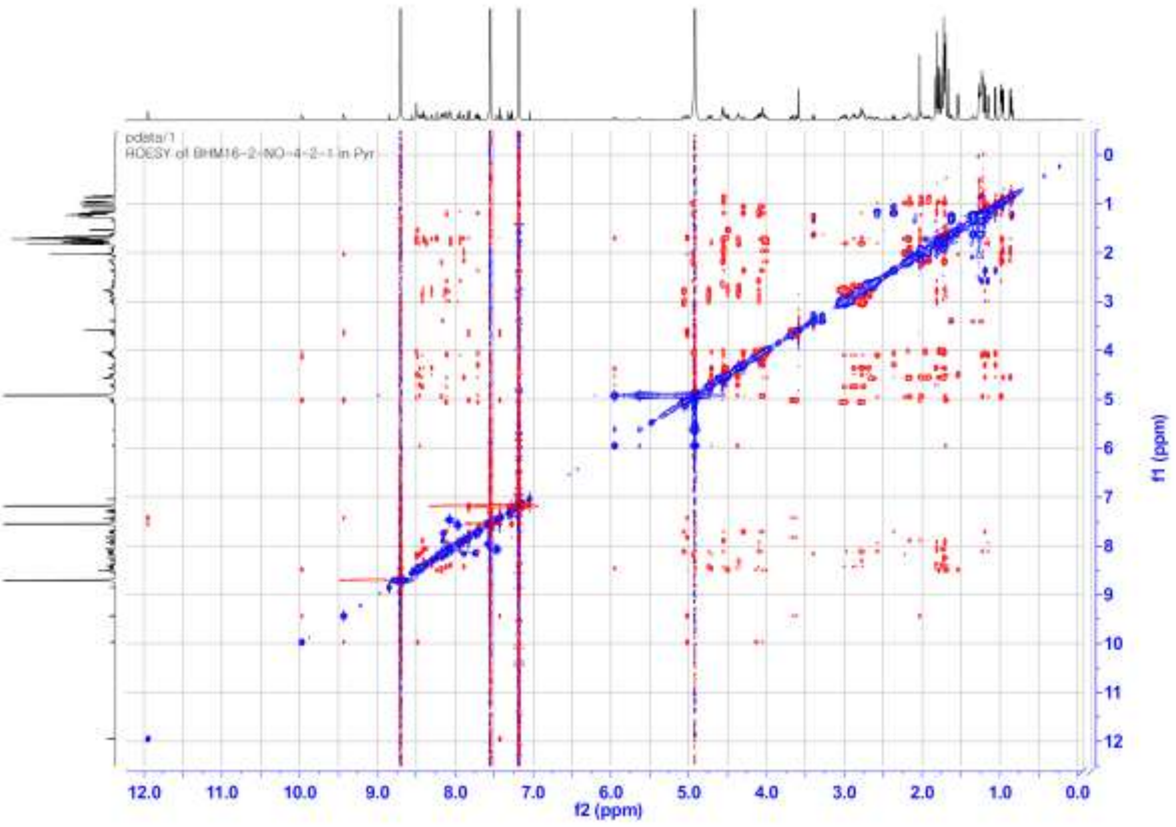

Figure S20. ROESY spectrum of compound **2**

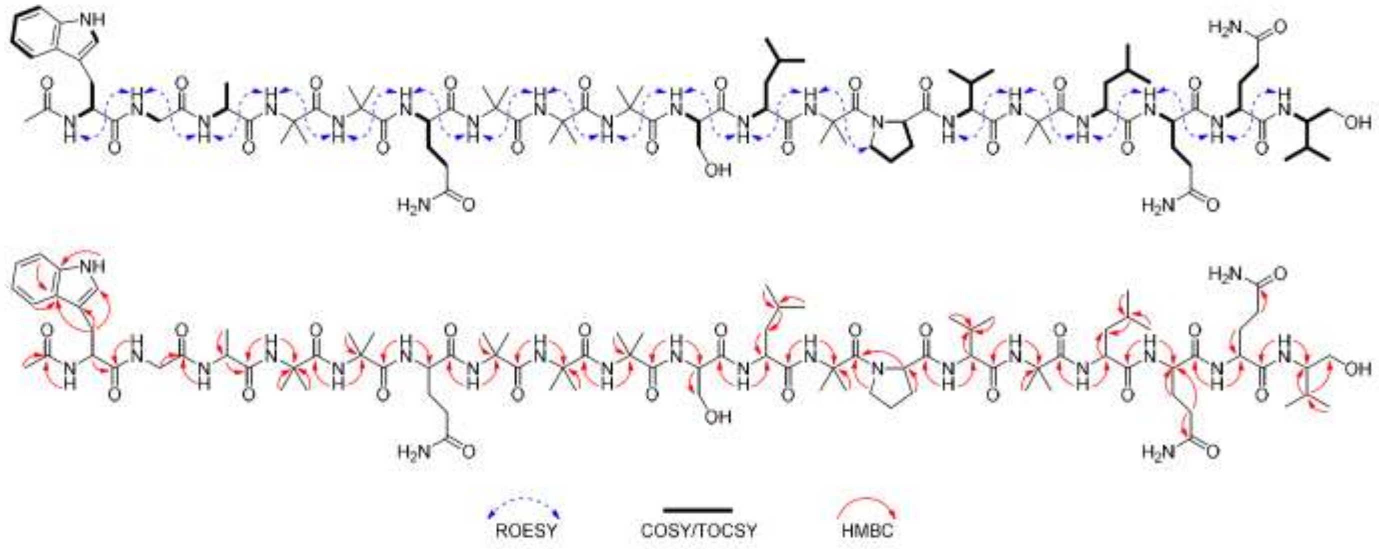

Figure S21. Key ROESY, COSY/TOCSY, and HMBC correlations of compound **2**

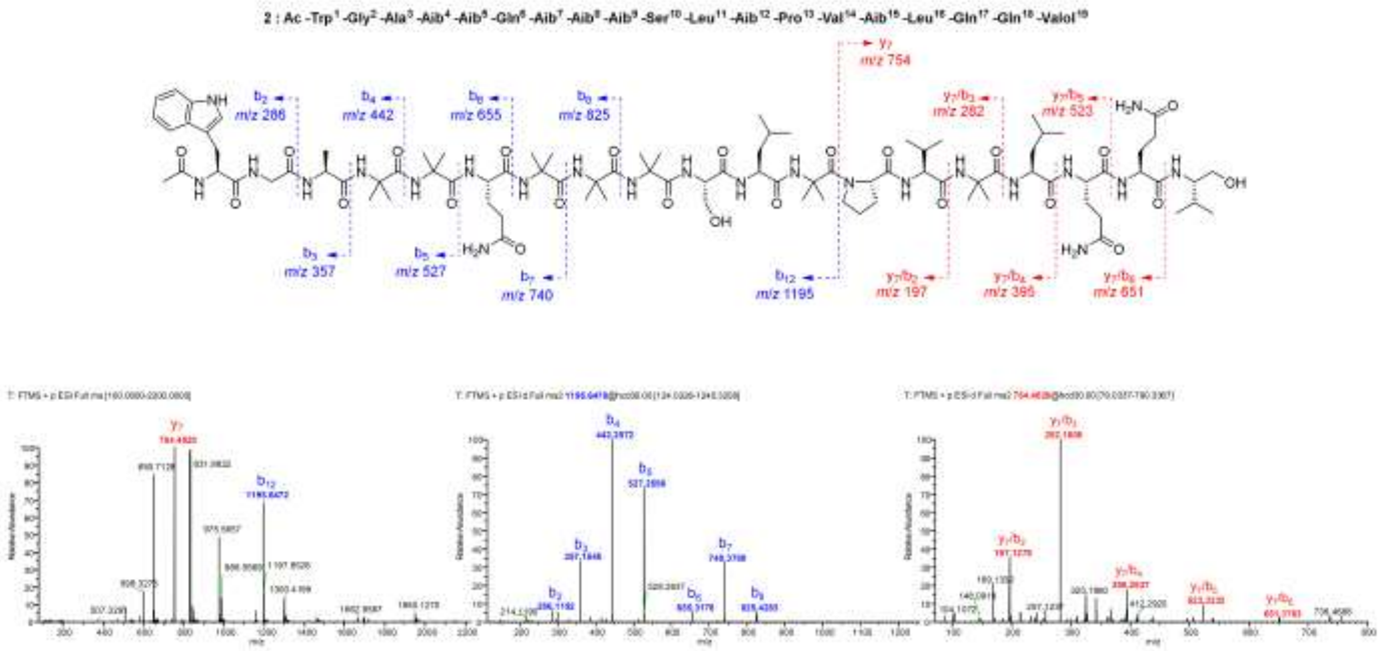

Figure S22. MS fragmentation analysis of compound **2**

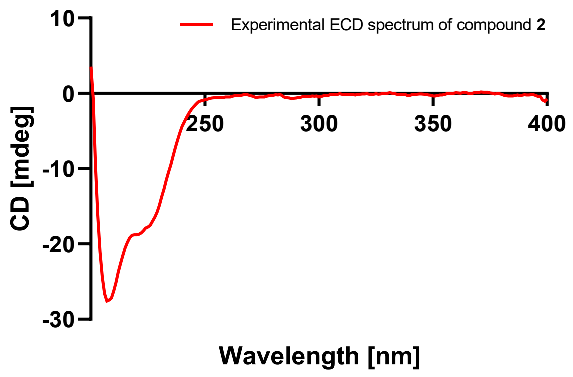

Figure S23. Experimental ECD spectrum of compound **2**

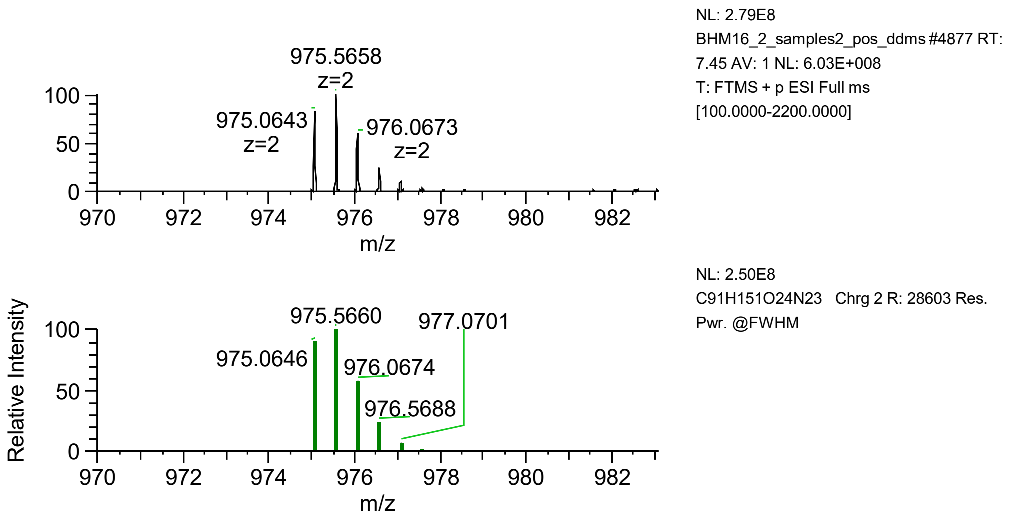

Figure S24. HRESIMS spectrum of compound **2**

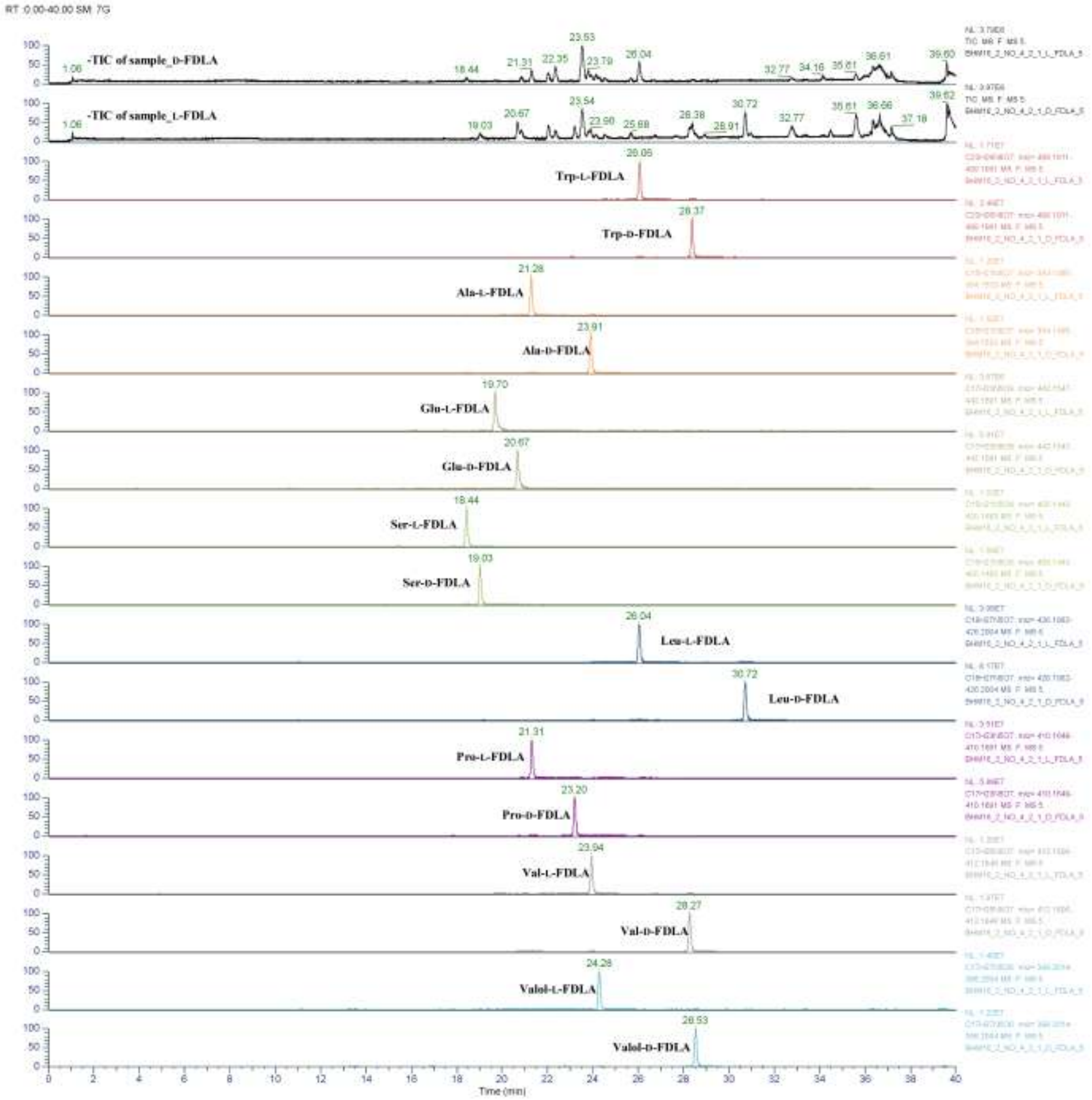

Figure S25. Advanced Marfey's analysis of compound **2**

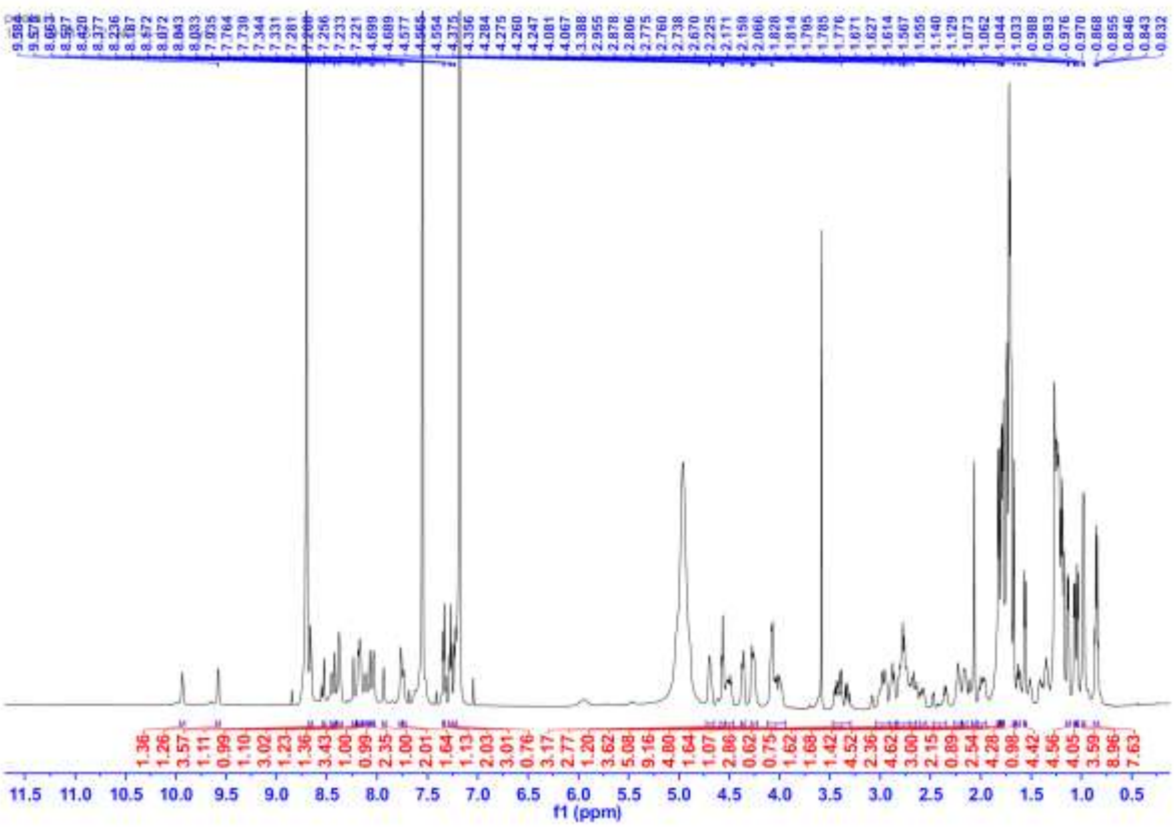

Figure S26. ^{1}H NMR spectrum of compound **3** (Pyridine-d_5, 600 MHz)

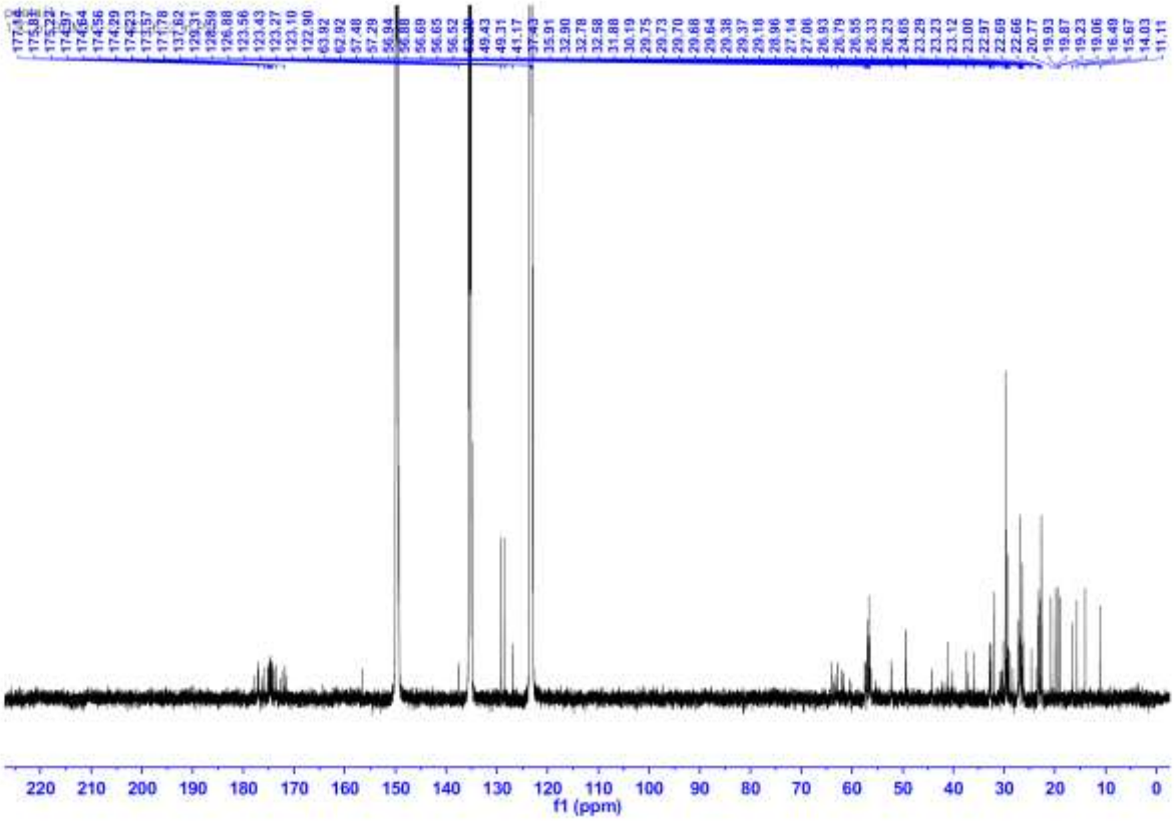

Figure S27. ^{13}C NMR spectrum of compound **3** (Pyridine-d_5, 150 MHz)

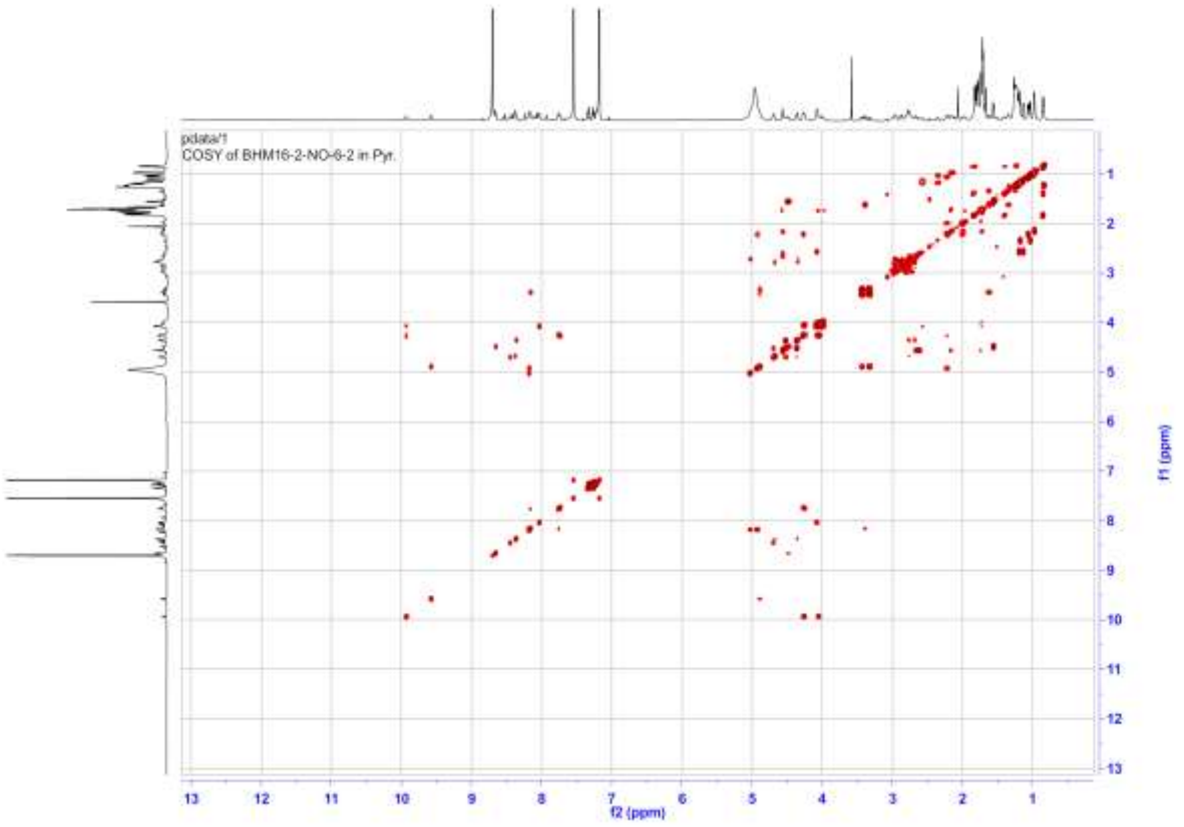

Figure S28. COSY spectrum of compound **3**

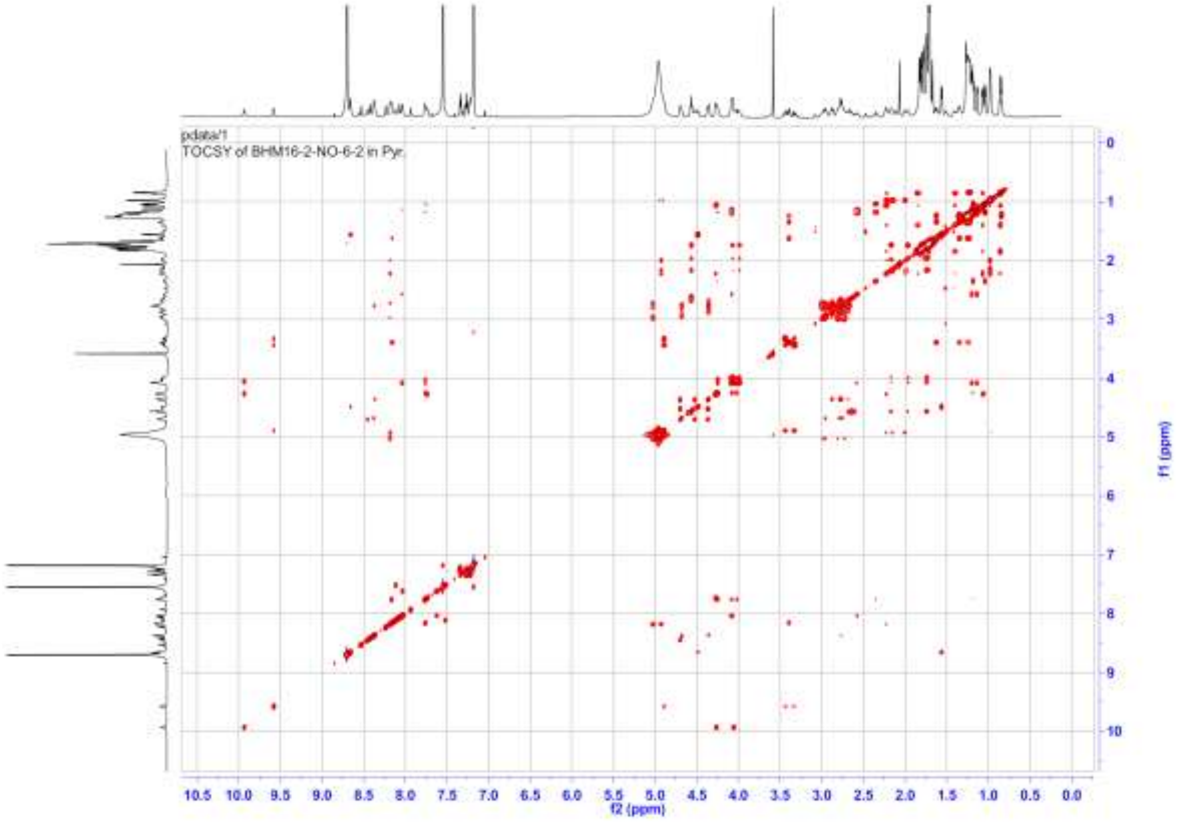

Figure S29. TOCSY spectrum of compound **3**

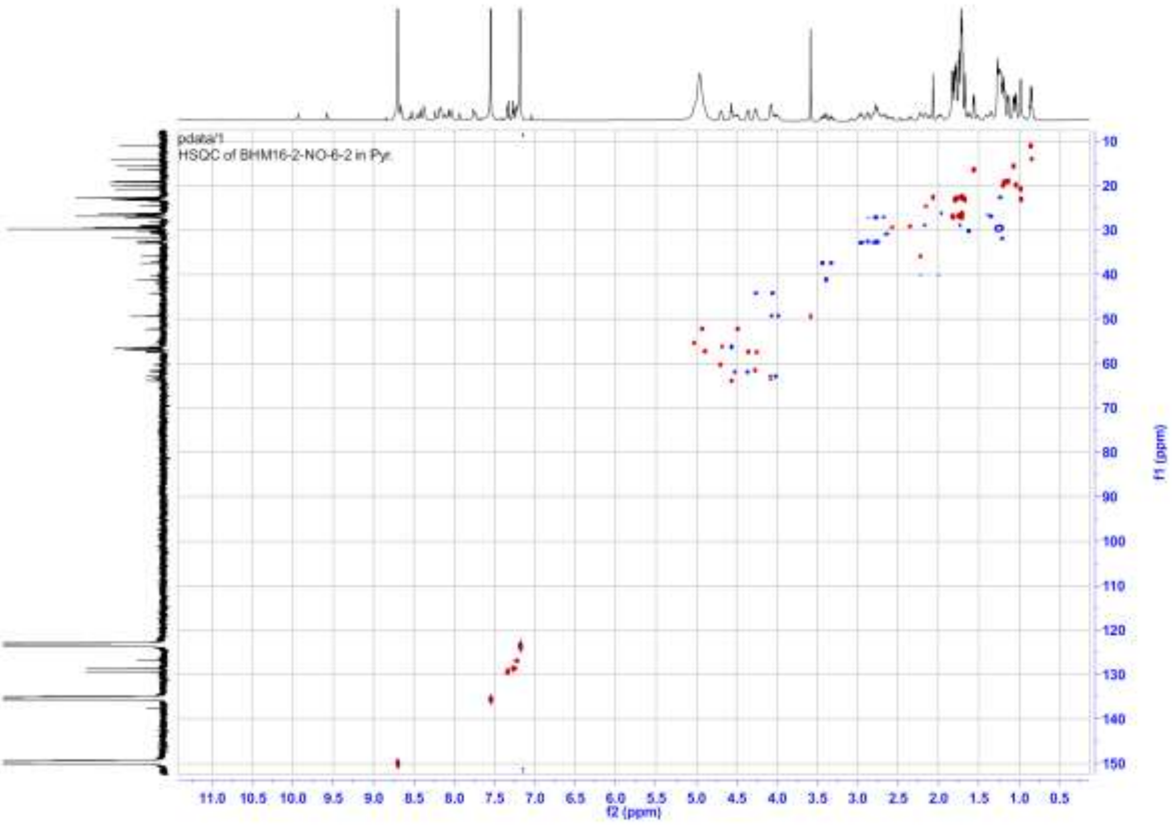

Figure S30. HSQC-DEPT spectrum of compound **3**

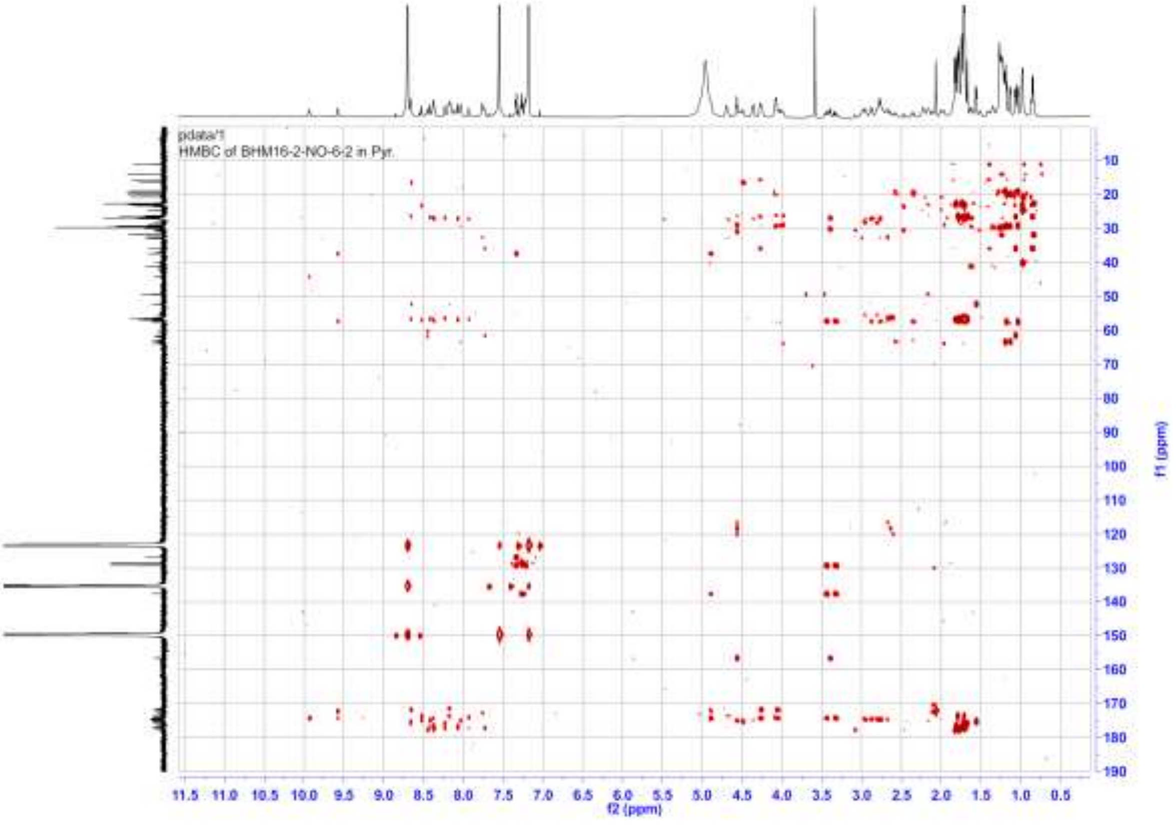

Figure S31. HMBC spectrum of compound **3**

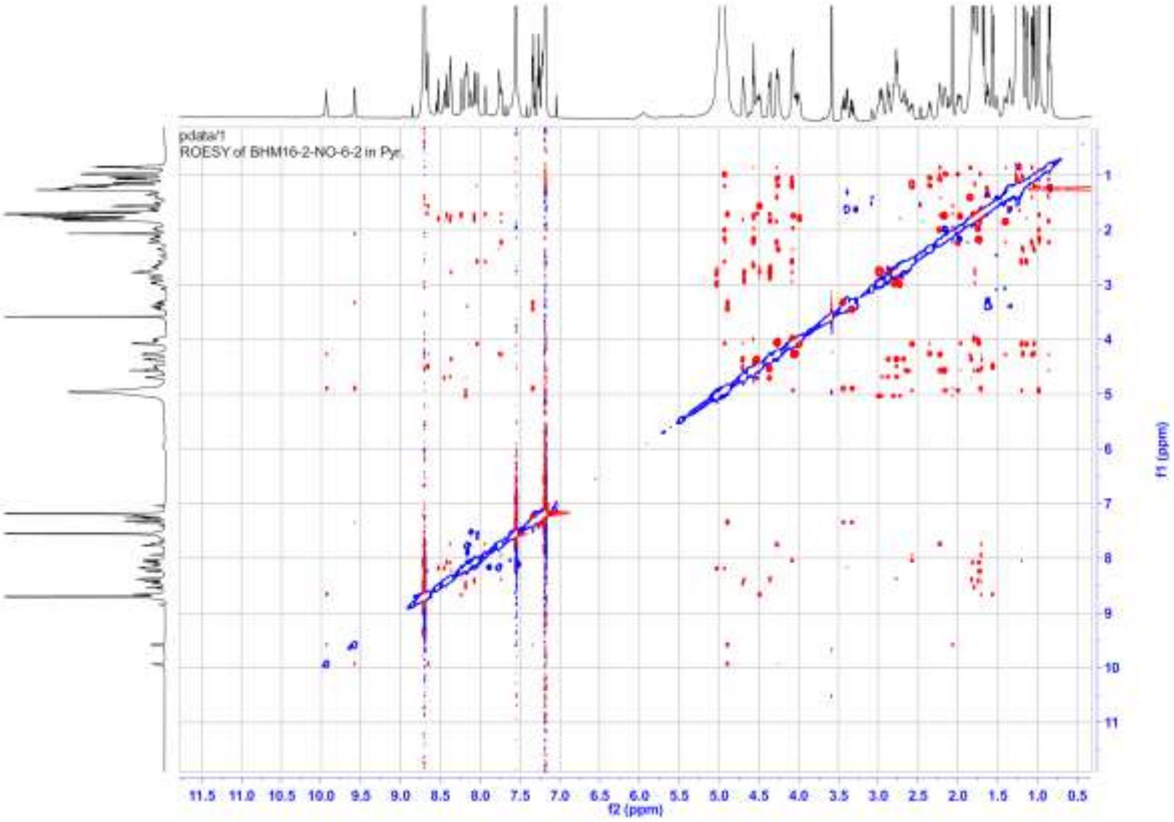

Figure S32. ROESY spectrum of compound **3**

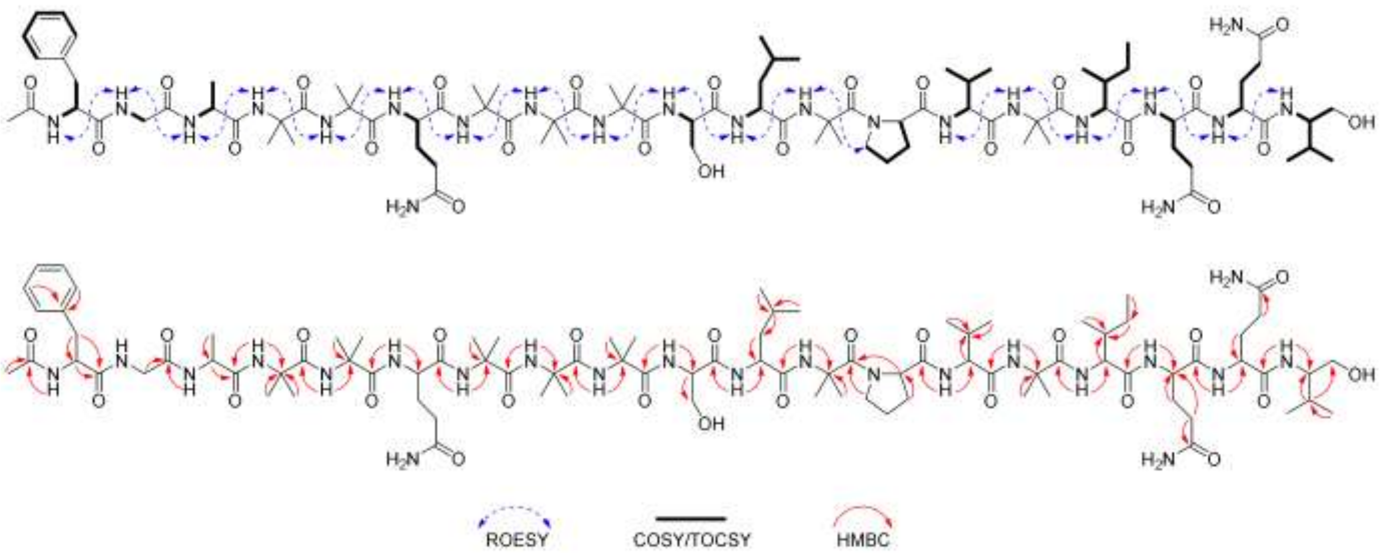

Figure S33. Key ROESY, COSY/TOCSY, and HMBC correlations of compound **3**

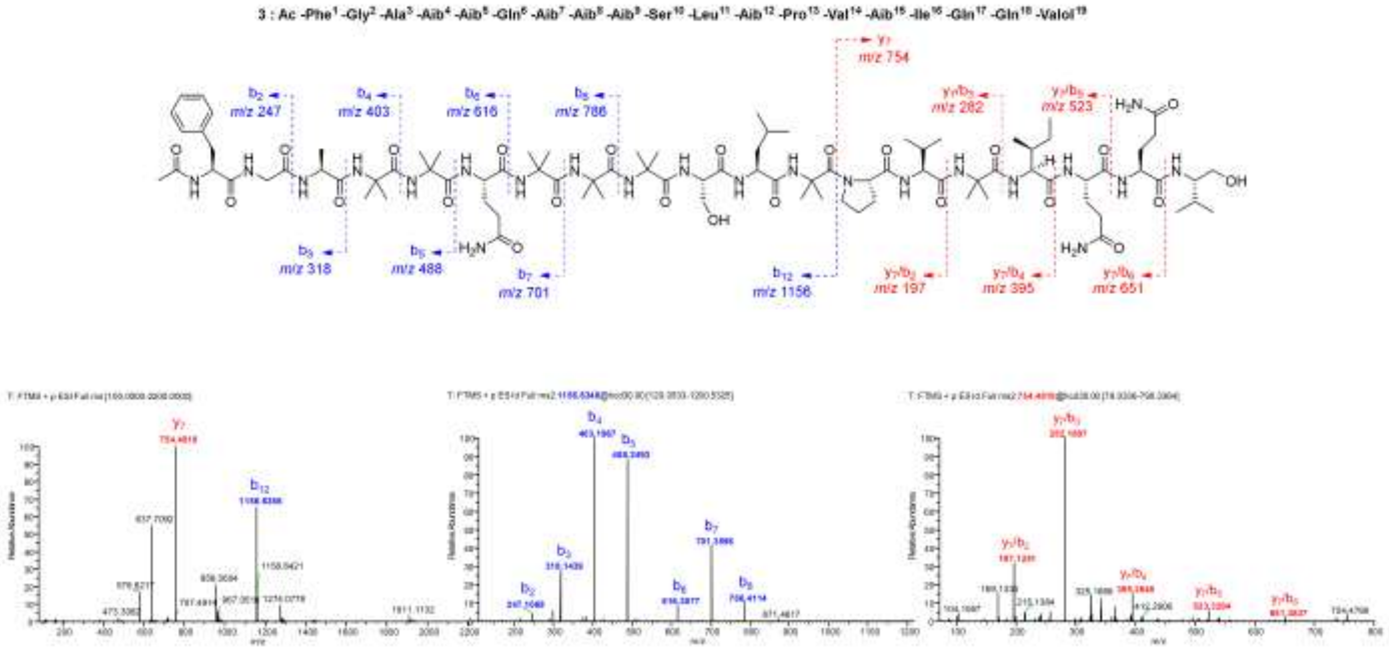

Figure S34. MS fragmentation analysis of compound **3**

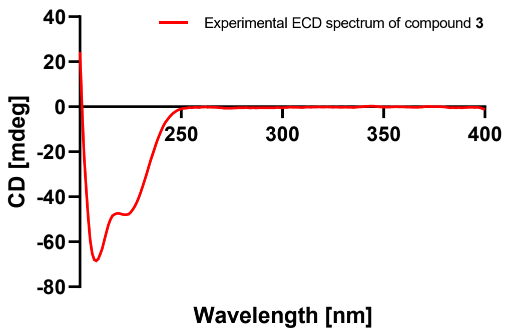

Figure S35. Experimental ECD spectrum of compound **3**

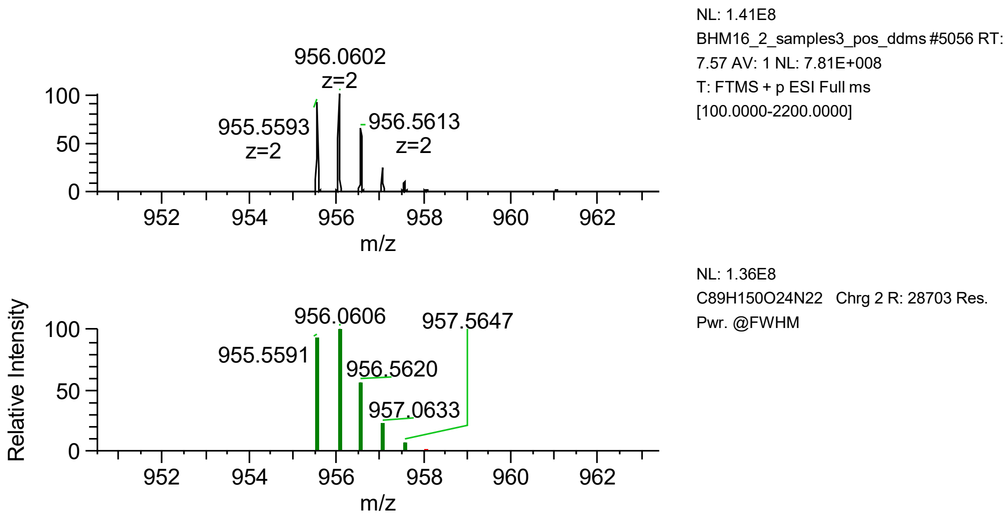

Figure S36. HRESIMS spectrum of compound **3**

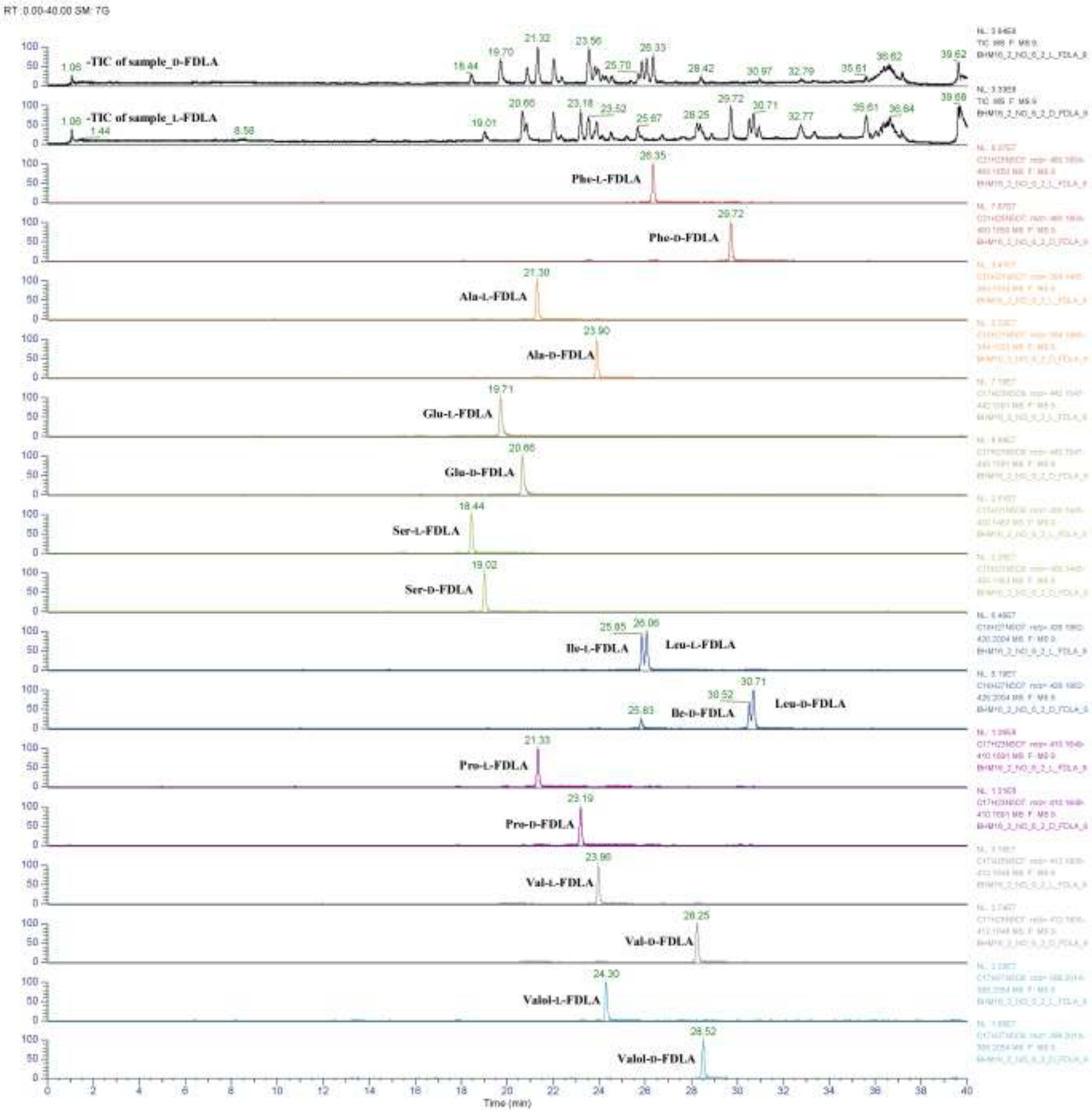

Figure S37. Advanced Marfey's analysis of compound **3**

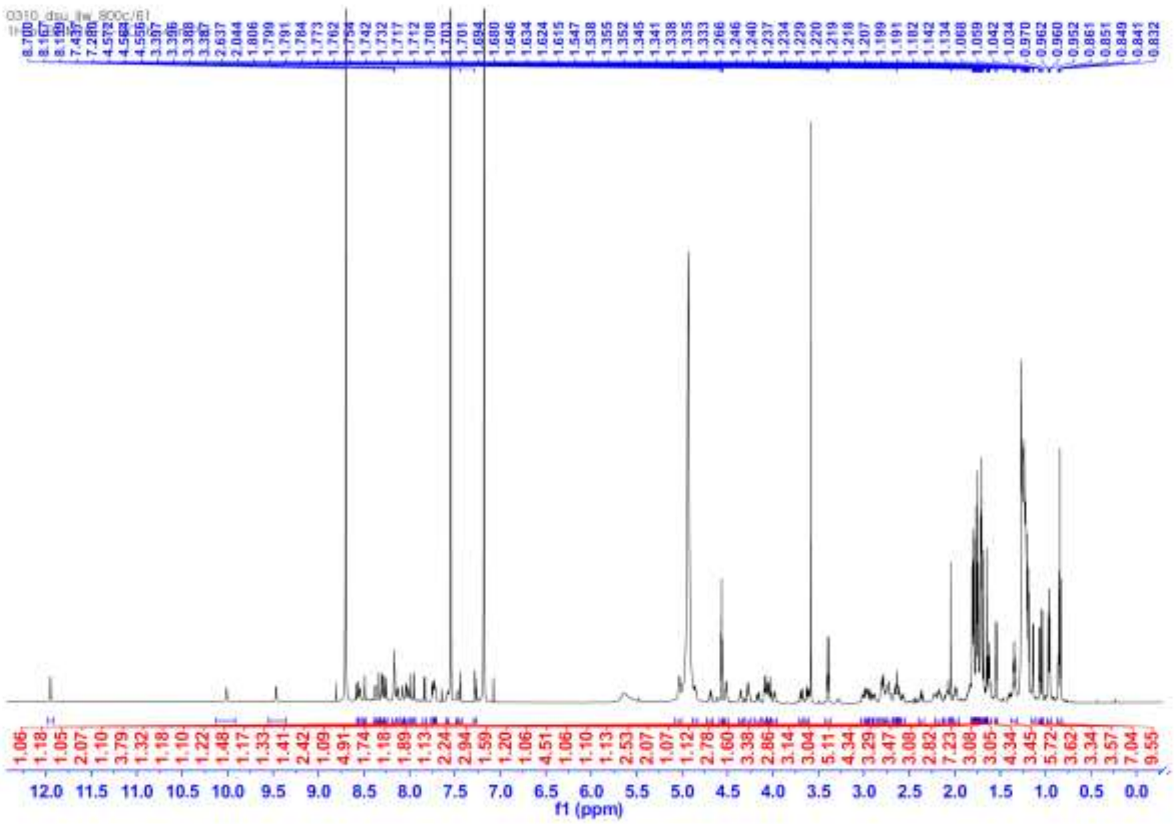

Figure S38. ^{1}H NMR spectrum of compound **4** (Pyridine-d_5, 800 MHz)

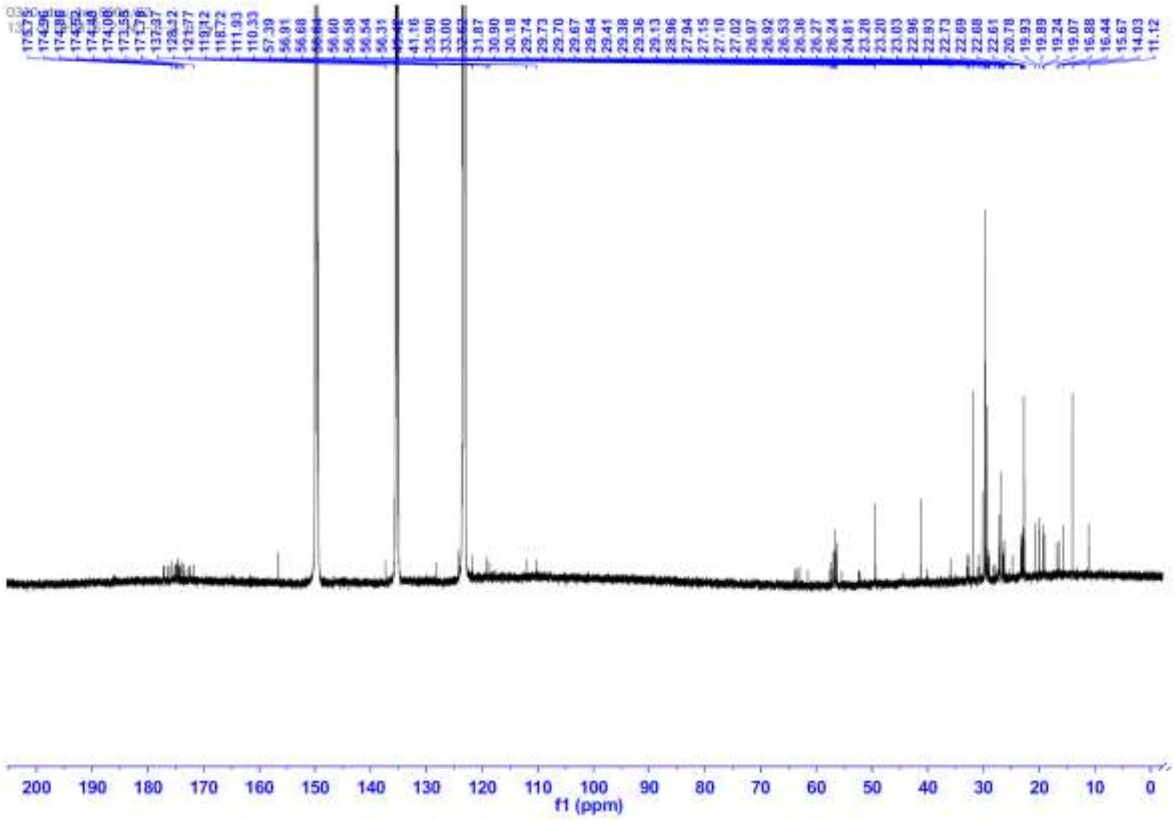

Figure S39. ^{13}C NMR spectrum of compound **4** (Pyridine-d_5, 200 MHz)

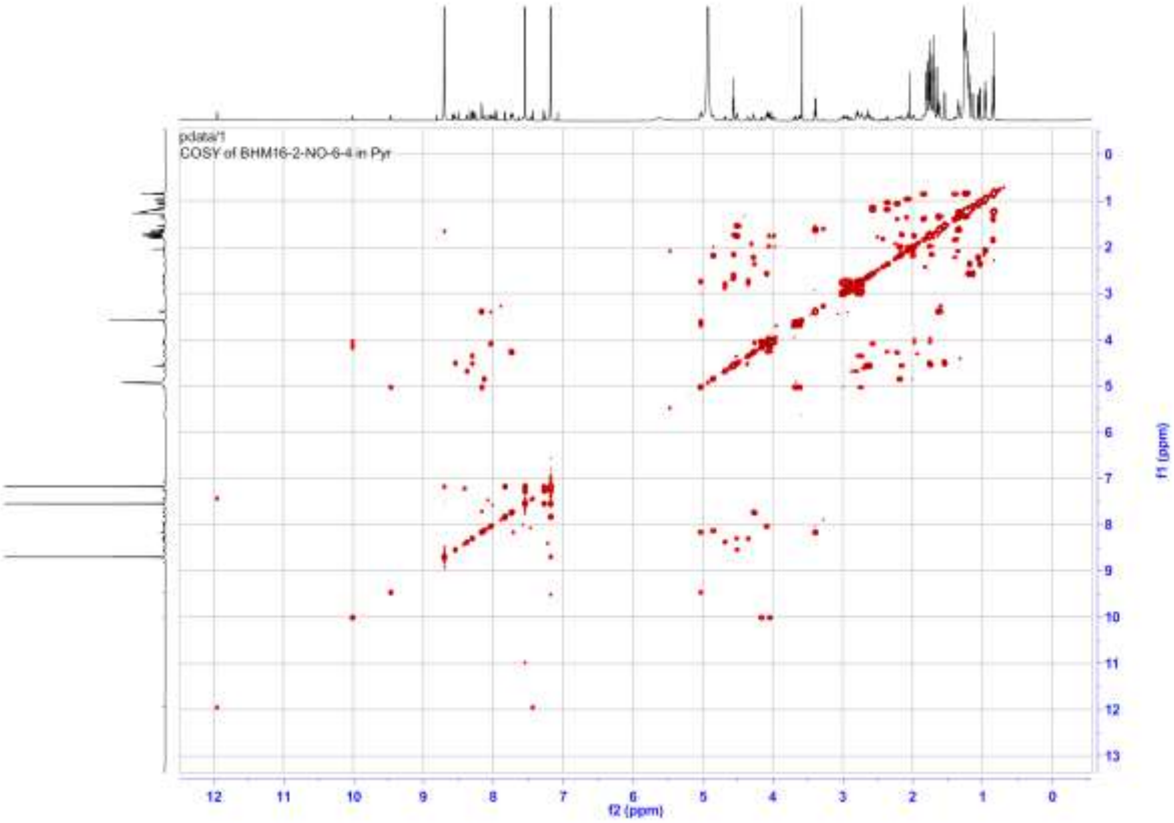

Figure S40. COSY spectrum of compound **4**

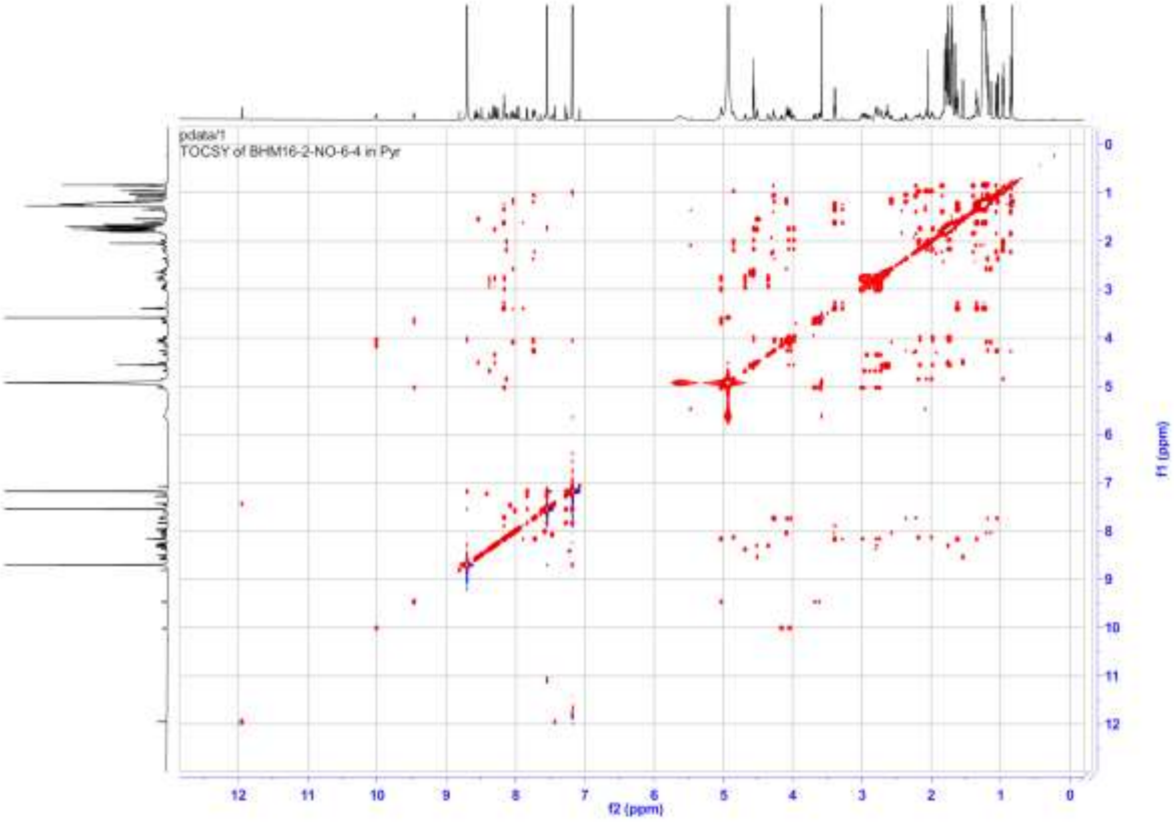

Figure S41. TOCSY spectrum of compound **4**

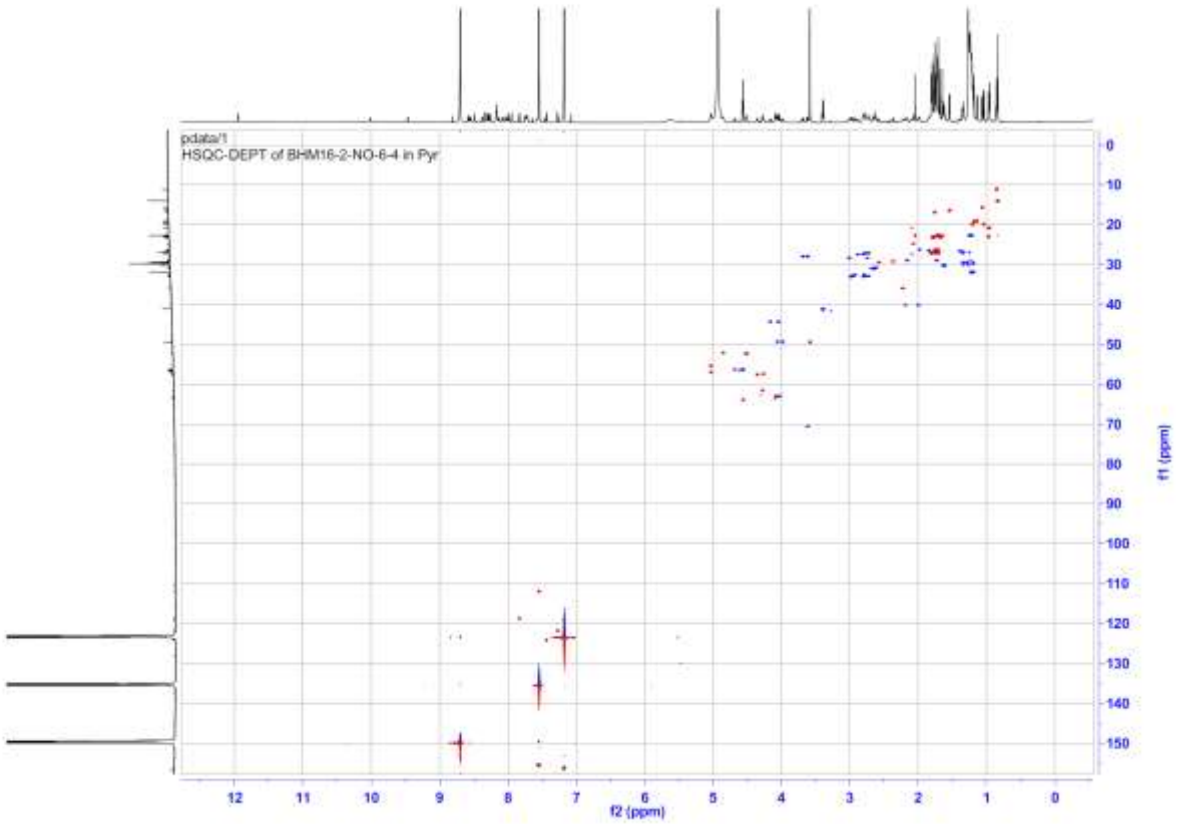

Figure S42. HSQC-DEPT spectrum of compound **4**

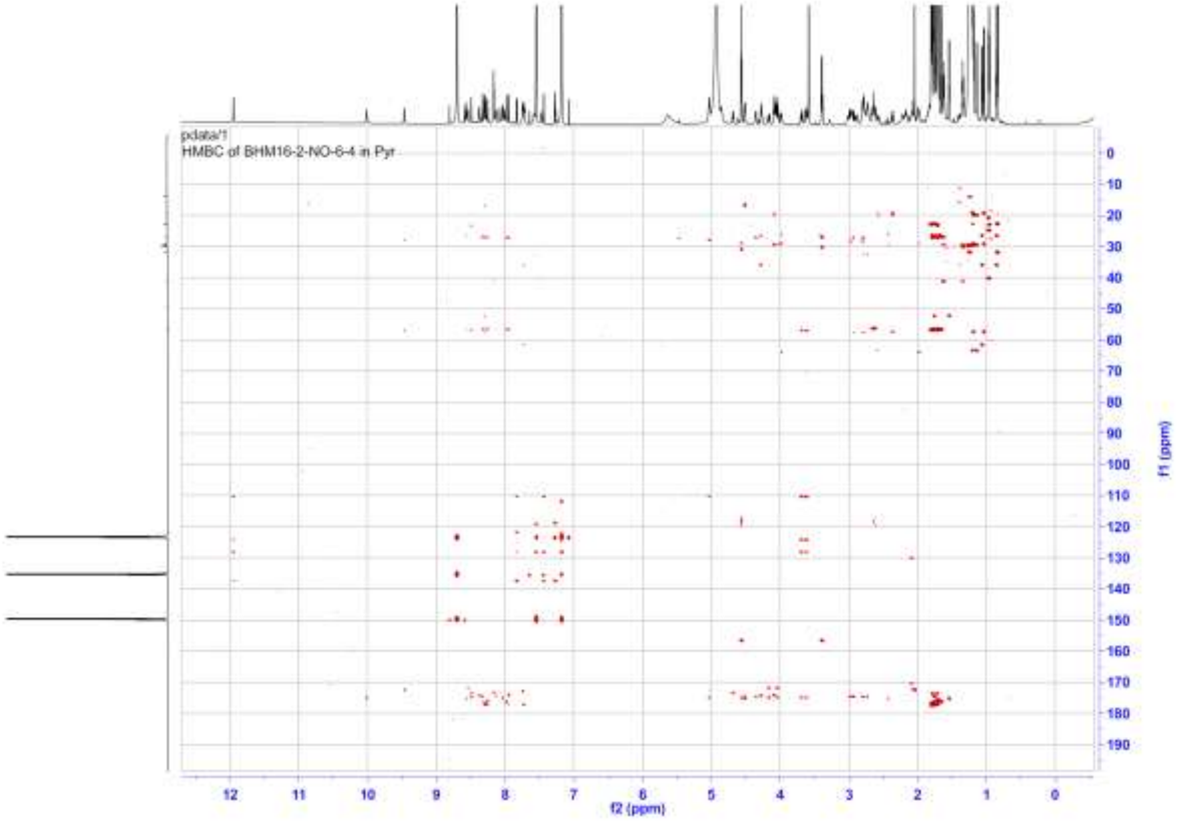

Figure S43. HMBC spectrum of compound **4**

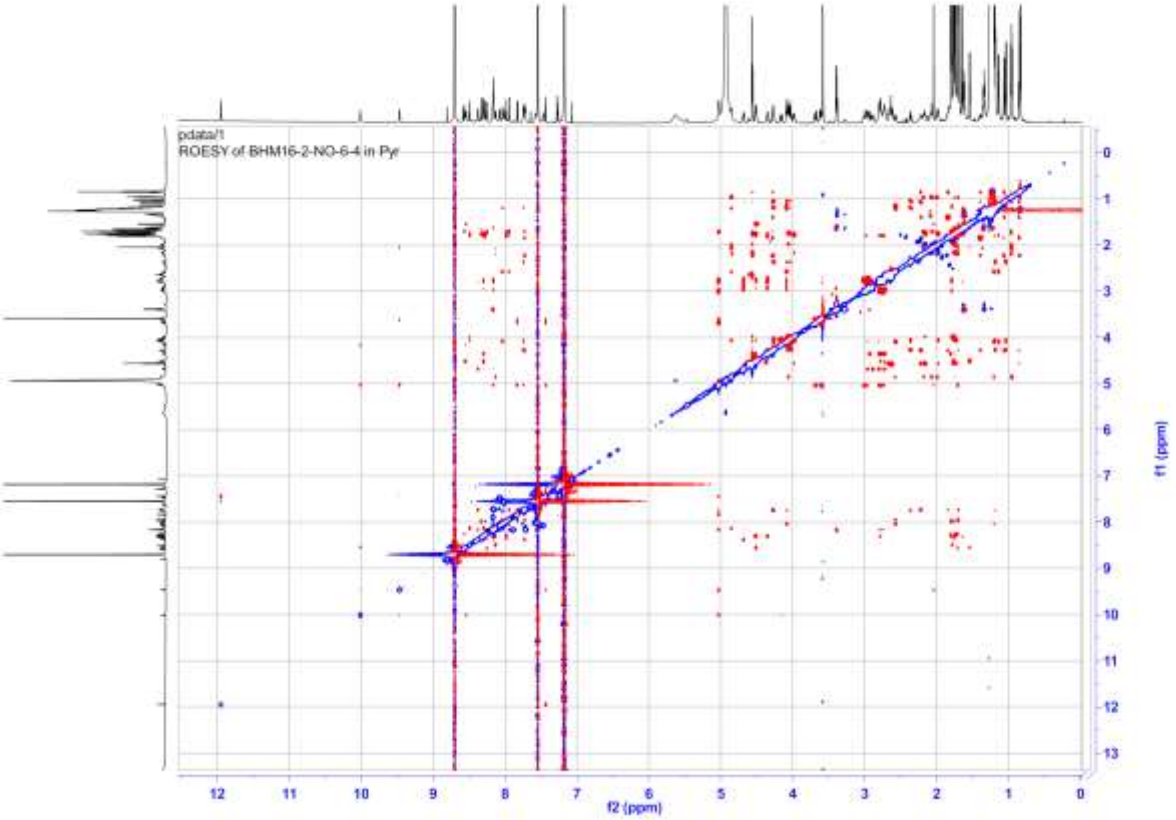

Figure S44. ROESY spectrum of compound **4**

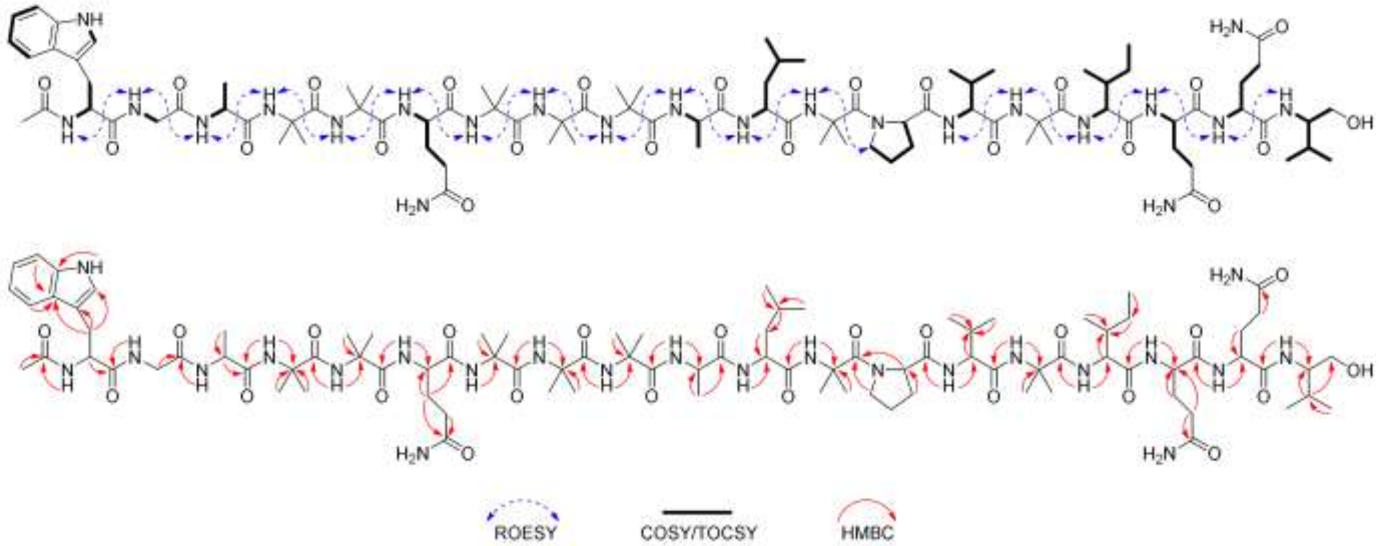

Figure S45. Key ROESY, COSY/TOCSY, and HMBC correlations of compound **4** **Figure S46**. MS

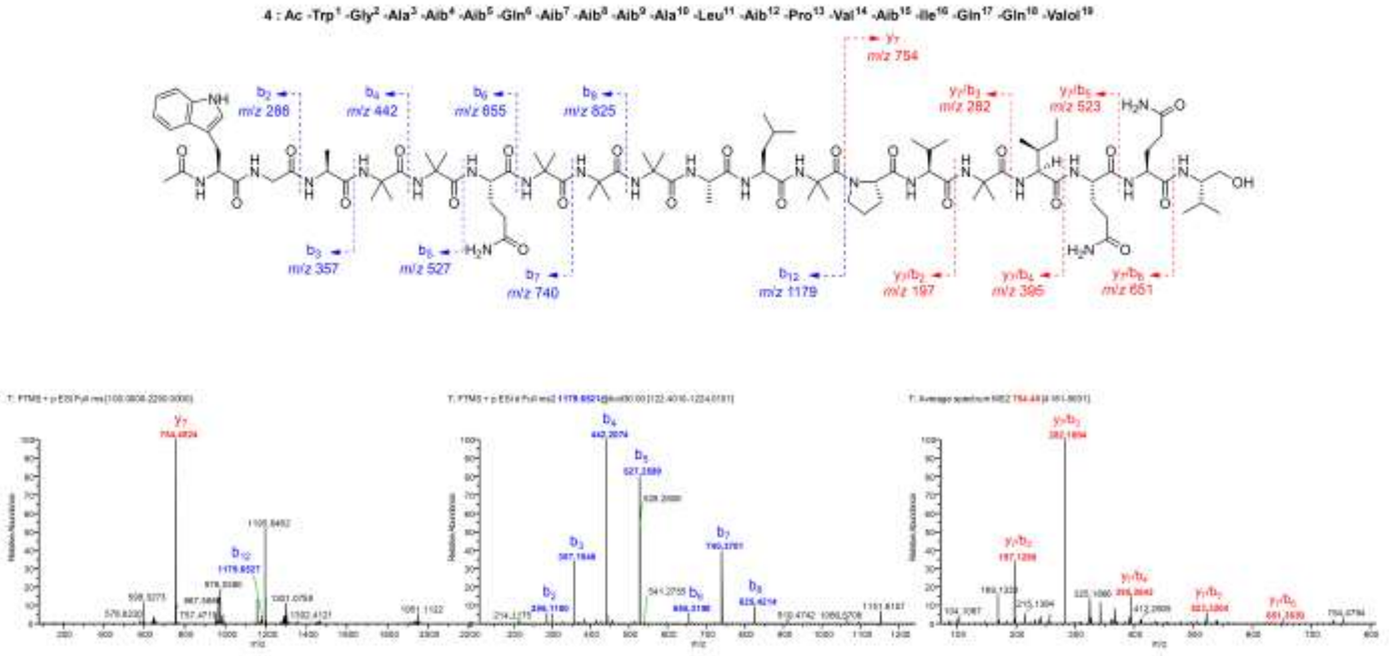

fragmentation analysis of compound **4**

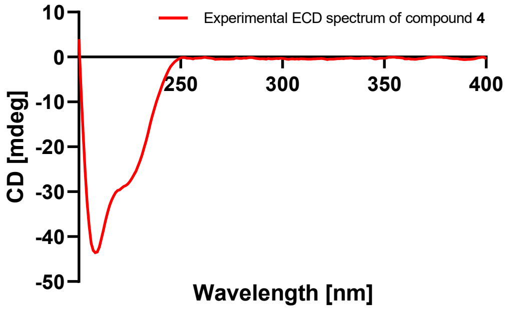

Figure S47. Experimental ECD spectrum of compound **4**

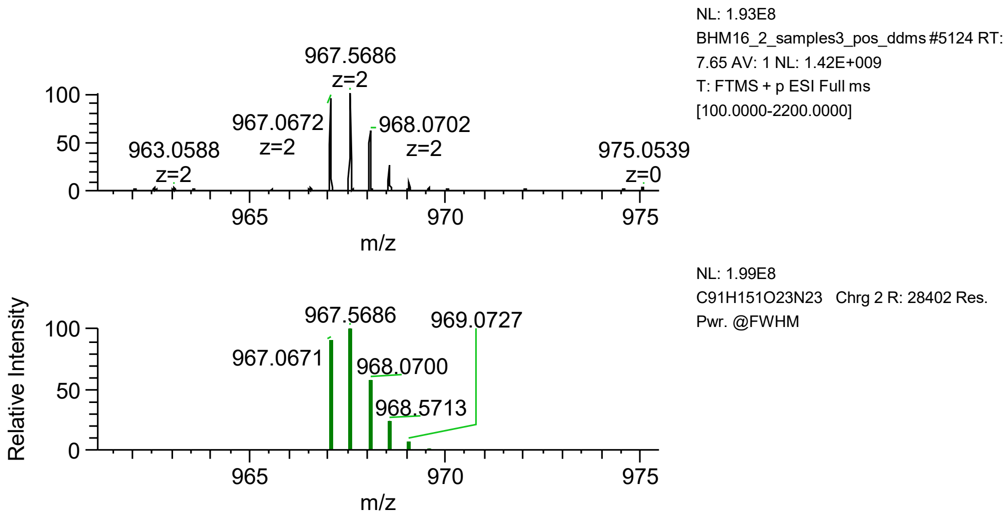

Figure S48. HRESIMS spectrum of compound **4**

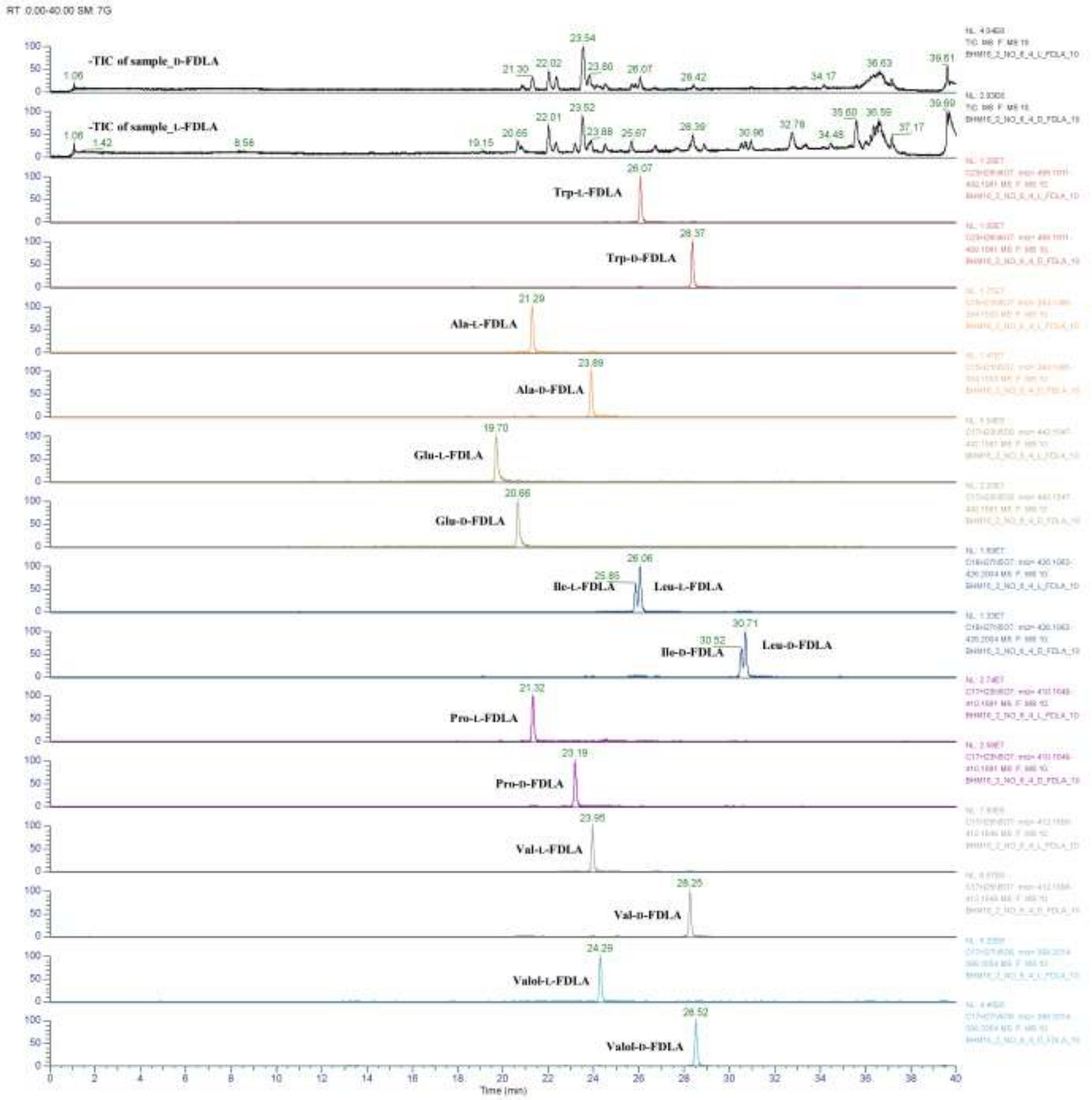

Figure S49. Advanced Marfey’s analysis of compound **4**

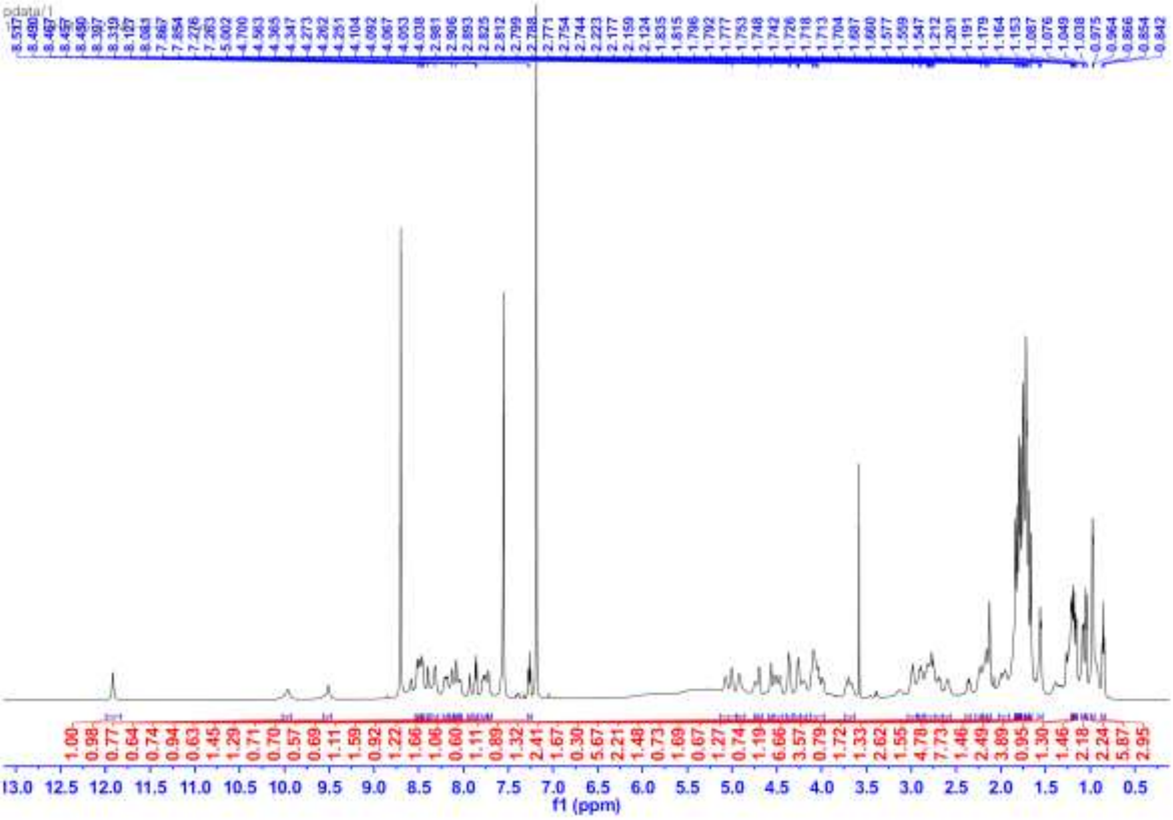

Figure S50. ^{1}H NMR spectrum of compound **5** (Pyridine-d_5, 600 MHz)

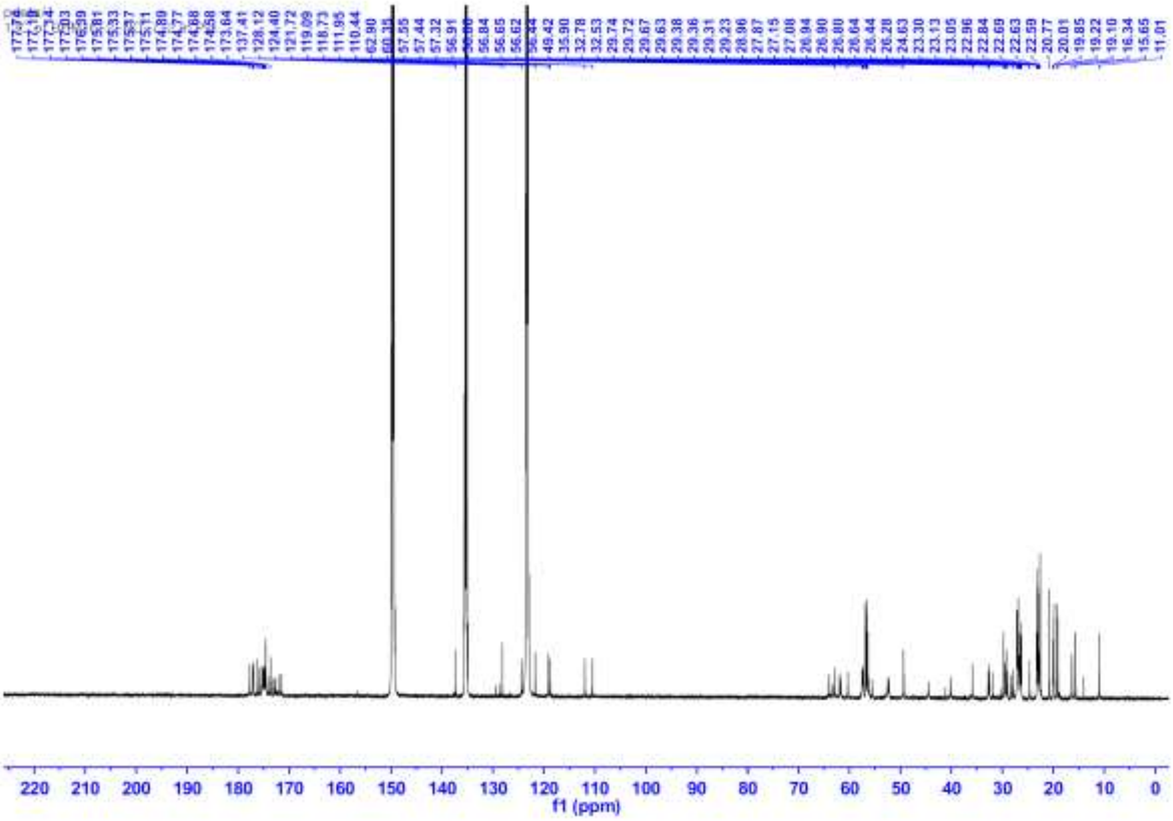

Figure S51. ^{13}C NMR spectrum of compound **5** (Pyridine-d_5, 150 MHz)

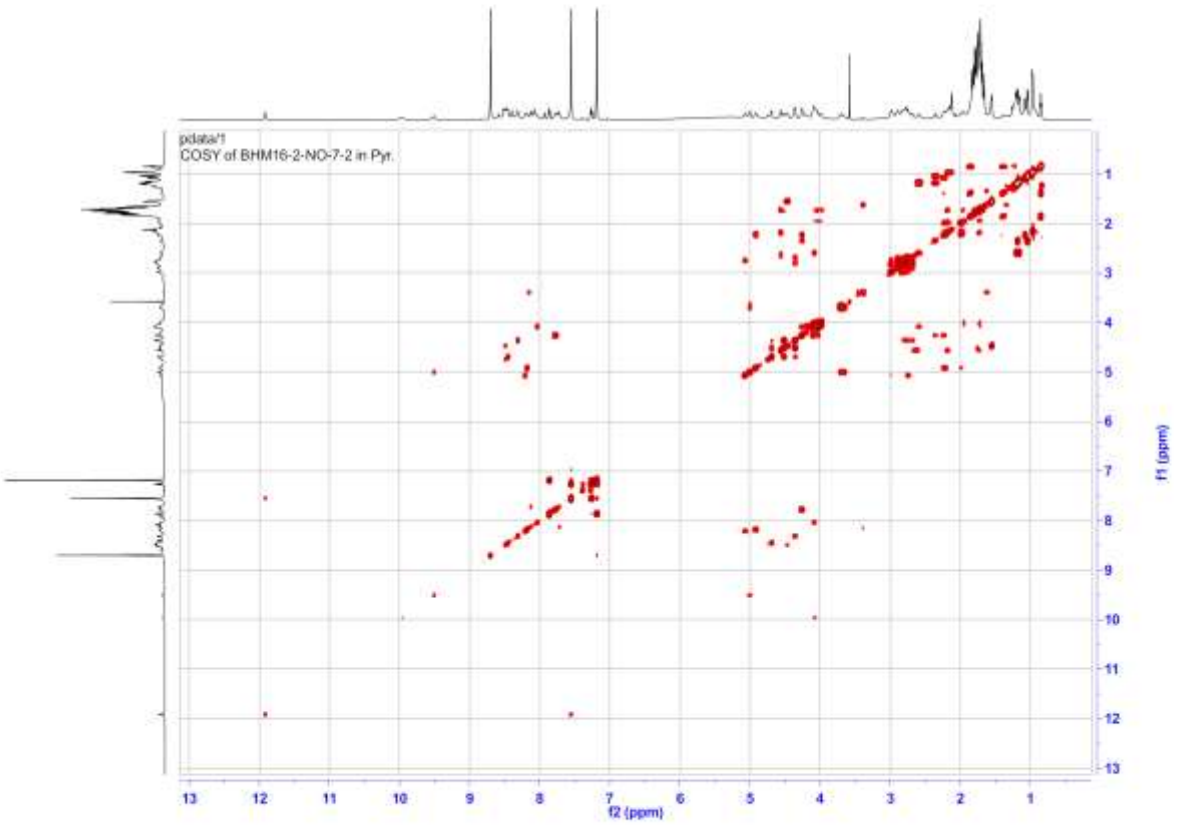

Figure S52. COSY spectrum of compound **5**

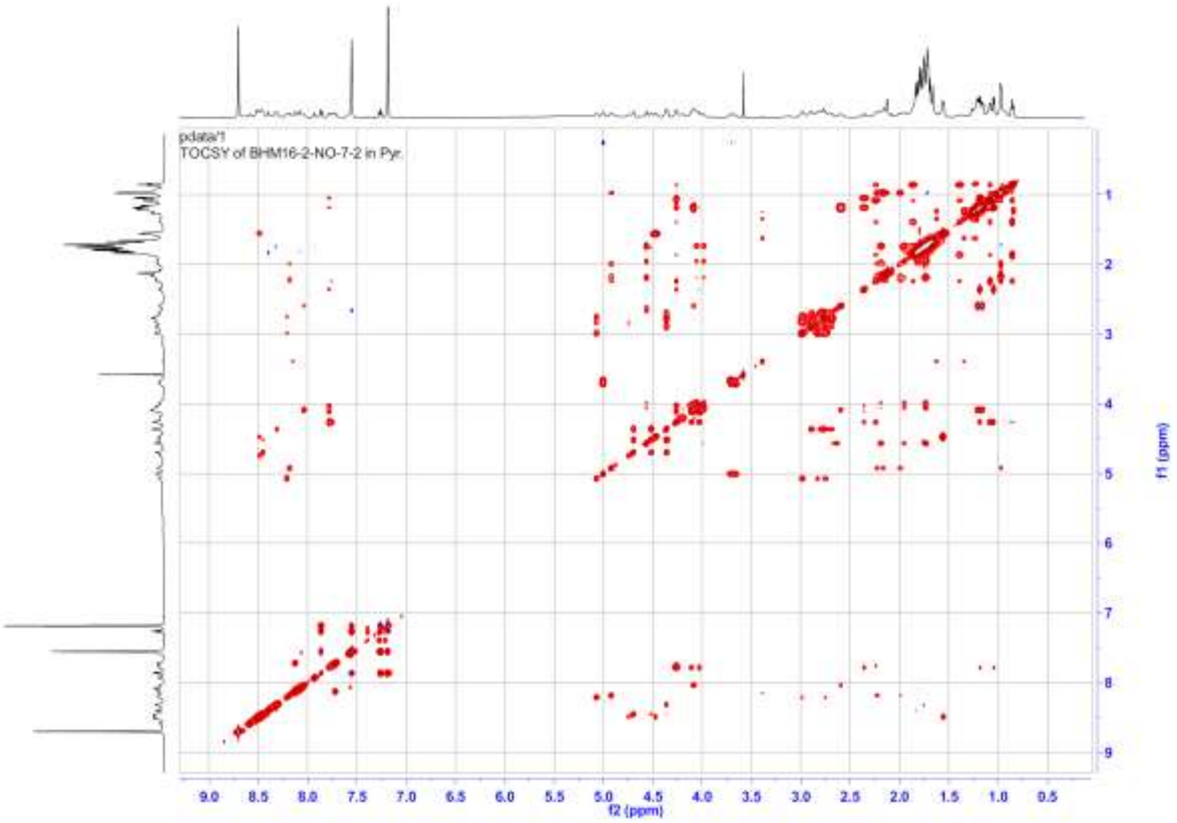

Figure S53. TOCSY spectrum of compound **5**

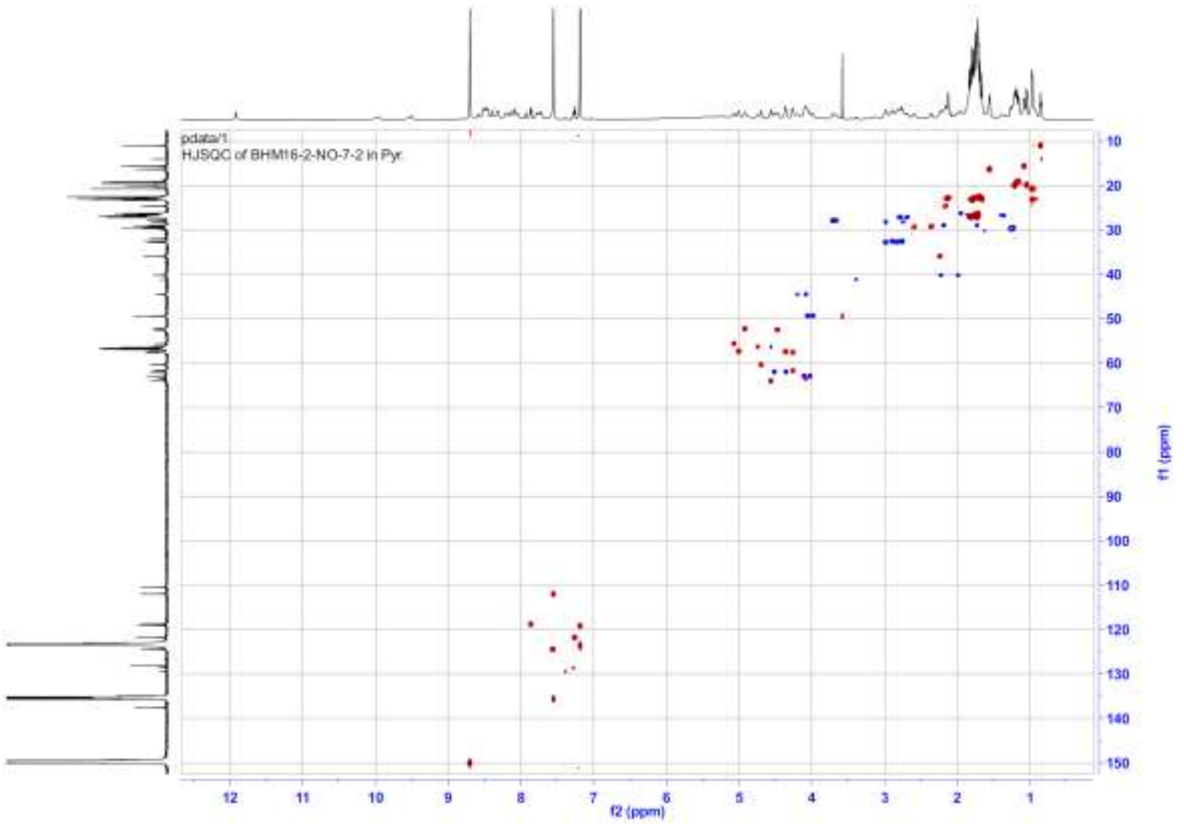

Figure S54. HSQC-DEPT spectrum of compound **5**

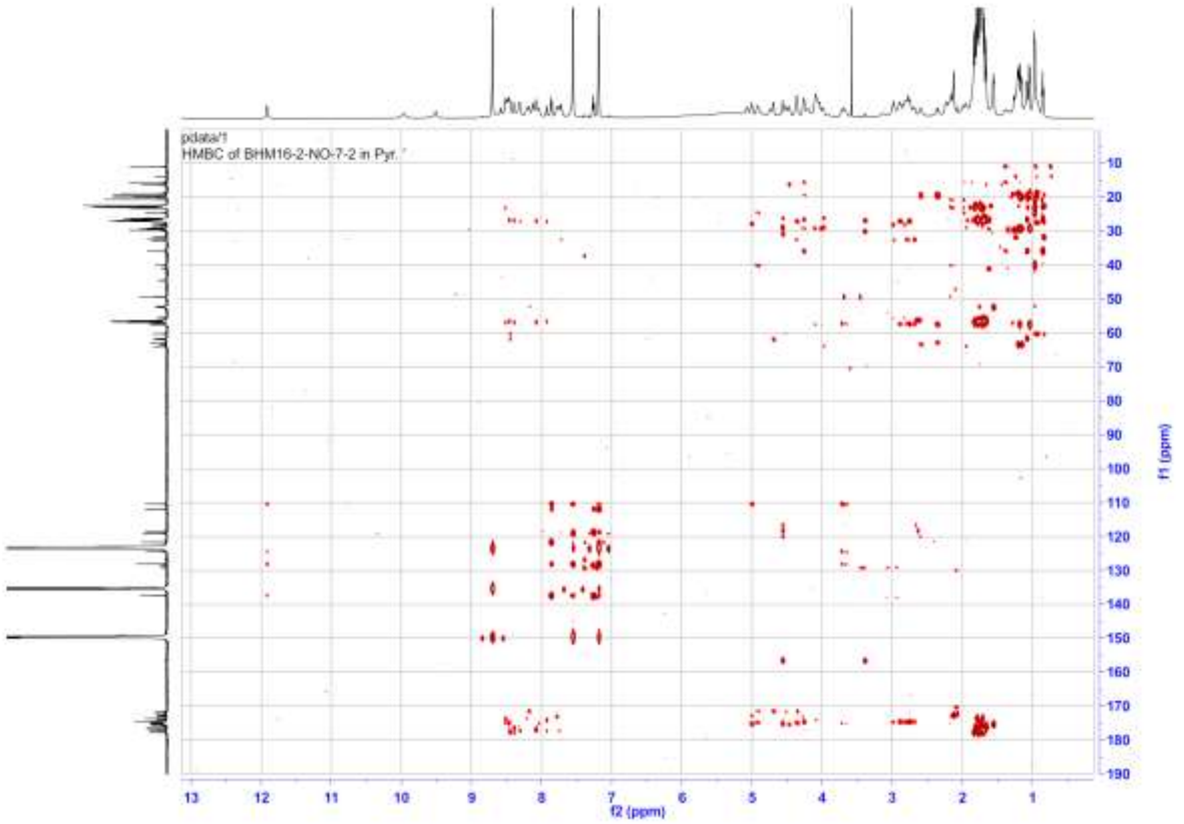

Figure S55. HMBC spectrum of compound **5**

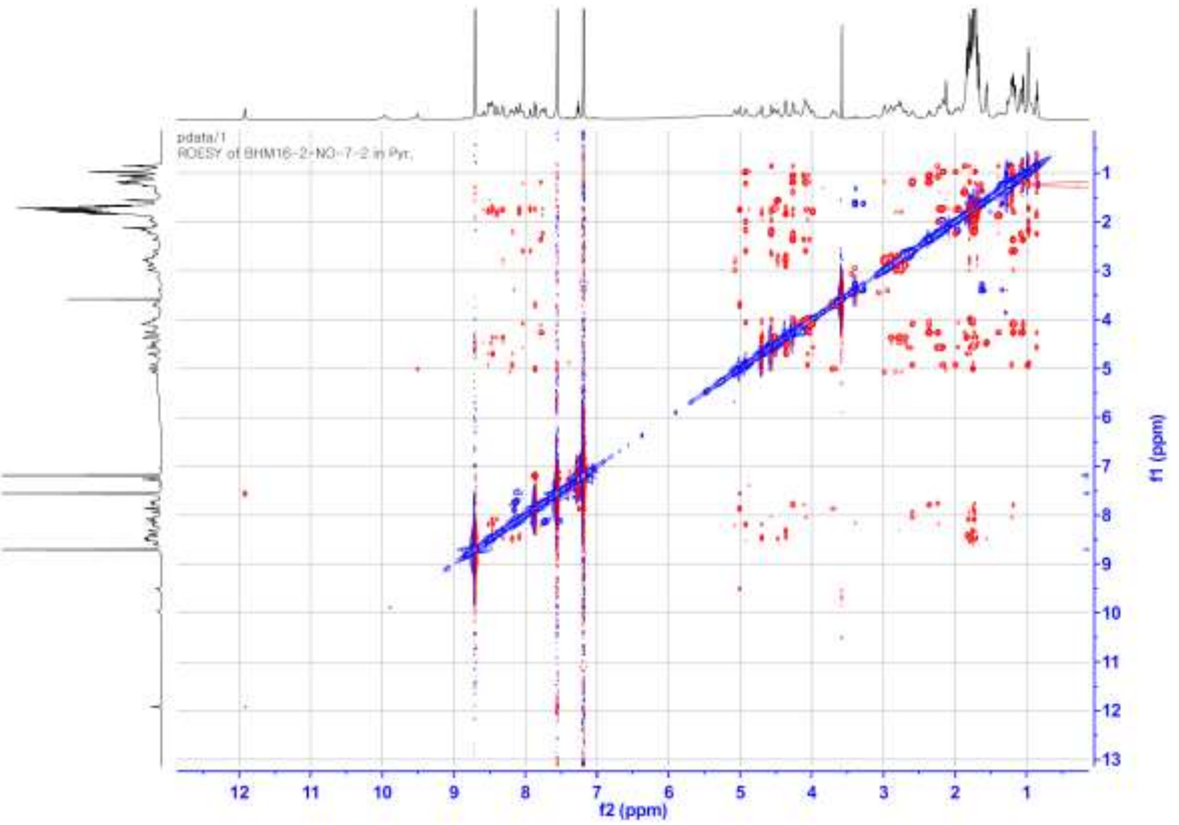

Figure S56. ROESY spectrum of compound **5**

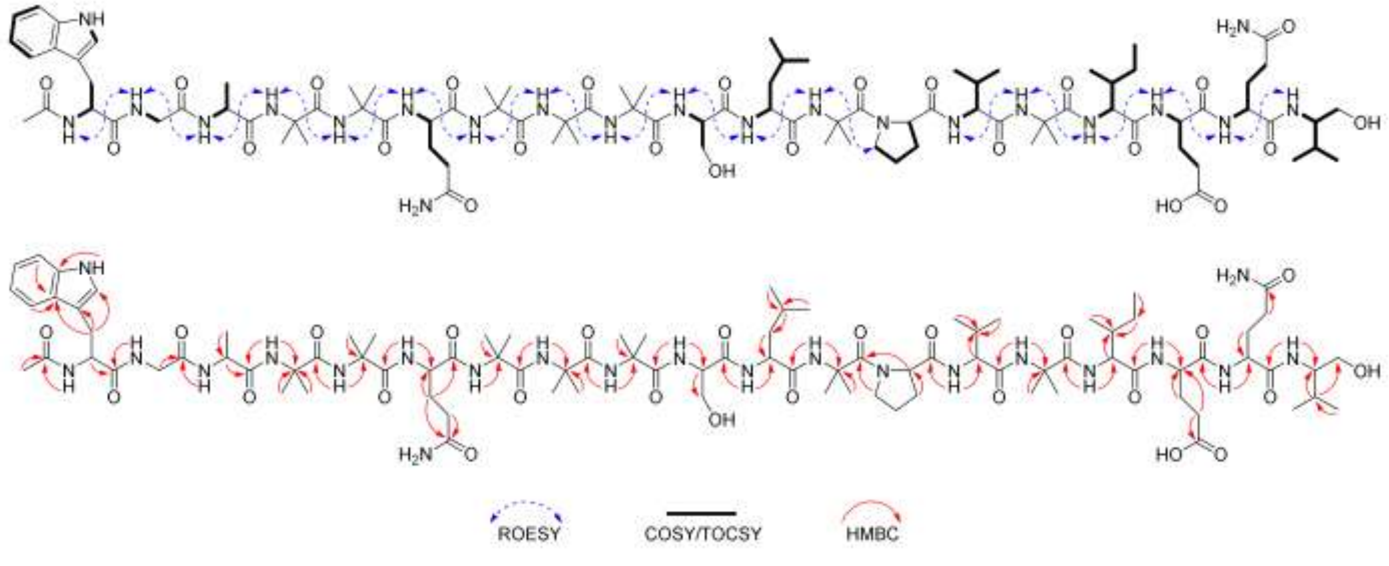

Figure S57. Key ROESY, COSY/TOCSY, and HMBC correlations of compound **5**

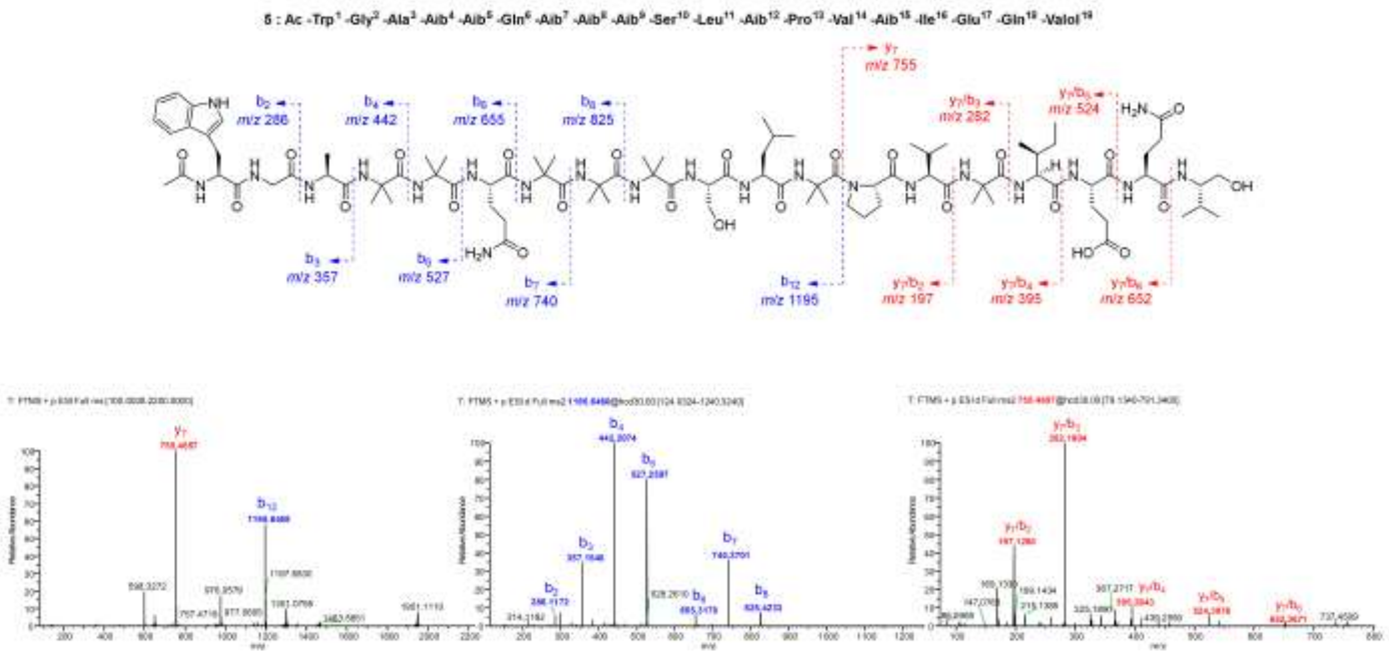

Figure S58. MS fragmentation analysis of compound **5**

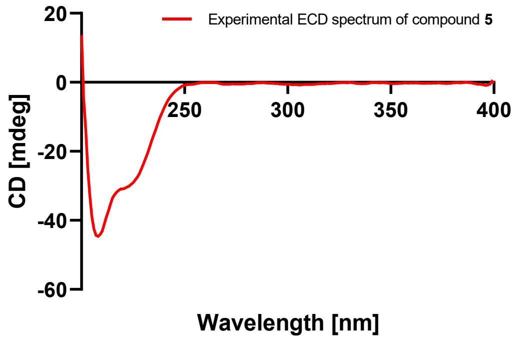

Figure S59. Experimental ECD spectrum of compound **5**

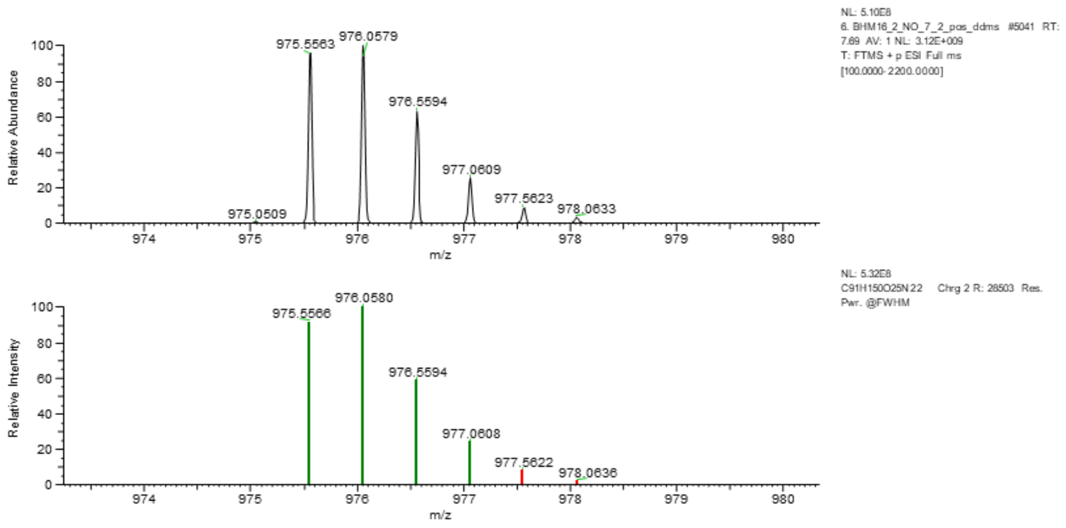

Figure S60. HRESIMS spectrum of compound **5**

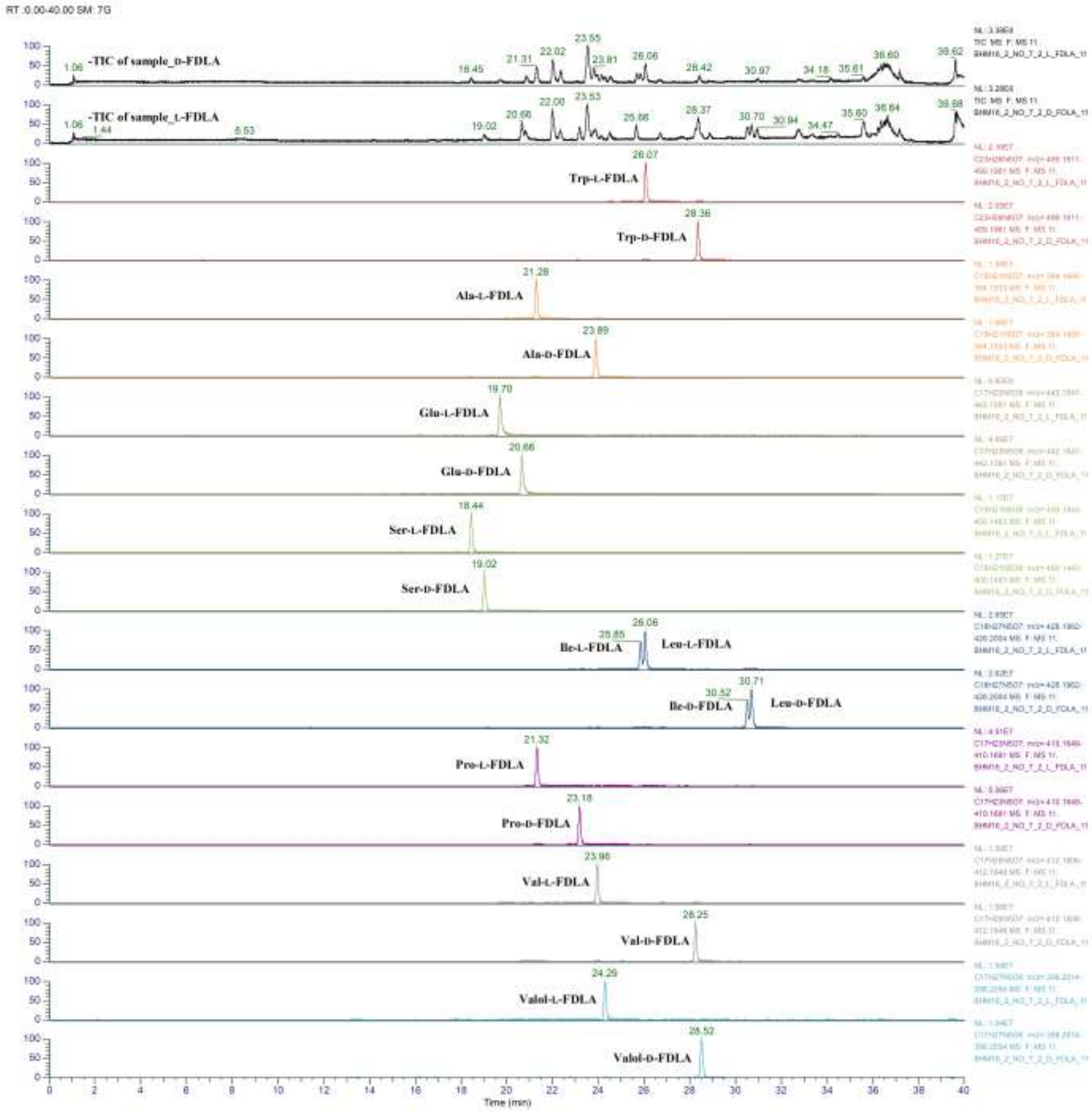

Figure S61. Advanced Marfey's analysis of compound **5**

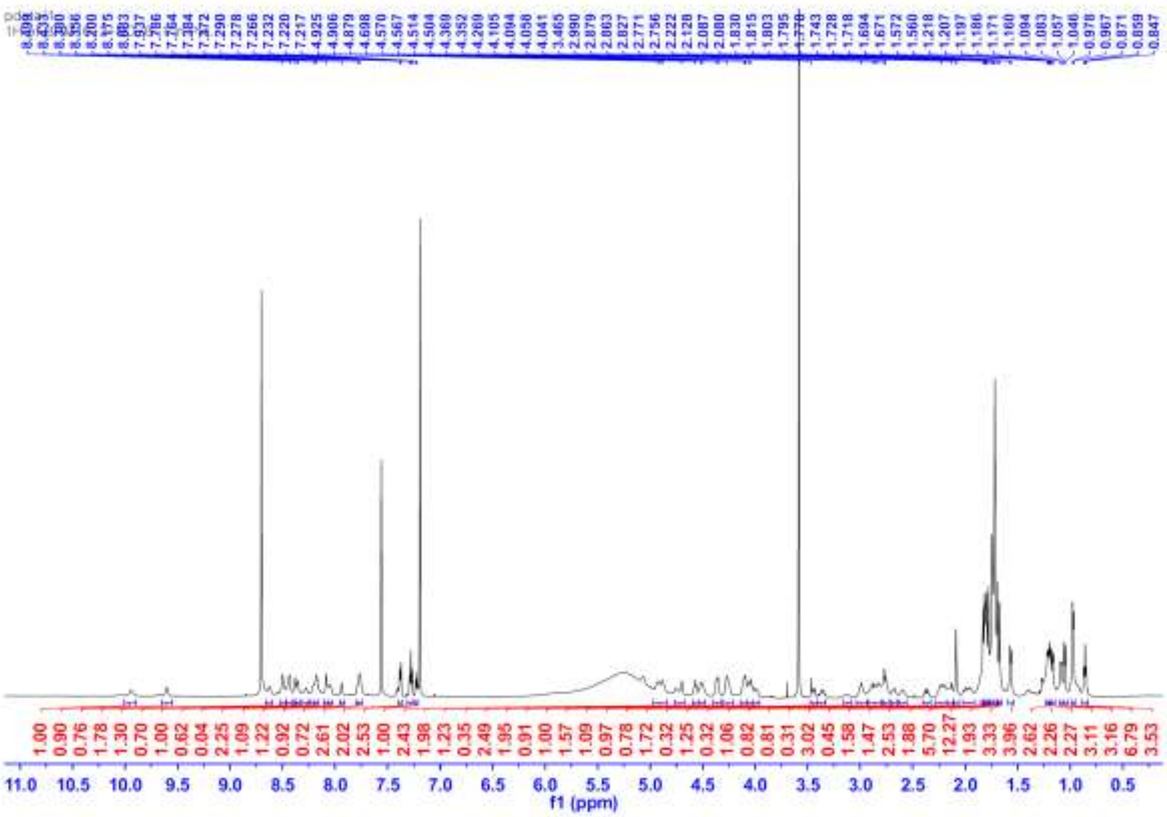

Figure S62. ^{1}H-NMR spectrum of compound **6** (Pyridine-d_5, 600 MHz)

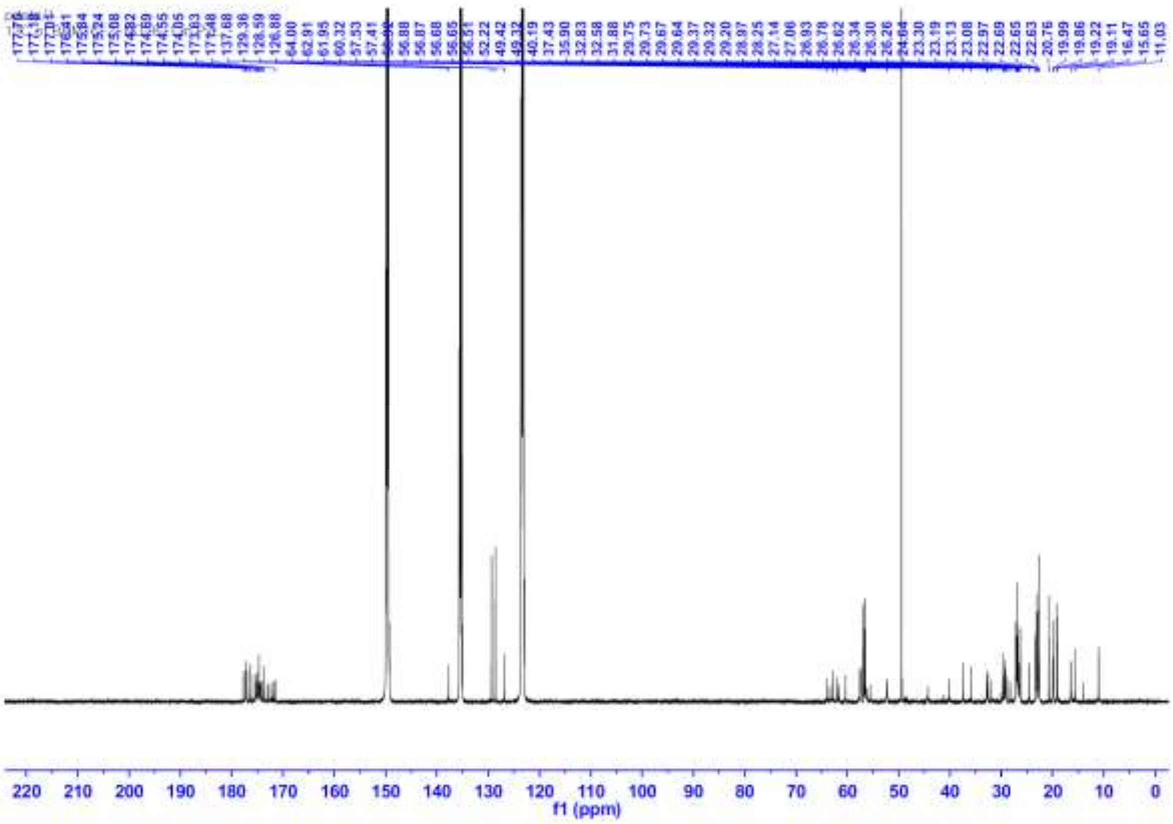

Figure S63. ^{13}C-NMR spectrum of compound **6** (Pyridine-d_5, 150 MHz)

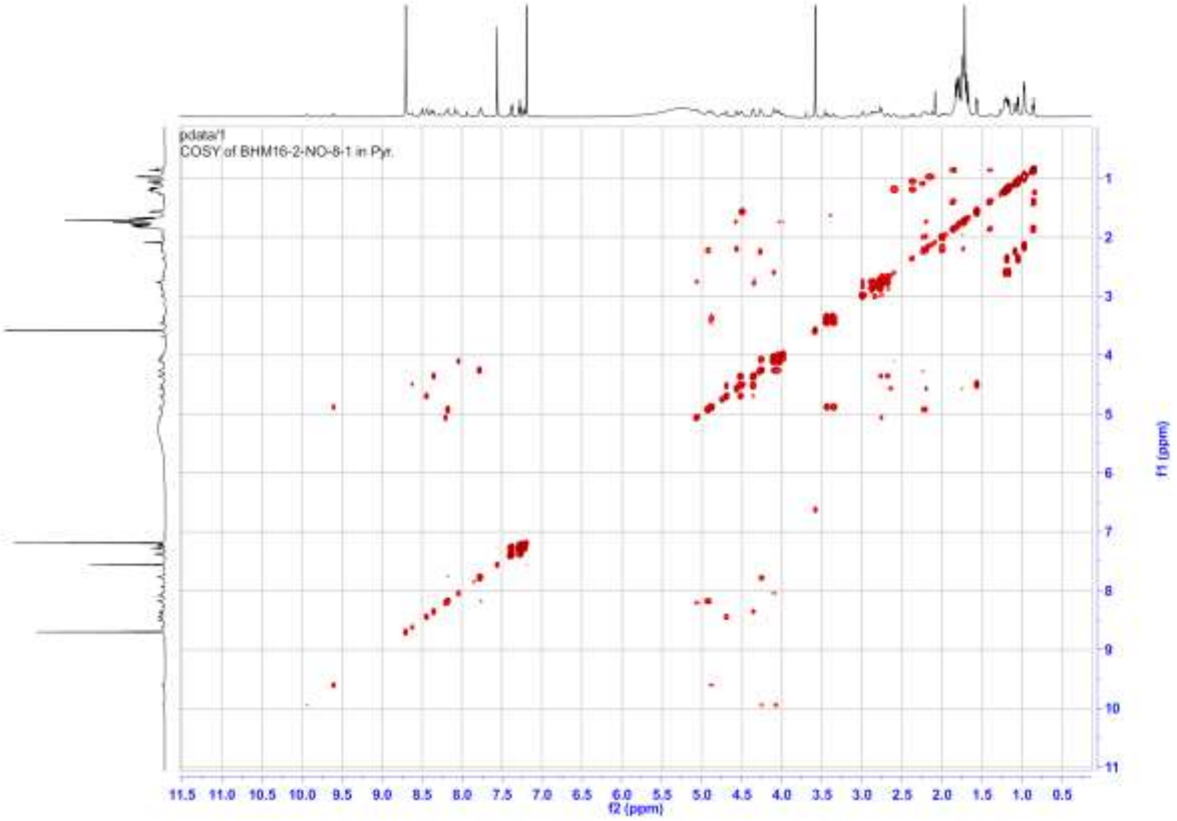

Figure S64. COSY spectrum of compound **6**

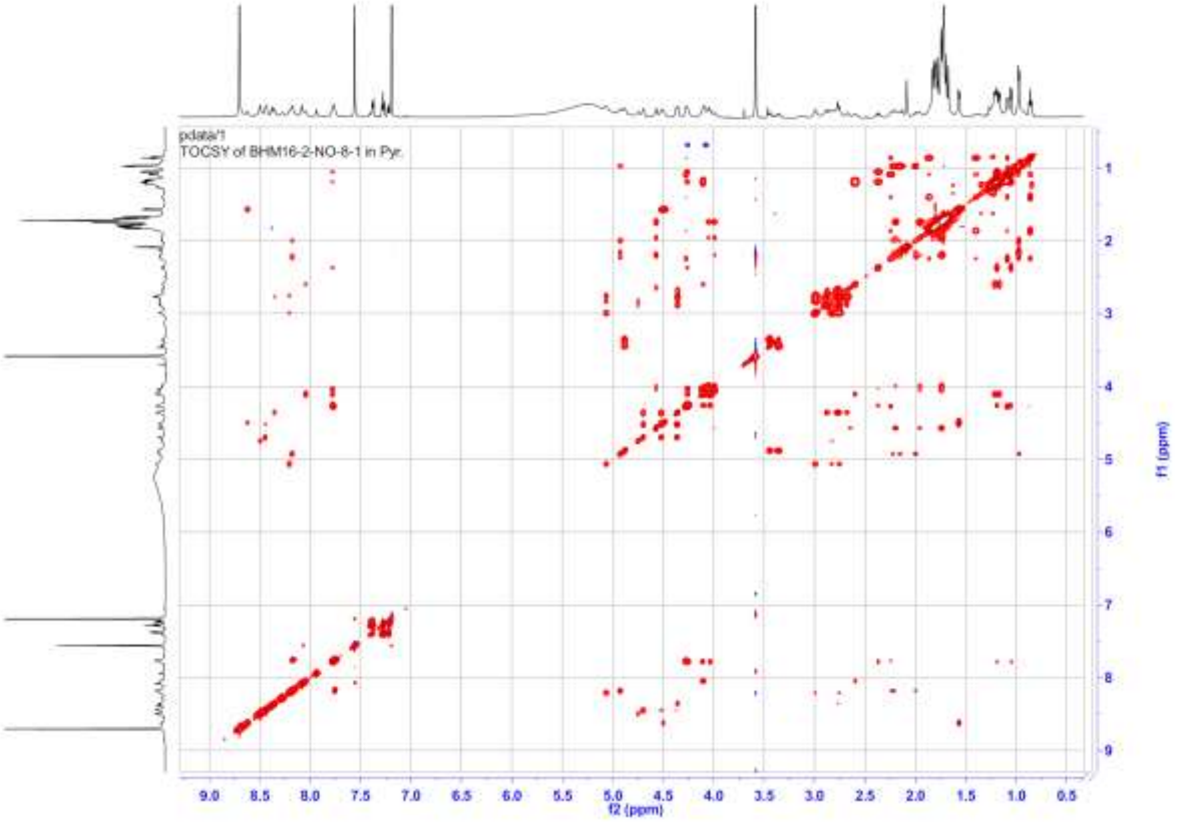

Figure S65. TOCSY spectrum of compound **6**

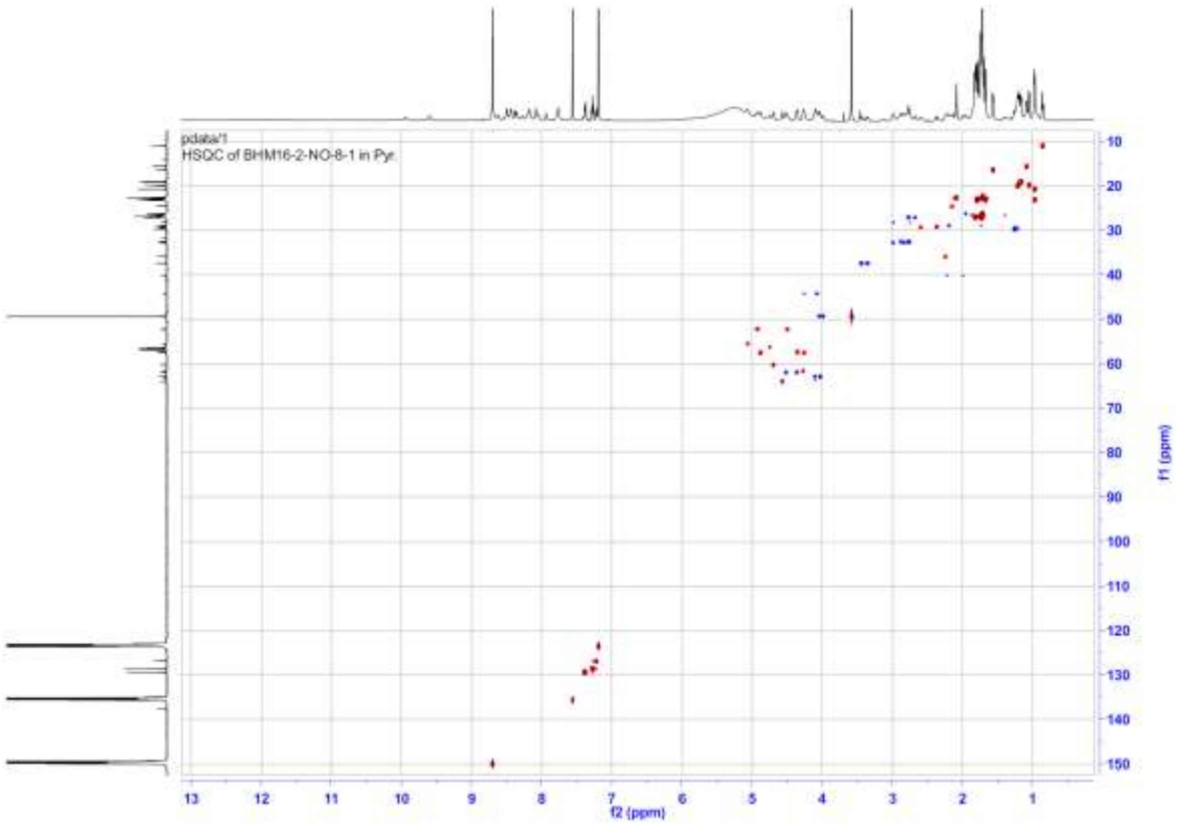

Figure S66. HSQC-DEPT spectrum of compound **6**

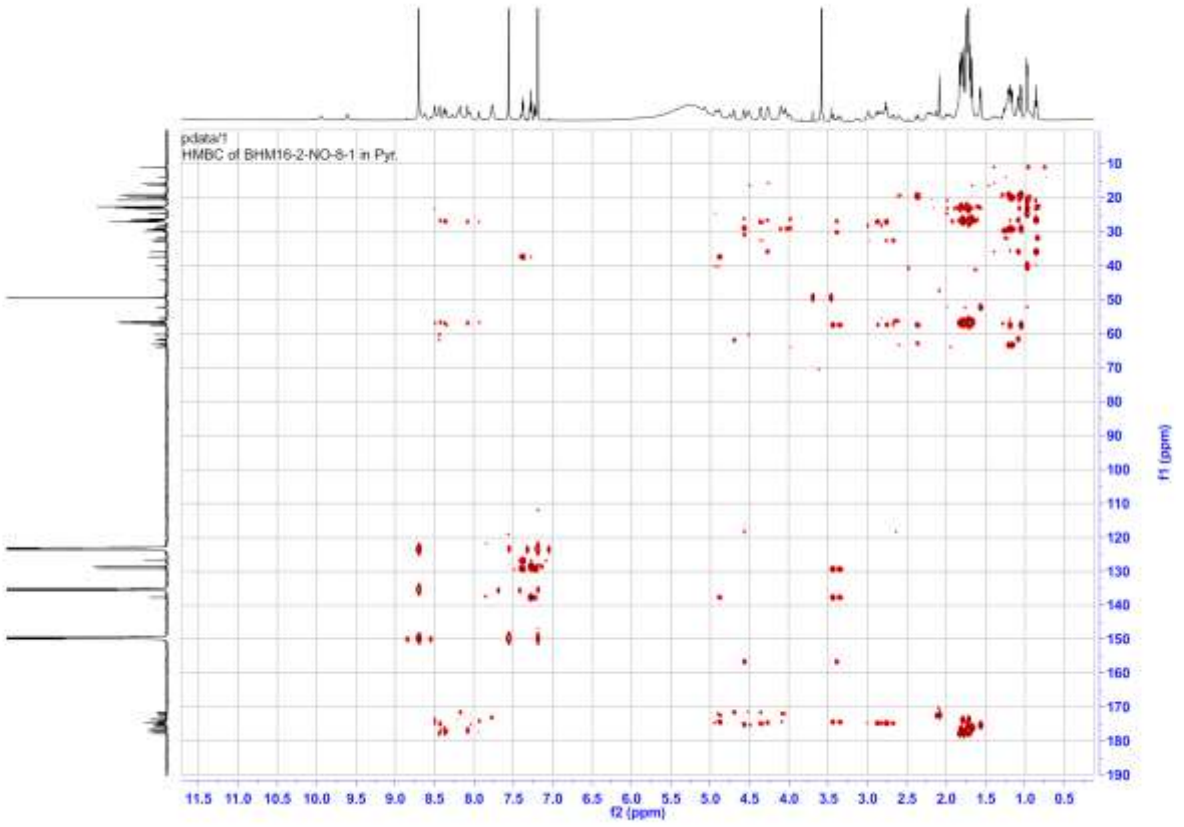

Figure S67. HMBC spectrum of compound **6**

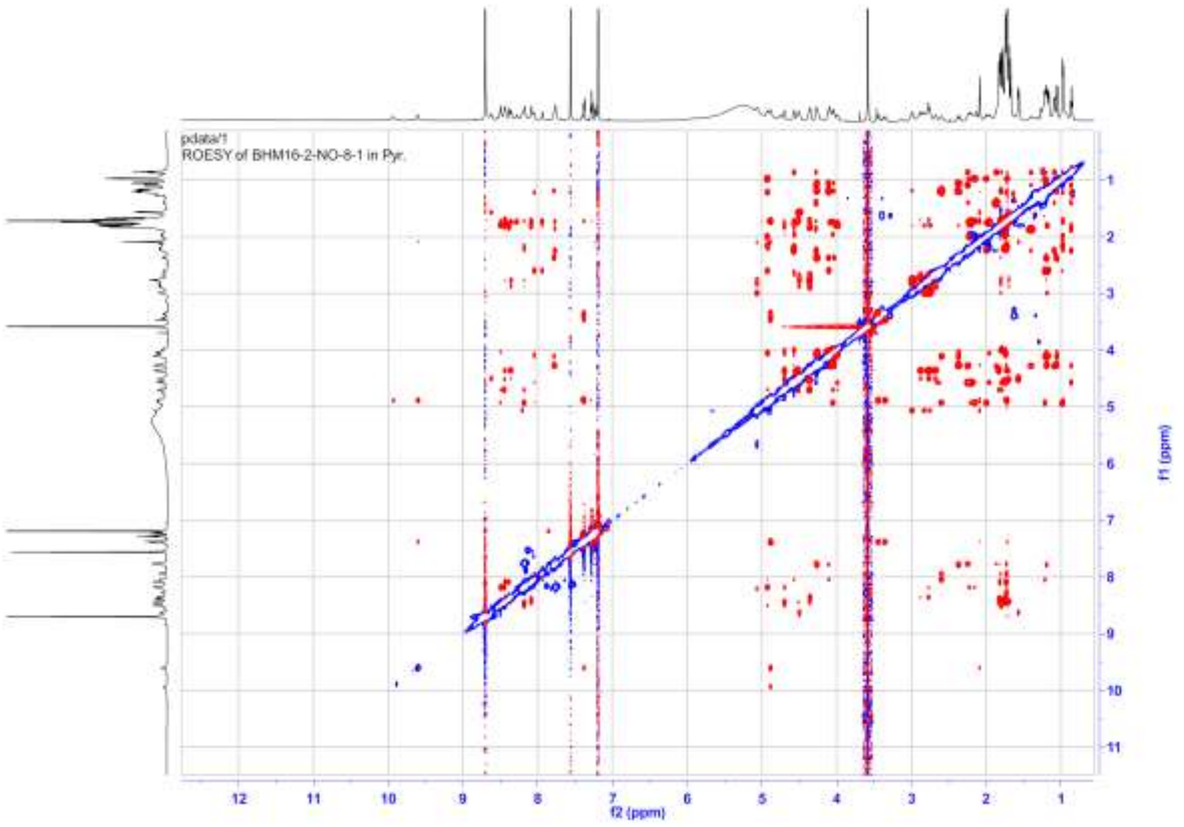

Figure S68. ROESY spectrum of compound **6**

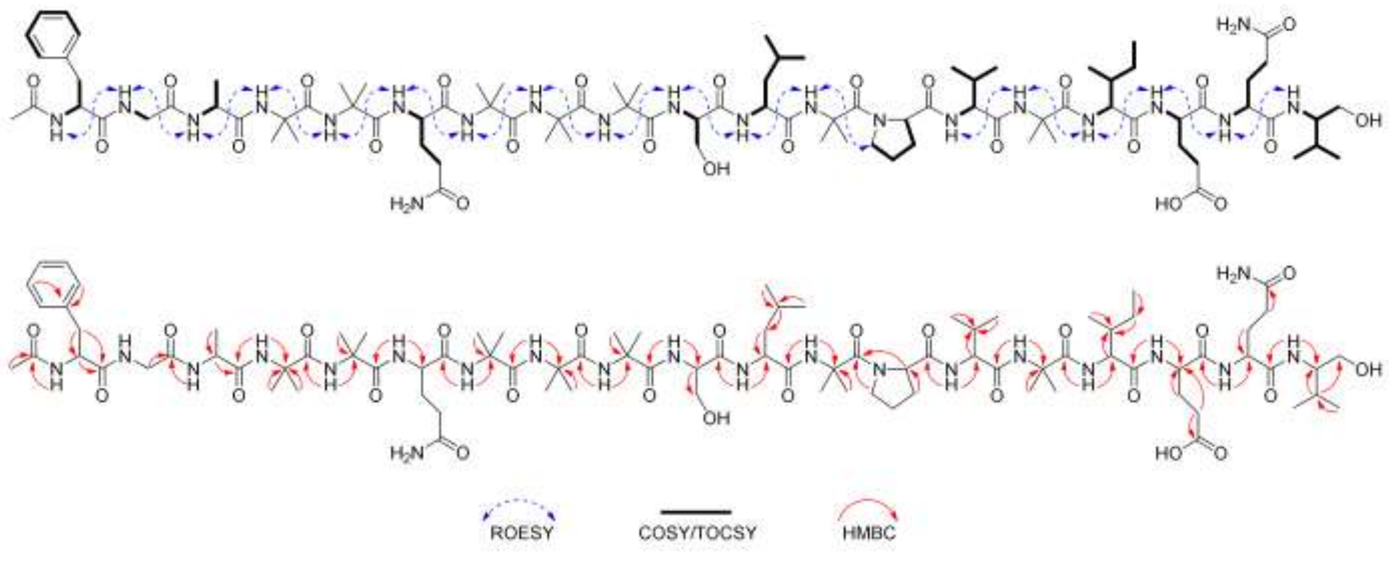

Figure S69. Key ROESY, COSY/TOCSY, and HMBC correlations of compound **6**

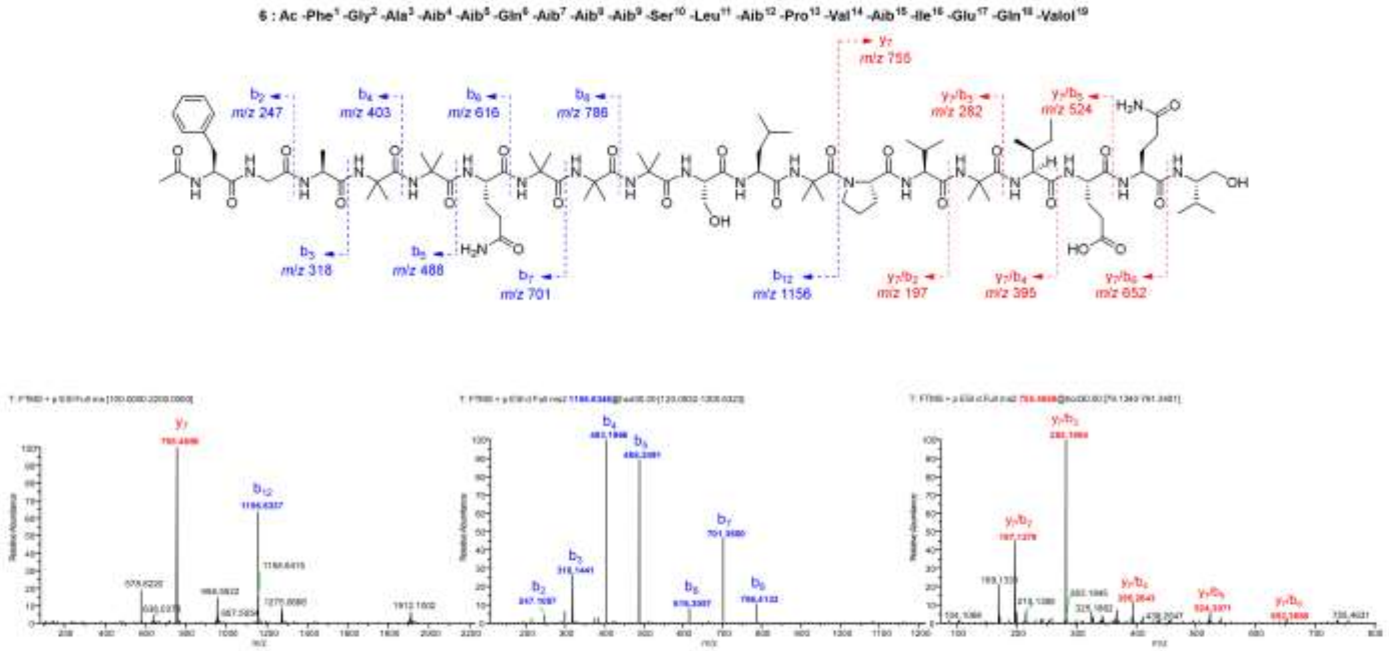

Figure S70. MS fragmentation analysis of compound **6**

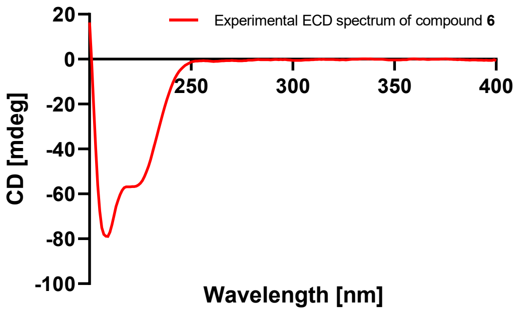

Figure S71. Experimental ECD spectrum of compound **6**

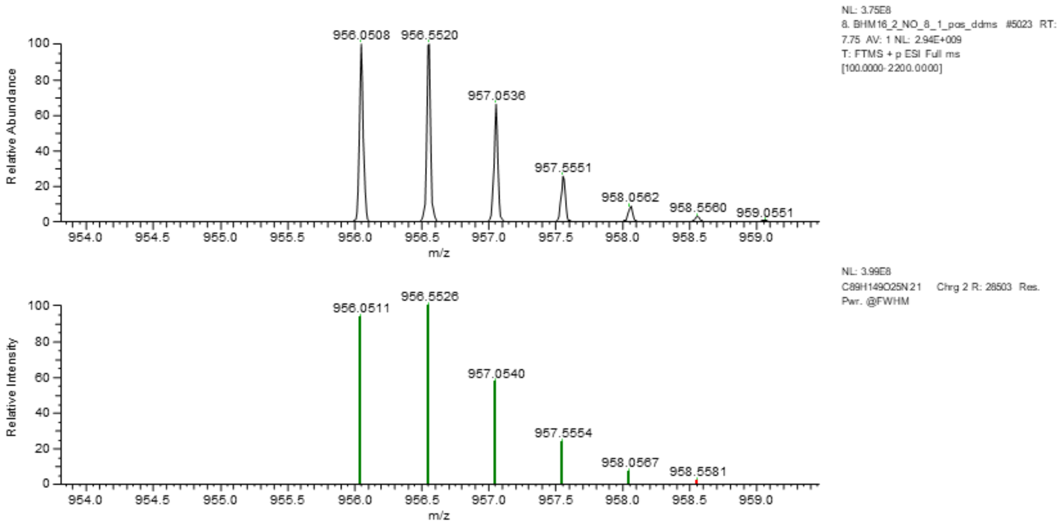

Figure S72. HRESIMS spectrum of compound **6**

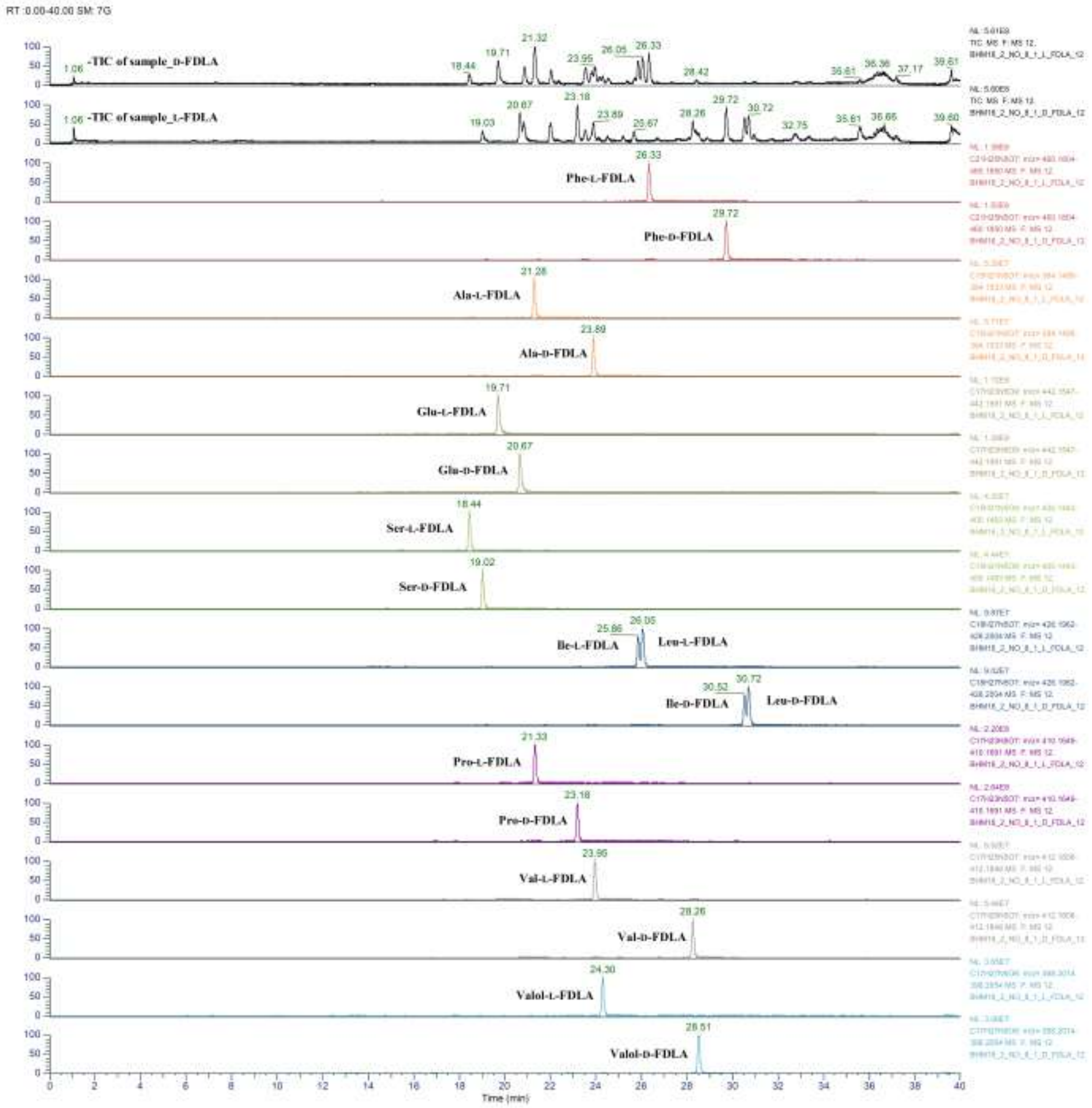

Figure S73. Advanced Marfey's analysis of compound **6**

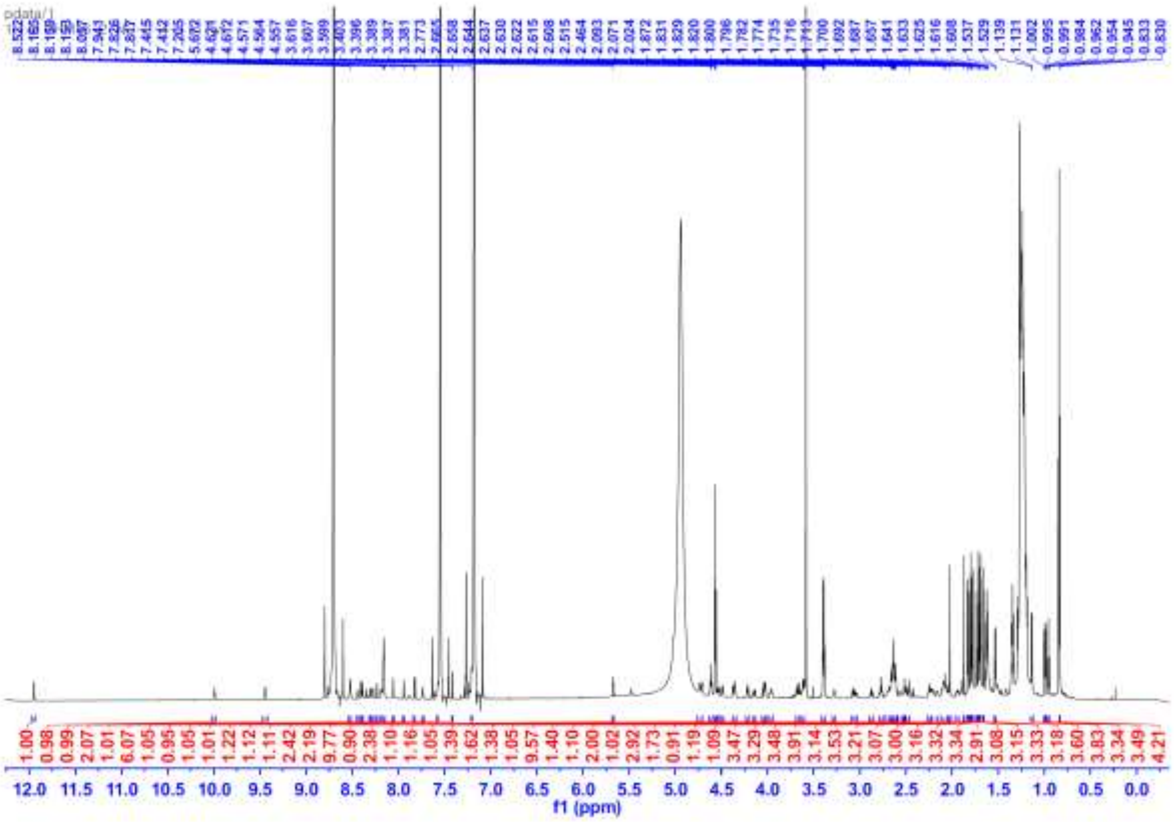

Figure S74. ^{1}H NMR spectrum of compound **7** (Pyridine-d_5, 900 MHz)

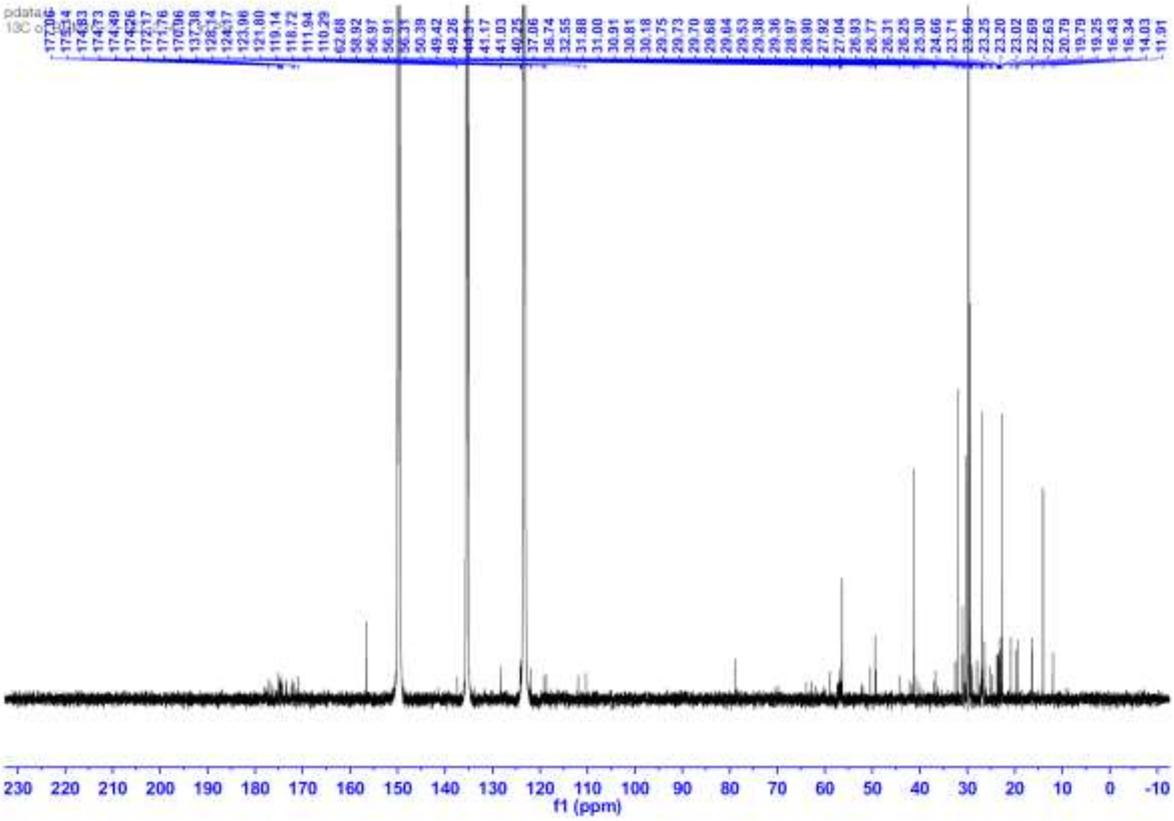

Figure S75. ^{13}C NMR spectrum of compound **7** (Pyridine-d_5, 225 MHz)

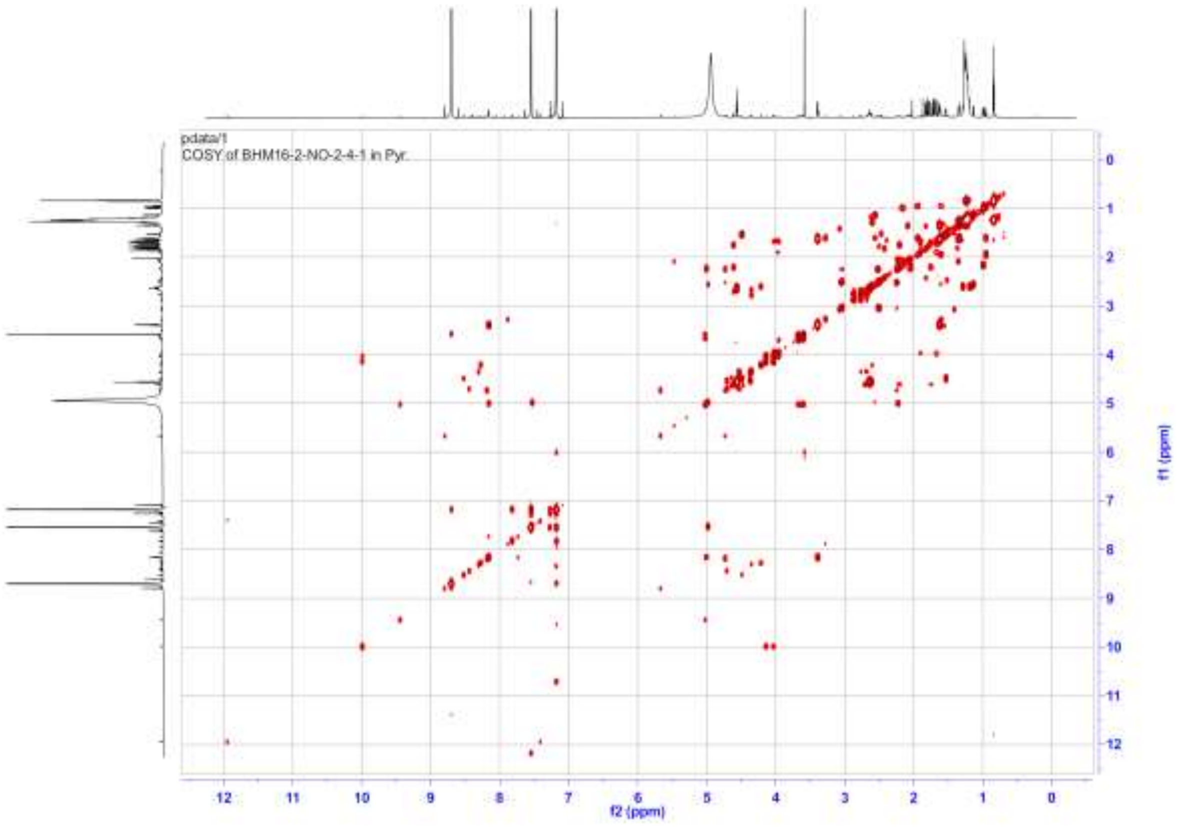

Figure S76. COSY spectrum of compound **7**

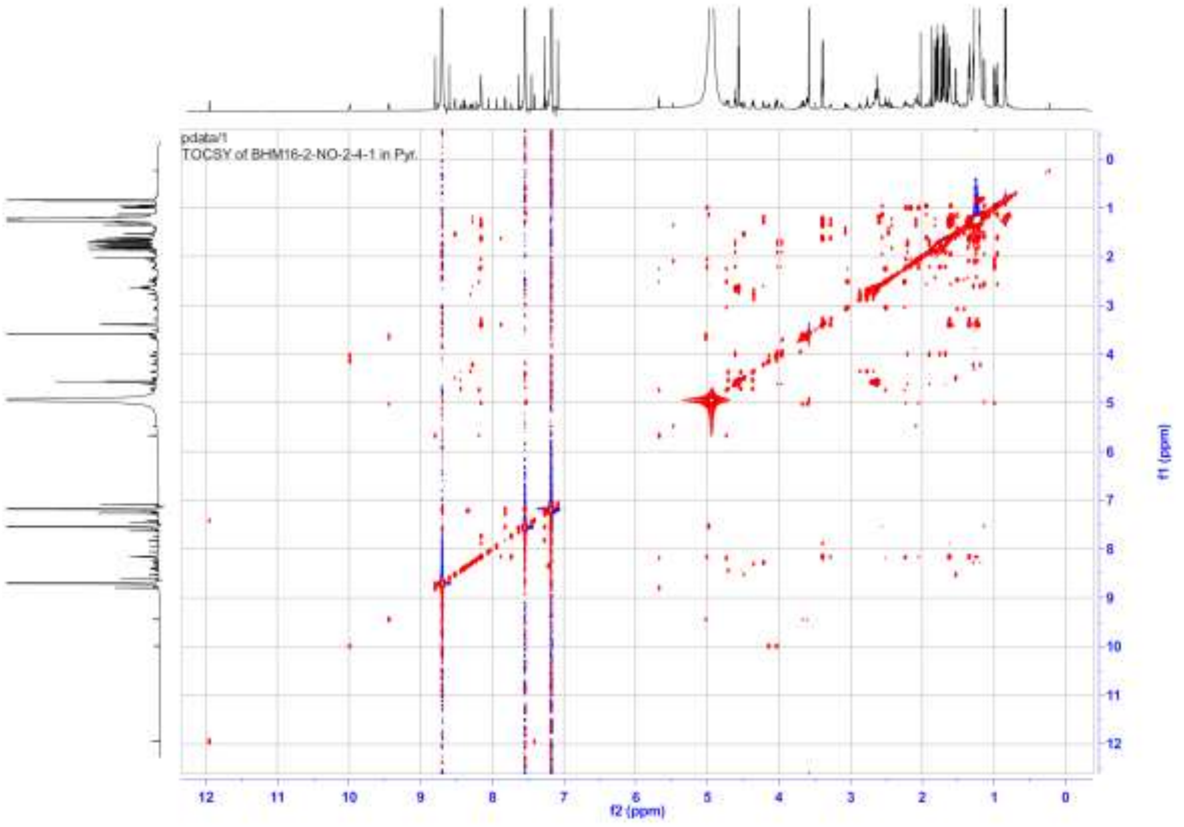

Figure S77. TOCSY spectrum of compound **7**

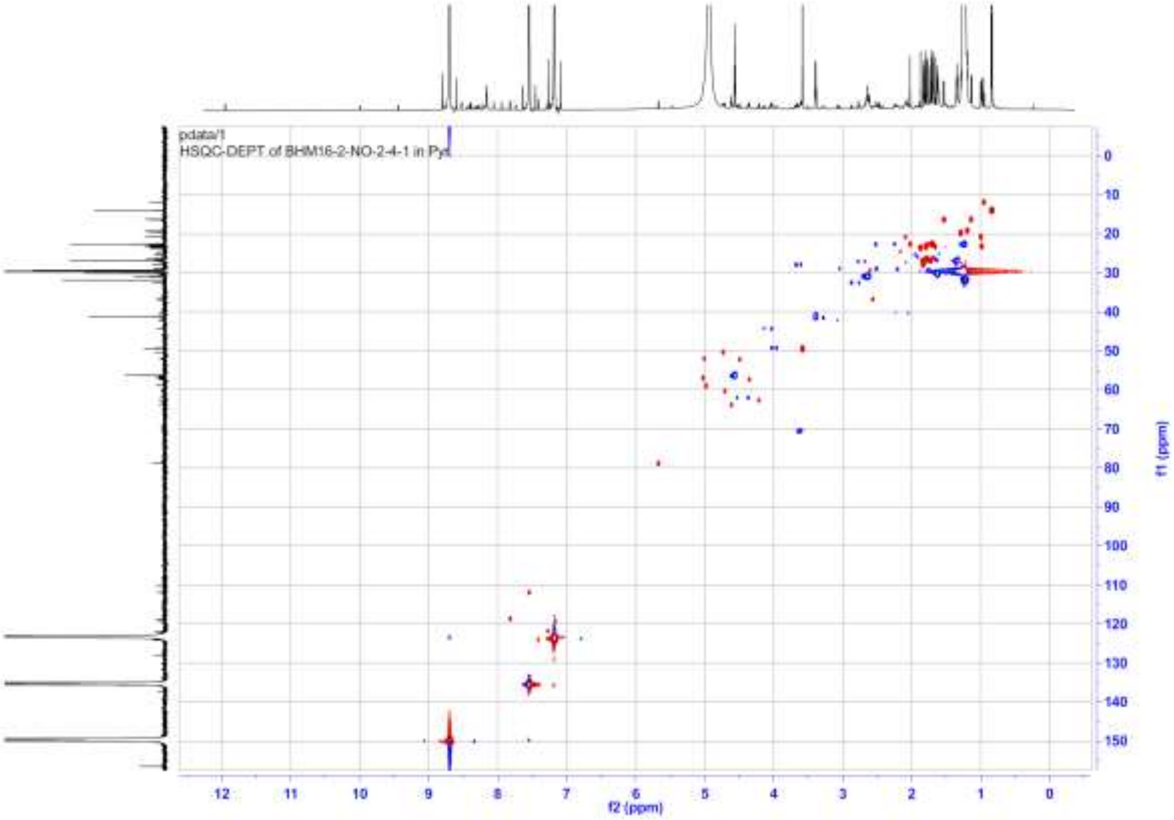

Figure S78. HSQC-DEPT spectrum of compound **7**

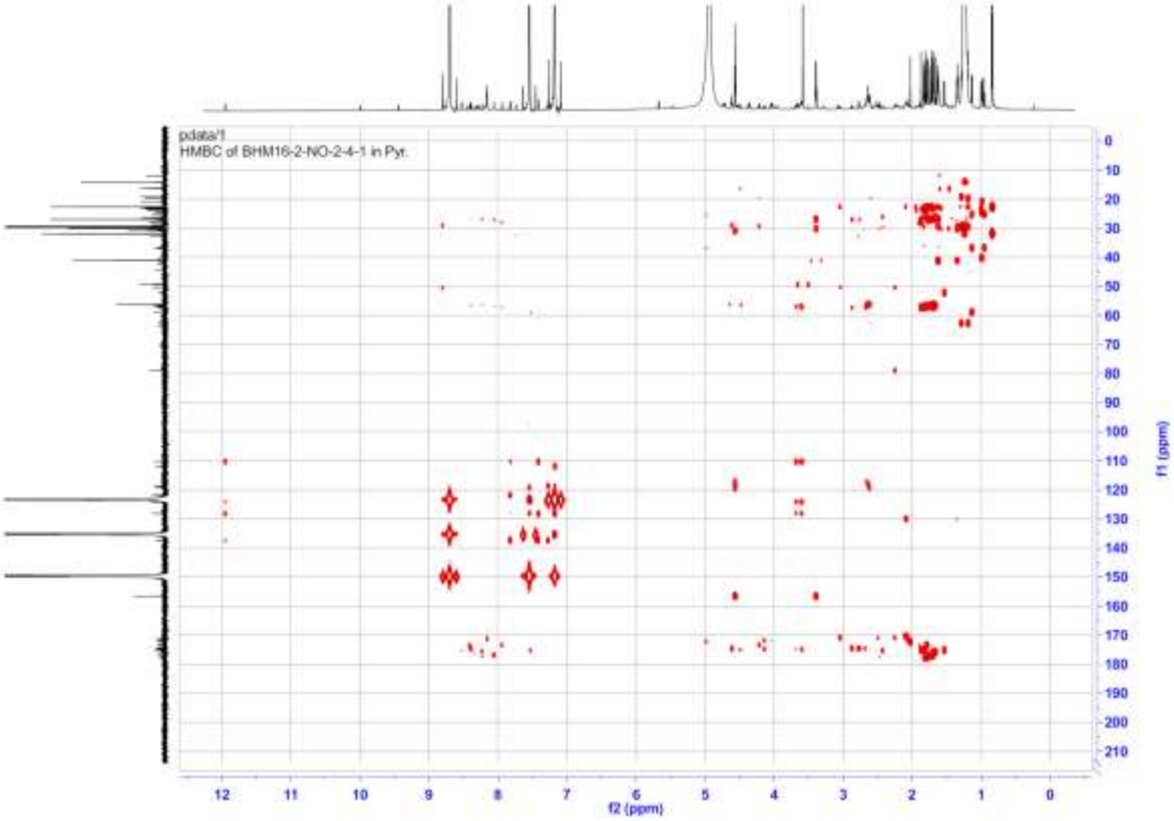

Figure S79. HMBC spectrum of compound **7**

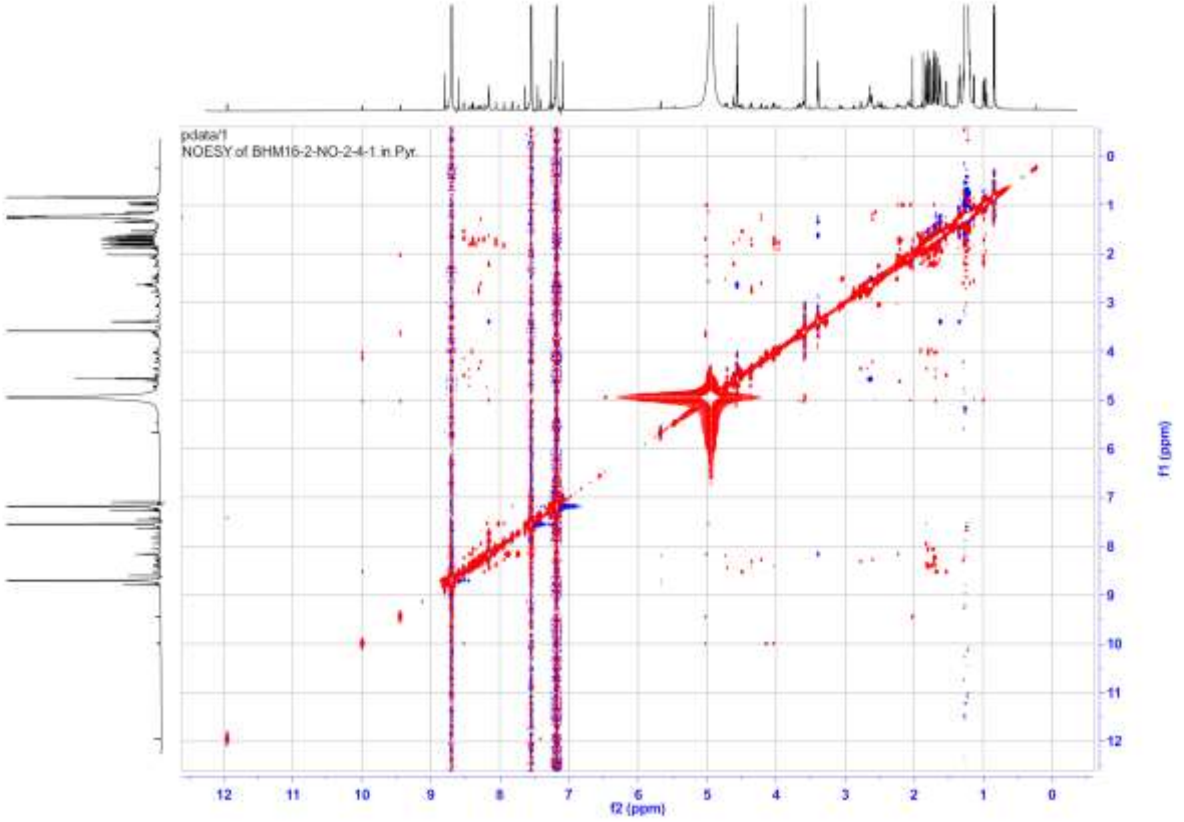

Figure S80. NOESY spectrum of compound **7**

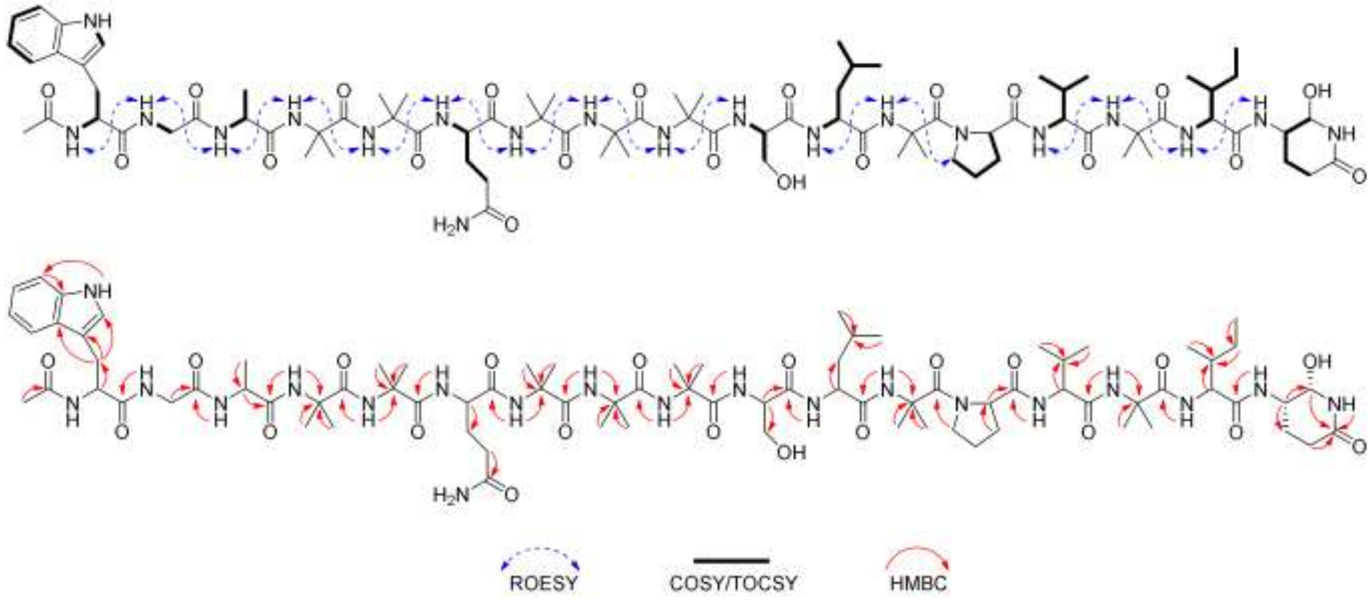

Figure S81. Key ROESY, COSY/TOCSY, and HMBC correlations of compound **7**

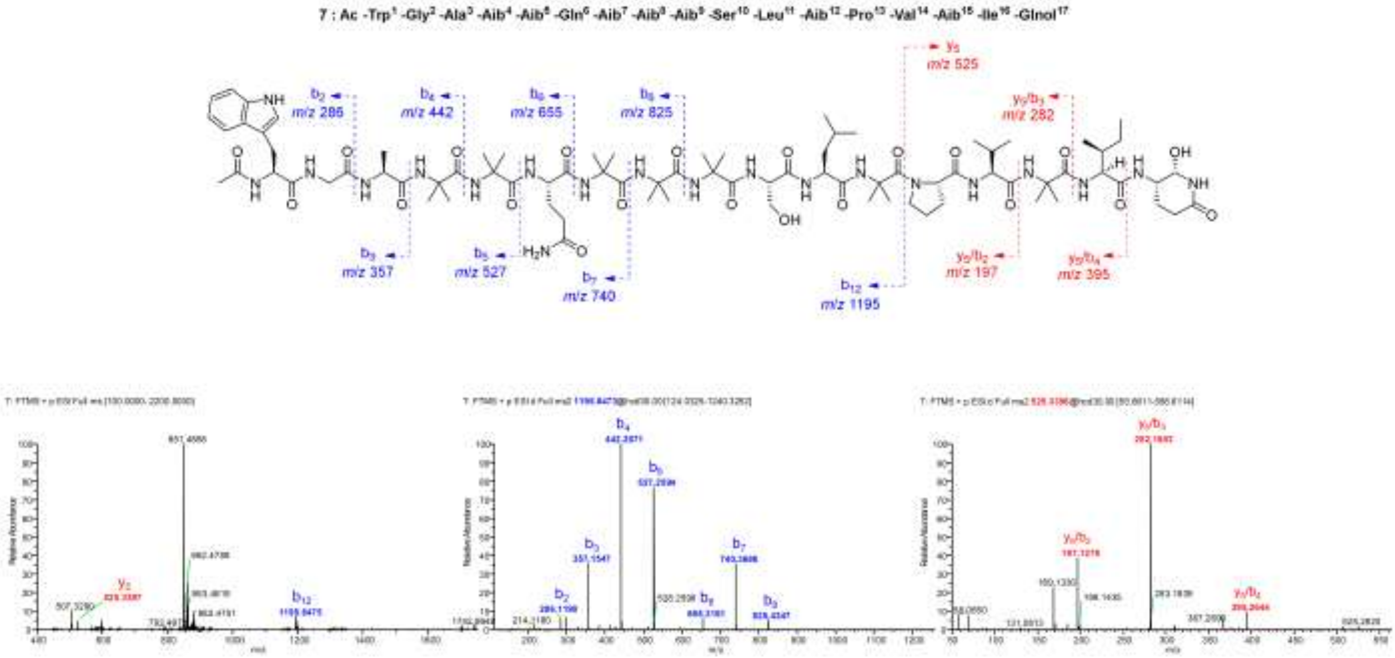

Figure S82. MS fragmentation analysis of compound 7

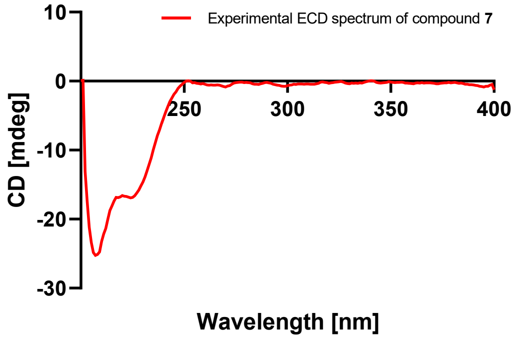

Figure S83. Experimental ECD spectrum of compound 7

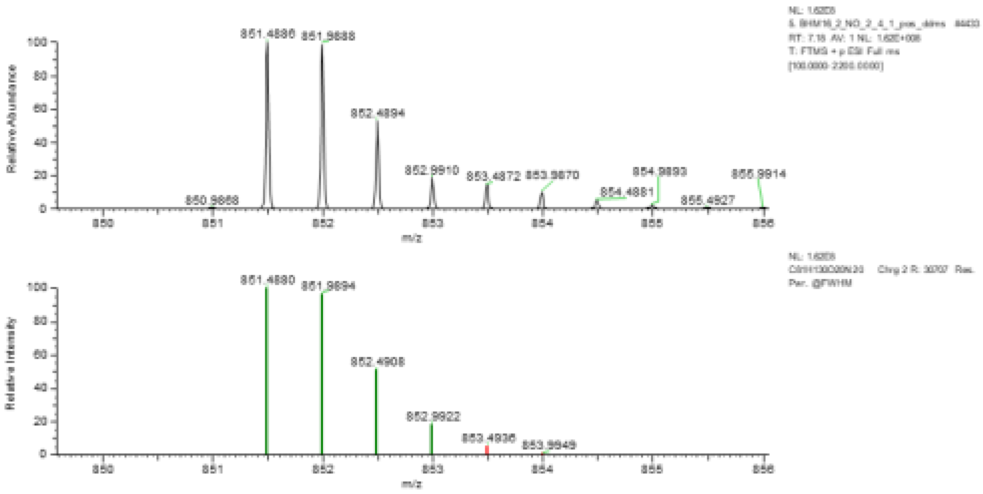

Figure S84. HRESIMS spectrum of compound 7

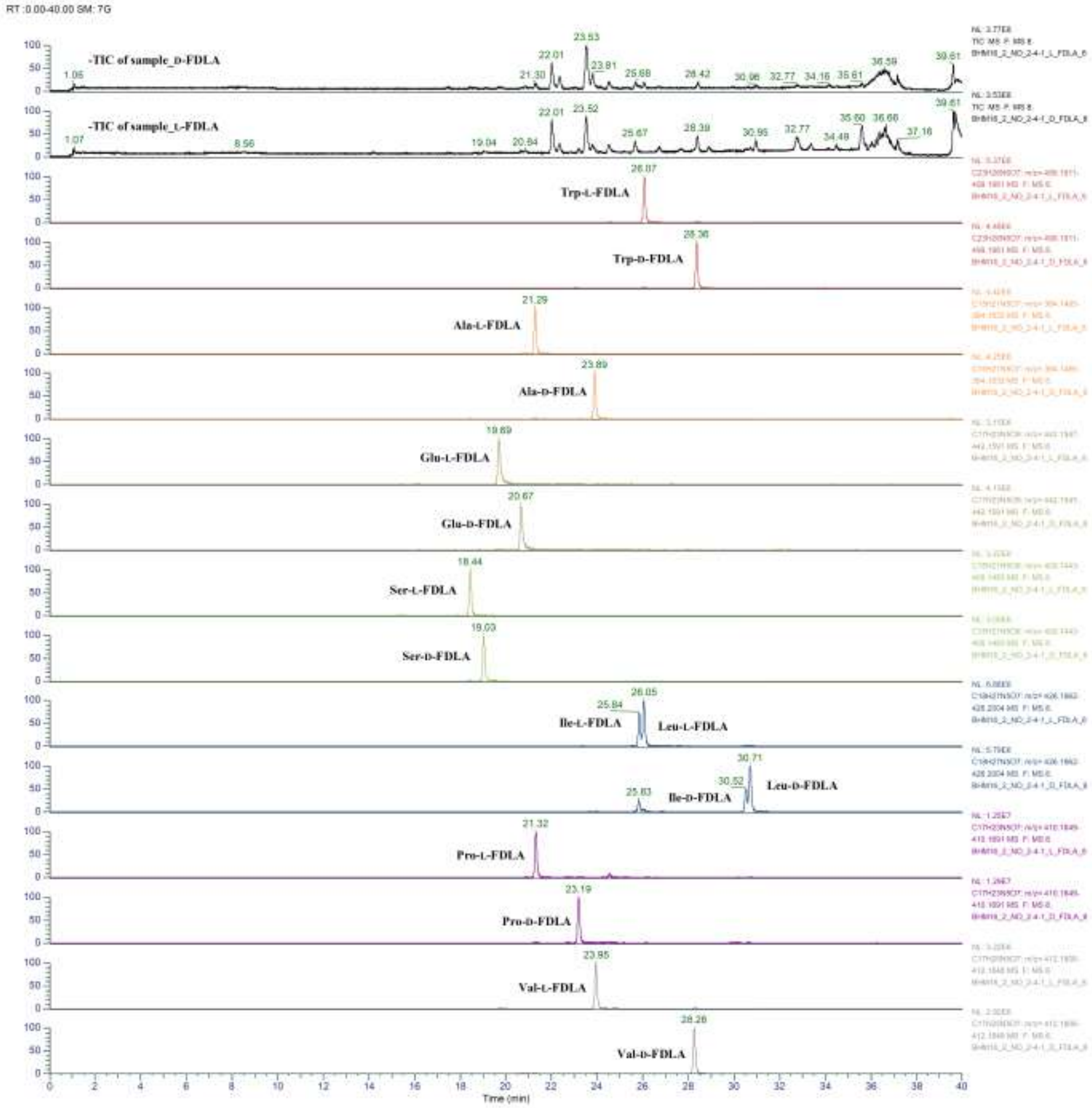

Figure S85. Advanced Marfey's analysis of compound **7**

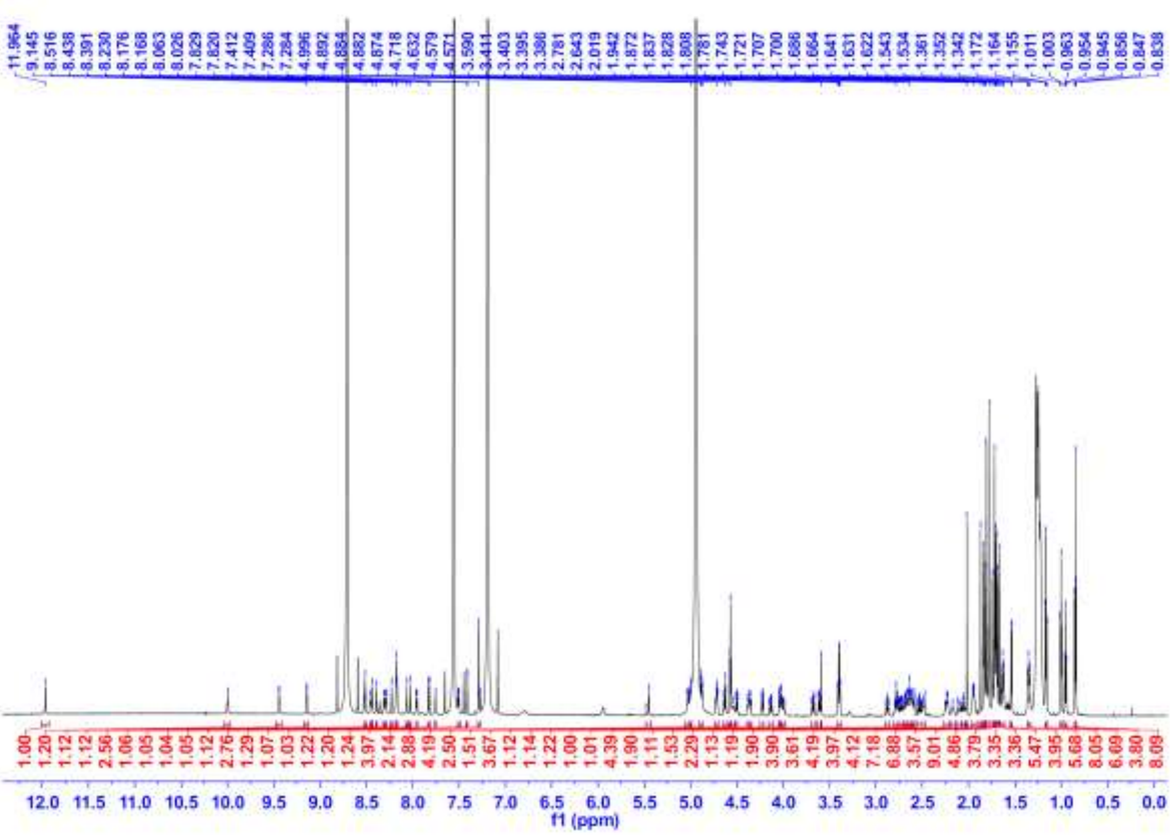

Figure S86. ^{1}H-NMR spectrum of compound **8** (Pyridine-d_5, 800 MHz)

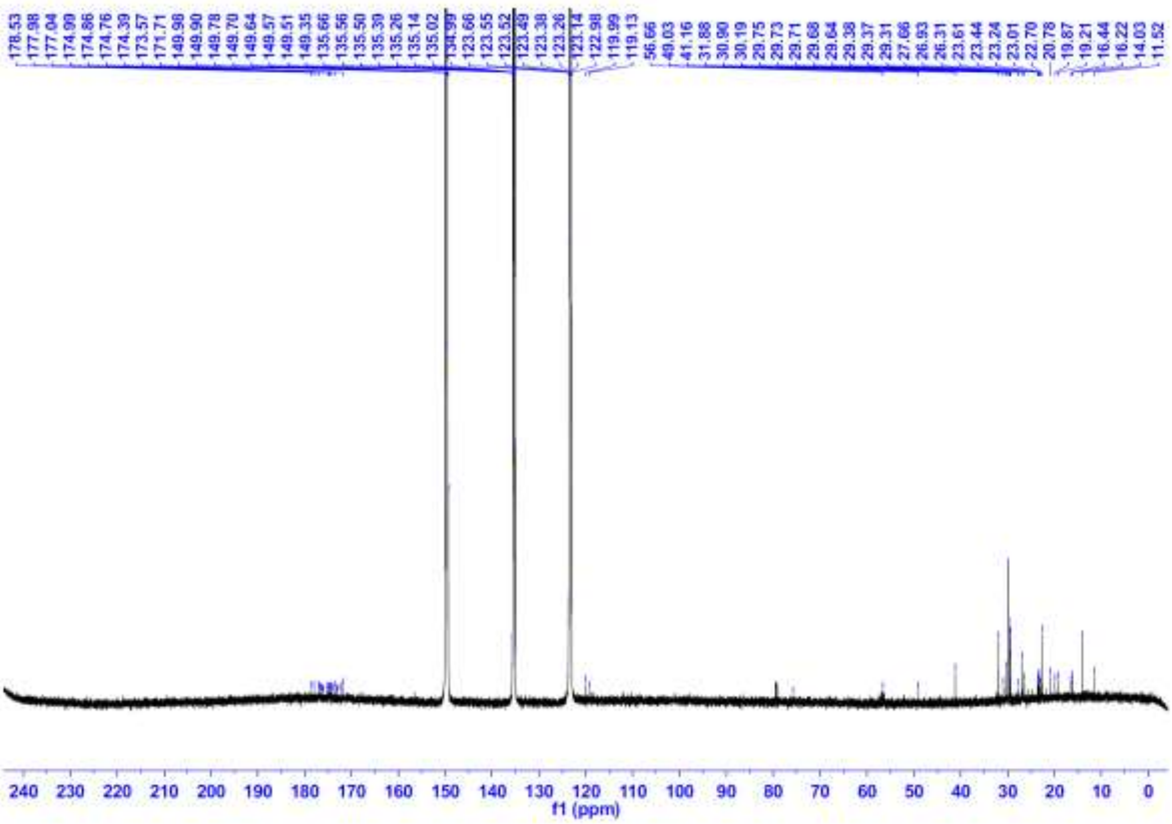

Figure S87. ^{13}C-NMR spectrum of compound **8** (Pyridine-d_5, 200 MHz)

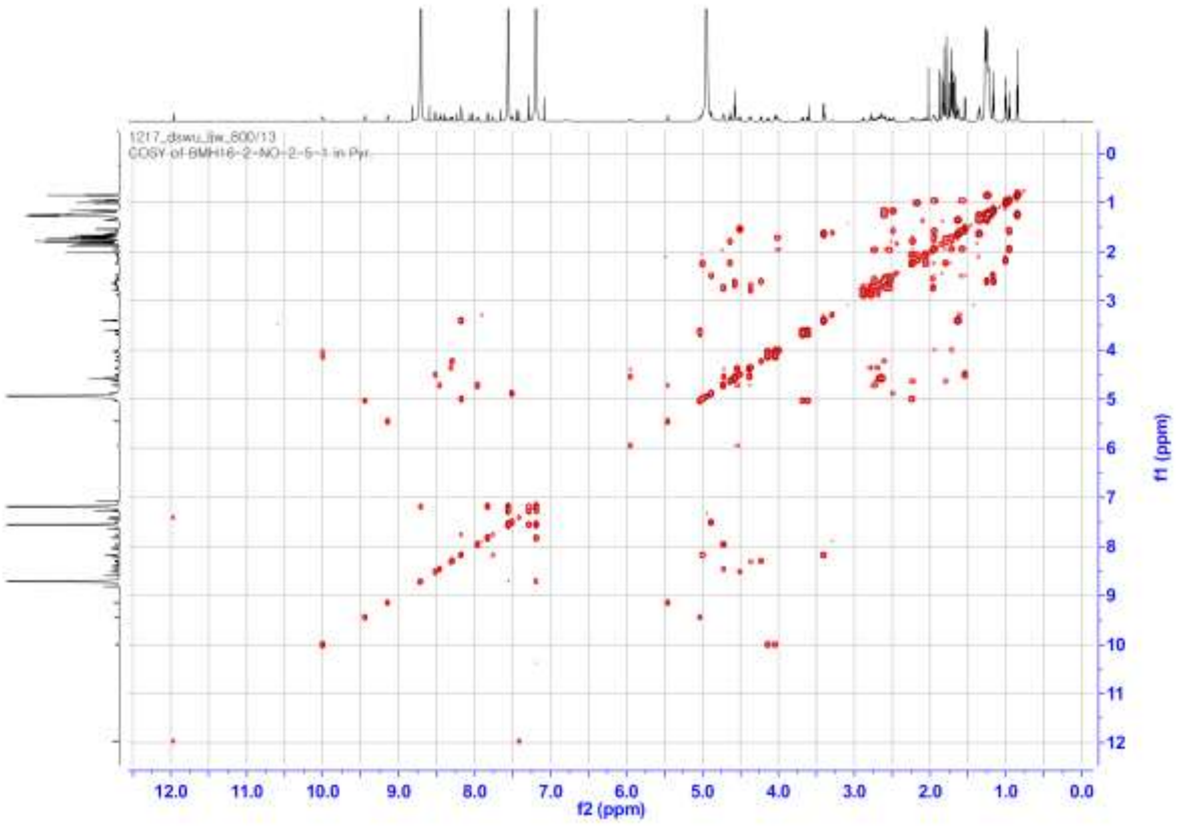

Figure S88. COSY spectrum of compound **8**

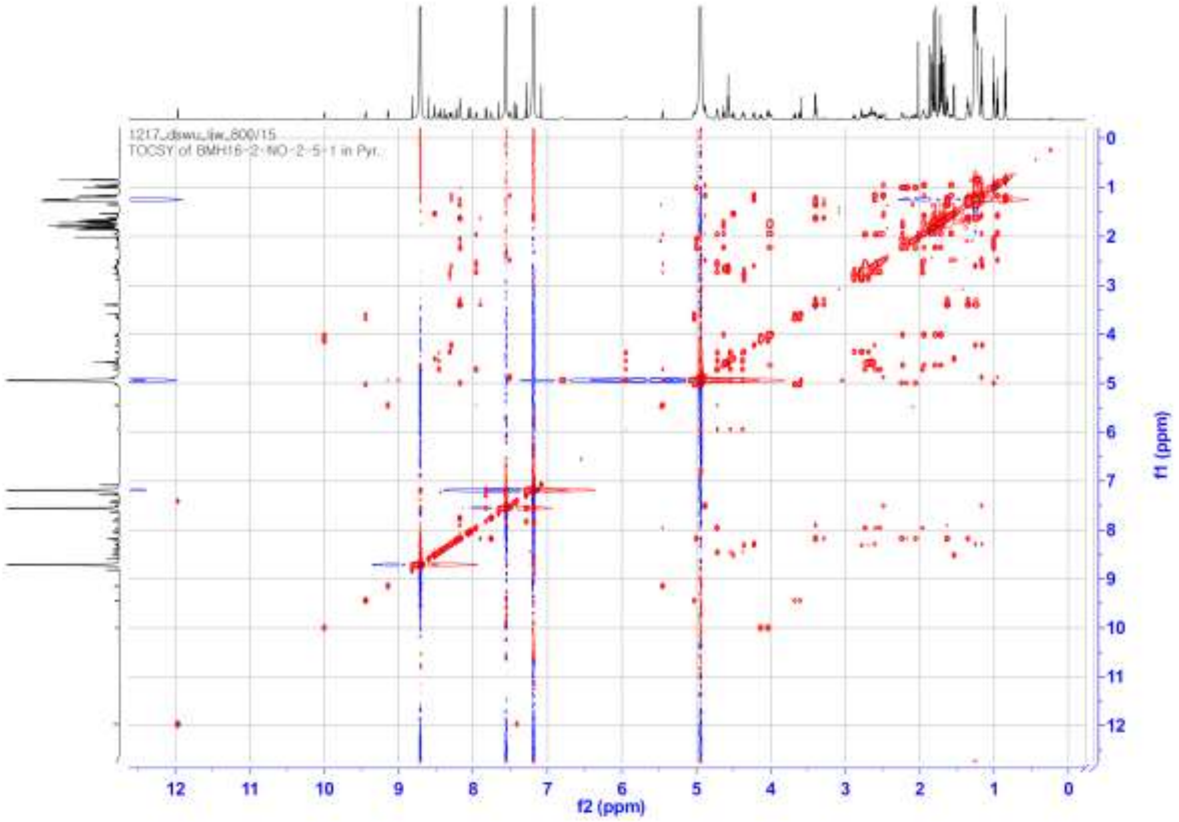

Figure S89. TOCSY spectrum of compound **8**

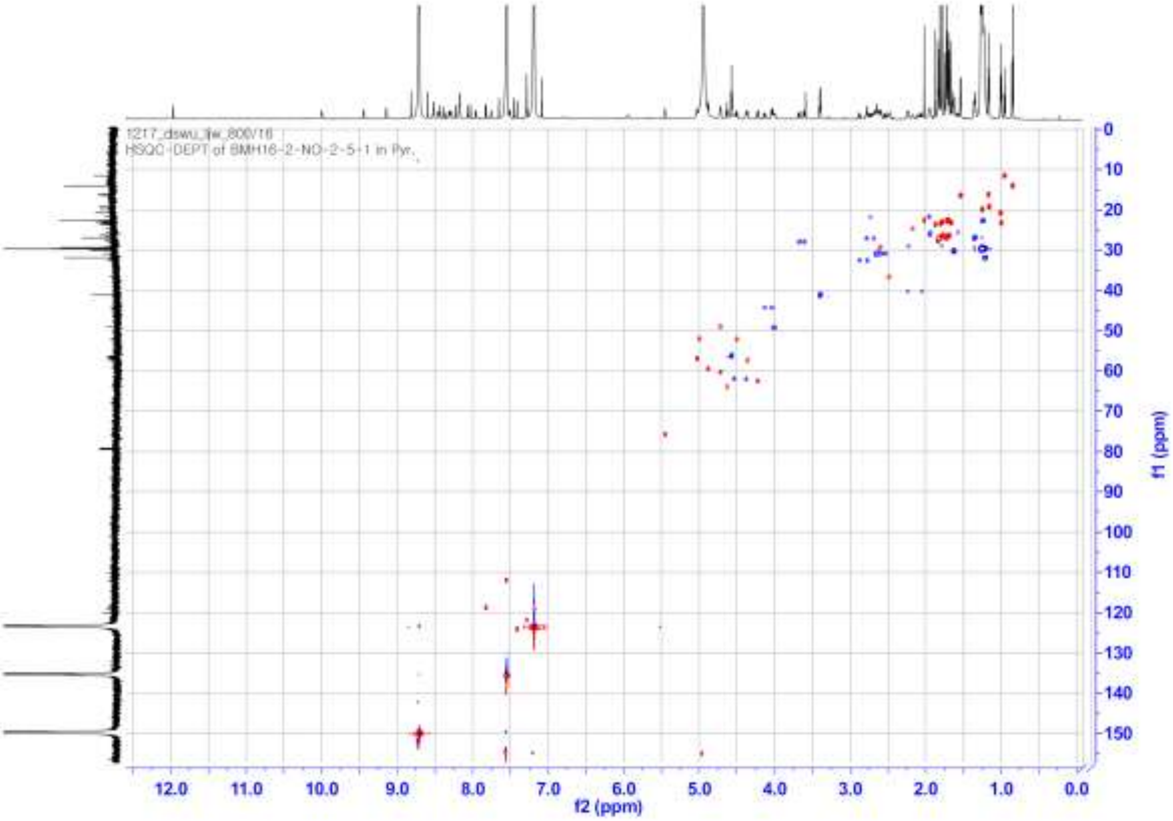

Figure S90. HSQC-DEPT spectrum of compound **8**

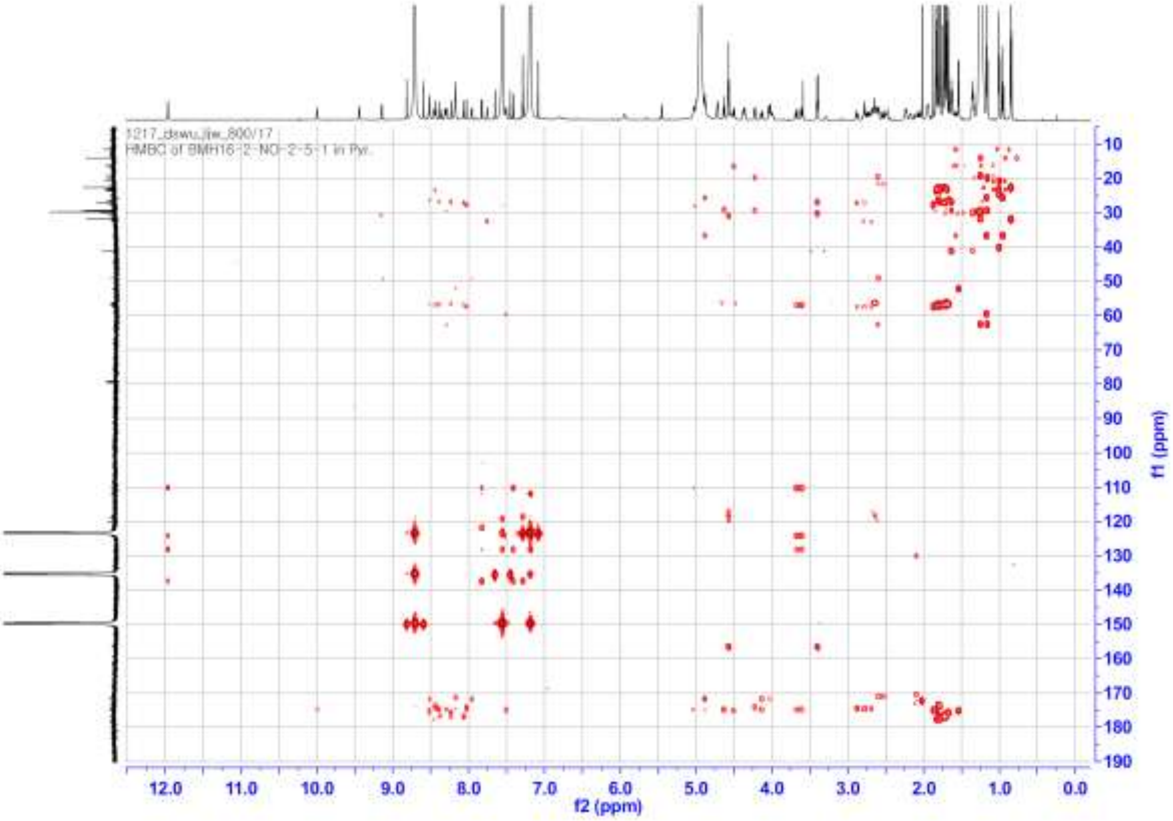

Figure S91. HMBC spectrum of compound **8**

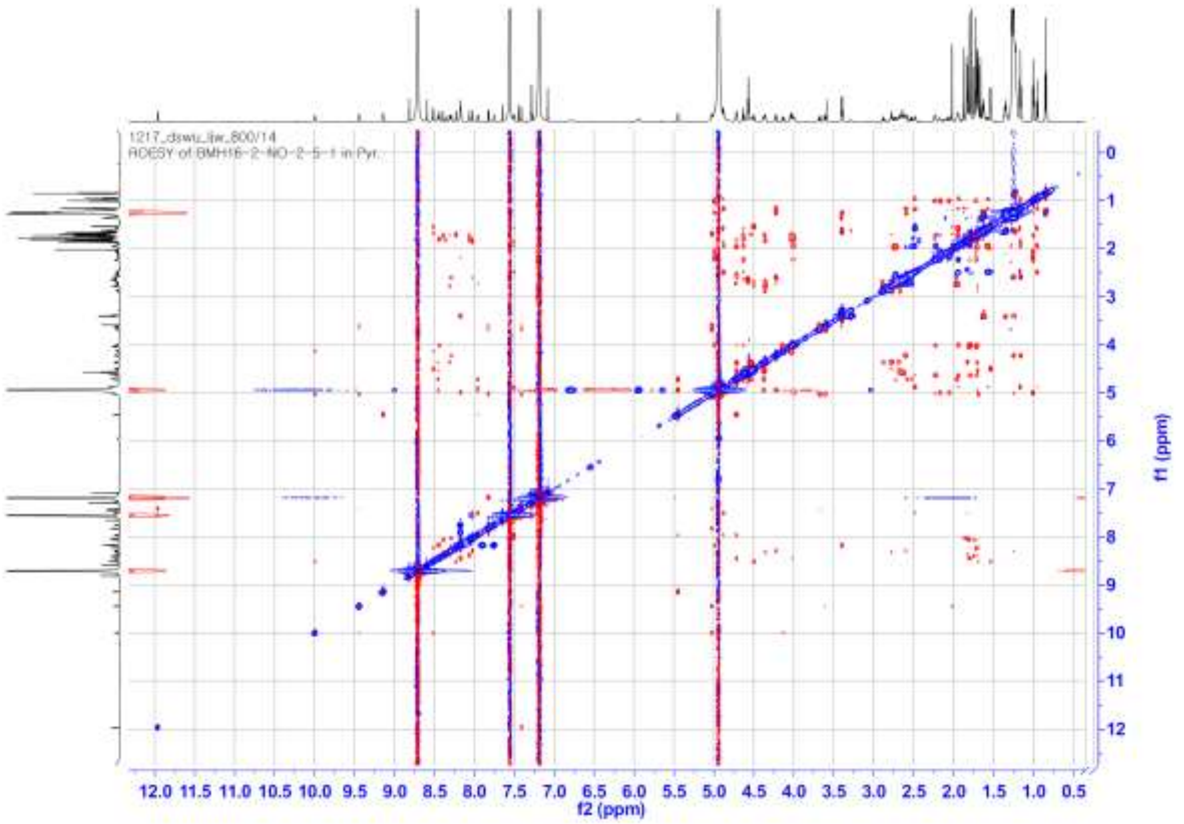

Figure S92. ROESY spectrum of compound **8**

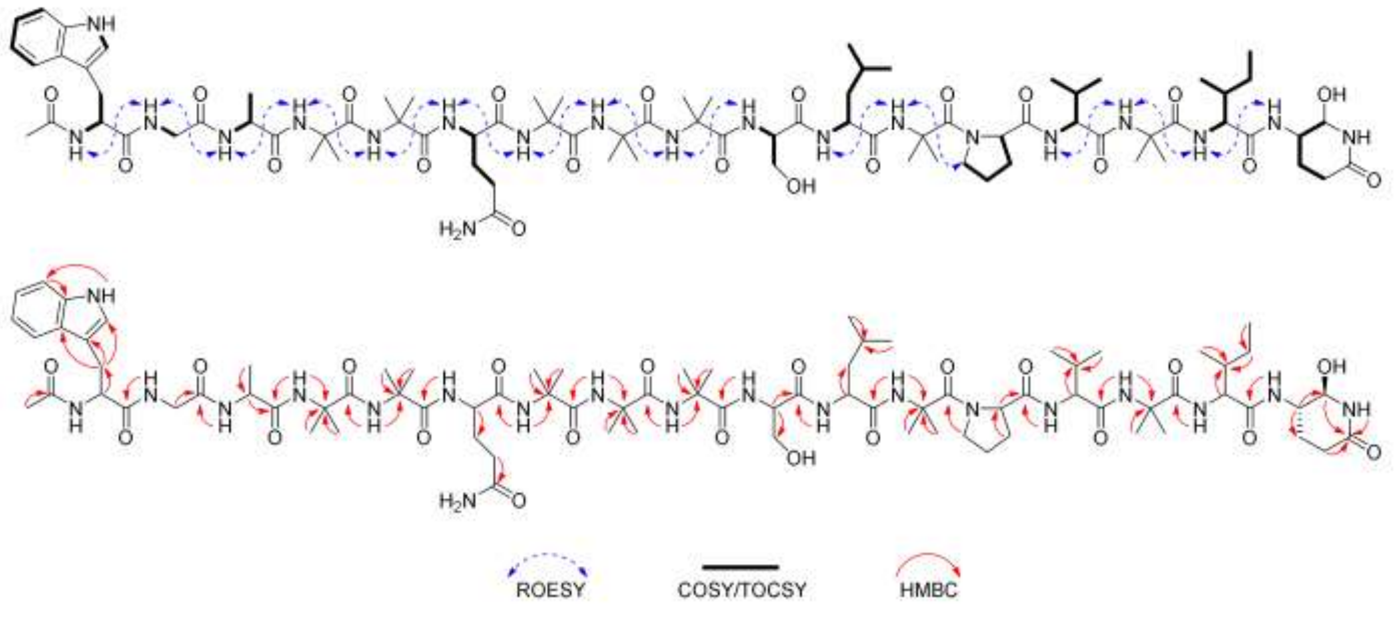

Figure S93. Key ROESY, COSY/TOCSY, and HMBC correlations of compound **8**

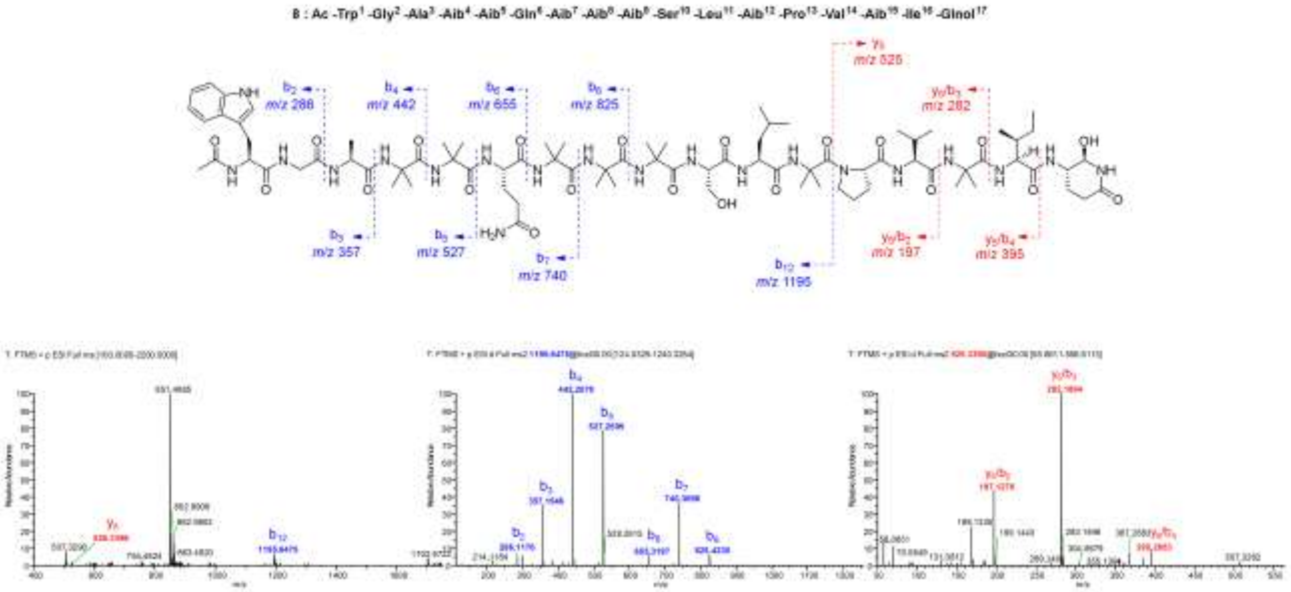

Figure S94. MS fragmentation analysis of compound **8**

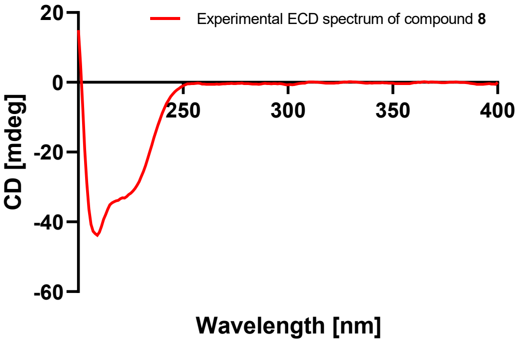

Figure S95. Experimental ECD spectrum of compound **8**

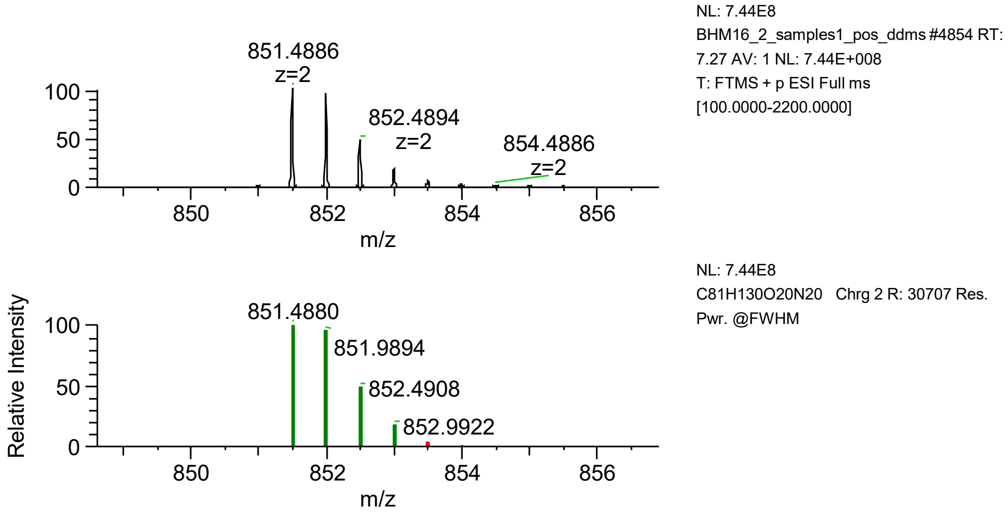

Figure S96. HRESIMS spectrum of compound **8**

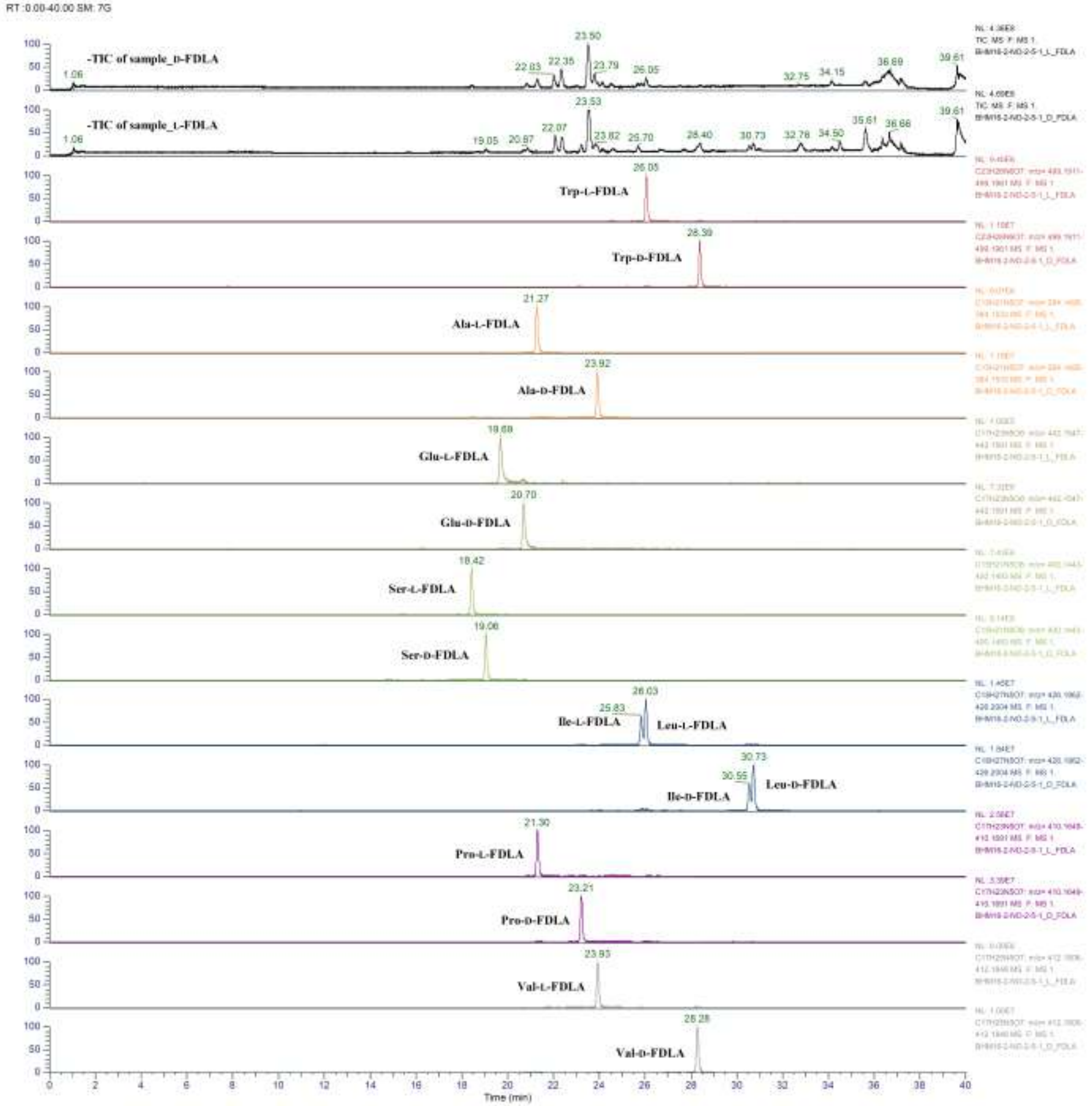

Figure S97. Advanced Marfey's analysis of compound **8**

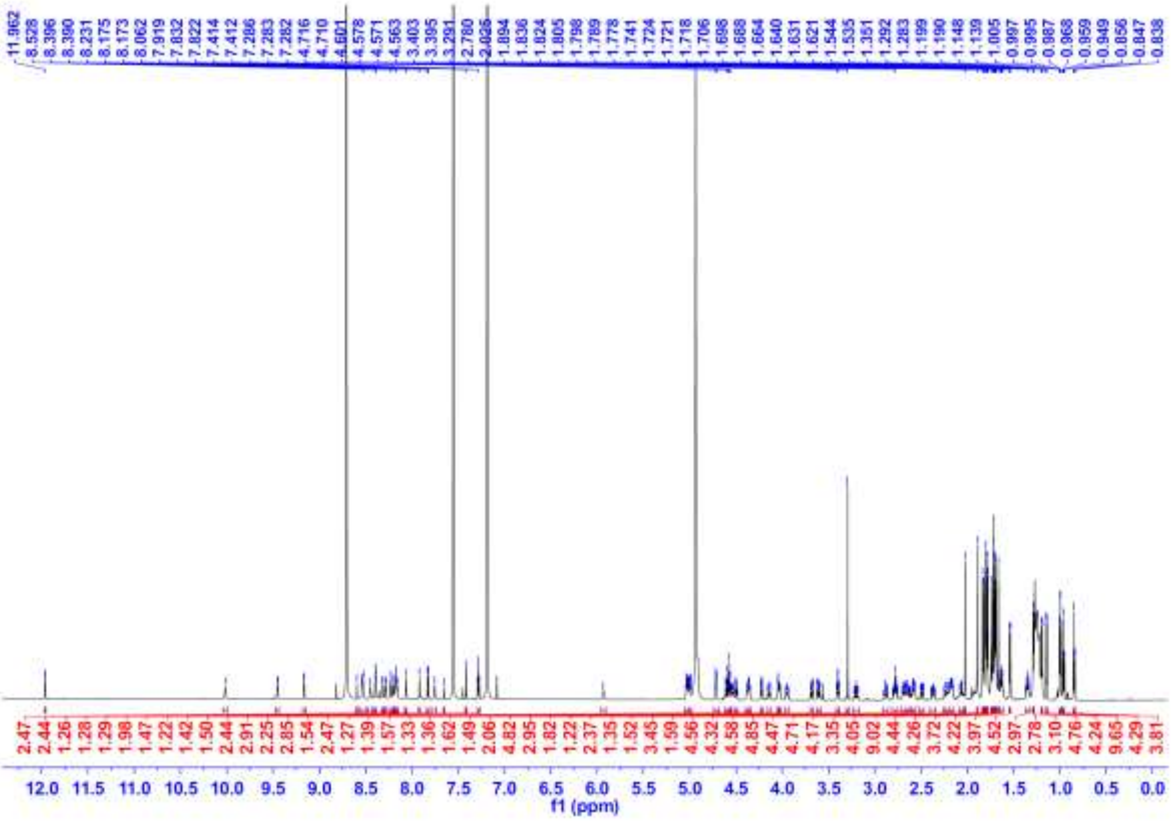

Figure S98. ^{1}H-NMR spectrum of compound **9** (Pyridine-d_5, 800 MHz)

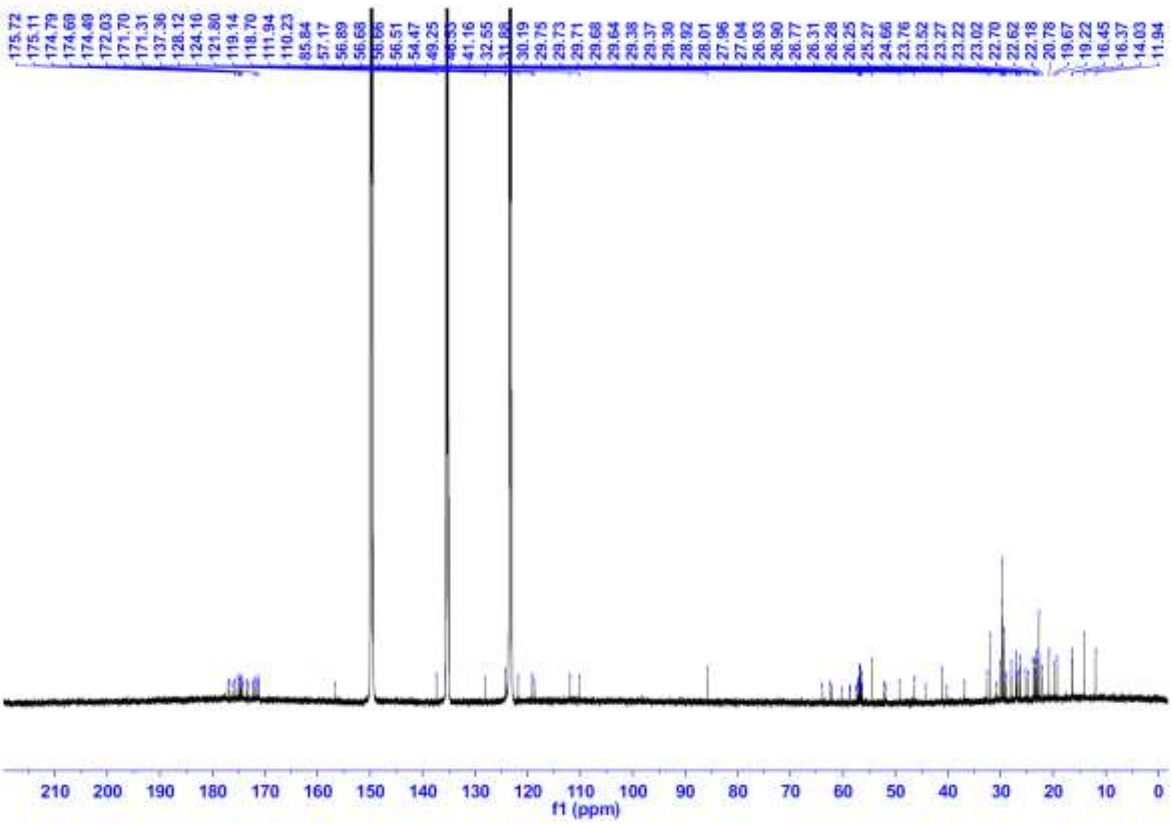

Figure S99. ^{13}C-NMR spectrum of compound **9** (Pyridine-d_5, 200 MHz)

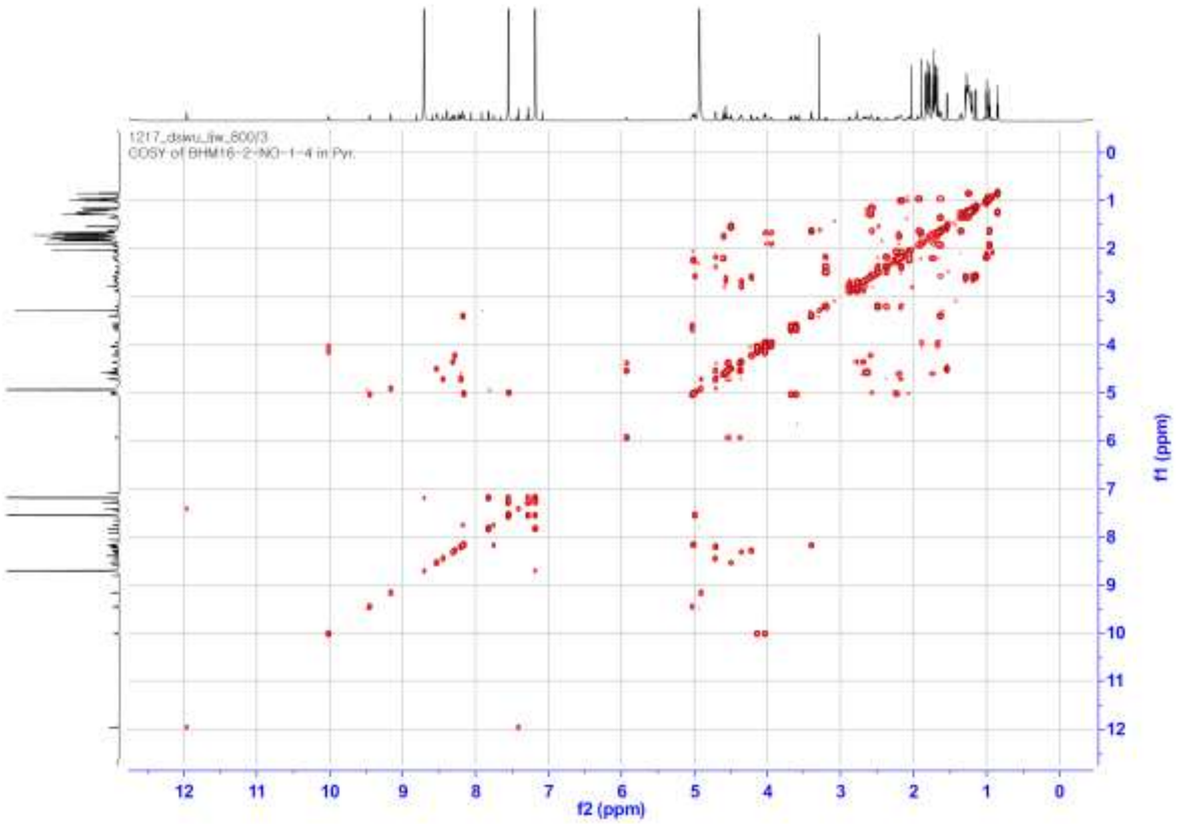

Figure S100. COSY spectrum of compound **9**

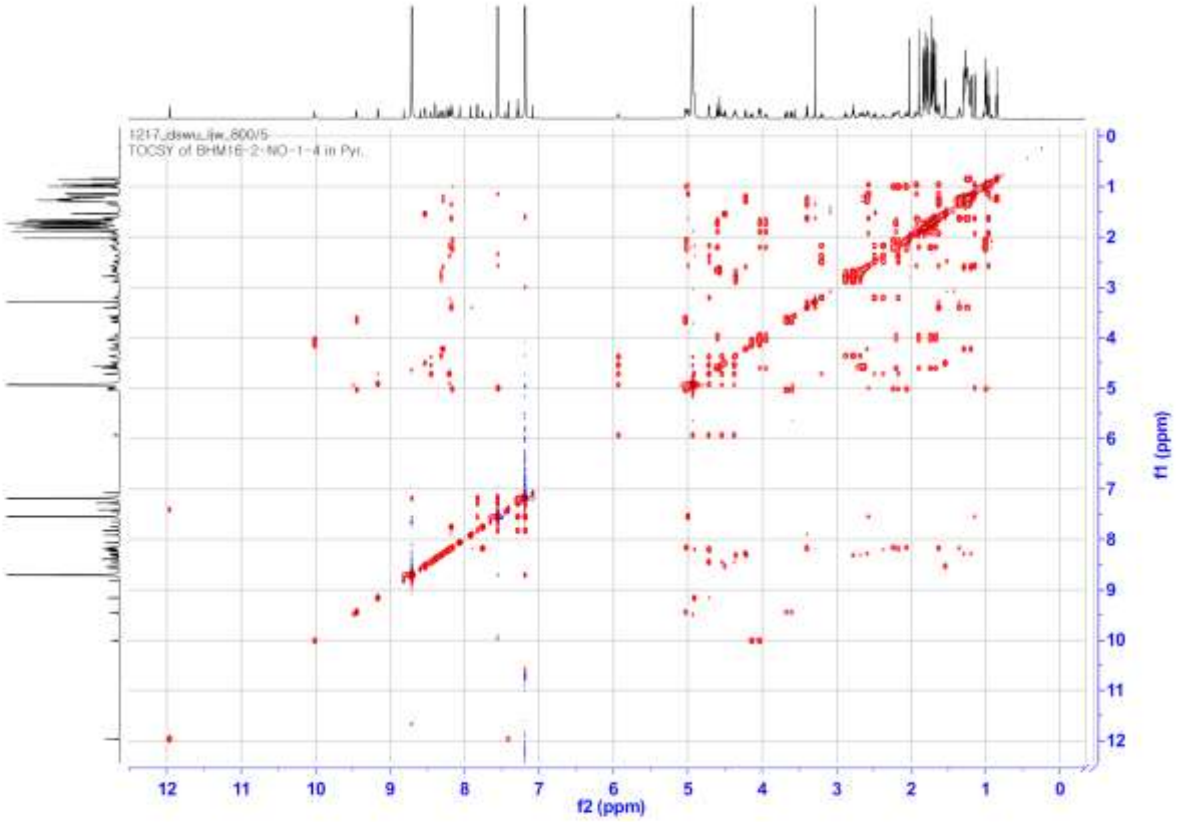

Figure S101. TOCSY spectrum of compound **9**

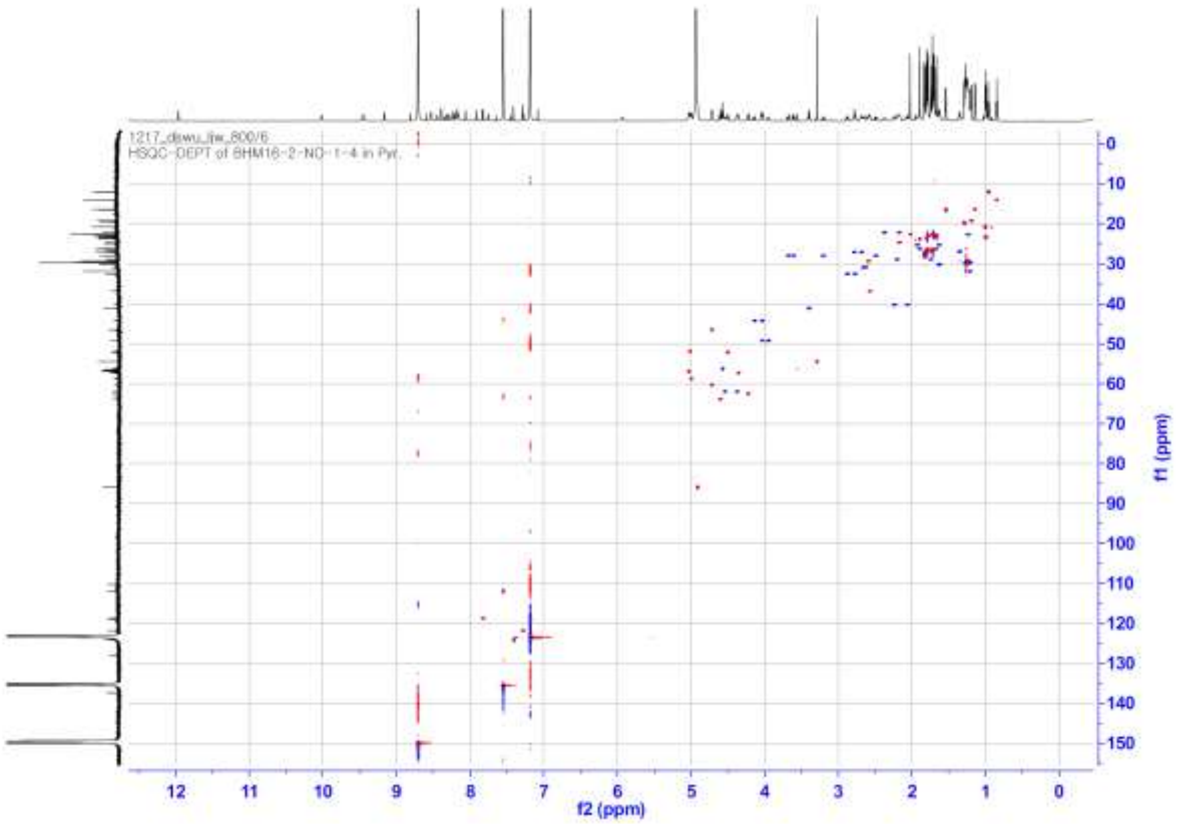

Figure S102. HSQC-DEPT spectrum of compound **9**

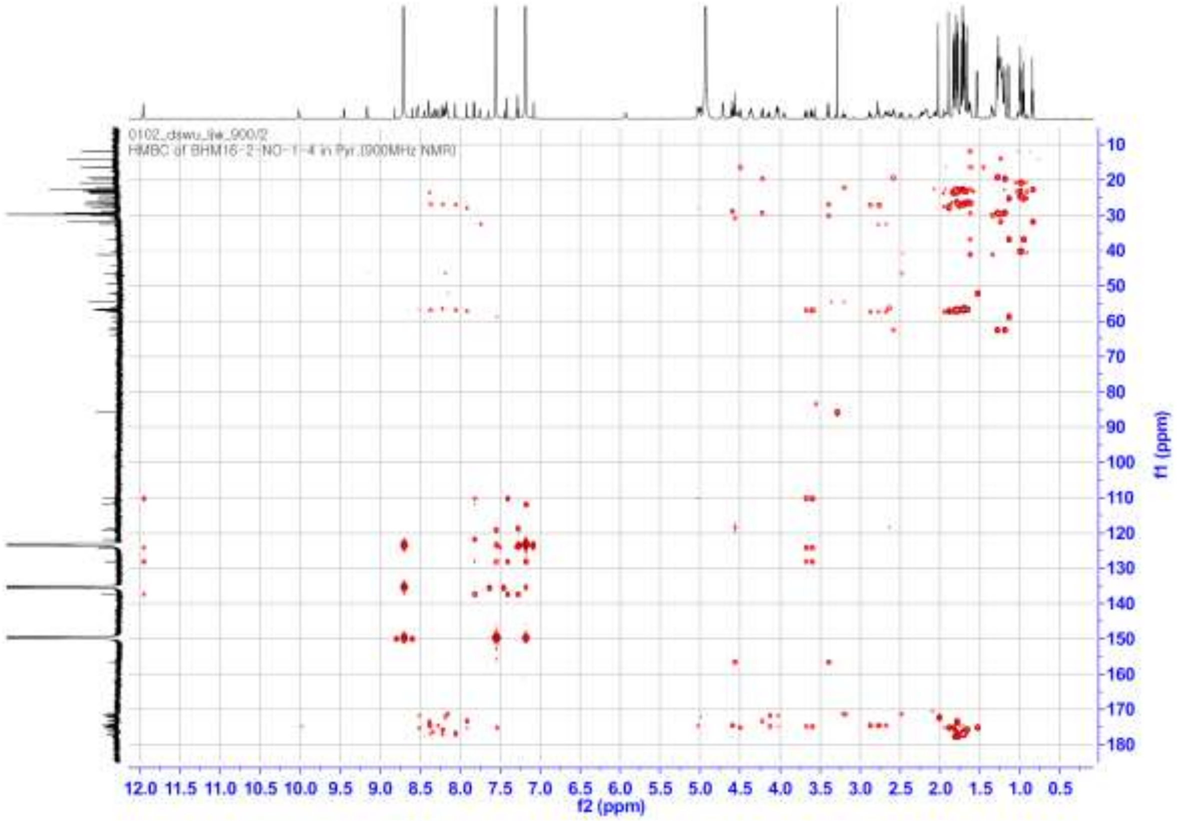

Figure S103. HMBC spectrum of compound **9**

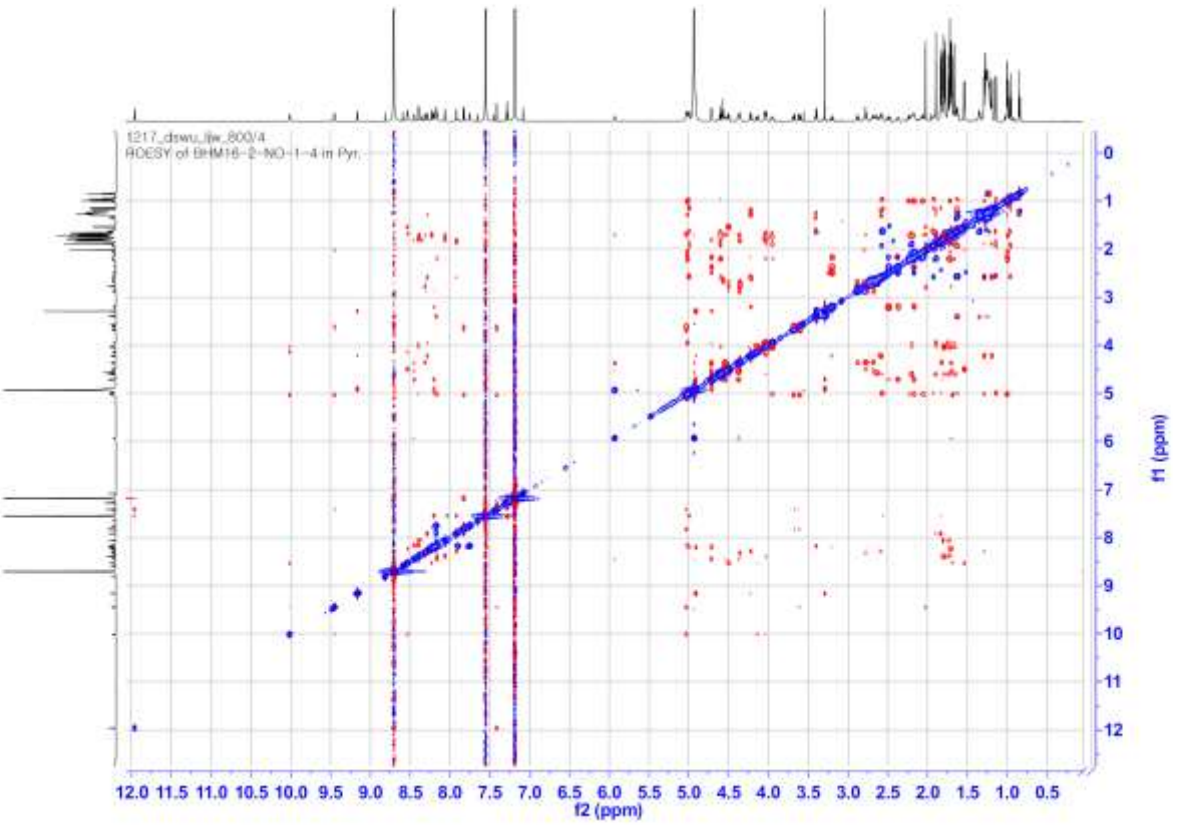

Figure S104. ROESY spectrum of compound **9**

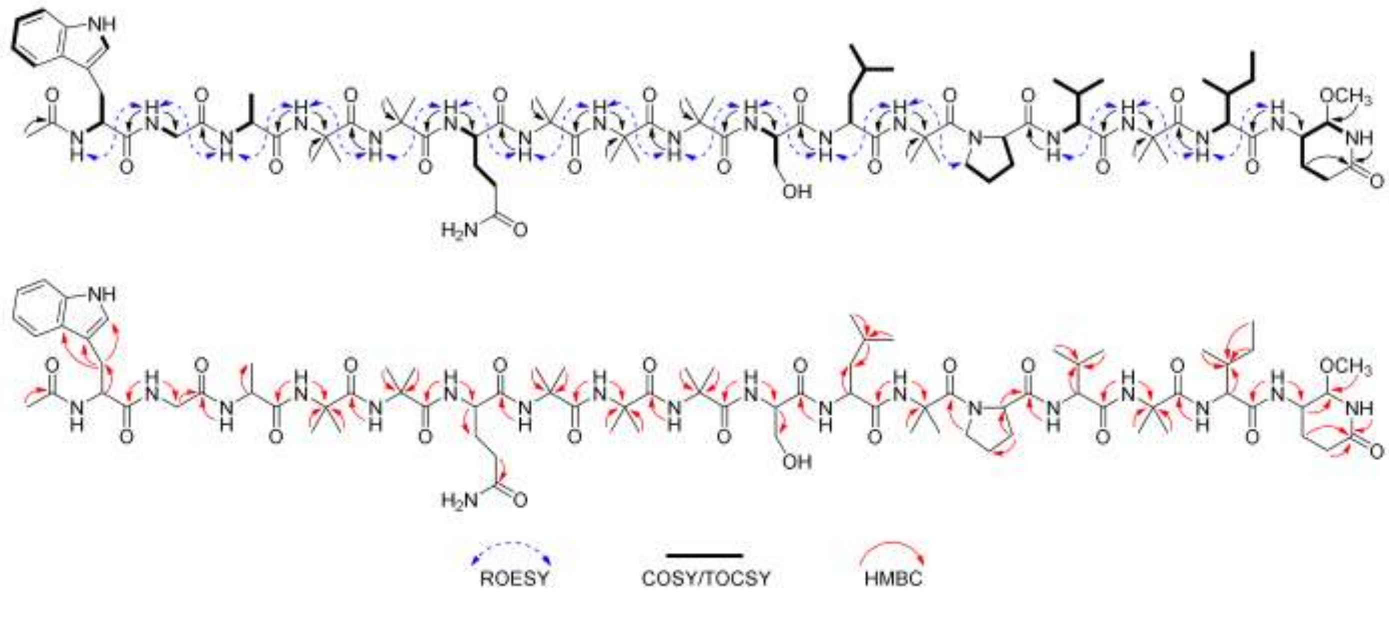

Figure S105. Key ROESY, COSY/TOCSY, and HMBC correlations of compound **9**

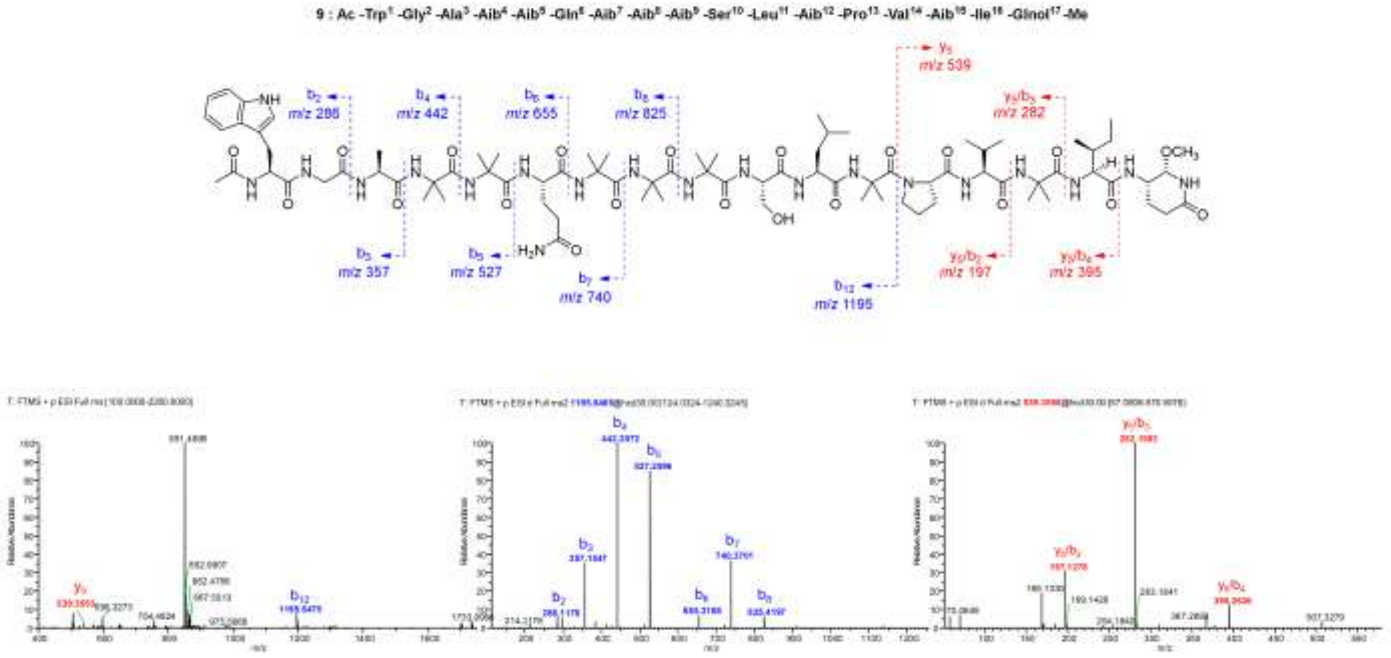

Figure S106. MS fragmentation analysis of compound **9**

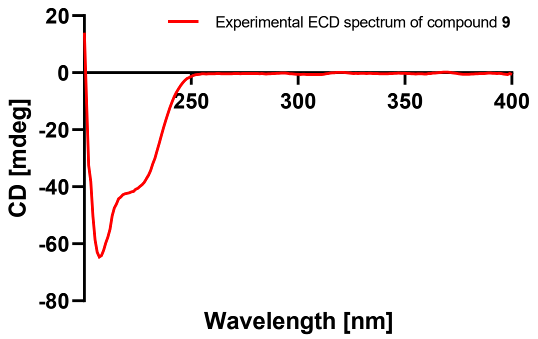

Figure S107. Experimental ECD spectrum of compound **9**

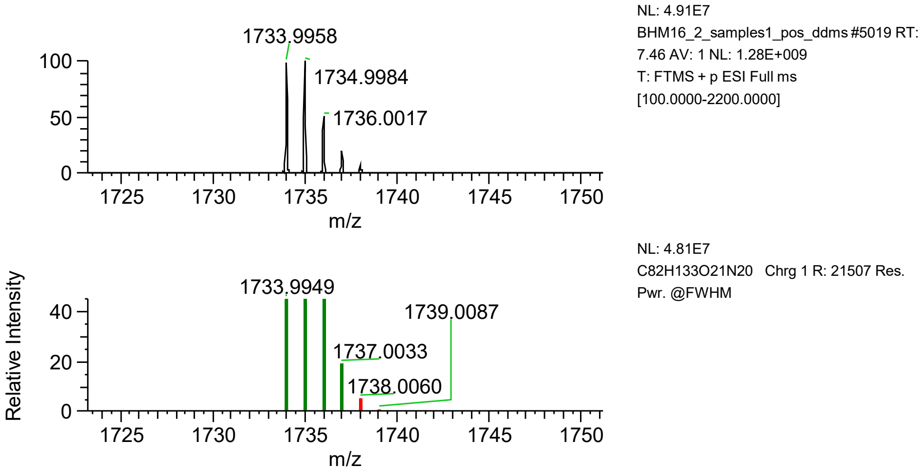

Figure S108. HRESIMS spectrum of compound **9**

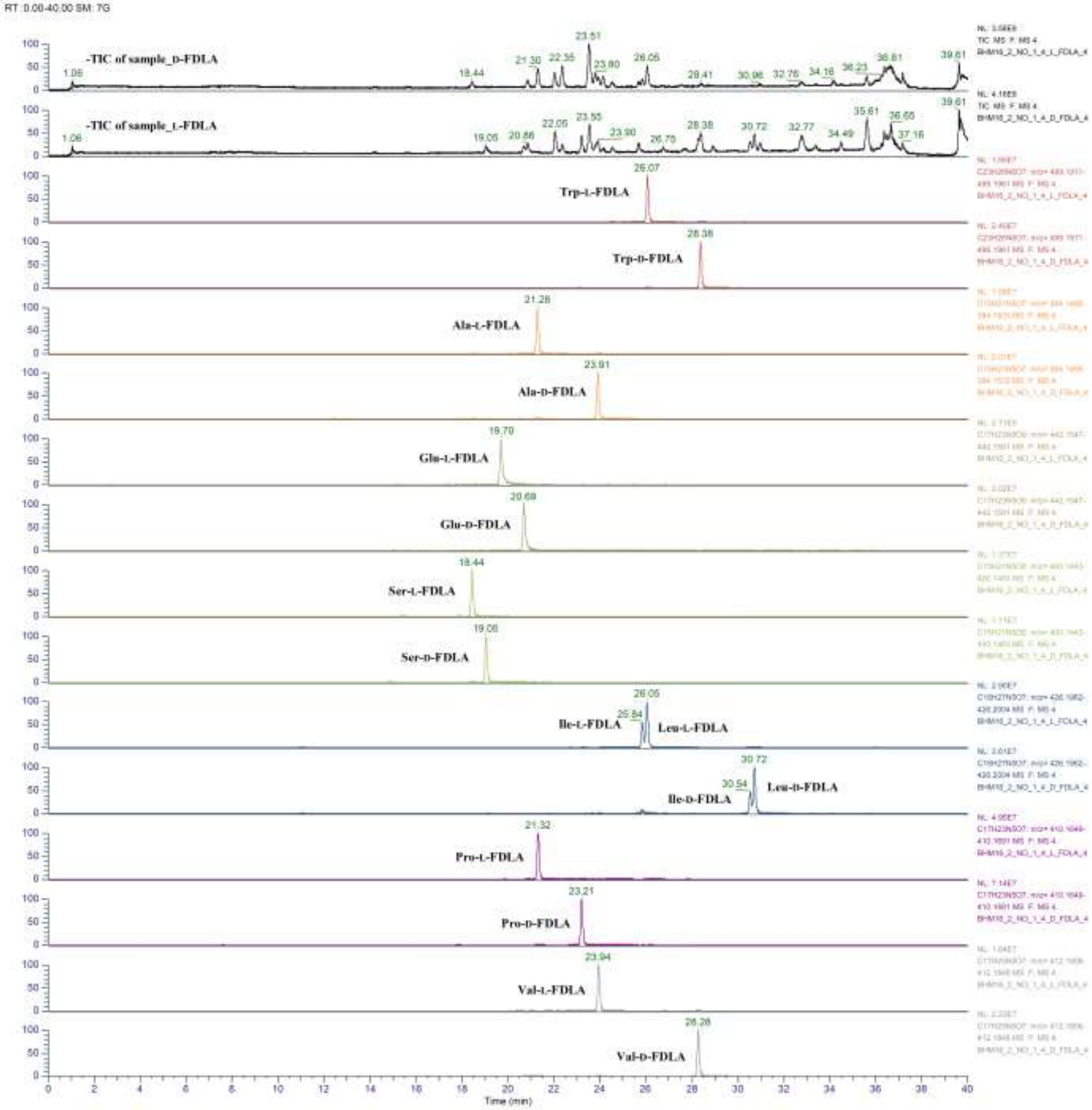

Figure S109. Advanced Marfey's analysis of compound **9**

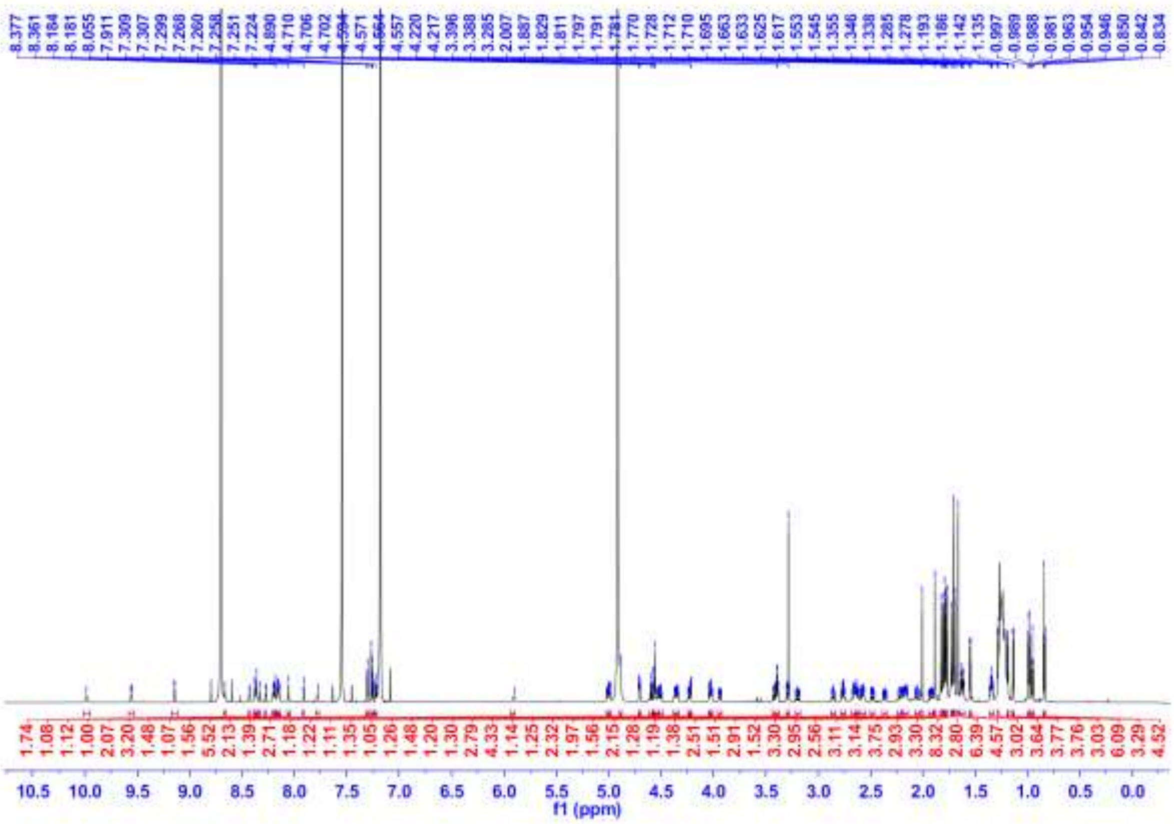

Figure S110. ^{1}H-NMR spectrum of compound **10** (Pyridine-d_5, 900 MHz)

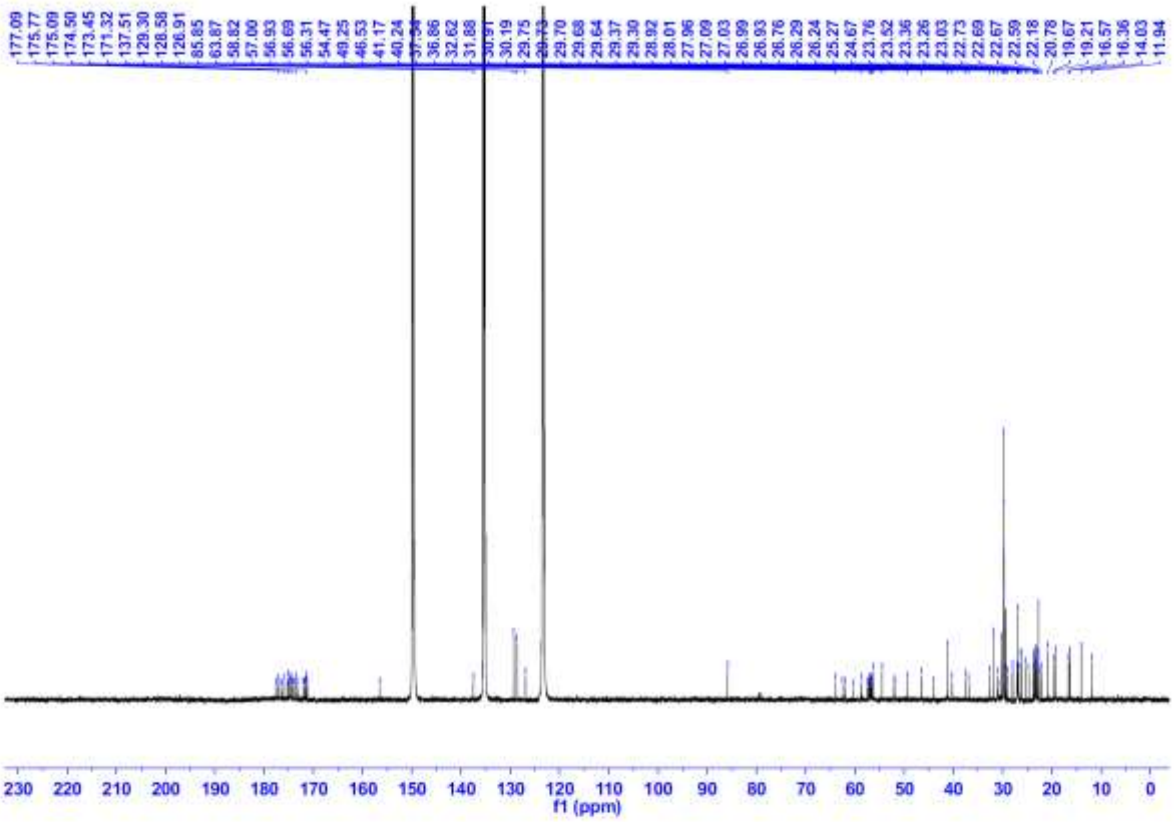

Figure S111. ^{13}C-NMR spectrum of compound **10** (Pyridine-d_5, 225 MHz)

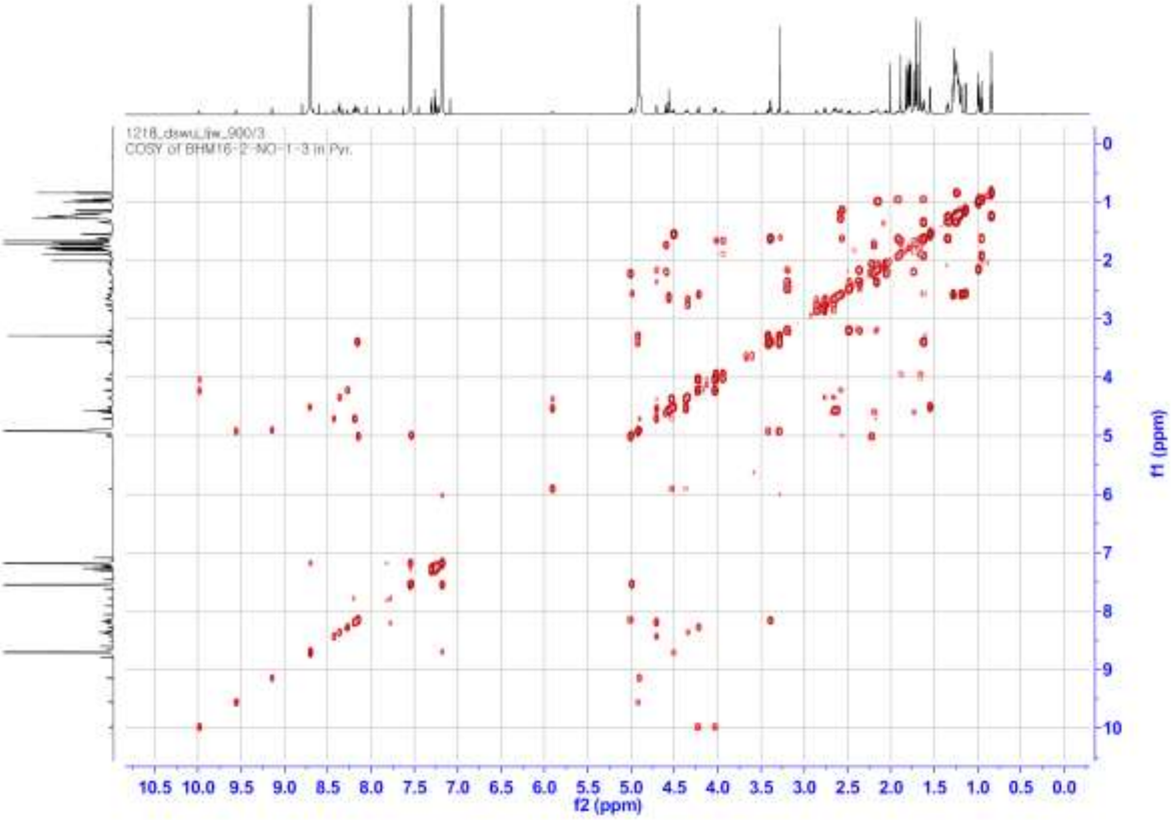

Figure S112. COSY spectrum of compound **10**

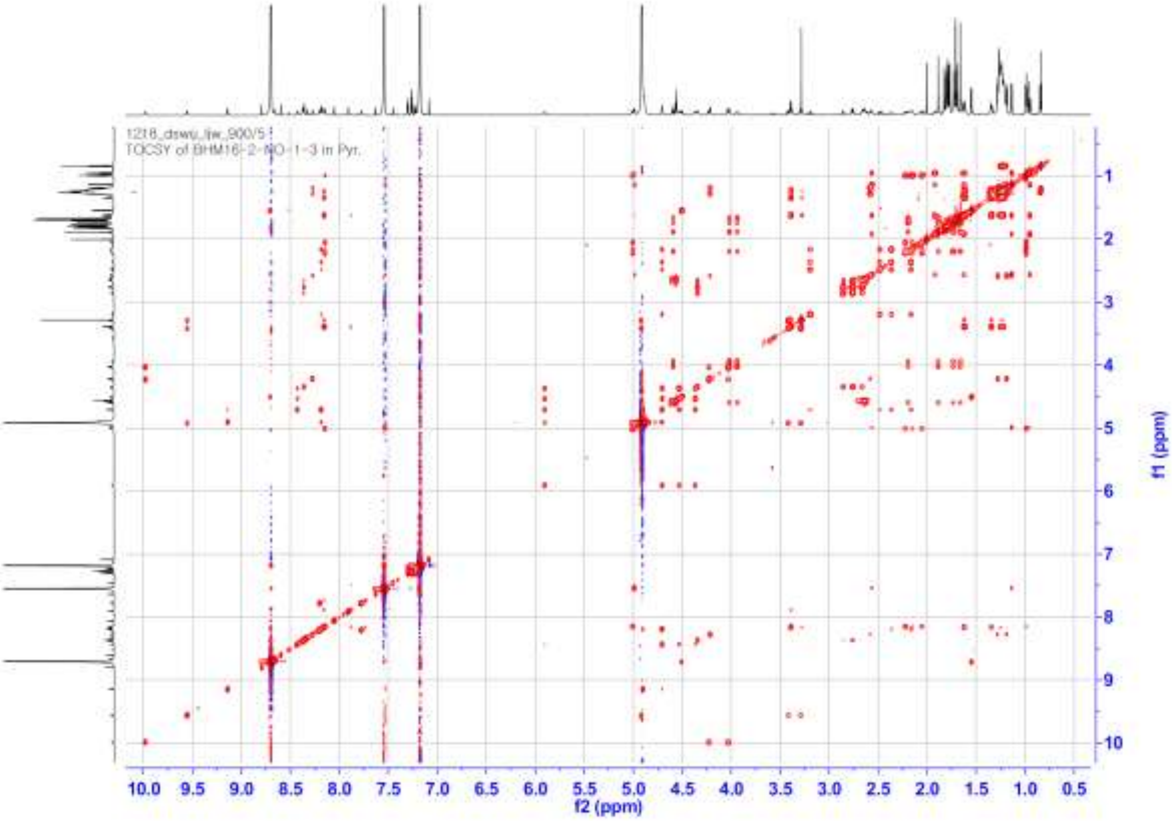

Figure S113. TOCSY spectrum of compound **10**

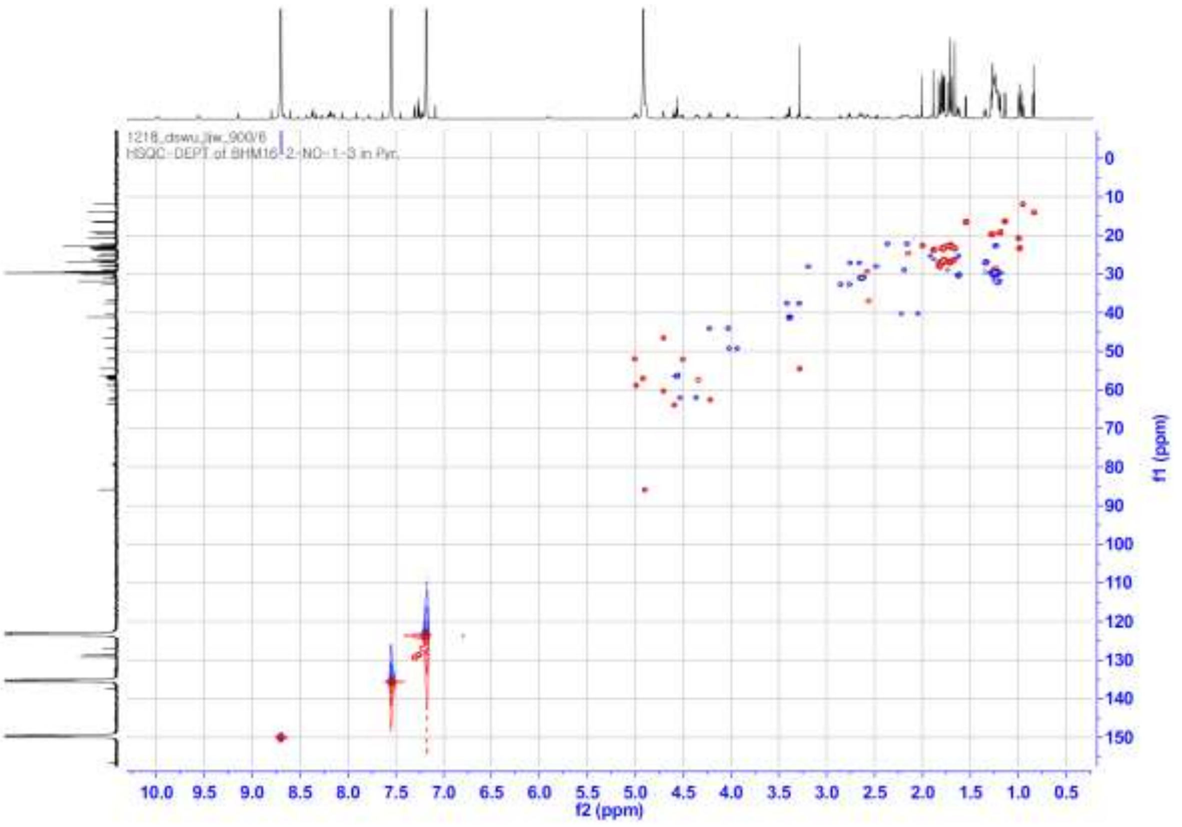

Figure S114. HSQC-DEPT spectrum of compound **10**

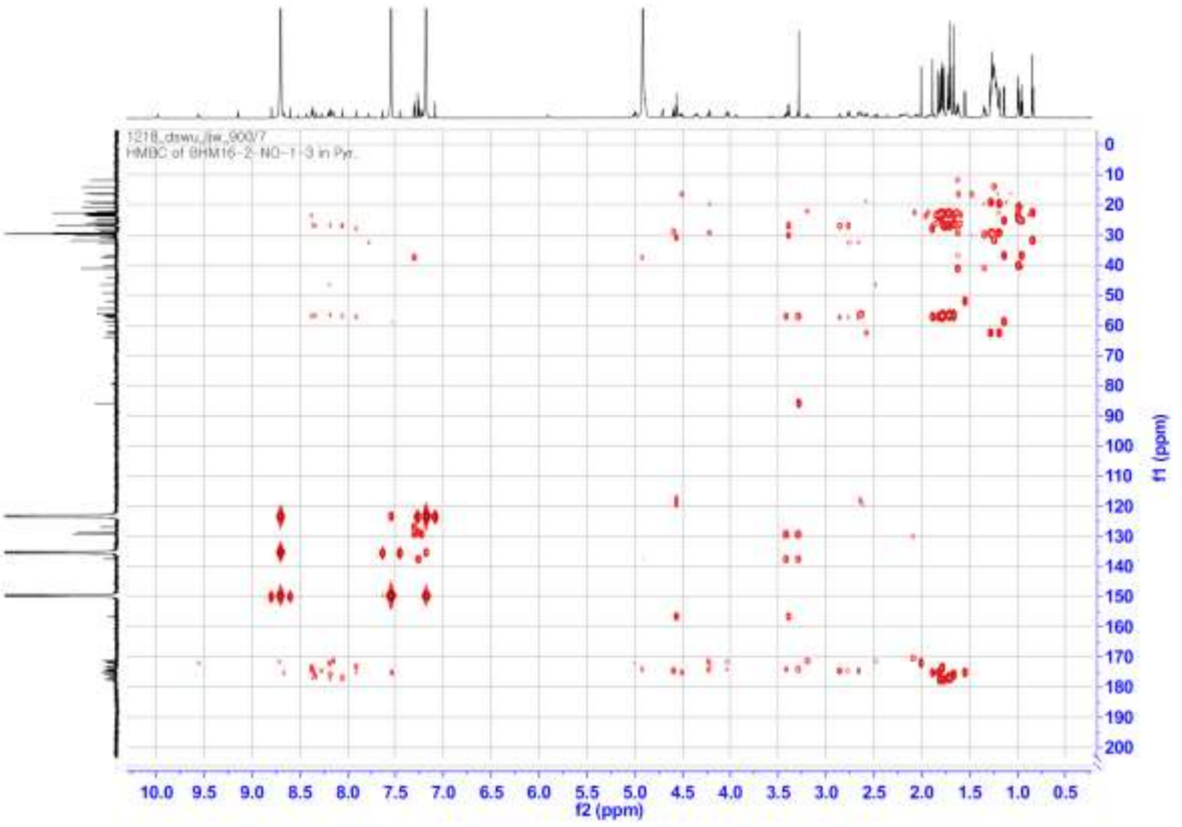

Figure S115. HMBC spectrum of compound **10**

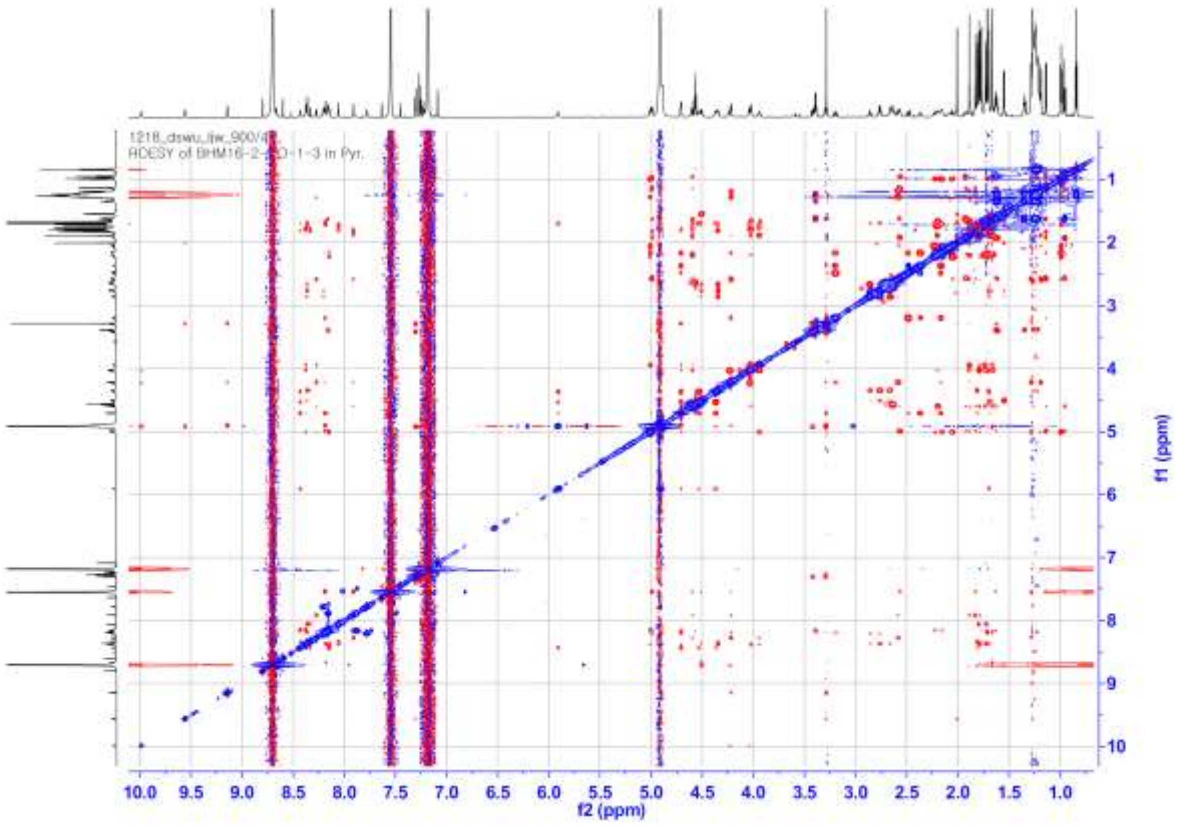

Figure S116. ROESY spectrum of compound **10**

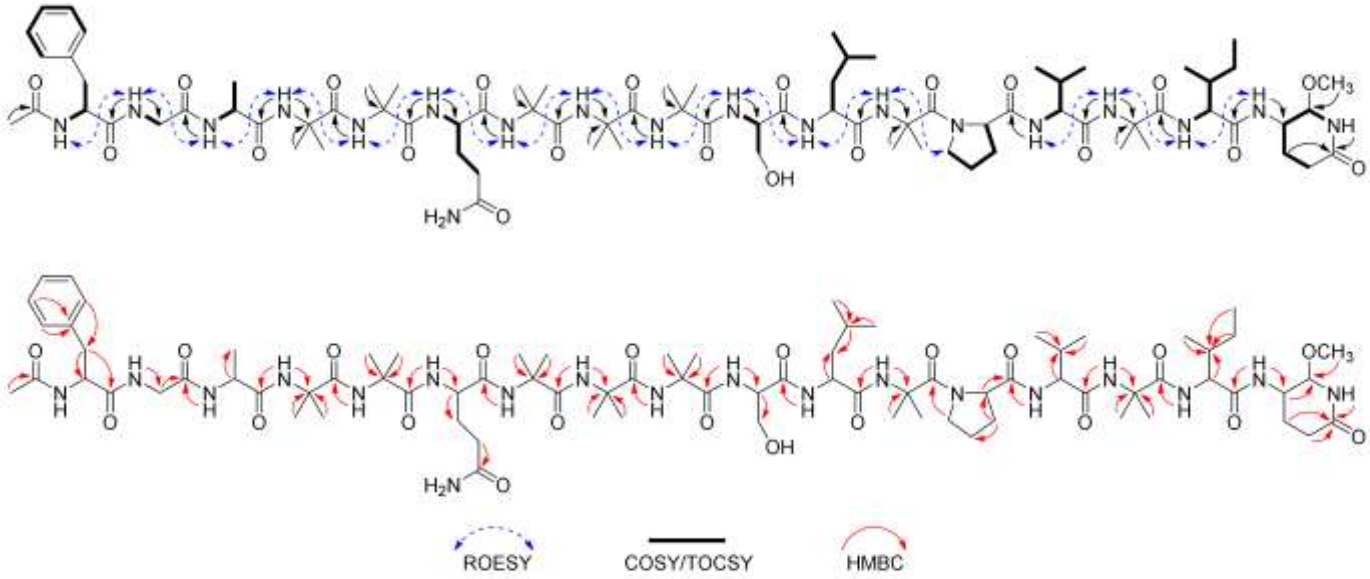

Figure S117. Key ROESY, COSY/TOCSY, and HMBC correlations of compound **10**

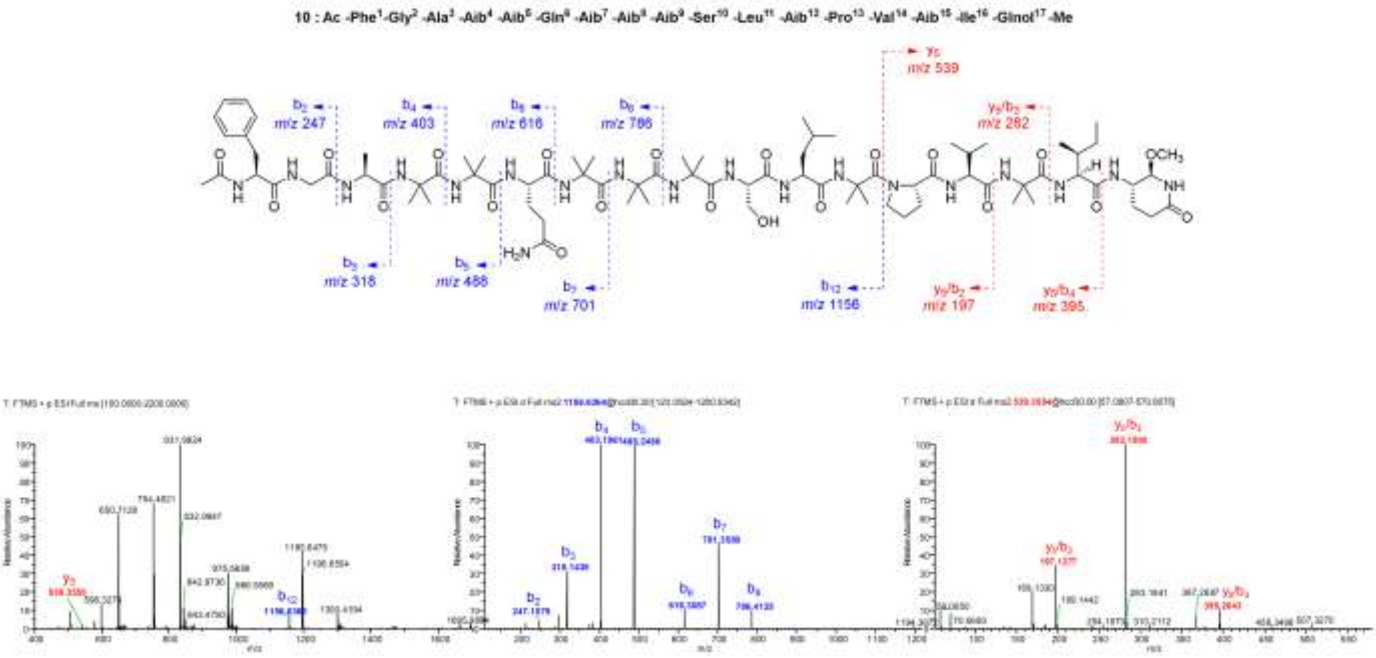

Figure S118. MS fragmentation analysis of compound **10**

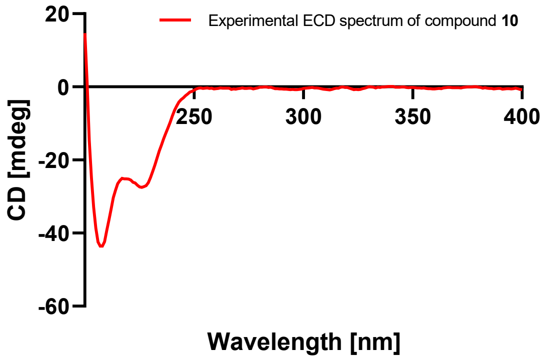

Figure S119. Experimental ECD spectrum of compound **10**

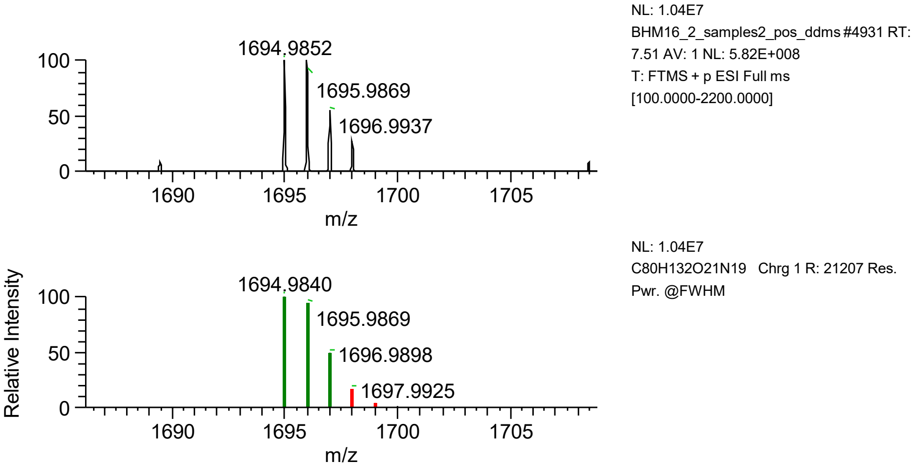

Figure S120. HRESIMS spectrum of compound **10**

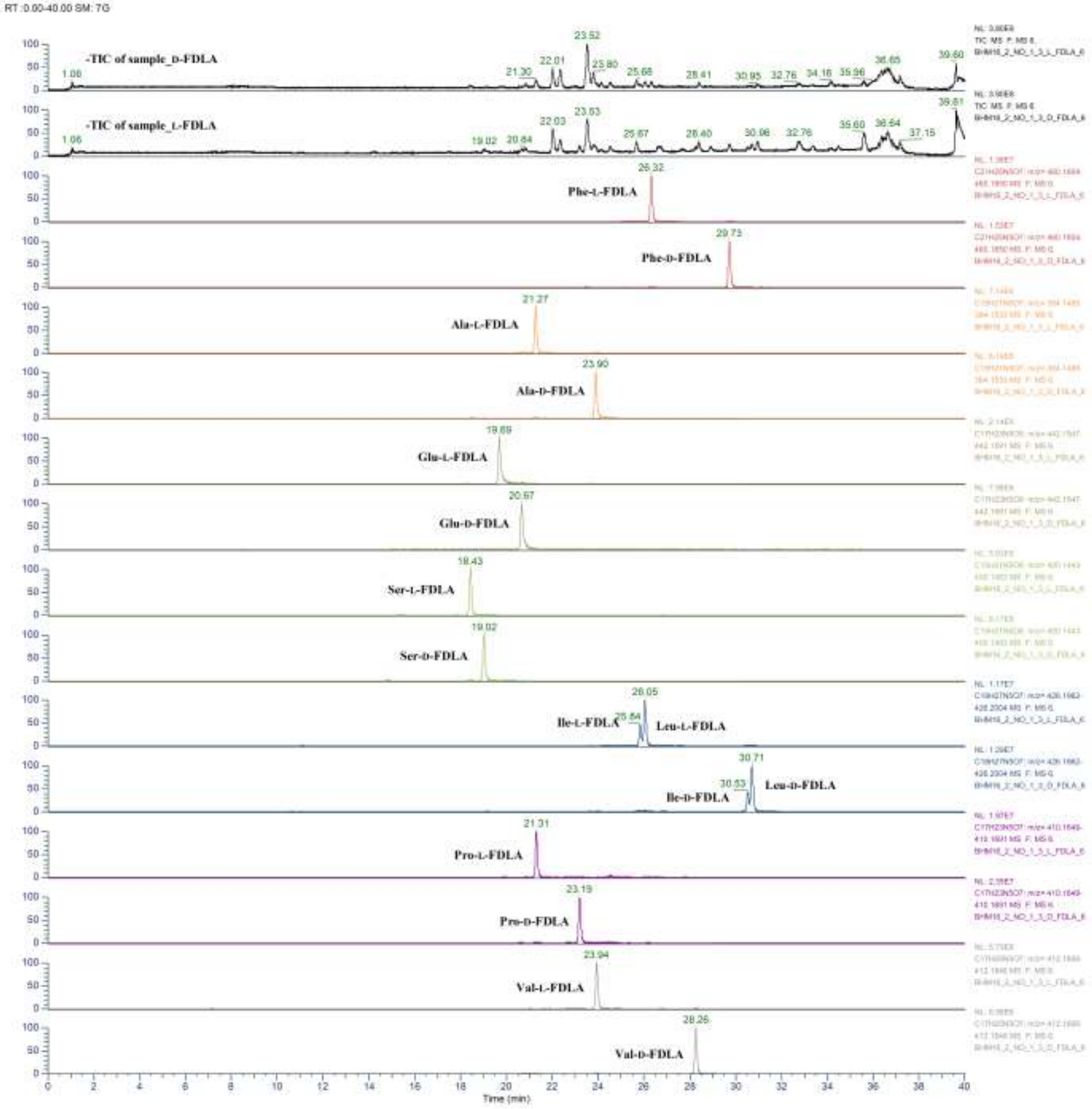

Figure S121. Advanced Marfey's analysis of compound **10**

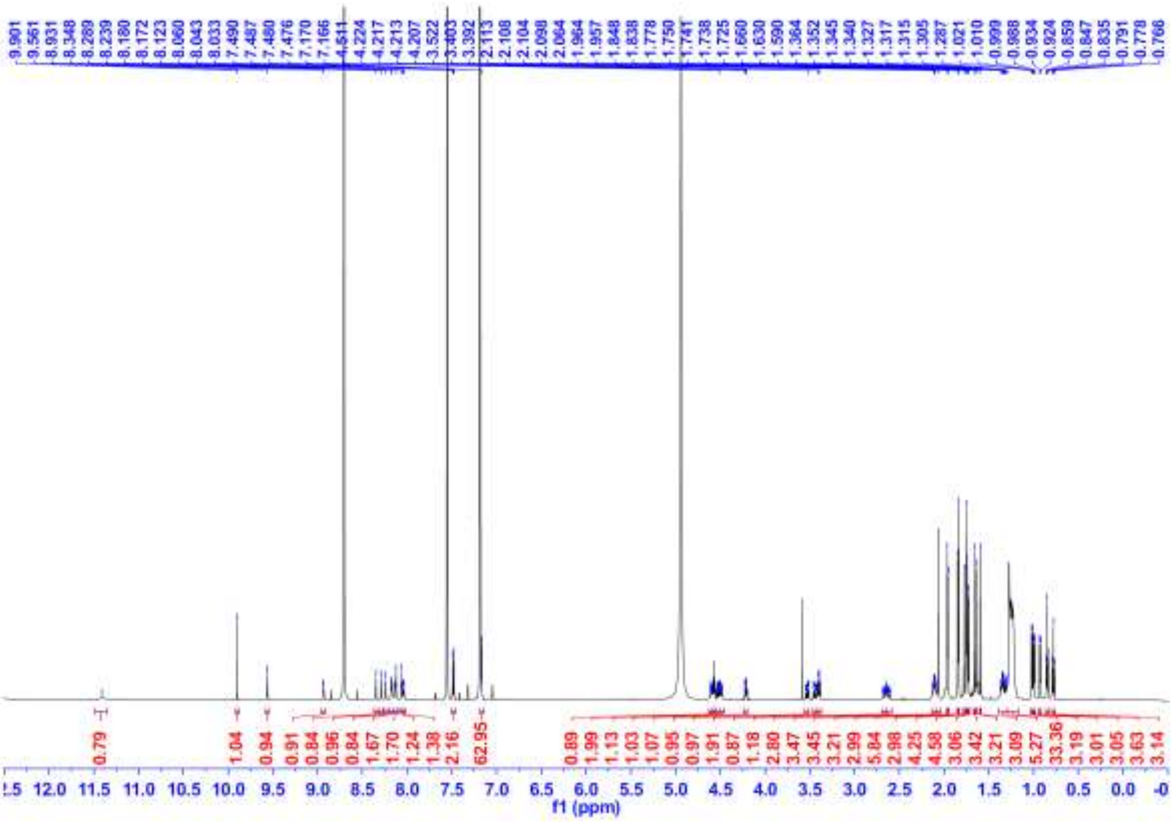

Figure S122. ^{1}H-NMR spectrum of compound **11** (Pyridine-d_5, 600 MHz)

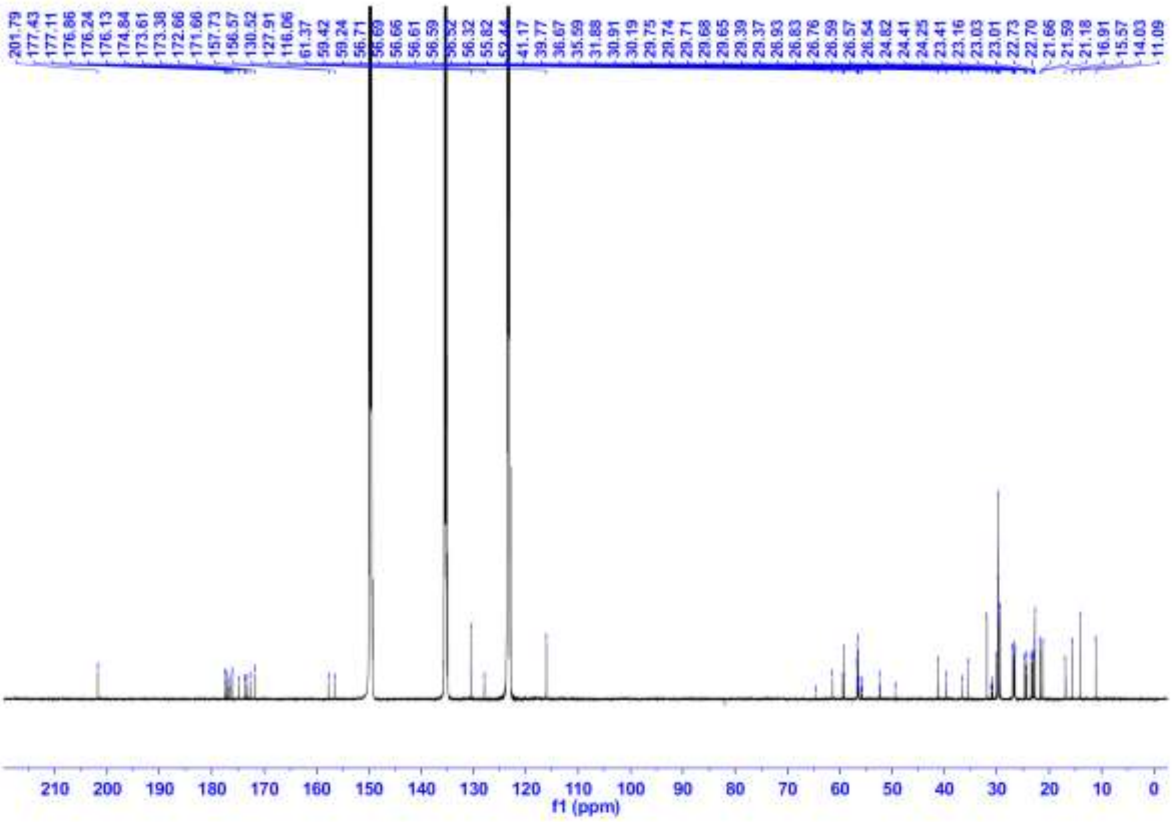

Figure S123. ^{13}C-NMR spectrum of compound **11** (Pyridine-d_5, 150 MHz)

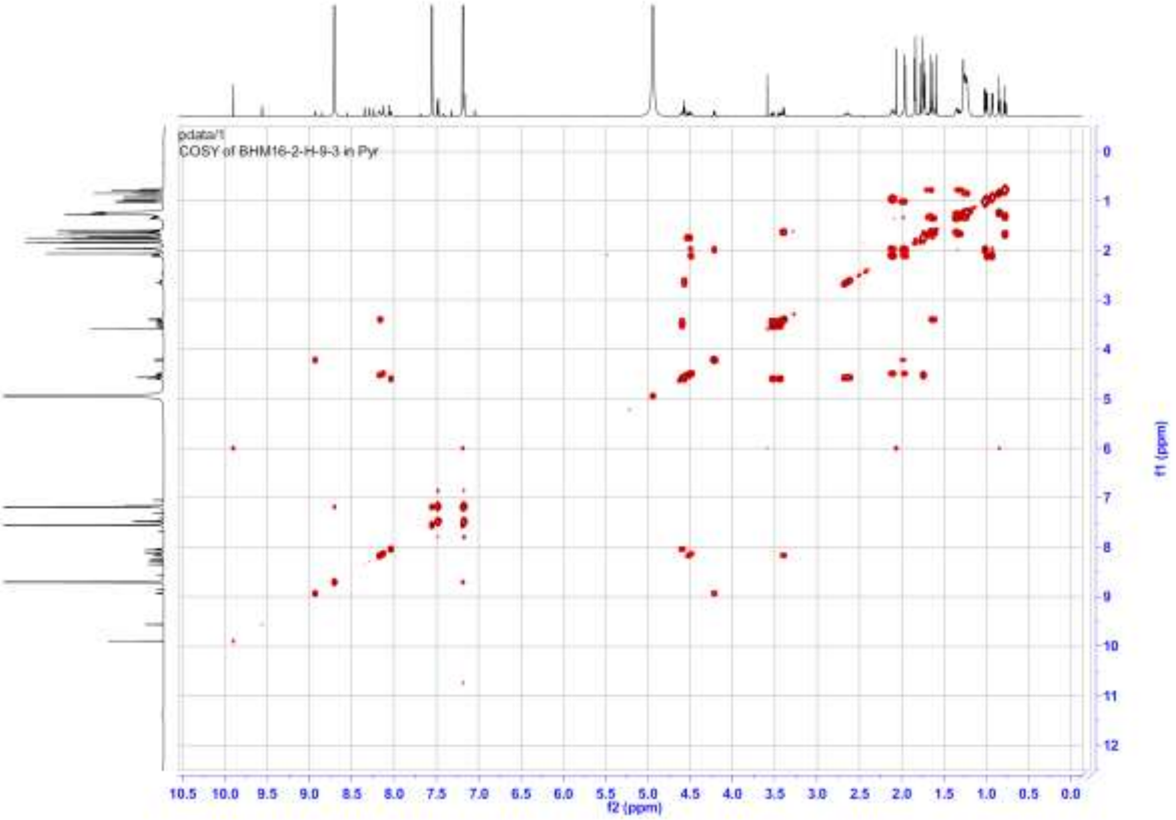

Figure S124. COSY spectrum of compound **11**

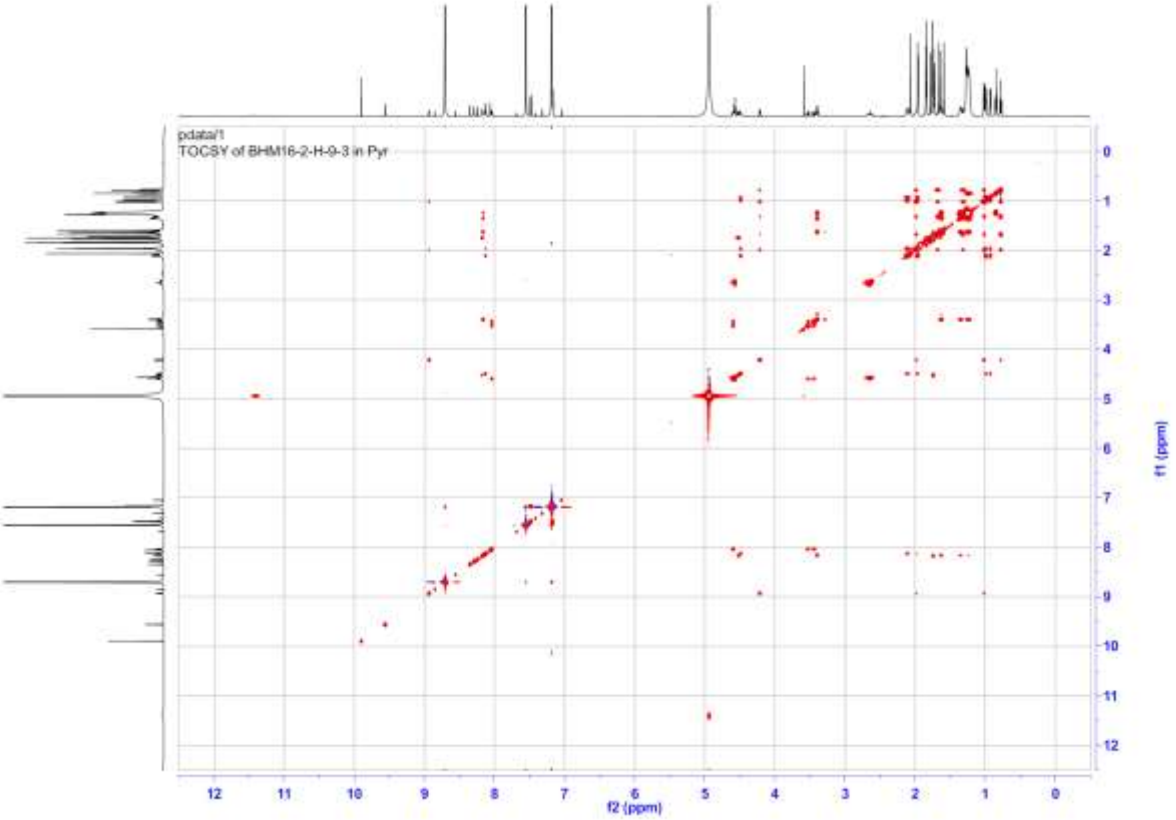

Figure S125. TOCSY spectrum of compound **11**

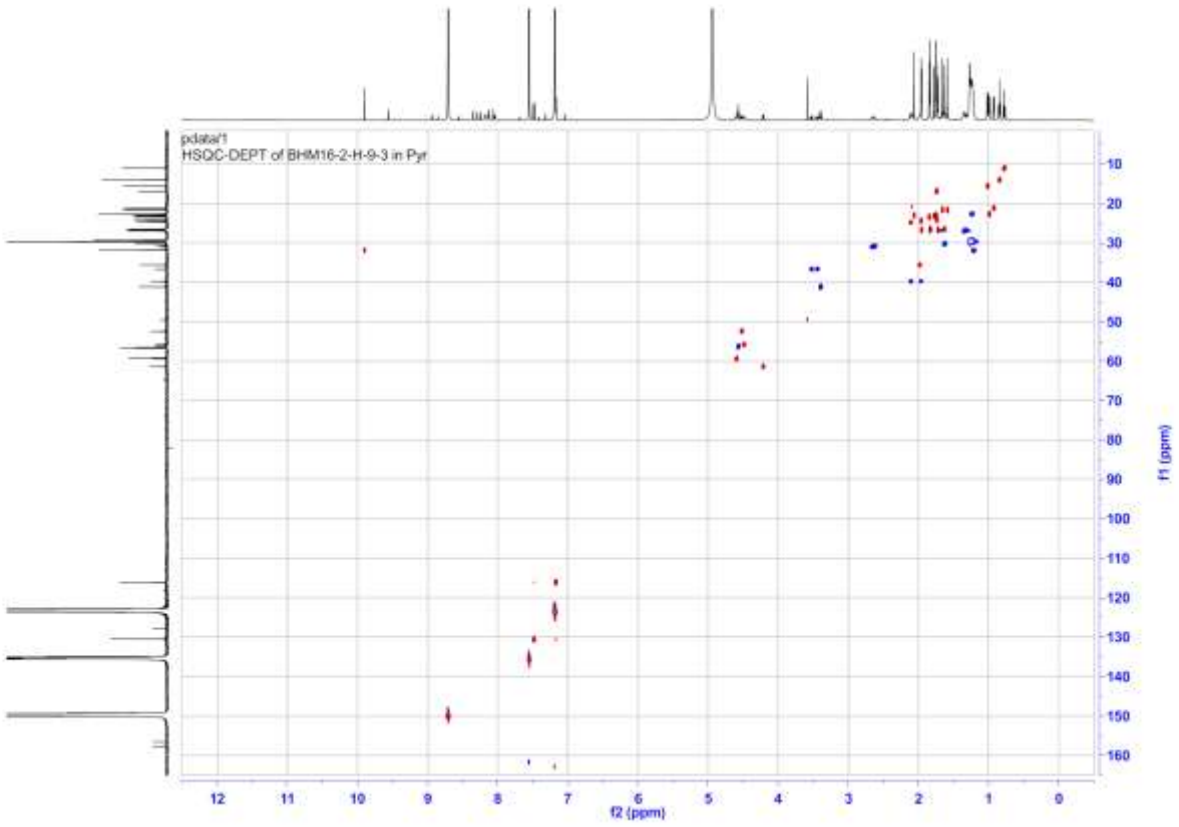

Figure S126. HSQC-DEPT spectrum of compound **11**

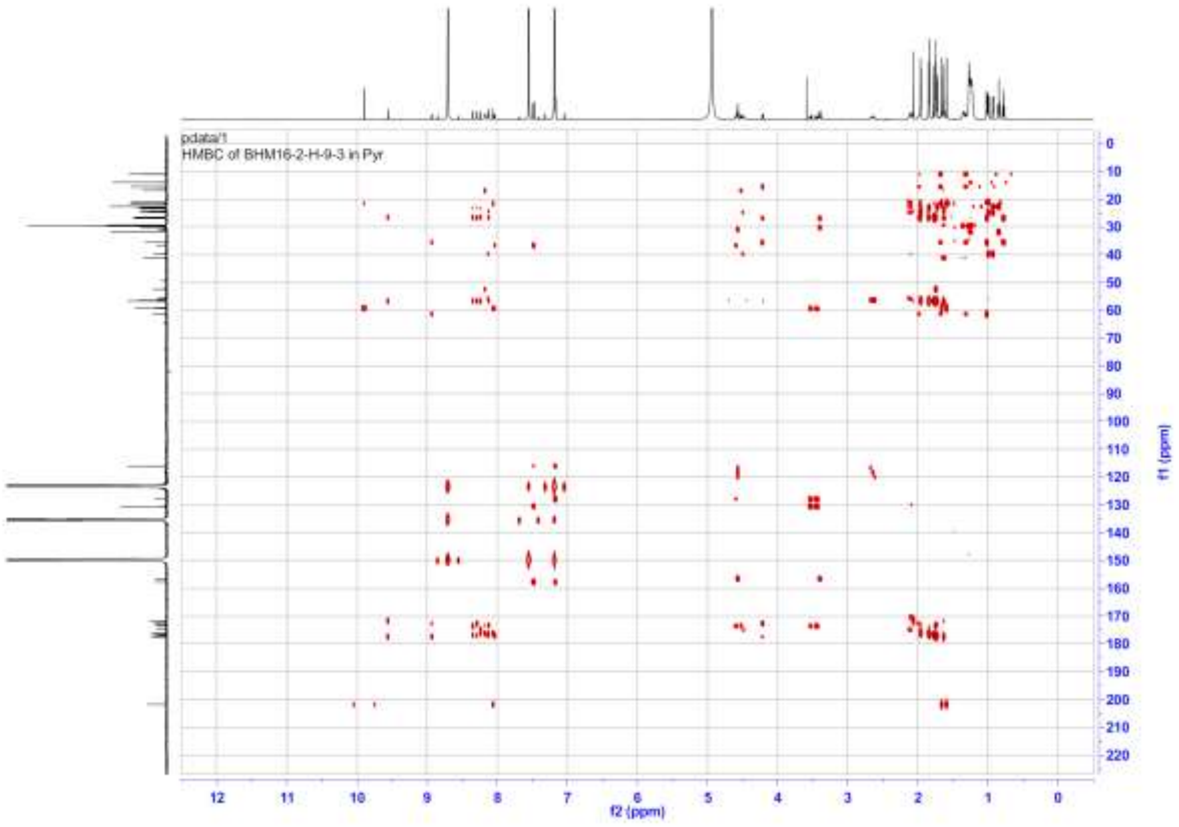

Figure S127. HMBC spectrum of compound **11**

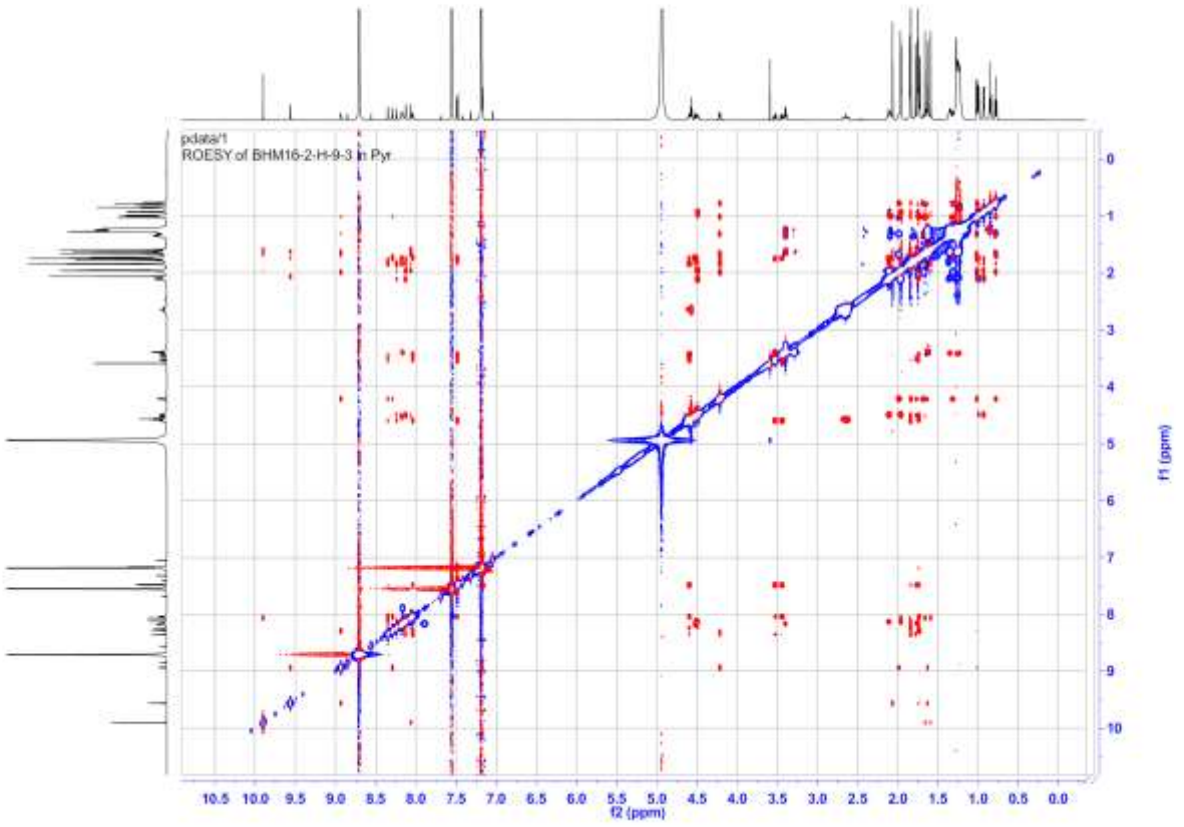

Figure S128. ROESY spectrum of compound **11**

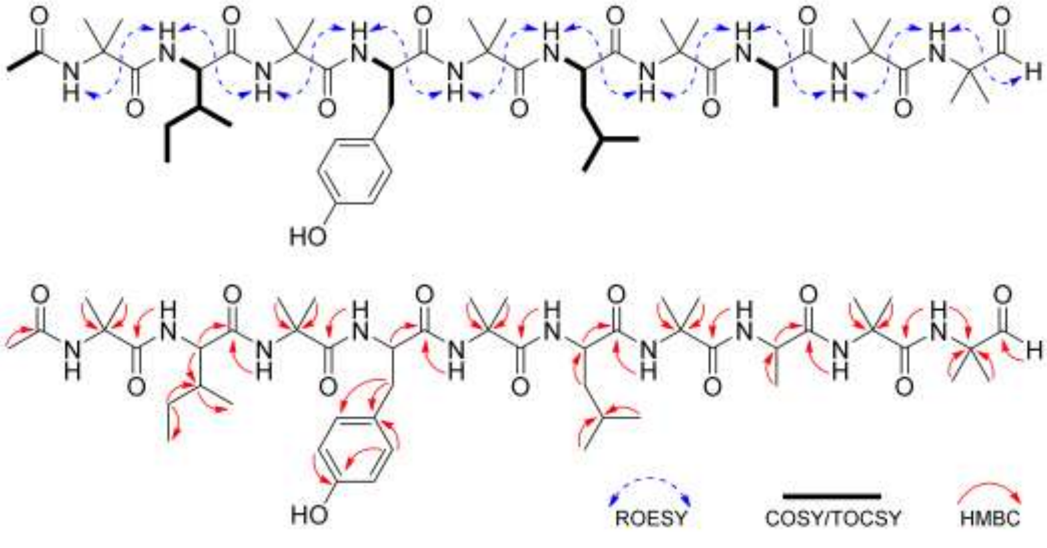

Figure S129. Key ROESY, COSY/TOCSY, and HMBC correlations of compound **11**

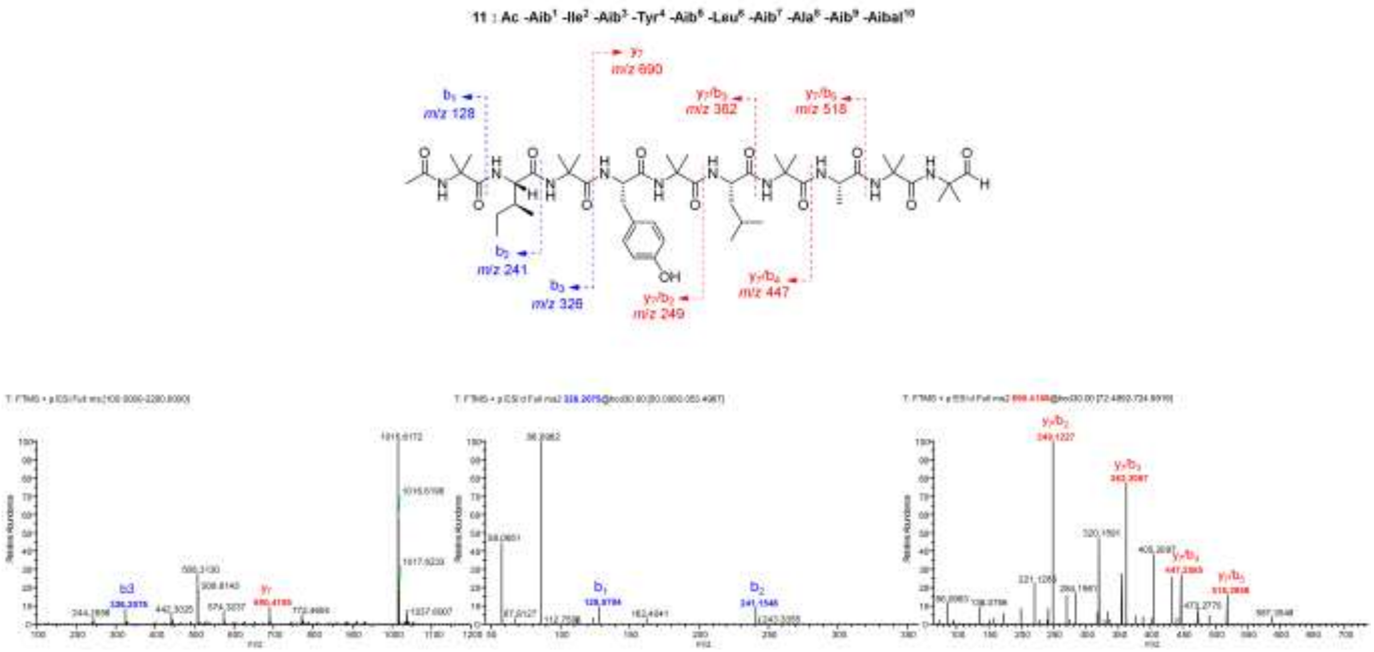

Figure S130. MS fragmentation analysis of compound **11**

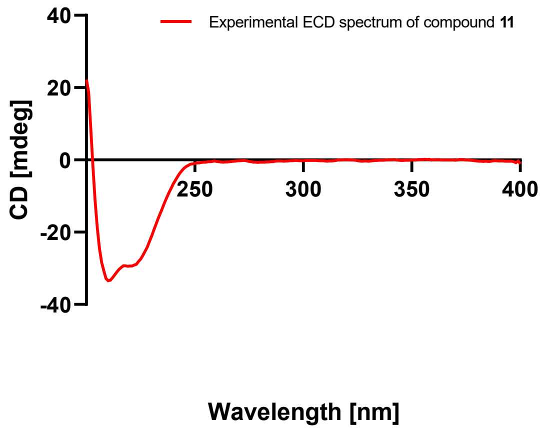

Figure S131. Experimental ECD spectrum of compound **11**

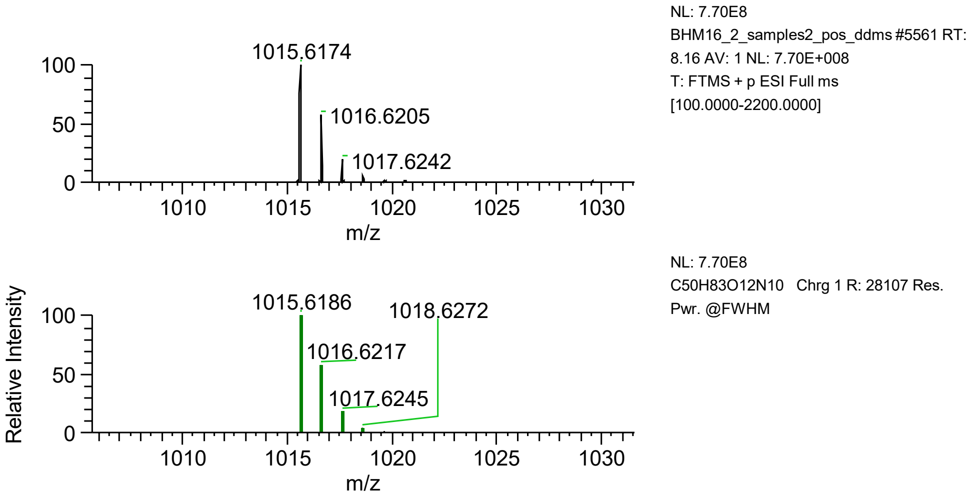

Figure S132. HRESIMS spectrum of compound **11**

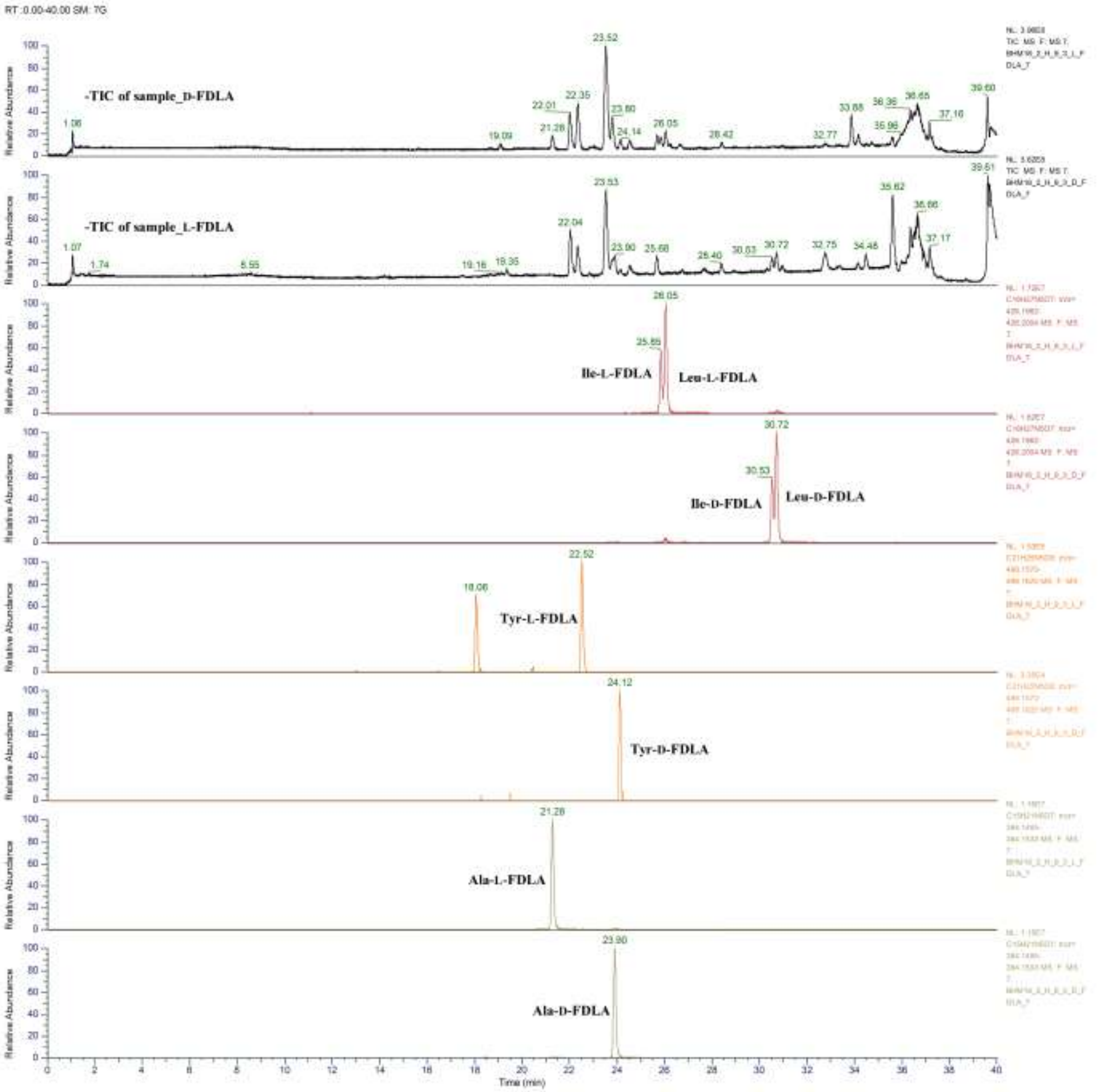

Figure S133. Advanced Marfey's analysis of compound **11**

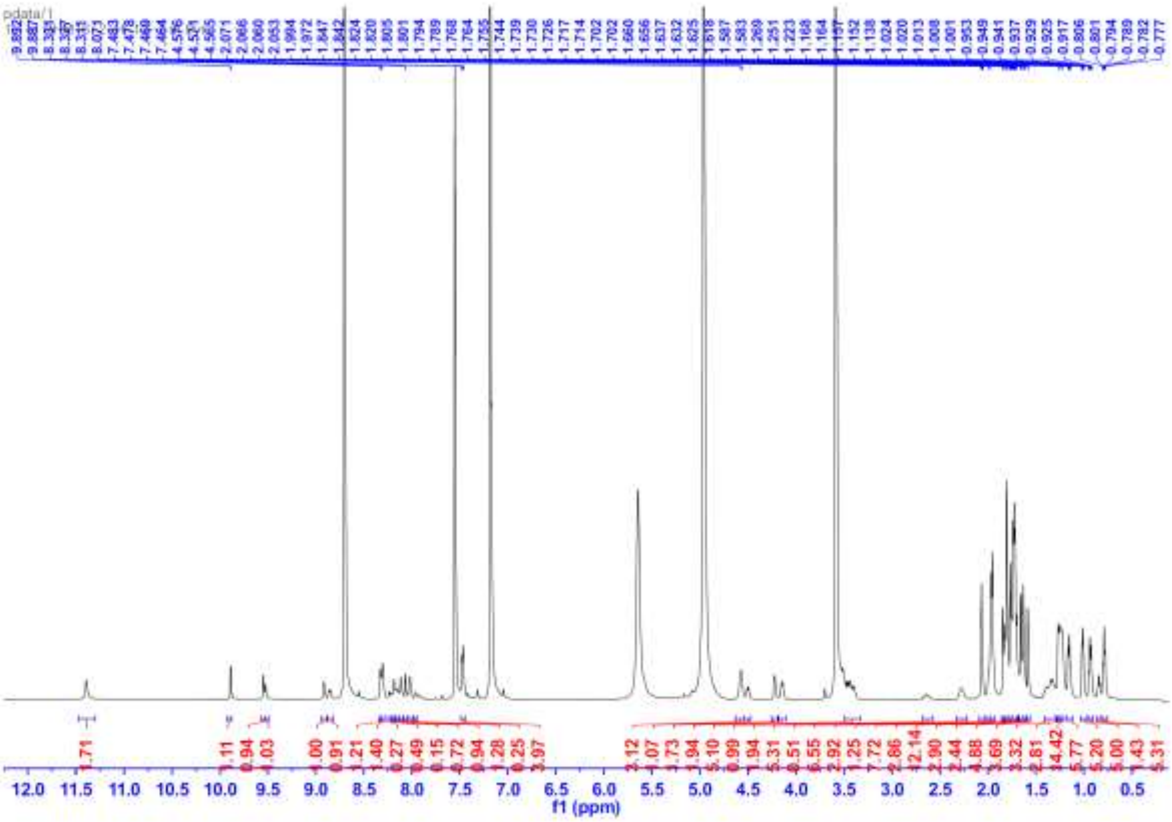

Figure S134. ^{1}H-NMR spectrum of compound **12** (Pyridine-d_5, 600 MHz)

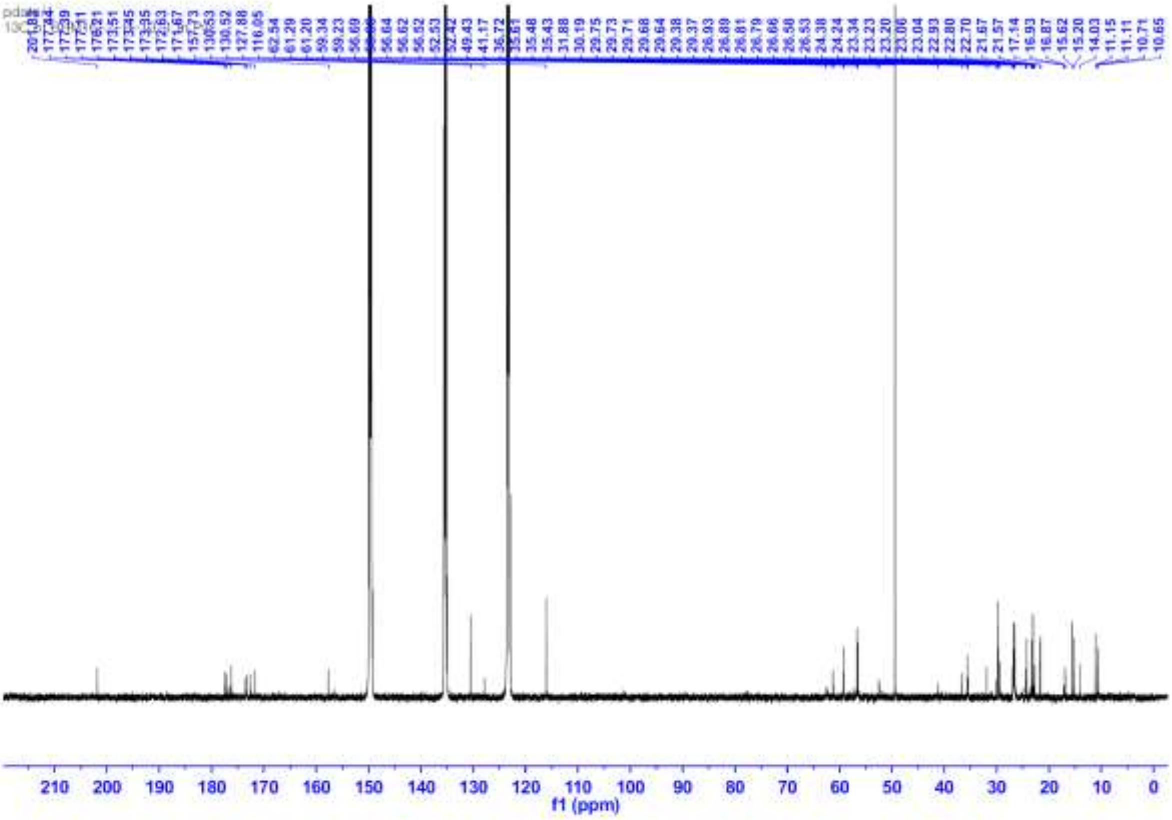

Figure S135. ^{13}C-NMR spectrum of compound **12** (Pyridine-d_5, 150 MHz)

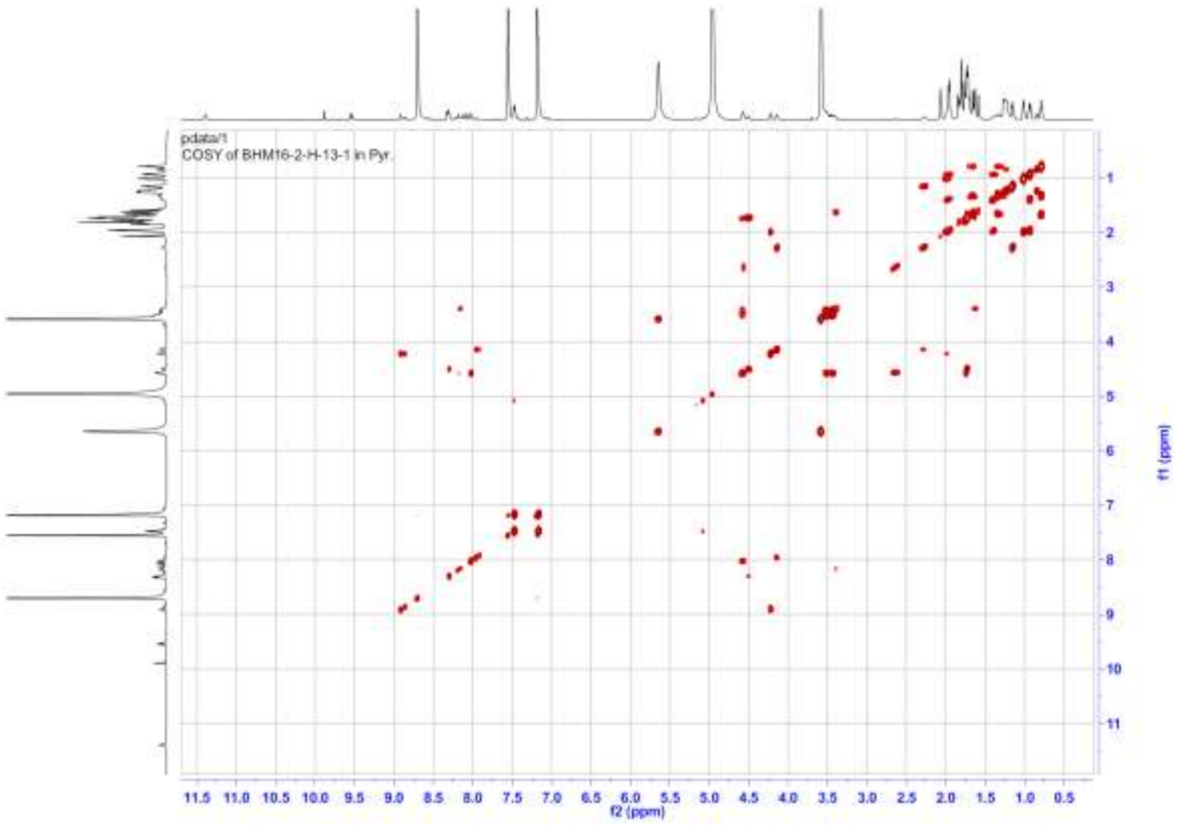

Figure S136. COSY spectrum of compound **12**

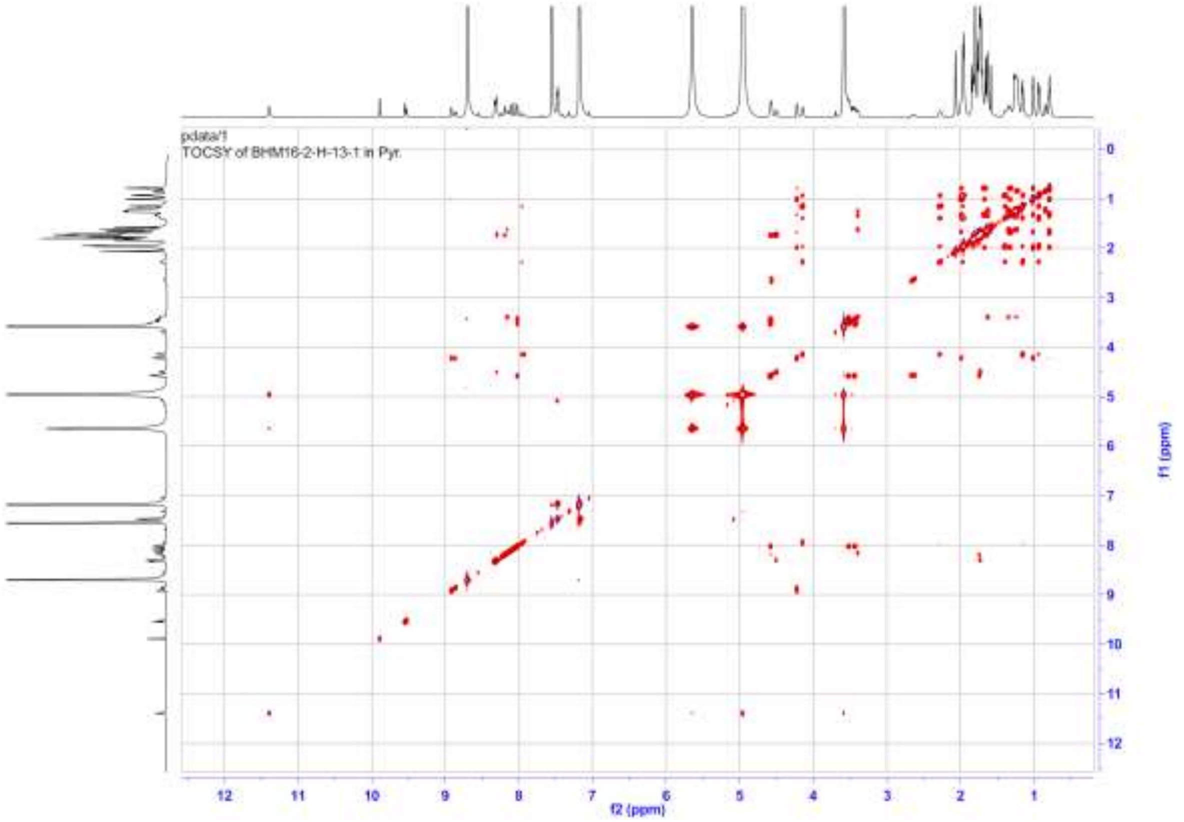

Figure S137. TOCSY spectrum of compound **12**

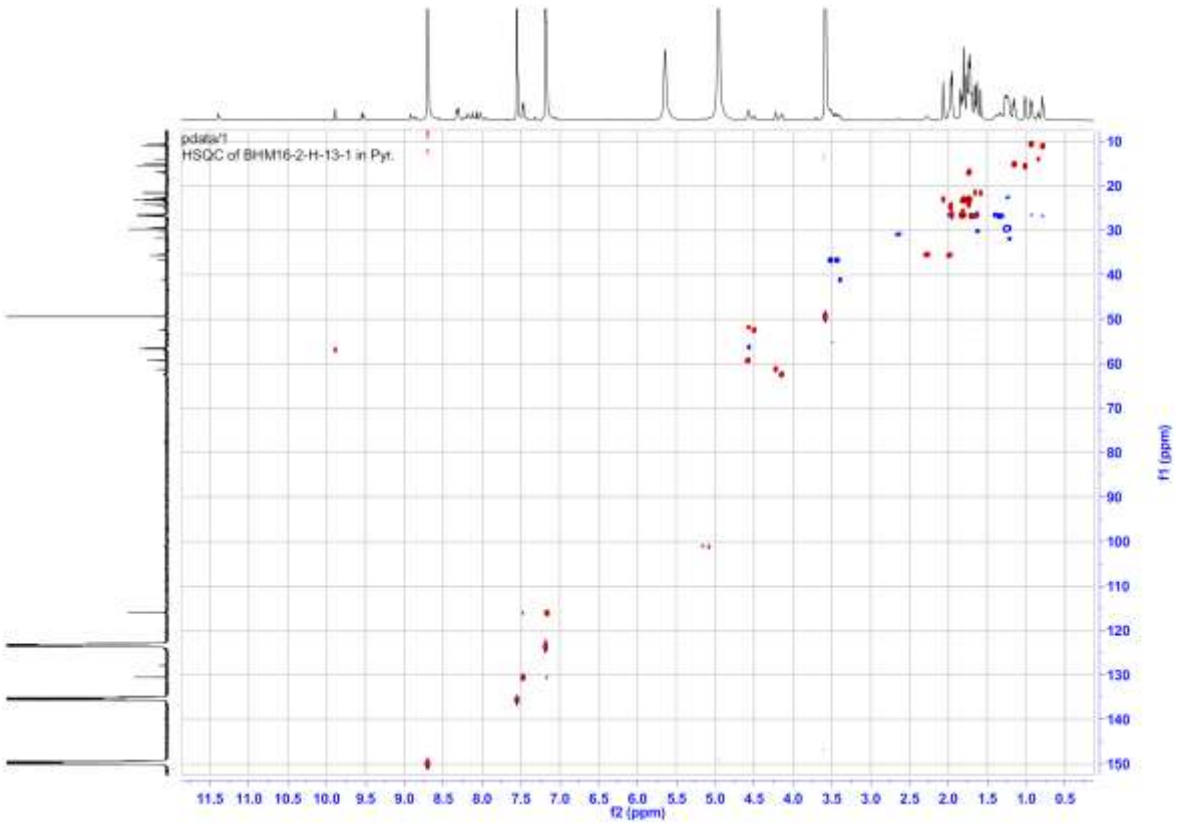

Figure S138. HSQC-DEPT spectrum of compound **12**

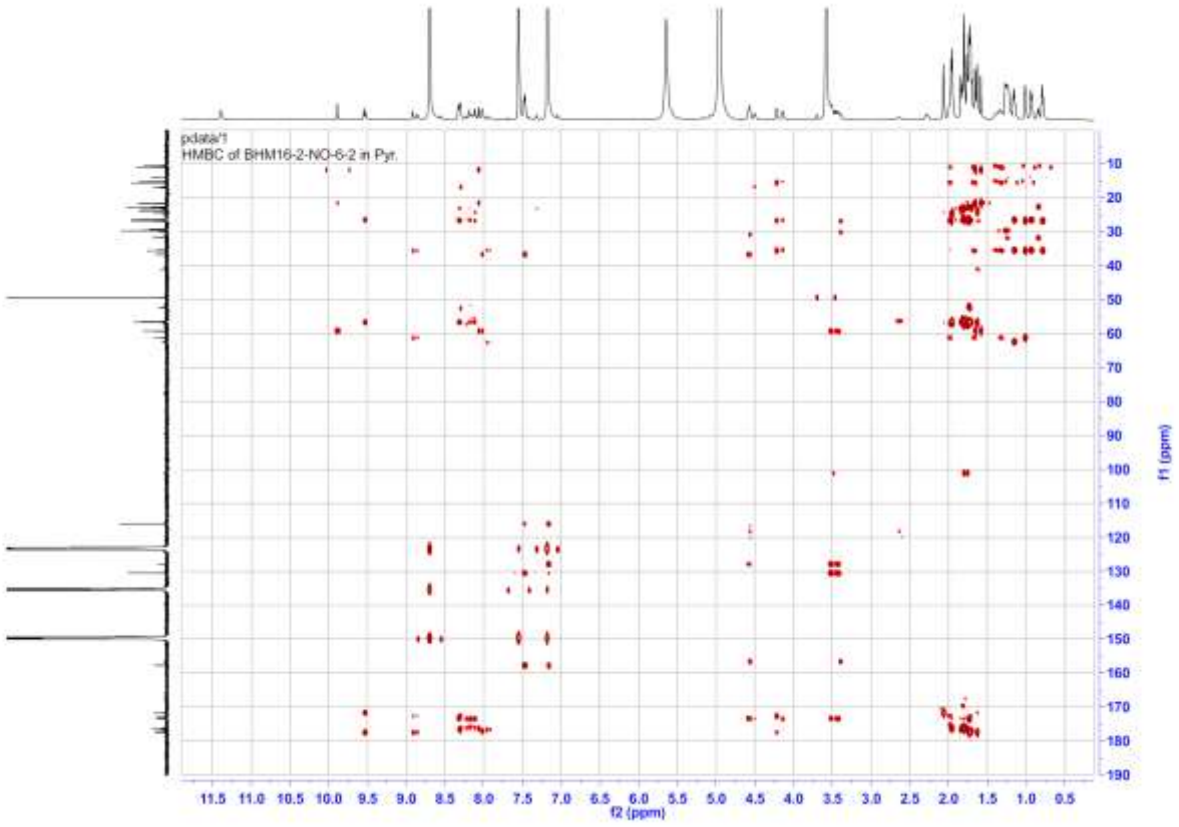

Figure S139. HMBC spectrum of compound **12**

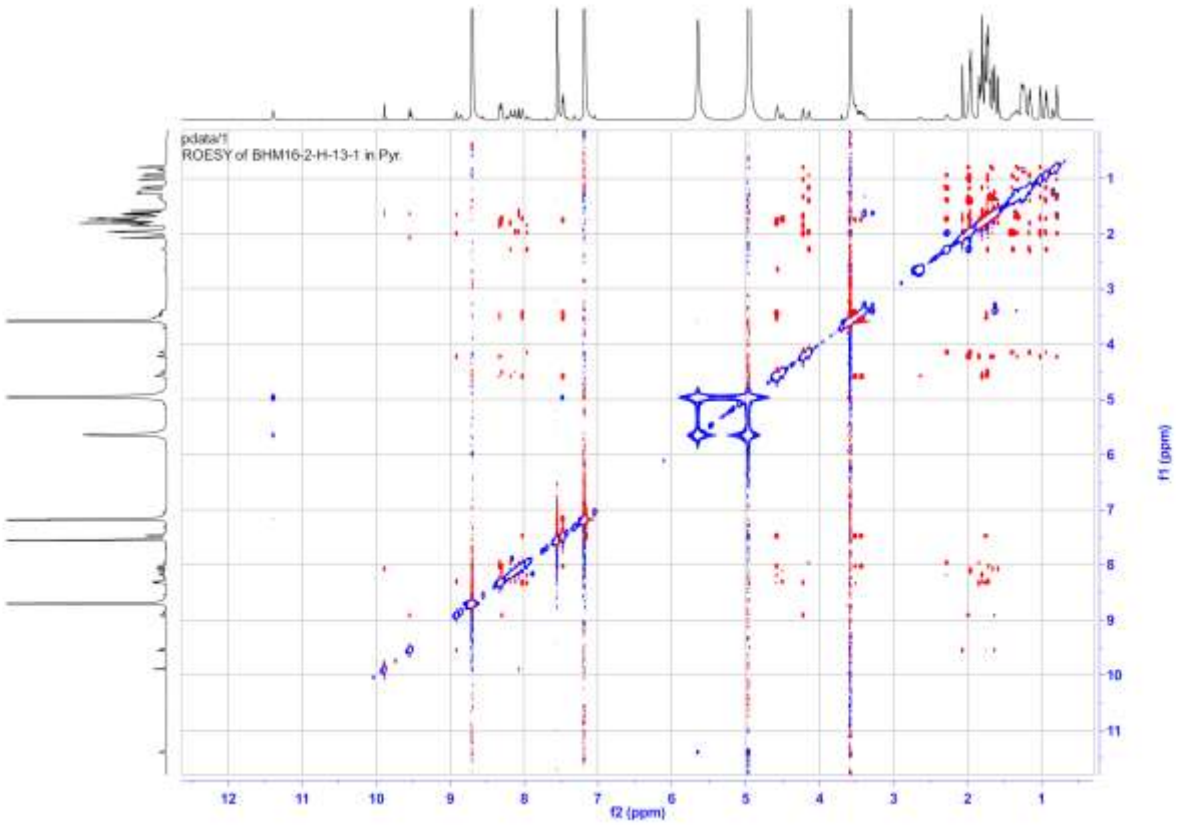

Figure S140. ROESY spectrum of compound **12**

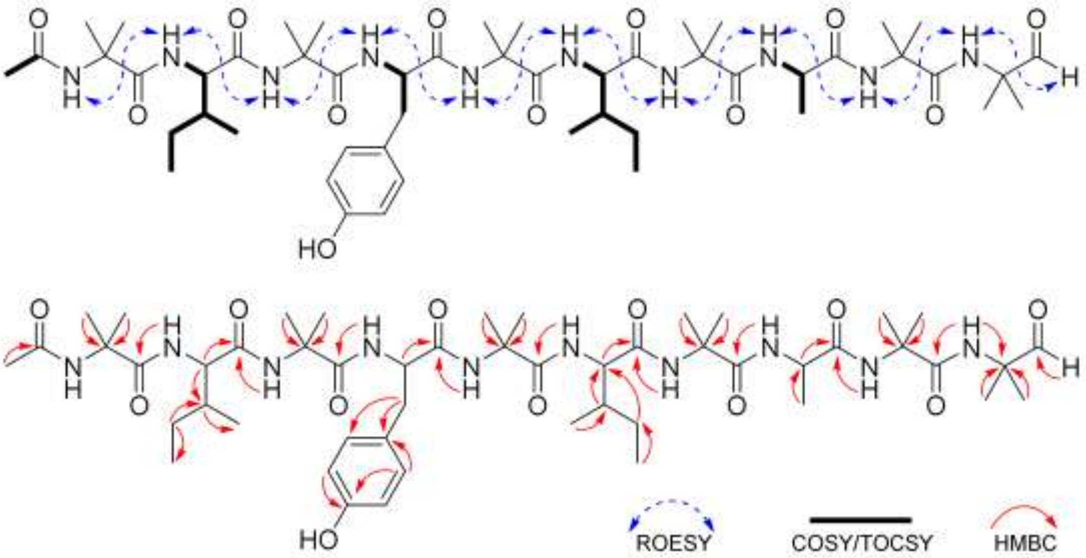

Figure S141. Key ROESY, COSY/TOCSY, and HMBC correlations of compound **12**

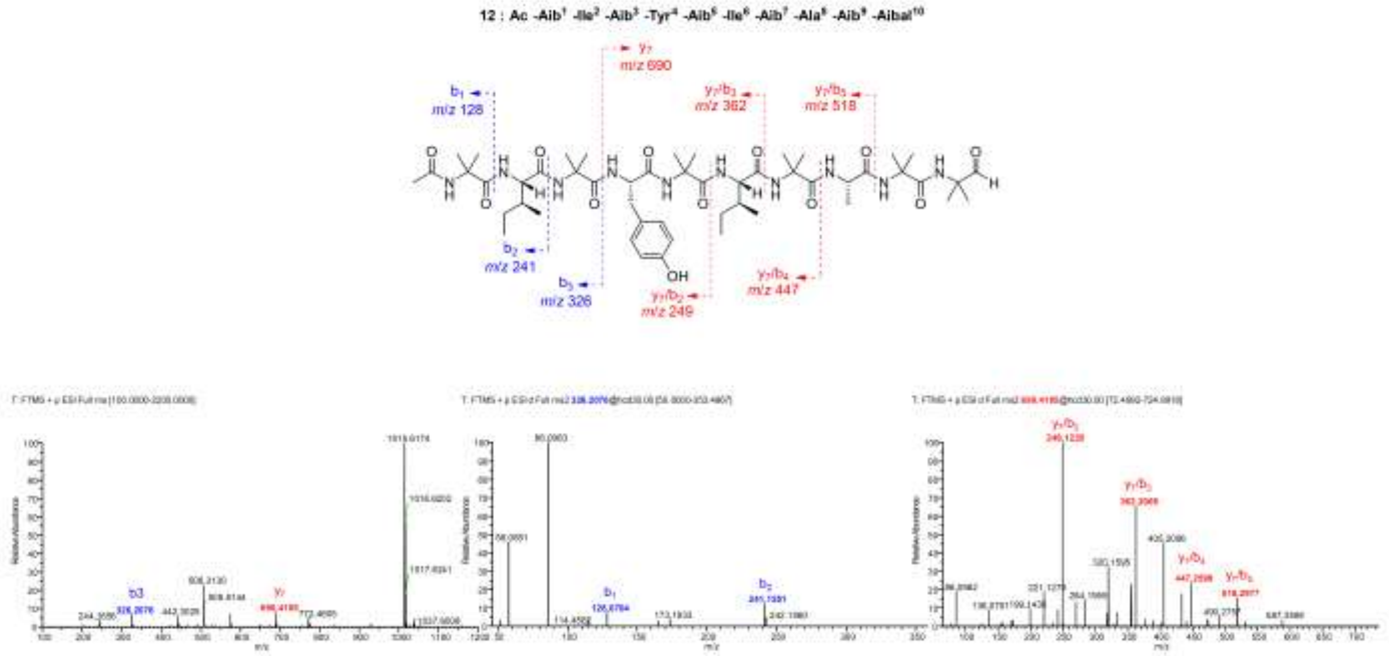

Figure S142. MS fragmentation analysis of compound **12**

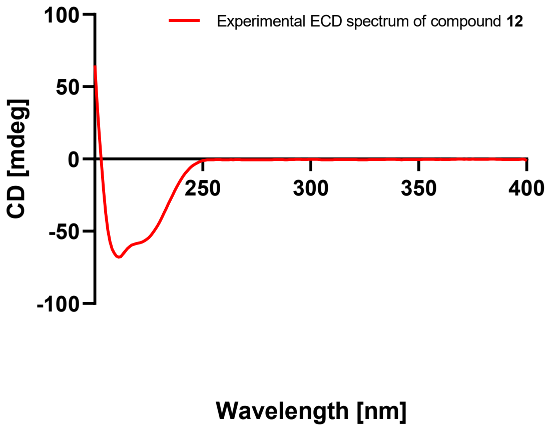

Figure S143. Experimental ECD spectrum of compound **12**

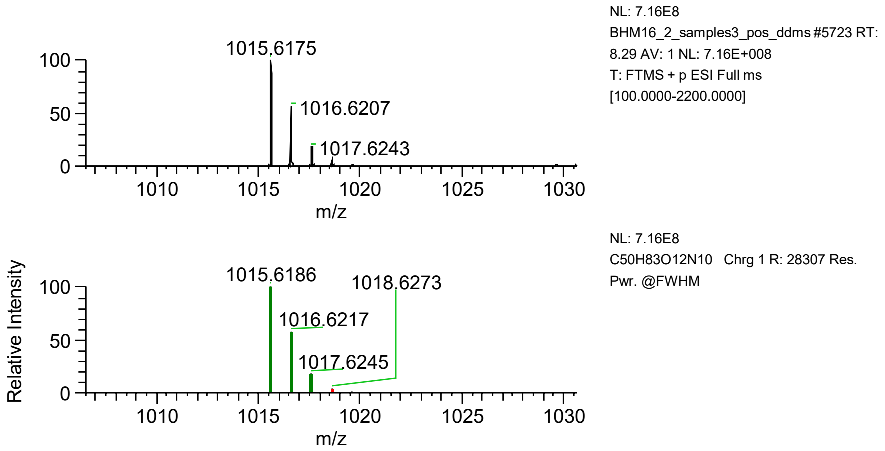

Figure S144. HRESIMS spectrum of compound **12**

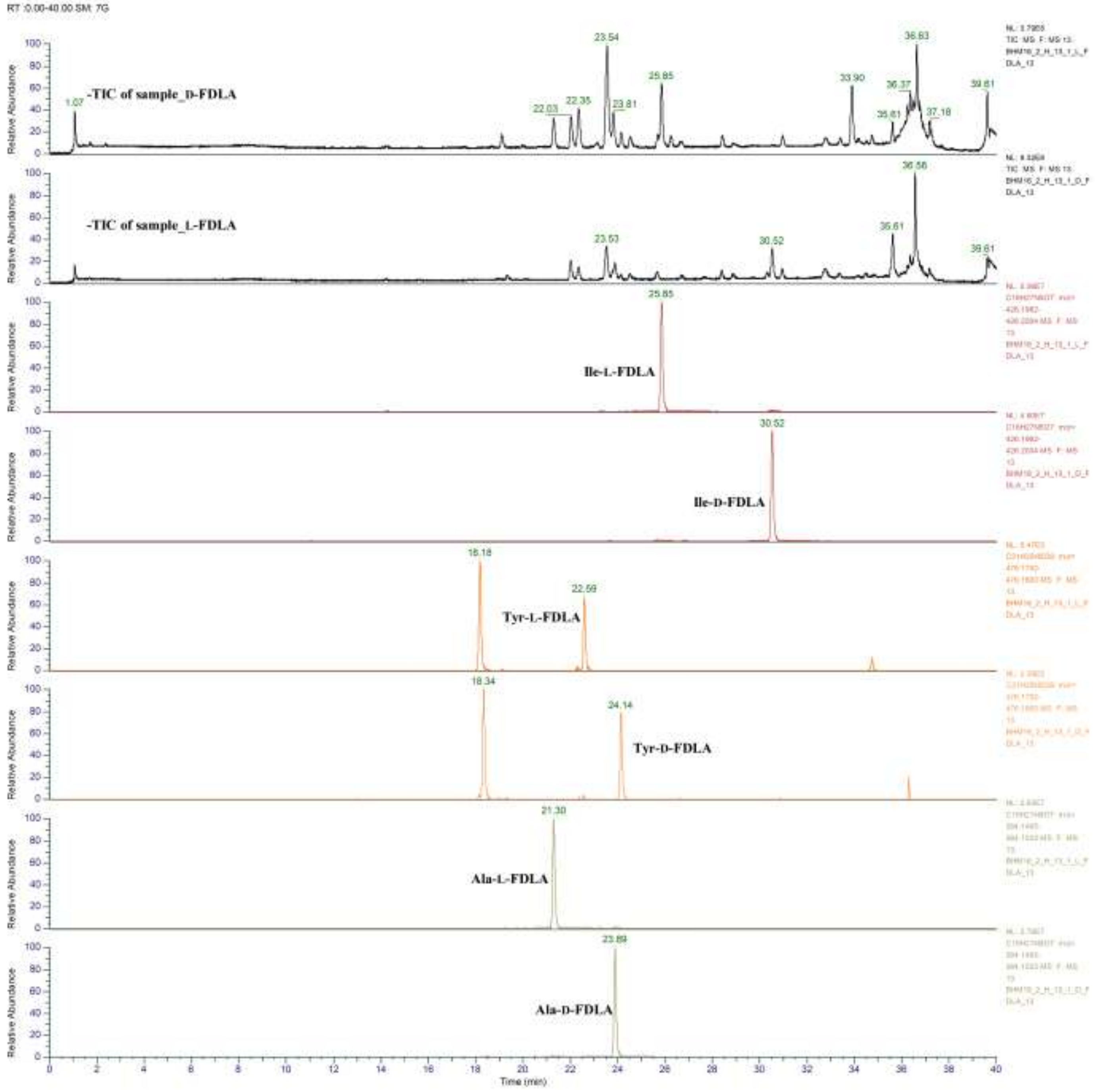

Figure S145. Advanced Marfey's analysis of compound **12**

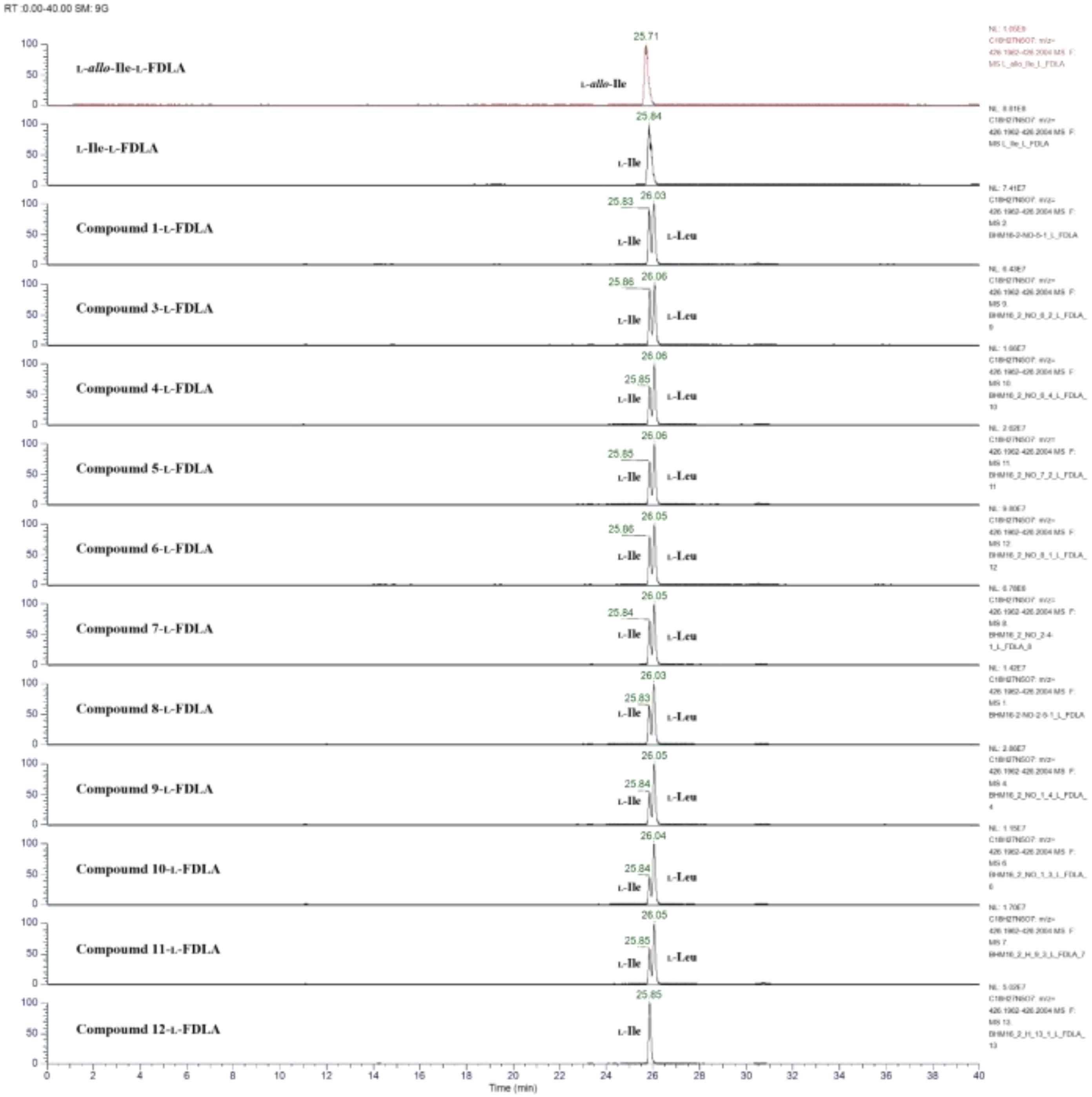

Figure S146. Advanced Marfey's analysis for L-Ile identification

Table S1. ^{1}H (600 MHz) data for compound **2** in pyridine-d_5 (δ in ppm, J in Hz)

	Position	δ_H		Position	δ_H
**Ac**	CH_3	2.03, s	**Leu¹¹**	N-H	8.18, d (8.2)
**Trp¹**	N-H	9.43, d (4.6)		α	4.93, m
	α	5.02, m		β	2.00, m; 2.23, m
	β	3.62, m; 3.70, m		γ	2.16, m
	1	11.95, d (2.5)		δ_1	0.99, d (4.1)
	2	7.42, m		δ_2	0.98, d (4.1)
	4	7.83, d (7,8)	**Aib¹²**	N-H	8.50, s
	5	7.18, m		α-Me¹	1.77, s
	6	7.28, t (7.6)		α-Me²	1.70, s
	7	7.56, m	**Pro¹³**	α	4.57, m
**Gly²**	N-H	9.97, t (7.6)		β	1.74, m; 2.18, m
	α	4.03, m; 4.14, m		γ	1.75, m; 1.97, m
**Ala³**	N-H	8.48, d (5.6)		δ	3.99, m; 4.03, m
	α	4.50, m	**Val¹⁴**	N-H	8.11, d (3.3)
	β	1.54, d (7.3)		α	4.10, m
**Aib⁴**	N-H	8.50, s		β	2.58, m
	α-Me¹	1.79, s		γ_1	1.22, d (6.8)
	α-Me²	1.70, s		γ_2	1.15, d (6.7)
**Aib⁵**	N-H	8.24, s	**Aib¹⁵**	N-H	7.93, s
	α-Me¹	1.72, s		α-Me¹	1.81, s
	α-Me²	1.70, s		α-Me²	1.72, s
**Gln⁶**	N-H	8.30, d (4.7)	**Leu¹⁶**	N-H	7.89, d (5.3)
	α	4.36, m		α	4.55, m
	β	2.69, m; 2.79, m		β	1.91, m; 2.01, m
	γ	2.77, m; 2.89, m		γ	2.16, m
	CON<u>H</u>₂	7.74, s; 8.15, s		δ_1	0.96, d (6.6)
**Aib⁷**	N-H	8.40, s		δ_2	0.86, d (6.4)
	α-Me¹	1.74, s	**Gln¹⁷**	N-H	8.42, d (5.7)
	α-Me²	1.70, s		α	4.74, m
**Aib⁸**	N-H	8.06, s		β	2.71, m; 2.88, m
	α-Me¹	1.81, s		γ	2.76, m; 3.00, m
	α-Me²	1.73, s		CON<u>H</u>₂	7.57, s; 7.96, s
**Aib⁹**	N-H	8.36, s	**Gln¹⁸**	N-H	8.12, m
	α-Me¹	1.83, s		α	5.06, m
	α-Me²	1.81, s		β	2.78, m; 3.04, m
**Ser¹⁰**	N-H	8.46, m		γ	2.73, m; 2.91, m
	α	4.72, m		CON<u>H</u>₂	7.47, s; 8.07, s
	β	4.31, m; 4.53, m	**Valol¹⁹**	N-H	7.71, d (9.3)
				α	4.30, m
				β	2.36, m
				γ_1	1.06, d (6.8)
				γ_2	1.19, d (6.8)
				β′	4.05, m

Table S2. ^{13}C NMR (150 MHz) data for compound **2** in pyridine-d_5

	Position	δ_C		Position	δ_C		Position	δ_C
**Ac**	CH_3	22.7	**Aib⁷**	C=O	177.0	**Val¹⁴**	C=O	174.5
	C=O	172.4		α	56.5		α	63.6
**Trp¹**	C=O	174.8		α-Me¹	26.8		β	29.4
	α	57.0		α-Me²	22.6		γ_1	20.1
	β	27.9	**Aib⁸**	C=O	176.4		γ_2	19.1
	2	124.2		α	56.9	**Aib¹⁵**	C=O	177.4
	3	110.3		α-Me¹	26.7		α	56.7
	3a	128.1		α-Me²	23.0		α-Me¹	27.1
	4	118.7	**Aib⁹**	C=O	177.7		α-Me²	22.7
	5	119.1		α	56.9	**Leu¹⁶**	C=O	175.2
	6	121.8		α-Me¹	27.0		α	55.1
	7	112.0		α-Me²	23.0		β	39.8
	7a	137.4	**Ser¹⁰**	C=O	171.4		γ	24.7
**Gly²**	C=O	171.8		α	60.4		δ_1	22.9
	α	44.4		β	62.0		δ_2	21.1
**Ala³**	C=O	175.2	**Leu¹¹**	C=O	173.6	**Gln¹⁷**	C=O	173.2
	α	52.2		α	52.2		α	55.1
	β	16.4		β	40.2		β	27.4
**Aib⁴**	C=O	175.8		γ	25.1		γ	33.0
	α	56.9		δ_1	20.8		<u>C</u>ONH$_2$	174.6
	α-Me¹	26.4		δ_2	22.9	**Gln¹⁸**	C=O	172.5
	α-Me²	23.1	**Aib¹²**	C=O	175.8		α	55.2
**Aib⁵**	C=O	177.1		α	56.9		β	28.4
	α	56.5		α-Me¹	26.9		γ	32.9
	α-Me¹	26.6		α-Me²	23.0		<u>C</u>ONH$_2$	174.5
	α-Me²	22.6	**Pro¹³**	C=O	175.2	**Valol¹⁹**	α	57.2
**Gln⁶**	C=O	174.7		α	64.0		β	29.2
	α	57.4		β	28.9		γ_1	19.9
	β	27.1		γ	26.2		γ_2	19.0
	γ	32.5		δ	49.3		β′	63.0
	<u>C</u>ONH$_2$	174.5						

Table S3. ^1H (600 MHz) data for compound **3** in pyridine-d_5 (δ in ppm, J in Hz)

	Position	δ_H		Position	δ_H
**Ac**	CH_3	2.07, s	**Leu¹¹**	N-H	8.18, m
**Phe¹**	N-H	9.58, d (5.3)		α	4.93, m
	α	4.90, m		β	1.99, m; 2.23, m
	β	3.33, m; 3.43, m		γ	2.16, m
	2	7.34, m		δ_1	0.97, m
	3	7.27, m		δ_2	0.98, m
	4	7.23, m	**Aib¹²**	N-H	8.53, s
	5	7.27, m		α-Me¹	1.81, s
	6	7.34, m		α-Me²	1.79, s
			**Pro¹³**	α	4.57, m
**Gly²**	N-H	9.93, m		β	1.74, m; 2.17, m
	α	4.04, m; 4.27, m		γ	1.97, m
**Ala³**	N-H	8.66, m		δ	3.99, m; 4.07, m
	α	4.49, m	**Val¹⁴**	N-H	8.04, m
	β	1.56, d (7.2)		α	4.08, m
**Aib⁴**	N-H	8.24, s		β	2.59, m
	α-Me¹	1.74, s		γ_1	1.18, m
	α-Me²	1.72, s		γ_2	1.13, d (6.6)
**Aib⁵**	N-H	8.37, s	**Aib¹⁵**	N-H	7.93, s
	α-Me¹	1.82, s		α-Me¹	1.72, s
	α-Me²	1.81, s		α-Me²	1.71, s
**Gln⁶**	N-H	8.36, m	**Ile¹⁶**	N-H	7.74, m
	α	4.36, m		α	4.27, m
	β	2.68, m; 2.77, m		β	2.23, m
	γ	2.77, m; 2.87, m		γ_1	1.84, m
	CON<u>H₂</u>	7.76, s; 8.17, s		γ_2	1.07, d (6.7)
**Aib⁷**	N-H	8.42, s		δ	0.85, d (7.8)
	α-Me¹	1.79, s	**Gln¹⁷**	N-H	8.38, m
	α-Me²	1.72, s		α	4.68, m
**Aib⁸**	N-H	8.07, s		β	2.72, m; 3.01, m
	α-Me¹	1.81, s		γ	2.77, m; 2.87, m
	α-Me²	1.73, s		CON<u>H₂</u>	7.52, s; 8.11, s
**Aib⁹**	N-H	8.38, s	**Gln¹⁸**	N-H	8.18, m
	α-Me¹	1.83, s		α	5.03, m
	α-Me²	1.79, s		β	2.68, m; 2.80, m
**Ser¹⁰**	N-H	8.45, m		γ	2.73, m; 2.98, m
	α	4.70, m		CON_	7.62, s; 8.03, s
	β	4.37, m; 4.53, m	**Valol¹⁹**	N-H	7.76, m
				α	4.27, m
				β	2.36, m
				γ_1	1.04, d (6.7)
				γ_2	1.18, m
				β′	4.04, m; 4.07, m

Table S4. ^{13}C (150 MHz) data for compound **3** in pyridine-d_5

	Position	δ_C		Position	δ_C		Position	δ_C
**Ac**	CH_3	22.7	**Aib⁷**	C=O	177.0	**Val¹⁴**	C=O	174.0
	C=O	172.2		α	56.7		α	63.5
**Phe¹**	C=O	174.3		α-Me¹	26.8		β	29.4
	α	57.3		α-Me²	22.6		γ_1	19.2
	β	37.4	**Aib⁸**	C=O	177.2		γ_2	19.1
	1	137.6		α	56.9	**Aib¹⁵**	C=O	177.2
	2	129.3		α-Me¹	26.9		α	56.7
	3	128.6		α-Me²	23.0		α-Me¹	26.3
	4	126.9	**Aib⁹**	C=O	177.7		α-Me²	23.1
	5	129.3		α	56.7	**Ile¹⁶**	N-H	174.3
	6	128.6		α-Me¹	27.0		α	61.6
				α-Me²	23.3		β	35.9
			**Ser¹⁰**	C=O	171.4		γ_1	26.8
**Gly²**	C=O	171.8		α	60.3		γ_2	15.7
	α	44.2		β	62.0		δ	11.1
**Ala³**	C=O	175.4	**Leu¹¹**	C=O	173.6	**Gln¹⁷**	C=O	173.5
	α	52.3		α	52.3		α	56.2
	β	16.5		β	40.2		β	28.3
**Aib⁴**	C=O	177.2		γ	24.7		γ	32.8
	α	56.5		δ_1	23.1		<u>C</u>ONH₂	174.6
	α-Me¹	26.8		δ_2	20.8	**Gln¹⁸**	C=O	172.8
	α-Me²	22.7	**Aib¹²**	C=O	174.9		α	55.4
**Aib⁵**	C=O	177.0		α	57.0		β	27.1
	α	56.9		α-Me¹	26.8		γ	32.9
	α-Me¹	27.0		α-Me²	23.3		<u>C</u>ONH₂	174.6
	α-Me²	23.3	**Pro¹³**	C=O	175.0	**Valol¹⁹**	α	57.5
**Gln⁶**	C=O	174.8		α	63.9		β	29.2
	α	57.5		β	29.0		γ_1	19.9
	β	27.1		γ	26.2		γ_2	19.1
	γ	32.6		δ	49.3		β′	63.0
	<u>C</u>ONH₂	174.6						

Table S5. ^1H (800 MHz) data for compound **4** in pyridine-d_5 (δ in ppm, *J* in Hz)

	Position	δ_H		Position	δ_H
**Ac**	CH_3	2.04, s	**Leu[11]**	N-H	8.13 d, (8.2)
**Trp[1]**	N-H	9.47, d (4.7)		α	4.85, m
	α	5.03, m		β	2.01, m; 2.18, m
	β	3.62, m; 3.69, m		γ	2.07, m
	1	11.95, d (2.4)		δ_1	0.96, d (6.4)
	2	7.44, m		δ_2	0.97, d (6.4)
	4	7.83, d (7.9)	**Aib[12]**	N-H	8.49, s
	5	7.18, m		α-Me[1]	1.78, s
	6	7.28, dt (6.2, 1.2)		α-Me[2]	1.76, s
	7	7.58, m	**Pro[13]**	α	4.56, m
**Gly[2]**	N-H	10.01, t (5.6)		β	1.75, m; 2.17, m
	α	4.05, m; 4.16, m		γ	1.75, m; 1.98, m
**Ala[3]**	N-H	8.54, d (5.8)		δ	3.97, m; 4.01, m
	α	4.51, m	**Val[14]**	N-H	8.04, d (6.7)
	β	1.54, d (7.3)		α	4.09, m
**Aib[4]**	N-H	8.57, s		β	2.57, m
	α-Me[1]	1.70, s		γ_1	1.20, d (6.7)
	α-Me[2]	1.70, s		γ_2	1.14, d (6.8)
**Aib[5]**	N-H	8.25, s	**Aib[15]**	N-H	7.95, s
	α-Me[1]	1.73, s		α-Me[1]	1.70, s
	α-Me[2]	1.72, s		α-Me[2]	1.70, s
**Gln[6]**	N-H	8.30, d (4.9)	**Ile[16]**	N-H	7.73, d (5.3)
	α	4.35, m		α	4.27, m
	β	2.72, m; 2.78, m		β	2.22, m
	γ	2.78, m; 2.93, m		γ_1	1.40, m
	CON<u>H</u>$_2$	7.72, s; 8.16, s		γ_2	1.06, d (6.8)
**Aib[7]**	N-H	8.34, s		δ	0.85, m
	α-Me[1]	1.76, s	**Gln[17]**	N-H	8.38, d (5.5)
	α-Me[2]	1.75, s		α	4.68, m
**Aib[8]**	N-H	7.98, s		β	2.81, m; 2.89, m
	α-Me[1]	1.79, s		γ	2.80, m; 2.97, m
	α-Me[2]	1.71, s		CON<u>H</u>$_2$	7.58, s; 8.01, s
**Aib[9]**	N-H	8.28, s	**Gln[18]**	N-H	8.16, m
	α-Me[1]	1.80, s		α	5.03, m
	α-Me[2]	1.80, s		β	2.77, m; 3.02, m
**Ala[10]**	N-H	8.30, m		γ	2.72, m; 2.97, m
	α	4.51, m		CON<u>H</u>$_2$	7.47, s; 8.08, s
	β	1.76, m	**Valol[19]**	N-H	7.75, d (9.3)
				α	4.27, m
				β	2.37, m
				γ_1	1.20, m
				γ_2	1.14, d (6.8)
				β′	4.03, m; 4.08, m

Table S6. ^{13}C (200 MHz) data for compound **4** in pyridine-d_5.

	Position	δ_C		Position	δ_C		Position	δ_C
**Ac**	CH_3	22.7	**Aib⁷**	C=O	176.2	**Val¹⁴**	C=O	174.2
	C=O	172.4		α	56.6		A	63.4
**Trp¹**	C=O	175.0		α-Me¹	27.0		β	29.4
	α	57.0		α-Me²	23.0		γ_1	19.9
	β	27.9	**Aib⁸**	C=O	176.5		γ_2	19.1
	2	124.0		α	56.6	**Aib¹⁵**	C=O	177.1
	3	110.3		α-Me¹	26.3		α	56.6
	3a	128.1		α-Me²	23.1		α-Me¹	27.2
	4	118.7	**Aib⁹**	C=O	177.1		α-Me²	22.7
	5	119.1		α	56.6	**Ile¹⁶**	N-H	174.4
	6	121.8		α-Me¹	27.2		α	61.5
	7	111.9		α-Me²	23.1		β	35.9
	7a	137.4	**Ala¹⁰**	C=O	174.4		γ_1	26.5
**Gly²**	C=O	171.8		α	52.4		γ_2	15.7
	α	44.3		β	16.9		δ	11.1
**Ala³**	C=O	175.0	**Leu¹¹**	C=O	173.6	**Gln¹⁷**	C=O	173.6
	α	52.3		α	52.2		α	56.3
	β	16.4		β	40.2		β	27.4
**Aib⁴**	C=O	175.8		γ	24.8		γ	32.6
	α	56.7		δ_1	20.8		<u>C</u>ONH₂	174.5
	α-Me¹	26.3		δ_2	22.9	**Gln¹⁸**	C=O	172.8
	α-Me²	22.7	**Aib¹²**	C=O	174.8		α	55.4
**Aib⁵**	C=O	177.1		α	57.0		β	28.3
	α	56.6		α-Me¹	27.1		γ	32.9
	α-Me¹	27.0		α-Me²	23.2		<u>C</u>ONH₂	174.5
	α-Me²	23.2	**Pro¹³**	C=O	175.0	**Valol¹⁹**	α	57.4
**Gln⁶**	C=O	174.6		α	63.9		β	29.3
	α	57.4		β	29.0		γ_1	19.9
	β	27.1		γ	26.2		γ_2	19.1
	γ	32.6		δ	49.4		β′	63.0
	<u>C</u>ONH₂	174.6						

Table S7. ^{1}H (600 MHz) data for compound **5** in pyridine-d_5 (δ in ppm, J in Hz)

	Position	δ_H		Position	δ_H
**Ac**	CH_3	2.12, s	**Leu^{11}**	N-H	8.18, m
**Trp^1**	N-H	9.51, m		α	4.91, m
	α	5.00, m		β	1.99, m; 2.22, m
	β	3.69, m		γ	2.14, m
	1	11.92, m		δ_1	0.97, d (6.3)
	2	7.55, m		δ_2	0.96, d (6.3)
	4	7.86, d (7.8)	**Aib^{12}**	N-H	8.51, s
	5	7.18, m		α-Me^1	1.79, s
	6	7.28, m		α-Me^2	1.78, s
	7	7.54, m	**Pro^{13}**	α	4.56, m
**Gly^2**	N-H	9.96, m		β	1.73, m; 2.18, m
	α	4.09, m; 4.20, m		γ	1.95, m
**Ala^3**	N-H	8.49, m		δ	3.98, m; 4.05, m
	α	4.47, m	**Val^{14}**	N-H	8.03, m
	β	1.55, d (7.2)		α	4.09, m
**Aib^4**	N-H	8.59, s		β	2.59, m
	α-Me^1	1.75, s		γ_1	1.21, d (6.5)
	α-Me^2	1.66, s		γ_2	1.16, d (6.4)
**Aib^5**	N-H	8.20, s	**Aib^{15}**	N-H	7.93, s
	α-Me^1	1.75, s		α-Me^1	1.74, s
	α-Me^2	1.72, s		α-Me^2	1.72, s
**Gln^6**	N-H	8.32, m	**Ile^{16}**	N-H	7.75, m
	α	4.36, m		α	4.26, m
	β	2.69, m; 2.78, m		β	2.23, m
	γ	2.77, m; 2.89, m		γ_1	1.39, m; 1.85, m
	CON$\underline{H_2}$	7.72, s; 8.13, s		γ_2	1.08, d (6.6)
**Aib^7**	N-H	8.47, s		δ	0.85, t (7.3)
	α-Me^1	1.75, s	**Glu^{17}**	N-H	8.49, m
	α-Me^2	1.71, s		α	4.74, m
**Aib^8**	N-H	8.08, s		β	2.79, m; 2.88, m
	α-Me^1	1.84, s		γ	2.74, m; 2.76, m
	α-Me^2	1.72, s		COOH	-
**Aib^9**	N-H	8.40 s	**Gln^{18}**	N-H	8.20, m
	α-Me^1	1.83, s		α	5.07, m
	α-Me^2	1.78, s		β	2.75, m; 2.83, m
**Ser^{10}**	N-H	8.45, m		γ	2.75, m; 2.99, m
	α	4.70, m		CON$\underline{H_2}$	7.58, s; 8.05, s
	β	4.36, m; 4.52, m	**Valol^{19}**	N-H	7.78, m
				α	4.26, m
				β	2.36, m
				γ_1	1.19, d (6.7)
				γ_2	1.04, d (6.7)
				β'	4.03, m; 4.11, m

Table S8. ^{13}C (150 MHz) data for compound **5** in pyridine-d_5

	Position	δC		Position	δC		Position	δC
**Ac**	CH_3	22.8	**Aib⁷**	C=O	177.0	**Val¹⁴**	C=O	174.1
	C=O	172.7		α	56.8		α	63.5
**Trp¹**	C=O	175.1		α-Me¹	27.0		β	29.3
	α	57.3		α-Me²	23.3		γ_1	20.0
	β	27.9	**Aib⁸**	C=O	177.1		γ_2	19.1
	2	124.4		α	56.9	**Aib¹⁵**	C=O	177.2
	3	110.4		α-Me¹	26.9		α	56.7
	3a	128.1		α-Me²	22.7		α-Me¹	26.9
	4	118.7	**Aib⁹**	C=O	177.8		α-Me²	22.6
	5	119.1		α	57.4	**Ile¹⁶**	C=O	174.5
	6	121.7		α-Me¹	26.8		α	61.7
	7	112.0		α-Me²	22.7		β	35.9
	7a	137.4	**Ser¹⁰**	C=O	171.5		γ_1	26.6
**Gly²**	C=O	171.9		α	60.4		γ_2	15.7
	α	44.5		β	61.9		δ	11.0
**Ala³**	C=O	174.9	**Leu¹¹**	C=O	173.6	**Glu¹⁷**	C=O	173.6
	α	52.5		α	52.3		α	56.3
	β	16.3		β	40.2		β	27.1
**Aib⁴**	C=O	175.8		γ	24.6		γ	32.5
	α	56.6		δ_1	20.8		<u>C</u>OOH	174.6
	α-Me¹	26.8		δ_2	23.1	**Gln¹⁸**	C=O	173.1
	α-Me²	22.6	**Aib¹²**	C=O	174.8		α	55.6
**Aib⁵**	C=O	177.1		α	56.9		β	27.1
	α	56.9		α-Me¹	26.8		γ	32.8
	α-Me¹	27.1		α-Me²	23.0		<u>C</u>ONH₂	174.6
	α-Me²	23.2	**Pro¹³**	C=O	175.1	**Valol¹⁹**	α	57.6
**Gln⁶**	C=O	174.9		α	64.0		β	29.2
	α	57.4		β	29.0		γ_1	19.2
	β	27.1		γ	26.3		γ_2	19.9
	γ	32.5		δ	49.3		β′	63.5
	<u>C</u>ONH₂	174.6						

Table S9. ^{1}H (600 MHz) data for compound **6** in pyridine-d_5 (δ in ppm, J in Hz)

	Position	δ_H		Position	δ_H
**Ac**	CH_3	2.09, s	**Leu**[11]	N-H	8.18, m
**Phe**[1]	N-H	9.60, m		α	4.93, m
	α	4.89, m		β	2.00, m; 2.22, m
	β	3.36, m; 3.44, m		γ	2.15, m
	2	7.38, d (7.5)		δ_1	0.97, d (6.3)
	3	7.28, m		δ_2	0.97, d (6.3)
	4	7.22, m	**Aib**[12]	N-H	8.49, s
	5	7.38, d (7.5)		α-Me[1]	1.80, s
	6	7.28, m		α-Me[2]	1.79, s
			**Pro**[13]	α	4.56, m
**Gly**[2]	N-H	9.94, m		β	1.74, m; 2.20, m
	α	4.10, m; 4.25, m		γ	1.74, m; 1.96, m
**Ala**[3]	N-H	8.63, m		δ	3.99, m; 4.04, m
	α	4.51, m	**Val**[14]	N-H	8.04, m
	β	1.57, d (7.2)		α	4.10, m
**Aib**[4]	N-H	8.28, s		β	2.60, m
	α-Me[1]	1.82, s		γ_1	1.21, d (6.6)
	α-Me[2]	1.79, s		γ_2	1.17, d (6.6)
**Aib**[5]	N-H	8.43, s	**Aib**[15]	N-H	7.94, s
	α-Me[1]	1.74, s		α-Me[1]	1.74, s
	α-Me[2]	1.72, s		α-Me[2]	1.73, s
**Gln**[6]	N-H	8.36, m	**Ile**[16]	N-H	7.76, m
	α	4.36, m		α	4.25, m
	β	2.67, m; 2.77, m		β	2.24, m
	γ	2.77, m; 2.87, m		γ_1	1.40, m; 1.86, m
	CON$\underline{H_2}$	7.76, s; 8.18, s		γ_2	1.09, d (6.6)
**Aib**[7]	N-H	8.42, s		δ	0.85, t (7.4)
	α-Me[1]	1.74, s	**Glu**[17]	N-H	8.50, m
	α-Me[2]	1.72, s		α	4.75, m
**Aib**[8]	N-H	8.08, s		β	2.81, m; 2.88, m
	α-Me[1]	1.82, s		γ	2.76, m; 2.85, m
	α-Me[2]	1.73, s		COOH	-
**Aib**[9]	N-H	8.38, s	**Gln**[18]	N-H	8.20, m
	α-Me[1]	1.83, s		α	5.06, m
	α-Me[2]	1.80, s		β	2.75, m; 2.83, m
**Ser**[10]	N-H	8.44, m		γ	2.75, m; 2.99, m
	α	4.72, m		$CONH_2$	7.56, s; 8.05, s
	β	4.36, m; 4.52, m	**Valol**[19]	N-H	7.77, m
				α	4.27, m
				β	2.36, m
				γ_1	1.05, d (6.7)
				γ_2	1.19, d (6.6)
				β′	4.03, m; 4.10, m

Table S10. ^{13}C (150 MHz) data for compound **6** in pyridine-d_5

	Position	δ_C		Position	δ_C		Position	δ_C
**Ac**	CH_3	22.7	**Aib⁷**	C=O	177.0	**Val¹⁴**	C=O	174.1
	C=O	172.3		α	56.7		α	63.4
**Phe¹**	C=O	174.4		α-Me¹	26.6		β	29.3
	α	57.5		α-Me²	22.7		γ_1	20.0
	β	37.4	**Aib⁸**	C=O	176.4		γ_2	19.1
	1	137.7		α	56.9	**Aib¹⁵**	C=O	177.3
	2	129.4		α-Me¹	27.0		α	56.7
	3	128.6		α-Me²	23.0		α-Me¹	26.9
	4	126.9	**Aib⁹**	C=O	177.8		α-Me²	23.3
	5	129.4		α	57.5	**Ile¹⁶**	N-H	174.7
	6	128.6		α-Me¹	27.1		α	61.7
				α-Me²	23.2		β	35.9
			**Ser¹⁰**	C=O	171.5		γ_1	26.6
**Gly²**	C=O	171.8		α	60.3		γ_2	15.7
	α	44.3		β	62.0		δ	11.0
**Ala³**	C=O	175.3	**Leu¹¹**	C=O	173.6	**Glu¹⁷**	C=O	173.6
	α	52.3		α	52.2		α	56.3
	β	16.5		β	40.2		β	27.1
**Aib⁴**	C=O	177.0		γ	24.6		γ	32.6
	α	57.5		δ_1	20.8		<u>C</u>OOH	174.7
	α-Me¹	27.0		δ_2	23.2	**Gln¹⁸**	C=O	173.0
	α-Me²	23.2	**Aib¹²**	C=O	174.9		α	55.5
**Aib⁵**	C=O	177.8		α	57.0		β	28.3
	α	56.7		α-Me¹	26.8		γ	32.9
	α-Me¹	26.9		α-Me²	23.3		<u>C</u>ONH₂	174.7
	α-Me²	22.7	**Pro¹³**	C=O	175.1	**Valol¹⁹**	α	57.5
**Gln⁶**	C=O	174.8		α	64.0		β	29.2
	α	57.4		β	29.0		γ_1	19.9
	β	27.1		γ	26.3		γ_2	19.2
	γ	32.6		δ	49.3		β′	62.9
	<u>C</u>ONH₂	174.7						

Table S11. ^{1}H (800 MHz) data for compound **8** in pyridine-d_5 (δ in ppm, J in Hz)

	Position	δ_H		Position	δ_H
**Ac**	CH_3	2.02, s	**Ser^{10}**	N-H	8.46, m
**Trp^1**	N-H	9.44, d (4.7)		α	4.71, m
	α	5.03, m		β	4.36, m; 4.54, m
	β	3.61, m; 3.68, m	**Leu^{11}**	N-H	8.17, m
	1	11.96, m		α	5.00, m
	2	7.41, d (2.3)		β	2.06, m; 2.24, m
	4	7.82, d (7.9)		γ	2.17, m
	5	7.18, m		δ_1	1.01, d (6.4)
	6	7.28, m		δ_2	1.00, d (6.4)
	7	7.56, m	**Aib^{12}**	N-H	8.44, s
**Gly^2**	N-H	10.00, m		α-Me^1	1.81, s
	α	4.04, m; 4.14, m		α-Me^2	1.78, s
**Ala^3**	N-H	8.52, m	**Pro^{13}**	α	4.63, m
	α	4.50, m		β	1.79, m; 2.23, m
	β	1.54, d (7.3)		γ	1.71, m; 1.94, m
**Aib^4**	N-H	8.53, s		δ	3.99, m; 4.02, m
	α-Me^1	1.70, s	**Val^{14}**	N-H	8.29, d (6.2)
	α-Me^2	1.66, s		α	4.22, m
**Aib^5**	N-H	8.23, s		β	2.61, m
	α-Me^1	1.69, s		γ_1	1.25, d (7.2)
	α-Me^2	1.67, s		γ_2	1.16, d (6.6)
**Gln^6**	N-H	8.31, d (4.8)	**Aib^{15}**	N-H	8.03, s
	α	4.36, d (8.1)		α-Me^1	1.87, s
	β	2.67, m; 2.78, m		α-Me^2	1.83, s
	γ	2.78, m; 2.88, m	**Ile^{16}**	N-H	7.51, d (8.5)
	CON$\underline{H}_2$	7.76, s; 8.18, s		α	4.89, m
**Aib^7**	N-H	8.39, s		β	2.48, m
	α-Me^1	1.74, s		γ_1	1.57, m; 1.94, m
	α-Me^2	1.70, s		γ_2	1.17, d (6.6)
**Aib^8**	N-H	8.06, s		δ	0.95, t (7.4)
	α-Me^1	1.81, s	**cycloGln^{17}**	N-H	7.96, d (8.2)
	α-Me^2	1.74, s		α	4.72, m
**Aib^9**	N-H	8.36, s		β	1.96, m; 2.73, m
	α-Me^1	1.84, s		γ	2.53, m; 2.59, m
	α-Me^2	1.81, s		β′	5.45, t (2.5)
				CON$\underline{H}$	9.14, d (4.2)

Table S12. ^{13}C (200 MHz) data for compound **8** in pyridine-d_5

	Position	δ_C		Position	δ_C		Position	δ_C
**Ac**	CH_3	22.6	**Gln⁶**	C=O	174.8	**Pro¹³**	C=O	174.8
	C=O	172.3		α	57.4		α	63.9
**Trp¹**	C=O	174.8		β	27.0		β	29.0
	α	56.9		γ	32.5		γ	25.9
	β	28.0		<u>C</u>ONH₂	174.5		δ	49.2
	2	124.1	**Aib⁷**	C=O	176.9	**Val¹⁴**	C=O	174.3
	3	110.3		α	56.6		α	62.5
	3a	128.1		α-Me¹	26.7		β	29.3
	4	118.7		α-Me²	22.6		γ1	19.9
	5	119.1	**Aib⁸**	C=O	176.5		γ2	19.2
	6	121.8		α	56.9	**Aib¹⁵**	C=O	175.0
	7	112.0		α-Me¹	26.8		α	57.4
	7a	137.4		α-Me²	23.4		α-Me¹	27.5
**Gly²**	C=O	171.7	**Aib⁹**	C=O	177.6		α-Me²	23.6
	α	44.3		α	56.9	**Ile¹⁶**	C=O	171.7
**Ala³**	C=O	175.1		α-Me¹	27.5		α	59.5
	α	52.1		α-Me²	23.4		β	36.6
	β	16.4	**Ser¹⁰**	C=O	171.3		γ1	25.6
**Aib⁴**	C=O	175.7		α	60.3		γ2	16.2
	α	56.7		β	62.0		δ	11.5
	α-Me¹	26.9	**Leu¹¹**	C=O	173.9	**cycGlu¹⁷**	α	49.0
	α-Me²	23.3		α	52.0		β	21.7
**Aib⁵**	C=O	177.0		β	40.3		γ	30.9
	α	56.8		γ	24.7		δ	171.1
	α-Me¹	26.4		δ1	20.8		β′	75.8
	α-Me²	22.6		δ2	23.3			
			**Aib¹²**	C=O	174.5			
				α	56.9			
				α-Me¹	27.0			
				α-Me²	23.4			

Table S13. ^{1}H (800 MHz) data for compound **9** in pyridine-d_5 (δ in ppm, *J* in Hz)

	Position	δ_H		Position	δ_H
**Ac**	CH_3	2.02, s	**Ser[10]**	N-H	8.45, m
**Trp[1]**	N-H	9.45, d (4.6)		α	4.72, m
	α	5.03, m		β	4.37, m; 4.54, m
	β	3.61, m; 3.68, m	**Leu[11]**	N-H	8.16, m
	1	11.96, d (2.4)		α	5.02, m
	2	7.41, d (2.2)		β	2.05, m; 2.24, m
	4	7.83, d (7.9)		γ	2.17, m
	5	7.18, m		δ_1	1.00, d (6.4)
	6	7.28, m		δ_2	1.00, d (6.4)
	7	7.55, m	**Aib[12]**	N-H	8.39, s
**Gly[2]**	N-H	10.02, m		α-Me[1]	1.80, s
	α	4.04, m; 4.14, m		α-Me[2]	1.74, s
**Ala[3]**	N-H	8.53, m	**Pro[13]**	α	4.60, m
	α	4.50, m		β	1.74, m; 2.20, m
	β	1.54, d (7.2)		γ	1.68, m; 1.89, m
**Aib[4]**	N-H	8.53, s		δ	3.95, m; 4.03, m
	α-Me[1]	1.70, s	**Val[14]**	N-H	8.28, d (5.9)
	α-Me[2]	1.66, s		α	4.22, m
**Aib[5]**	N-H	8.23, s		β	2.59, m
	α-Me[1]	1.69, s		γ_1	1.29, d (7.2)
	α-Me[2]	1.66, s		γ_2	1.19, d (6.9)
**Gln[6]**	N-H	8.31, d (4.6)	**Aib[15]**	N-H	7.92, s
	α	4.36, m		α-Me[1]	1.89, s
	β	2.68, m; 2.78, m		α-Me[2]	1.83, s
	γ	2.78, m; 2.88, m	**Ile[16]**	N-H	7.55, m
	CON<u>H</u>$_2$	7.75, s; 8.18, s		α	5.00, m
**Aib[7]**	N-H	8.39, s		β	2.57, m
	α-Me[1]	1.80, s		γ_1	1.63, m; 1.93, m
	α-Me[2]	1.71, s		γ_2	1.14, d (6.8)
**Aib[8]**	N-H	8.06, s		δ	0.96, t (7.4)
	α-Me[1]	1.80, s	**cycGln[17]**	N-H	8.20, d (7.7)
	α-Me[2]	1.71, s		α	4.71, m
**Aib[9]**	N-H	8.38, s		β	2.37, m; 2.71, m
	α-Me[1]	1.82, s		γ	2.49, m; 3.20, m
	α-Me[2]	1.71, s		β′	4.91, t (3.6)
				CON<u>H</u>	9.16, d (3.8)
				OCH_3	3.29, s

Table S14. ^{13}C (200 MHz) data for compound **9** in pyridine-d_5

	Position	δ_C		Position	δ_C		Position	δ_C
**Ac**	CH_3	22.6	**Gln⁶**	C=O	174.8	**Pro¹³**	C=O	174.7
	C=O	172.3		α	57.4		α	63.9
**Trp¹**	C=O	174.7		β	27.0		β	28.9
	α	56.9		γ	32.6		γ	26.3
	β	28.0		<u>C</u>ONH₂	174.5		δ	49.3
	2	124.2	**Aib⁷**	C=O	176.9	**Val¹⁴**	C=O	173.3
	3	110.2		α	56.9		α	62.5
	3a	128.1		α-Me¹	26.9		β	29.3
	4	118.7		α-Me²	23.5		γ1	19.7
	5	119.1	**Aib⁸**	C=O	176.4		γ2	19.2
	6	121.8		α	56.7	**Aib¹⁵**	C=O	175.1
	7	111.9		α-Me¹	26.8		α	57.1
	7a	137.4		α-Me²	23.5		α-Me¹	27.9
**Gly²**	C=O	171.9	**Aib⁹**	C=O	177.9		α-Me²	23.8
	α	44.3		α	56.9	**Ile¹⁶**	C=O	172.0
**Ala³**	C=O	175.1		α-Me¹	26.9		α	58.8
	α	52.2		α-Me²	22.2		β	36.9
	β	16.5	**Ser¹⁰**	C=O	171.3		γ1	25.3
**Aib⁴**	C=O	175.7		α	60.3		γ2	16.4
	α	56.7		β	62.0		δ	11.9
	α-Me¹	26.9	**Leu¹¹**	C=O	174.1	**cycGlu¹⁷**	α	46.5
	α-Me²	23.3		α	52.0		β	22.2
**Aib⁵**	C=O	177.1		β	40.3		γ	28.0
	α	56.6		γ	24.7		δ	171.3
	α-Me¹	26.3		δ1	20.8		β′	85.8
	α-Me²	23.2		δ2	23.3		OCH_3	54.5
			**Aib¹²**	C=O	174.7			
				α	56.8			
				α-Me¹	26.8			
				α-Me²	23.5			

Table S15. ^{1}H (900 MHz) data for compound **10** in pyridine-d_5 (δ in ppm, J in Hz)

	Position	δ_H		Position	δ_H
**Ac**	CH_3	2.02, s	**Ser^{10}**	N-H	8.43, d (4.6)
**Phe^1**	N-H	9.56, d (5.4)		α	4.71, m
	α	4.92, m		β	4.37, m; 4.53, m
	β	3.29, m; 3.42, m	**Leu^{11}**	N-H	8.15, d (8.3)
	2	7.30, d (7.1)		α	5.01, m
	3	7.26, m		β	2.05, m; 2.22, m
	4	7.27, m		γ	2.16, m
	5	7.30, d (7.1)		δ_1	0.99, d (6.6)
	6	7.26, m		δ_2	1.00, d (6.6)
			**Aib^{12}**	N-H	8.38, s
**Gly^2**	N-H	9.98, m		α-Me^1	1.80, s
	α	4.04, m; 4.23, m		α-Me^2	1.78, s
**Ala^3**	N-H	8.71, m	**Pro^{13}**	α	4.59, m
	α	4.51, m		β	1.74, m; 2.20, m
	β	1.54, d (7.3)		γ	1.66, m; 1.89, m
**Aib^4**	N-H	8.66, s		δ	3.94, m; 4.02, m
	α-Me^1	1.69, s	**Val^{14}**	N-H	8.29, d (6.0)
	α-Me^2	1.66, s		α	4.22, m
**Aib^5**	N-H	8.19, s		β	2.58, m
	α-Me^1	1.71, s		γ_1	1.28, d (6.8)
	α-Me^2	1.66, s		γ_2	1.19, d (6.8)
**Gln^6**	N-H	8.36, d (5.0)	**Aib^{15}**	N-H	7.91, s
	α	4.34, m		α-Me^1	1.89, s
	β	2.66, m; 2.76, m		α-Me^2	1.83, s
	γ	2.78, m; 2.88, m	**Ile^{16}**	N-H	7.54, m
	CON$\underline{H_2}$	7.78, s; 8.20, s		α	4.99, m
**Aib^7**	N-H	8.38, s		β	2.57, m
	α-Me^1	1.73, s		γ_1	1.62, m; 1.92, m
	α-Me^2	1.71, s		γ_2	1.14, d (6.9)
**Aib^8**	N-H	8.05, s		δ	0.95, t (7.4)
	α-Me^1	1.80, s	**cycGln^{17}**	N-H	8.18, m
	α-Me^2	1.71, s		α	4.71, m
**Aib^9**	N-H	8.33, s		β	2.16, m; 2.37, m
	α-Me^1	1.81, s		γ	2.49, m; 3.20, m
	α-Me^2	1.79, s		β'	4.89, m
				CON$\underline{H}$	9.14, d (3.9)
				OCH_3	3.29, s

Table S16. ^{13}C (225 MHz) data for compound **10** in pyridine-d_5

	Position	δ_C		Position	δ_C		Position	δ_C
**Ac**	CH_3	22.7	**Gln**[6]	C=O	174.2	**Pro**[13]	C=O	174.5
	C=O	171.9		α	57.4		α	63.9
**Phe**[1]	C=O	174.1		β	27.1		β	28.9
	α	57.0		γ	32.6		γ	26.2
	β	37.5		$\underline{C}ONH_2$	174.6		δ	49.3
	1	137.5	**Aib**[7]	C=O	177.0	**Val**[14]	C=O	173.3
	2	129.3		α	56.7		α	62.5
	3	128.6		α-Me[1]	26.8		β	29.3
	4	126.9		α-Me[2]	22.7		γ_1	19.7
	5	128.6	**Aib**[8]	C=O	176.4		γ_2	19.2
	6	129.3		α	56.9	**Aib**[15]	C=O	175.2
				α-Me[1]	27.0		α	57.1
				α-Me[2]	22.7		α-Me[1]	28.0
**Gly**[2]	C=O	171.3	**Aib**[9]	C=O	177.7		α-Me[2]	23.8
	α	44.0		α	56.9	**Ile**[16]	C=O	172.1
**Ala**[3]	C=O	175.1		α-Me[1]	27.1		α	58.8
	α	52.0		α-Me[2]	23.5		β	36.9
	β	16.6	**Ser**[10]	C=O	171.2		γ_1	25.3
**Aib**[4]	C=O	175.8		α	60.3		γ_2	16.4
	α	56.7		β	62.0		δ	11.9
	α-Me[1]	26.3	**Leu**[11]	C=O	173.5	**cycGlu**[17]	α	46.5
	α-Me[2]	23.3		α	52.0		β	22.2
**Aib**[5]	C=O	177.0		β	40.2		γ	28.0
	α	56.6		γ	24.7		δ	171.3
	α-Me[1]	26.2		δ_1	20.8		β′	85.9
	α-Me[2]	22.7		δ_2	23.3		OCH_3	54.5
			**Aib**[12]	C=O	175.1			
				α	57.1			
				α-Me[1]	27.0			
				α-Me[2]	23.4			

Table S17. ^{1}H (600 MHz) and ^{13}C (150 MHz) data for compound **12** in pyridine-d_5

	Position	δ_C	δ_H		Position	δ_C	δ_H
**Ac**	CH_3	23.1	2.07, s	**Ile⁶**	N-H	-	7.96, m
	C=O	171.7	-		C=O	173.5	-
**Aib¹**	N-H	-	9.55, s		α	62.5	4.14, m
	C=O	177.4	-		β	35.5	2.28 (m)
	α	56.7	-		γ_1	26.5	1.40, m; 1.98, m
	α-Me¹	26.5	1.63, s		γ_2	15.2	1.16, m
	α-Me²	24.2	1.73, s		δ	10.7	0.94, t (7.3)
**Ile²**	N-H	-	8.92, m	**Aib⁷**	N-H	-	8.18, s
	C=O	172.6	-		C=O	176.2	-
	α	61.3	4.22, m		α	56.6	-
	β	35.6	1.99, m		α-Me¹	26.7	1.80, s
	γ_1	26.8	1.33, m; 1.69, m		α-Me²	23.3	1.80, s
	γ_2	15.6	1.01, m	**Ala⁸**	N-H	-	8.32, m
	δ	11.1	0.79, t (7.3)		C=O	173.5	-
**Aib³**	N-H	-	8.31, s		α	52.5	4.50, m
	C=O	177.1	-		β	17.0	1.73, m
	α	56.7	-	**Aib⁹**	N-H	-	8.12, s
	α-Me¹	26.8	1.73, s		C=O	176.2	-
	α-Me²	23.1	1.75, s		α	56.5	-
**Tyr⁴**	N-H	-	8.02, d (6.2)		α-Me¹	26.9	1.96, s
	C=O	173.4	-		α-Me²	24.4	1.95, s
	α	59.3	4.58, m	**Aibal¹⁰**	N-H	-	8.07, s
	β	36.7	3.44, m; 3.51, m		HC=O	201.8	9.89, s
	γ	127.9	-		α	59.2	-
	δ	130.5	7.47, m		α-Me¹	21.7	1.65, s
	ε	116.1	7.16, m		α-Me²	21.6	1.59, s
	ζ	157.7	-				
**Aib⁵**	N-H	-	8.33, s				
	C=O	176.6	-				
	α	56.6	-				
	α-Me¹	26.7	1.84, s				
	α-Me²	23.2	1.76, s				
